# Multiomics global landscape of stemness-related gene clusters in adipose-derived mesenchymal stem cells

**DOI:** 10.1186/s13287-020-01823-3

**Published:** 2020-07-22

**Authors:** Guan-Ming Lu, Yong-Xian Rong, Zhi-Jie Liang, Dong-Lin Hunag, Yan-Fei Ma, Zhi-Zhai Luo, Fang-Xiao Wu, Xin-Heng Liu, Yu Liu, Steven Mo, Zhong-Quan Qi, Hong-Mian Li

**Affiliations:** 1 Department of Breast and Thyroid Surgery, Affiliated Hospital of Youjiang Medical University for Nationalities, Baise, 533000 Guangxi China; 2 Department of Burn and Plastic Surgery, Guiping People’s Hospital, Guigping, 537200 Guangxi China; 3 Department of Plastic and Aesthetic Surgery, The Fifth Affiliated Hospital of Guangxi Medical University &The First People’s Hospital of Nanning, Nanning, 530022 Guangxi China; 4 Medical College of Guangxi University, Nanning, 530004 Guangxi China; 5 Nanning Qiuzhijian Biotechnology Co., Ltd., Nanning, 530229 Guangxi China

**Keywords:** Multiomics global landscape, Adipose-derived mesenchymal stem cells, AD-MSCs, Stemness gene clusters, Short time-series miner analysis

## Abstract

**Background:**

Adipose-derived mesenchymal stem cells (AD-MSCs) are a type of stem cell that is abundant and widely used. The molecular characteristics of AD-MSCs from different passages from donors of different ages have not been well elucidated.

**Methods:**

Six kinds of AD-MSCs ((E1, E2, E3, Y1, Y2, and Y3) with E denoting cells derived from an elderly patient, Y denoting cells derived from a young patient, and 1, 2, and 3 representing passages 3, 6, and 10) were obtained from human abdominal adipose tissue. We obtained the protein expression profile, the mRNA expression profile, the lncRNA expression profile, and the methylation profile of each kind of AD-MSC by sequencing. After calculating the stemness indices, genes related to stemness were extracted. The multiomics correlation analysis was performed in the stemness-related genes. In addition, short time-series expression miner (STEM) analysis was performed for all cell passages and donor ages. To further explore the biological functions of the stemness-related genes, we performed Gene Ontology (GO) and Kyoto Encyclopedia of Genes and Genomes (KEGG) enrichment analyses. Finally, the lncRNA-KEGG network and transcription factor (TF)-KEGG network were constructed based on the RNAInter database and TRRUST v2 database.

**Results:**

The stemness of the Y1, E1, and Y2 cells was higher than that of the E2, Y3, and E3 cells. The stemness was the highest for Y1 cells and the lowest for E3 cells. STEM analysis showed that five stemness-related gene clusters were associated with the cell passages, and only one gene cluster was associated with age. The enrichment analysis results showed that the biological processes (BPs) and KEGG pathways were mainly involved in the proliferation, differentiation, and migration of cells. The global regulatory landscape of AD-MSCs was constructed: 25 TFs and 16 lncRNAs regulated 21 KEGG pathways through 27 mRNAs. Furthermore, we obtained a core stemness-related gene set consisting of ITGAV, MAD2L1, and PCNA. These genes were expressed at higher levels in Y1 cells than in E3 cells.

**Conclusion:**

The multiomics global landscape of stemness-related gene clusters was determined for AD-MSCs, which may be helpful for selecting AD-MSCs with increased stemness.

## Introduction

When used for different therapeutic procedures aimed at treating various forms of tissue damage, the autologous fat graft is indeed a solution. Recent studies have shown that adipose-derived stromal vascular fractions (AD-SVFs) [1, 2] and adipose-derived stem cells (ASCs) [3, 4] can enhance tissue regeneration potential [5]. ASCs are localized in AD-SVFs, which have a heterogeneous mesenchymal cell set [6] and can be obtained by 2 methods: enzymatic digestion and mechanical filtration [7, 8]. ASCs have the ability to differentiate into cells of mesenchymal origin in vitro; this includes osteoblasts, adipocytes, and chondrocytes. They can also enhance different tissues in vivo, including cartilage, muscle, bone, and fat [5]. As shown in previous studies, adipose-derived human follicle stem cells (AD-HFSCs) are helpful for hair regrowth [9]. Adipose-derived mesenchymal stem cells (AD-MSCs) contribute to wound healing and soft tissue defects [8]. In summary, SVFs and AD-MSCs are ideal tools for generating medicine.

Some studies have shown that platelet-rich plasma (PRF) and hyaluronic acid (HA) dressings alone or in combination are also helpful to promote tissue repair [10–12]. PRF can promote tissue repair in two ways by providing a bridge for tissue incorporation and new blood vessel formation [13, 14] as well as producing growth factors (b-FGF, PDGF, VEGF, EGF, TGF-β, and IGF-1) [15]. When AD-MSCs are injected into damaged parts of the human body, they can promote tissue regeneration through their own proliferation and differentiation. Furthermore, growth factors can accelerate this process by driving cell growth [16]. Interestingly, AD-SVFs/AD-MSCs/fat grafts are used in combination with PRF to help improve wound healing and soft tissue defects [16, 17]. More importantly, some studies reported that the Wnt/TGF-β/β-catenin signaling pathway is necessary for the growth of cells in wound healing and soft tissue defects [9, 18, 19].

The stemness of AD-MSCs is mainly reflected in their ability to maintain self-renewal, cell differentiation, and proliferation [20]. A previous study showed that the differentiation capacity is maintained with aging; however, AD-MSCs from younger donors may exhibit a higher proliferation rate [21]. In addition, long-term cell culture with serial passaging may negatively affect the stemness of stem cells [22]. These findings indicate that the stemness of AD-MSCs is influenced by the ages of donors and the number of cell passages. A previous study indicated that CD13, CD29, CD44, CD73, CD90, CD105, and CD106 in AD-MSCs are upregulated in expansion culture compared to those in the stromal vascular fraction (SVF) [23, 24]. In addition, the senescence-related proteins p53, p21, and p16 are strongly expressed [25]. These studies show that AD-MSCs from different passages may present different molecular characteristics. However, the molecular characteristics of AD-MSCs from different passages from donors of different ages have not been well elucidated.

This study attempts to elucidate the multiomics and molecular characteristics of AD-MSCs derived from donors of different ages and from different cell passages. We found that the stemness of AD-MSCs from younger donors was increased. In addition, the stemness of early-passage (passage 3) AD-MSCs was the highest compared with that of intermediate-passage (passage 6) and late-passage (passage 10) AD-MSCs. These findings may provide guidance on how to select AD-MSCs with increased stemness, as well as whether AD-MSCs should be injected directly or post-expanded.

## Methods

### Sample acquisition and cell culture

**Fig. 1:**
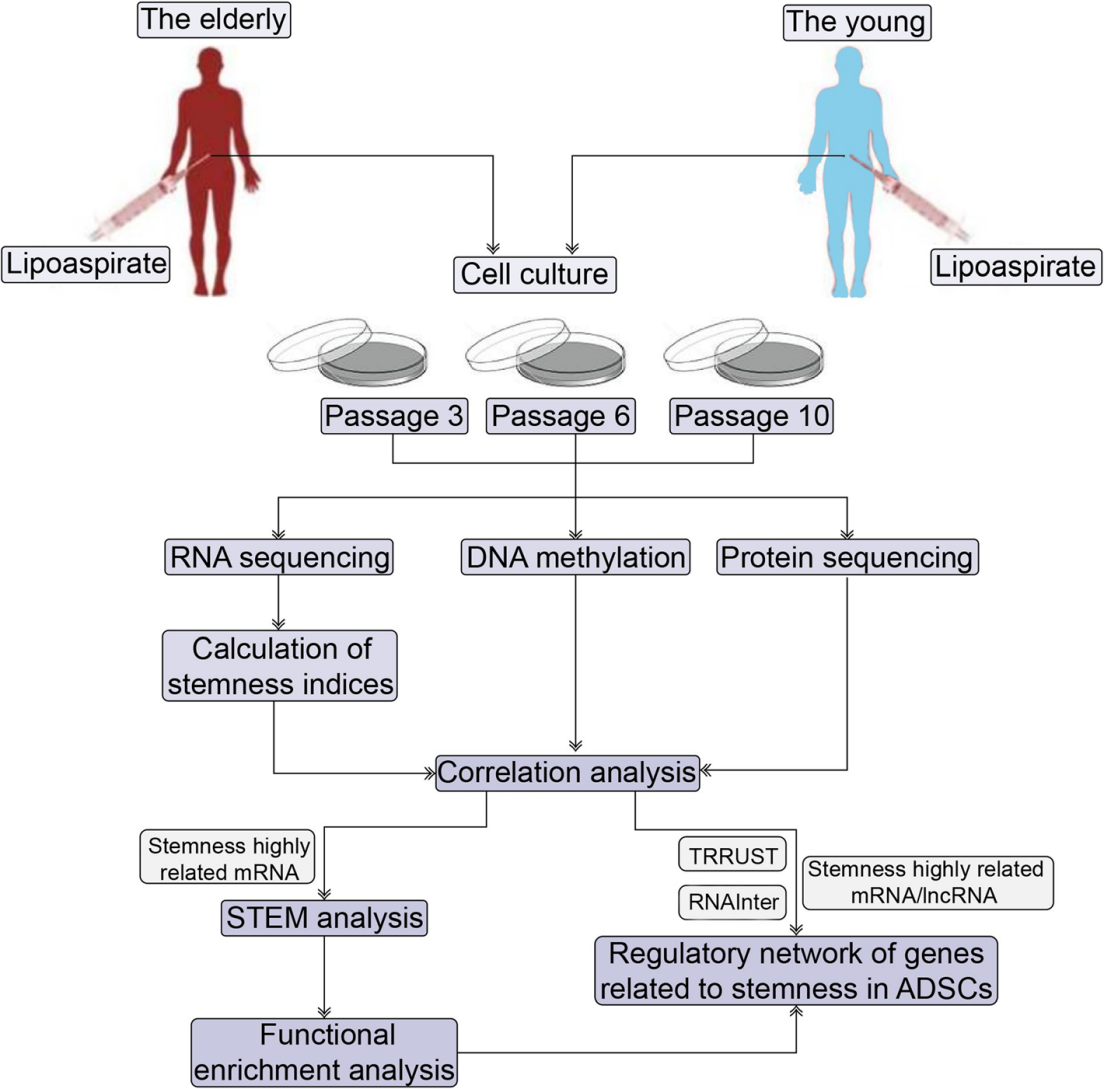
The workflow of the study

The study was performed with the permission of the ethics committee of the First People’s Hospital of Nanning, Guangxi Zhuang Autonomous Region. We obtained written informed consent from donors. Human lipoaspirate from the abdominal subcutaneous tissue of healthy females (one young patient who was 27 years old and an elderly patient who was 63 years old) was stored for less than 48 h at 4 °C before processing. The adipose tissue (AT) was gently aspirated using 10-ml Luer-Lok syringes and then placed in a tube, which was centrifuged for 3 min at 1200*g*. Then, we added collagenase solution (type II; Worthington Biochemical Corp., Lakewood, N.J.) to the AT, which was shaken gently in a 37 °C water bath to digest the adipose tissue. The digested AT was centrifuged again for 10 min and then filtered to remove the debris at 180*g*. Next, erythrocyte lysis buffer was added to the cell pellet (stromal vascular part), which was resuspended and centrifuged for 10 min at 180*g*. The stromal vascular fraction was plated and cultured in tissue culture-treated flasks in adipose-derived stem cell plating medium (0.001% dexamethasone, Dulbecco’s Modified Eagle Medium/Nutrient Mixture F12, 10% fetal bovine serum, and 1% Fungizone (Bristol-Myers Squibb, New York, N.Y.) and 1% penicillin/streptomycin. After overnight incubation, the nonadherent cells were removed, and the medium was changed to fresh medium. After 20 to 48 h, the cells were expanded until they were nearly confluent to obtain the passage 0 AD-MSCs. The expansion rate and morphology of the AD-MSCs were observed under a microscope. When the AD-MSCs were at a confluence of 80% to 90%, they were passaged at a ratio of 1:3. The cell number of passage 0 AD-MSCs was 1 × 10^6^ and 2 × 10^6^ in young patient and the elderly patient, respectively. When amplifying passages 3, 6, and 10, the cell number was 1 × 10^8^, 1 × 10^10^, and 2 × 10^12^ in the young patient. While in the elderly patient, the cell number was 1 × 10^8^, 5 × 10^9^, and 2 × 10^11^ when amplifying passage 3, 6, and 10. Subsequently, 1 × 10^8^ cells from each kind AD-MSCs were collected for sequencing. AD-MSCs from the two patients were harvested at three time points during amplification for multiomics analysis to obtain the early-passage (passage 3), intermediate-passage (passage 6), and late-passage (passage 10) cells. A total of 6 types of AD-MSCs (E1, E2, E3, Y1, Y2, and Y3) were obtained; E represents the elderly patient, Y represents the young patient, and 1, 2, and 3 represent passages 3, 6, and 10. The workflow of this study is shown in Fig. 1.

### RNA extraction and sequencing

All RNA was isolated from AD-MSCs by TRIzol purification (Invitrogen), and genomic DNA was removed with the help of gDNA eliminator columns from the RNeasy Mini Kit (Qiagen). In addition, spectrophotometry (NanoDrop, Thermo Fisher Scientific) was used to assess the RNA quality along with a Bioanalyzer (Agilent). The RNA was enriched for mRNA (Dynabeads mRNA purification kit, Invitrogen) by poly(A) isolation, and the mRNA was fragmented and subjected to first-strand cDNA synthesis. After second-strand cDNA synthesis, the double-stranded cDNA was quantified and purified. The library was constructed by using a protocol from Illumina with the NEBNext DNA Sample Prep Reagent kits (NEB). The double-stranded cDNA was end-repaired, and the adapters were ligated to the DNA fragments; poly(A) tracts RNA sequences were then added. After size selection (200~600 bp) and UDGase treatment to ensure strand specificity, the adapter-modified DNA fragments were amplified by PCR. Next, 76-base paired-end sequencing was performed on an Illumina instrument.

The strand-specific paired-end reads were screened for ribosomal RNA by alignment against known rRNA sequences (RefSeq) using Bowtie software [26]. The rRNA-subtracted paired-end reads were aligned with TopHat [27]. The maximum multibit was set to 1 m, and a micro-exon search was performed. In addition, a gene model was provided as a gene transfer format (GTF) file (Ensembl BDGP5.25.60). The aligned reads in valid pairs were subjected to FPKM estimation through Cuffilinks. Correction and bias detection were performed at this step, which only retained fragments compatible with Ensembl annotation (BDGP5.25.60). These reads were counted toward the number of mapped hits used in the FPKM denominator.

### DNA methylation assay

LCM-DNAs from AD-MSCs were fragmented into 100–500 bp fragments. Then, the genomic DNA was extracted from the AD-MSCs using a high-salt procedure, and the HELP assay was performed as previously described. The assay involved interrogating the cytosine methylation status and performing comparative isoschizomer profiling on a genomic scale. The genomic DNA from AD-MSCs was digested by HpaII (a methylcytosine-sensitive enzyme) and MspI, and then the MspI- and HpaII-produced fragments were amplified through PCR mediated by ligation. Both of the amplified fractions were submitted to Roche-NimbleGen, Inc. (Madison, WI). Then, these fractions were labeled and hybridized with a human hg17 custom-designed oligonucleotide array (50-mers) covering 25,626 HpaII-amplifiable fragments (HAFs), which were located at gene promoters. HpaII-amplifiable fragments are defined as genomic sequences. Each fragment on the array was represented by 15 individual probes distributed randomly throughout the microarray slide. The microarray covered 50,000 CpGs corresponding to 14,000 gene promoters. The signal intensities were calculated as the robust mean of their component probe-level signal intensities after intensive quality control. The log_2_(HpaII/MspI) value represented methylation and was analyzed as a continuous variable. For most loci, each fragment was categorized as either hypomethylated if the log ratio was greater than zero or methylated if the centered log HpaII/MspI ratio was less than zero. The average methylation level for all CpG islands in a gene was considered the methylation level of the corresponding gene in the present study.

### Proteome sequencing

Each sample was homogenized with 5 volumes of glass sand. Then, 200 μl radioimmunoprecipitation assay (RIPA) buffer containing phenylmethylsulfonyl fluoride (PMSF) was added to lyse the cells. Then, the samples were sonicated. All soluble proteins were collected after centrifugation at 13,400*g* for 20 min. The concentration of the protein was measured by a quantitative protein kit (2D-quant-kit, GEHealthcare).

One hundred micrograms of protein was reduced, alkylated, and precipitated by the chloroform precipitation method to digest the protein. First, 55 mM Reducing Reagent (8-plex iTRAQ kit, AB Sciex, USA) was added to the proteinase and incubated at 60 °C for 60 min. Then, the protein was mixed with Cysteine Blocking Reagent (8-plex iTRAQ kit, AB Sciex, USA). After deionized water and 70% ethanol were added to the 10 KD ultrafiltration cartridge, the protein solution was poured into the ultrafiltration cartridge. The solution was centrifuged for 20 min at 13,400*g*. The ultrafiltration cartridge was centrifuged in 0.25 M TEAB (triethylammonium bicarbonate). The protein pellets were reconstituted in 0.25 M urea/50 mM TEAB and digested with 2% trypsin overnight (Promega).

A high-performance liquid chromatography (HPLC) system (Phenomenex columns; Gemini-NX 3u C18 110A; 150*2.00 mM) was used to perform strong cation exchange (SCX) fractionation chromatography. The isobaric tags for relative and absolute quantification (iTRAQ)-labeled peptides were separated with a linear gradient formed by mobile phase B (80% ACN, 20 mM HCOONH_4_, pH 10) and mobile phase A (20 mM HCOONH_4_, pH 10). The flow for peptide elution was set to a rate of 20 μl/min. The fractions were collected and acidified with trifluoroacetic acid (50%). The fractions were vacuum-dried prior to LC-MS/MS. The fractions were dissolved in buffer and pelleted. The supernatant was loaded onto analytical columns and identified with a Q Exactive system (Thermal Scientific). The components of the mobile phase used for LC-MS were formic acid (0.1%) and 80% CAN. The flow rate of the analytical columns was set at 350 nl/min. The peptides were analyzed by using a 3-step gradient for 65 min. The parameters of the first-grade MS were a maximum injection time of 40 ms and a resolution of 70,000. The scan range was from 350 to 1800 m/z. The second-grade MS spectra were acquired at a resolution of 17,500. The top 20 precursors were selected for each MS cycle.

### Data preprocessing

The raw counts of the mapped reads were aggregated using featureCounts [28]. The gene-level quantification was performed with a gene transfer format (GTF) file (UCSC Genome Bioinformatics: Frequently Asked Questions: Data File Forma. https://genome.ucsc.edu/FAQ/FAQformat.html#format4. Accessed on 12 January 2016.). Then, to obtain the mRNA/lncRNA expression profiles, the data were normalized using the “*voom*” function of the limma package [29] in R. The protein expression profile and the methylation profile were constructed according to the proteome sequencing data and the methylation sequencing data, respectively, without standardization.

### Calculation of stemness

Stemness is considered to represent the potential for differentiation and self-renewal of the cell of origin, which possesses the ability to produce all cell types in the adult organism [30]. The TCGAbiolinks package [31] in R was used to calculate the stemness for the six kinds of AD-MSCs based on mRNA expression using the messenger RNA (mRNA) expression profiles.

### Correlation analysis

The correlation between stemness and mRNA/long noncoding RNA (lncRNA) expression/protein expression was used to identify mRNAs/lncRNAs/proteins that are highly related to stemness. The correlation between stemness and mRNA/lncRNA/DNA methylation was also explored. The Pearson correlation coefficient (*r*) was calculated using the *Hmisc* package [32] in R. *P* < 0.01 and *r* > 0.9 were considered to indicate a strong correlation. The correlation among coding gene (mRNA) DNA methylation, protein expression, and corresponding mRNA expression and the long noncoding gene (lncRNA) DNA methylation and corresponding lncRNA expression were also explored.

### Short time-series expression miner (STEM) analysis

The mRNAs highly related to stemness were used to perform STEM analysis using STEM v1.3.8 [33]. The mRNAs were organized into different profiles (clusters) based on expression patterns using STEM analysis. Donor age (young and elderly) and the AD-MSCs passage number (passages 3, 6, and 10) were considered time points.

### Functional enrichment analysis and gene set variation analysis (GSVA)

To explore the biological functions of the stemness-related gene profiles, Kyoto Encyclopedia of Genes and Genomes (KEGG) and Gene Ontology (GO) enrichment analyses were performed using the *clusterProfiler* package [34] in R. Then, the most significant (ranked by *P* value) biological processes (BPs) and KEGG pathways were visualized with a bubble diagram. GSVA [35] was used to score the individual samples according to the BPs and KEGG pathways, and each sample received a GSVA score. In addition, KEGG and BP enrichment analysis were also performed with the *ClueGO* plug-in [36] in *Cytoscape* software [37]. The GO and KEGG networks were used to determine the relationships between the GO terms according to the similarity of their related genes. A *P* value adjusted by the false discovery rate < 0.05 was considered significant.

### Stemness-related transcriptional regulatory network

Interactions between stemness-related lncRNAs and their target genes were downloaded from the RNAInter database [38]. The interactions between transcription factors (TFs) and stemness-related mRNAs were downloaded from the TRRUST v2 database [39]. Correlation analysis between the lncRNA/TFs and their targets was performed to reduce the number of false positives and noise. Subsequently, in addition to the enrichment analysis, an AD-MSCs stemness-related lncRNA/TF-target-KEGG pathway network was constructed.

## Results

### Multiomics atlas of genes related to AD-MSC stemness

**Fig. 2:**
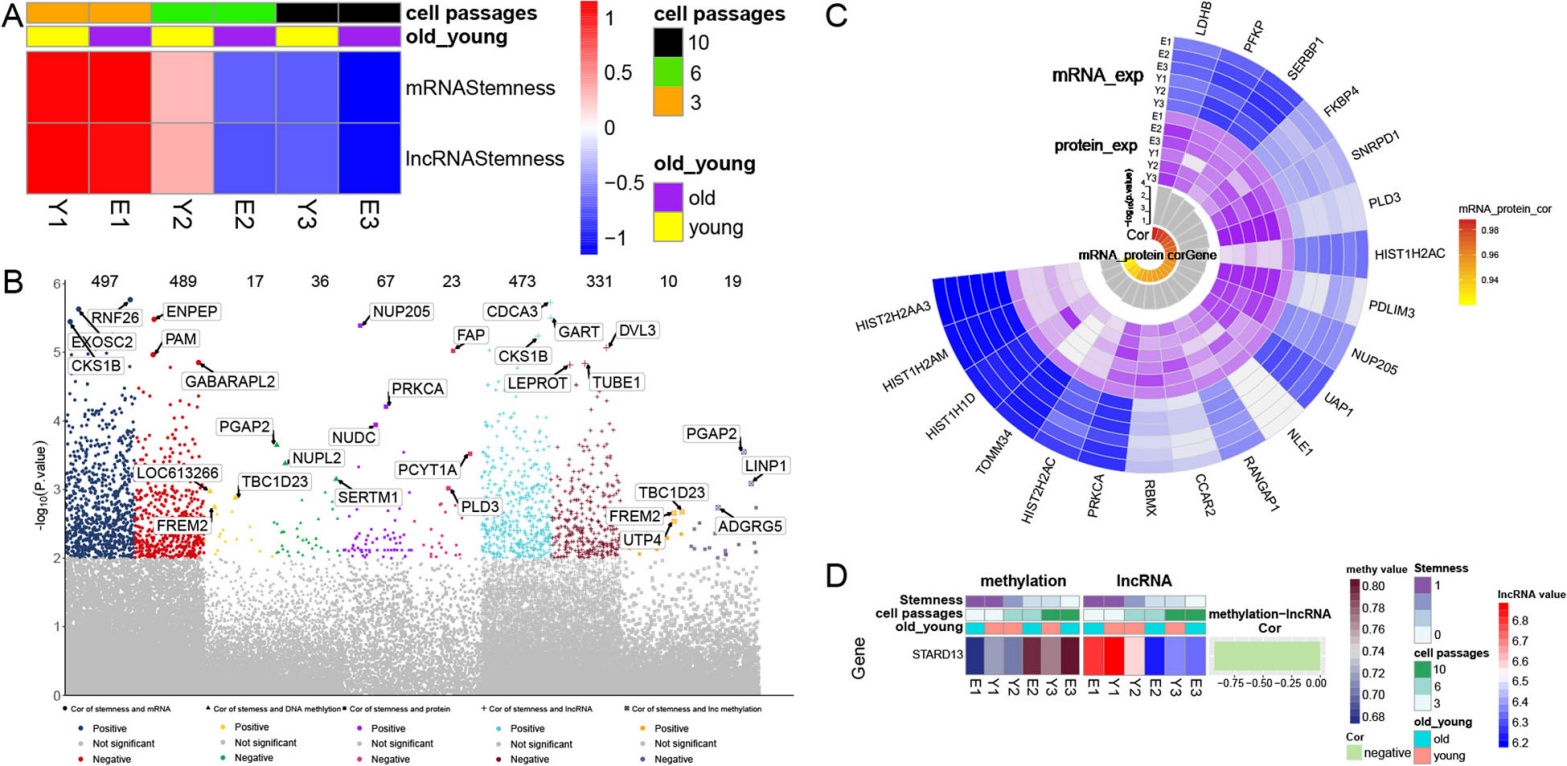
Multiomics analysis of genes related to stemness. **a** mRNA stemness indices of 6 types of AD-MSCs. **b** Manhattan plot of the differentially expressed stemness-related genes. The three most significant genes are marked. The numbers above the plot indicate the positively and negatively related genes. **c** Circos plot. Twenty genes are highly related to mRNA and protein expression. **d** Heatmap plot. STARD13 is negatively related to methylation but positively related to lncRNA expression. AD-MSCs, adipose-derived mesenchymal stem cells

A total of 19,189 mRNAs, 21,965 lncRNAs, and 3205 proteins were included in these analyses. The DNA methylation levels of 8070 mRNAs and 4308 lncRNAs were measured. Stemness was closely associated with the cell passage of the AD-MSCs in the following order of decreasing association: early passage > intermediate passage > late passage (Fig. 2a). A total of 1286 mRNAs, 90 proteins, and 804 lncRNAs were found to be highly related to stemness. The DNA methylation levels of 50 mRNAs and 29 lncRNAs were highly related to stemness (Fig. 2b). However, there were only 20 mRNAs and corresponding proteins that were highly related to stemness (Fig. 2c). In addition, the expression and DNA methylation level of the lncRNA STARD13 were both highly related to stemness. The expression of STARD13 was positively related to stemness, while the methylation level of STARD13 DNA was negatively related to stemness (Fig. 2d).

### Clusters of stemness-related genes associated with AD-MSC passage or donor age

**Fig. 3:**
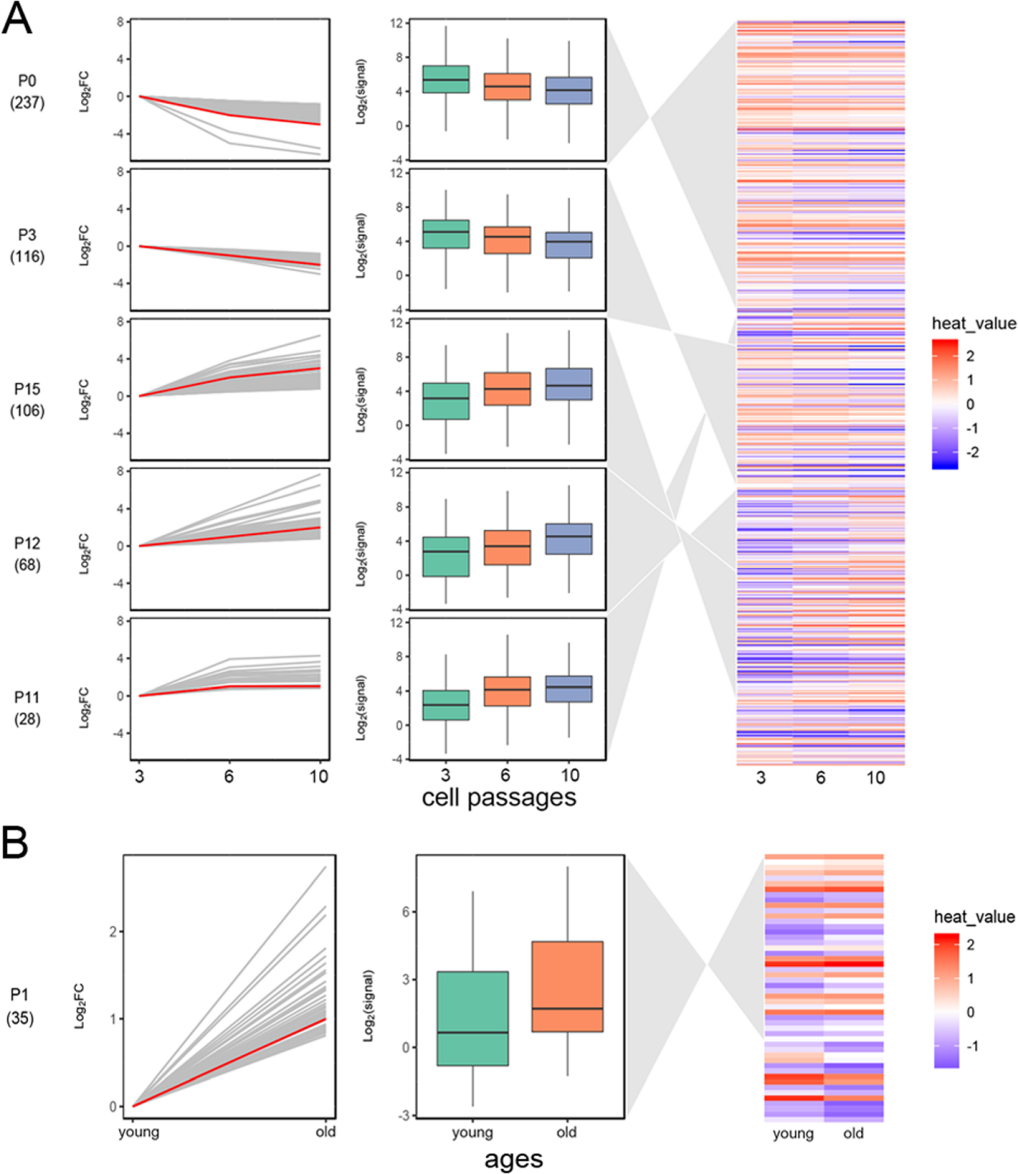
STEM analysis of genes related to stemness. **a** STEM analysis of AD-MSCs from different passages. **b** STEM analysis of ages. The diagram in the left panel represents log_2_|(fold change)|, and the fitted curve is in red. The figure in the middle panel shows the expression of each gene cluster. The plot in the right panel shows the distribution of representative genes. STEM, short time-series expression miner

In the STEM analysis, although 15 mRNA profiles were identified, only 5 mRNA profiles including a total of 555 genes were considered significant (Table S1). Two mRNA profiles (0 and 3) showed significantly gradually upregulated expression patterns that occurred during cell proliferation and passaging. Three mRNA profiles (11, 12, and 15) showed significantly gradually downregulated expression patterns (Fig. 3a). An mRNA profile comprising 35 genes (Table S2) was identified as representing expression patterns that were significantly upregulated with age (Fig. 3b).

### Stemness-related gene clusters involved in multiple functional pathways

**Fig. 4:**
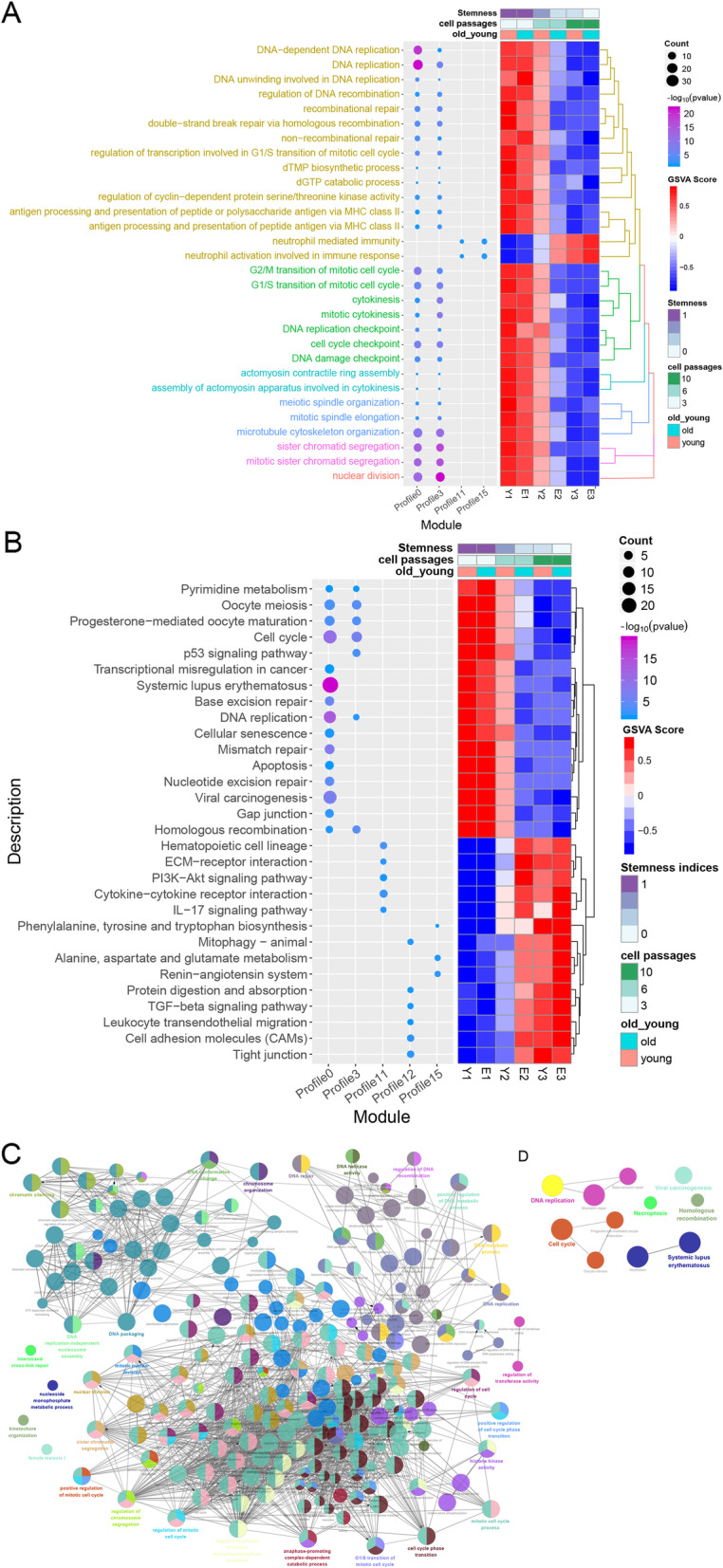
Biological processes and pathways of the stemness-related genes in cells from different passages of AD-MSCs. **a** GO cluster-GSVA heatmap. The GO_BPs with more than 1 functional module were significantly enriched. **b** Bubble-GSVA heatmap. The KEGG pathways with more than 1 functional module were significantly enriched. **c** GO_BP network for different passages of AD-MSCs. **d** KEGG pathway network for different passages of AD-MSCs. Each node indicates a representative enrichment pathway. The connection of the nodes represents the number of genes in common between the pathways. The color represents the enrichment classification of the nodes. AD-MSCs, adipose-derived mesenchymal stem cells; GO, Gene Ontology; KEGG, Kyoto Encyclopedia of Genes and Genomes; BPs, biological processes

The five AD-MSCs passage-related mRNA profiles, which included 555 genes, were used to perform functional enrichment analysis. The enrichment results showed that there were 2032 associated BPs (Table S3) and 56 associated KEGG pathways (Table S4). Unsurprisingly, multiple BPs, such as the PI3K-Akt signaling pathway [40], p53 signaling pathway [41], and apoptosis [42] (Fig. 4a), were mainly related to stem cells. The genes in profiles 11 and 15 were involved in some BPs related to the immune response and were negatively related to stemness. The five AD-MSCs passage-related mRNA profiles were involved in proliferation-, differentiation-, and migration-related pathways (Fig. 4b). In the *ClueGO* analysis, a total of 218 BPs were identified. The results of the *ClueGO* analysis also suggested that the stemness-related mRNA profiles were associated with cell proliferation-related BPs, such as DNA replication, the cell cycle phase transition, and regulation of the cell cycle (Fig. 4c). In addition, the stemness-related mRNA profiles were involved in DNA replication, necroptosis, the cell cycle, systemic lupus erythematosus, and homologous recombination pathways (Fig. 4d).

### Global regulatory landscape of stemness-related gene clusters at the multiomics level

**Fig. 5:**
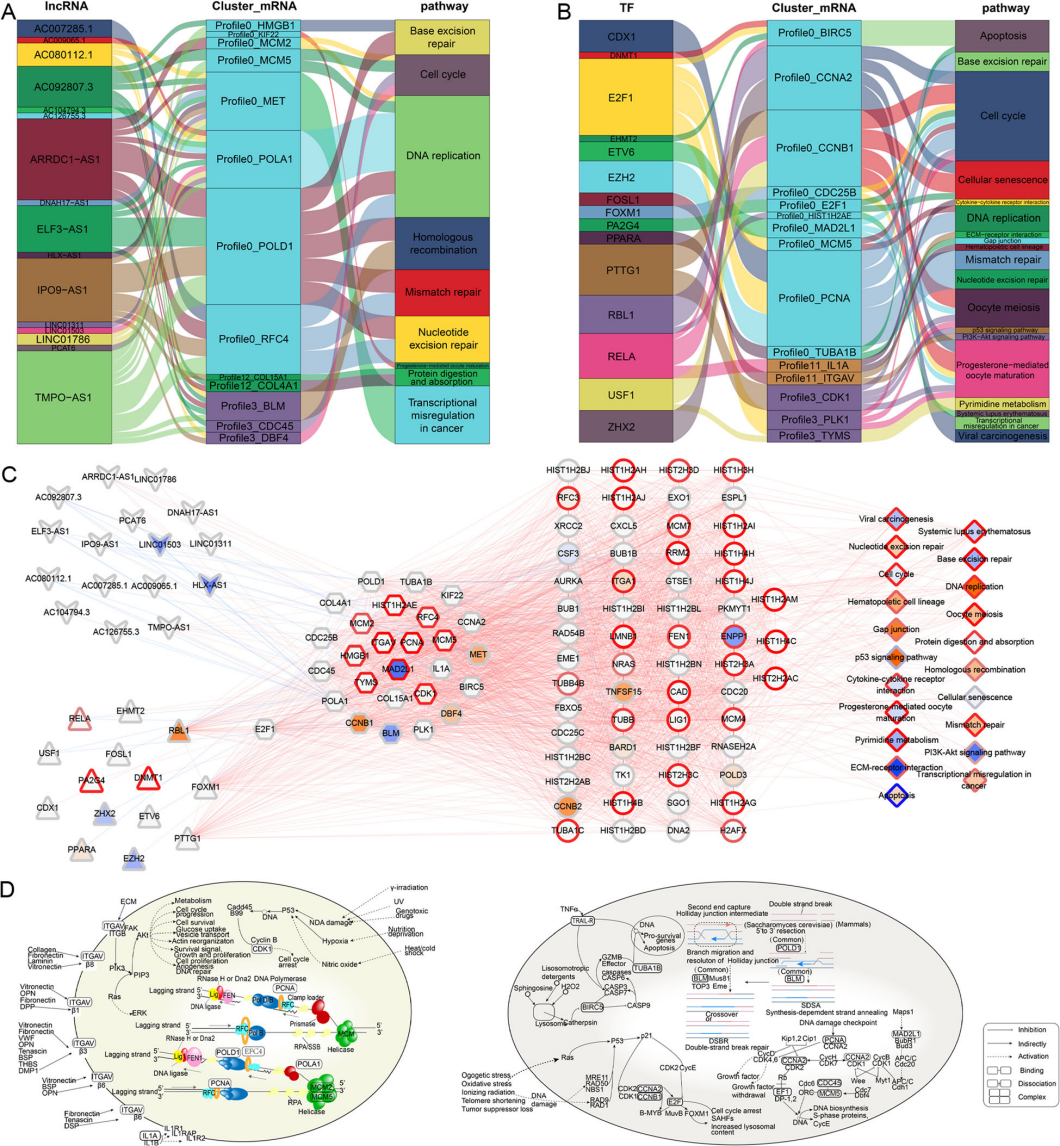
Global regulatory landscape of the stemness-related gene clusters at the multiomics level. **a** Graphical summary of the lncRNAs associated with the stemness regulation of KEGG pathways through mRNAs. **b** Graphical summary of the transcription factors regulating KEGG pathways through mRNAs. **c** Comprehensive regulatory network of lncRNAs associated with stemness and transcription factors regulating KEGG pathways through mRNAs. **d** Nine pathways of interest in AD-MSCs. The triangles represent TFs. Circles represent KEGG-related genes. The squares represent the KEGG pathways. The kernels show the correlations between methylation and the transcriptome. The borders show the correlations between RNAs and proteins. The edges show the RNA correlations between nodes

The interactions of stemness-related lncRNAs and mRNAs were extracted from the RNAInter database, and the interactions of TFs and stemness-related mRNAs were extracted from the TRRUST v2 database. By combining these interactions with the enrichment analysis, sixteen stemness-related lncRNAs were implicated in nine KEGG pathways by regulating 13 stemness-related mRNAs in profiles 0, 3, and 12 (Fig. 5a). Fifteen TFs that may regulate 15 stemness-related mRNAs in profiles 0, 3, and 11 were involved in 19 KEGG pathways (Fig. 5b). Subsequently, the AD-MSCs stemness-related lncRNA/TF-target-KEGG pathway networks were constructed and included 16 lncRNAs, 15 TFs, 27 mRNAs, and 21 KEGG pathways (Fig. 5c). Previous studies proposed that the pathways involving AD-MSCs are mainly involved in differentiation, proliferation, and migration [43]. Therefore, we found 9 pathways of interest for further study, including DNA replication, cell cycle, homologous recombination, cellular senescence, apoptosis, PI3K-Akt signaling, ECM-receptor interaction, cytokine-cytokine receptor interaction, and p53 signaling pathways. In DNA replication, the MCM complex unwinds the double-strand parental DNA. Subsequently, with the help of DNA polymerase (POLA1 and POLD1), the DNA clamp (PCNA), and the clamp loader (EFC4), the new DNA is synthesized. We found that CDK1 could affect the cell cycle in the p53 signaling pathway. In addition, the specific interaction between cells and ECMs is mediated by integrins containing ITGAV via the cytokine-cytokine receptor interaction and extracellular matrix (ECM)-receptor interaction. In apoptosis, BIRC5 and TUBA1B could be affected, thereby affecting cell apoptosis. In homologous recombination, BLM is helpful to maintain genome integrity. In cellular senescence, the p21 complex containing CCNA2 and CCNB1 may be affected as well as its downstream regulator (E2F1). Eventually, the cell cycle is affected. In the cell cycle, the Maps1 complex containing MAD2L1 and the complex containing PCNA are affected by the DNA damage checkpoint; therefore, CCNA2 is affected (Fig. 5d).

### Multiomics characteristics of core stemness-related genes and their lncRNA regulators

**Fig. 6:**
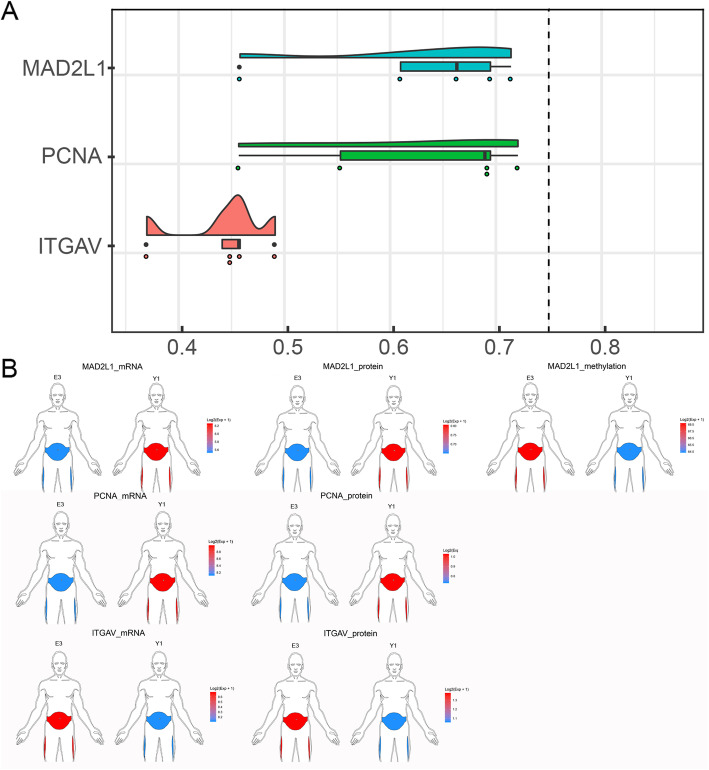
Multiomics characteristics of core stemness-related genes and their lncRNA regulators. **a** Three genes with the highest average semantic similarity. **b** Expression of core stemness-related genes in the mRNA expression profile, protein expression profile, and methylation profile in E3 and Y1 cells

Forty genes were shown to be highly related to both mRNA and protein expression. Three genes were identified by combining pathways of interest, including ITGAV, MAD2L1, and PCNA. These genes were defined as the core stemness genes. In addition, the 3 genes with the highest similarity were ITGAV, MAD2L1, and PCNA (Fig. 6a), which further verified our findings. Among them, only MAD2L1 had a methylation profile. As shown in Fig. 2, the stemness of AD-MSCs was the highest for Y1 cells and the lowest for E1 cells. Therefore, Y1 and E1 cells were chosen to determine the expression of the core stemness genes. Compared to those of E1 cells, the mRNA and protein expression profiles of Y1 cells showed that MAD2L1 was upregulated, but MAD2L1 was downregulated according to the methylation profile. Compared to E1 cells, Y1 cells showed the upregulation of PCNA according to the protein and mRNA expression profiles. The expression profile of ITGAV was exactly the opposite of that of PCNA (Fig. 6b).

## Discussion

AD-MSCs, which have a high potential for differentiation into many kinds of mature cells in an appropriate environment, are considered to be an ideal treatment in regenerative therapies [43]. In the current study, we found that stemness of AD-MSCs is closely associated with the cell passage in the decreasing order of early passage > intermediate passage > late passage. The potential biological functions of stemness-related mRNAs were highly related to proliferation, differentiation, and migration. In addition, we explored the regulatory network of AD-MSCs.

In this study, we found that the stemness of Y1, E1, and Y2 cells was higher than that of E2, Y3, and E3 cells. This is consistent with previous studies, which showed that as the age and the number of cell passages increased, the proliferation and differentiation potential of AD-MSCs decreased [44, 45]. In addition, 5 stemness gene clusters were obtained, which showed that the maintenance of stemness in AD-MSCs was the result of the interaction of multiple genes.

The comprehensive regulatory network indicated that lncRNAs and TFs could influence mRNA expression in AD-MSCs, thereby affecting their phenotype. Among the 9 pathways that were analyzed further, some had been reported in previous studies related to stem cells, such as the cell cycle [46], homologous recombination [47], apoptosis [42], PI3K-Akt signaling [40], and p53 signaling pathways [41]. In addition, we found that DNA replication, cellular senescence, cytokine-cytokine receptor interactions, and ECM-receptor interactions were related to stem cells.

Furthermore, we found that the genes in the above pathways were widely involved in the regulation of the cell cycle and intercellular functions in cells in the current study. MCM5 [48] and CCNA2 [49] play roles in the regulation of the mammalian cell cycle. In addition, MCM2 plays an important role in cell division and DNA replication [50]. Moreover, the protein encoded by PCNA plays a central role in recruiting and retaining many of the enzymes required for DNA replication and repair [51]. POLA1 [52], POLD1 [53], PCNA [54], and RFC4 [55] can participate in DNA replication by forming DNA polymerase. MAD2L1 is required for execution of the mitotic checkpoint [56]. CDK1 is a catalytic subunit of a protein kinase complex that induces cell entry into mitosis [57]. CCNB1 is expressed predominantly in the G2/M phase of cell division [58]. CDC45 is an important component of the replication fork, participating in DNA unwinding [59]. TUBA1B is involved in mitosis, cell movement, intracellular movement, and other biological processes [60]. When activated in response to DNA damage, E2F1 can promote proliferation or apoptosis [61]. BIRC5 is an inhibitor of apoptosis that can regulate apoptosis [62]. BLM is a tumor suppressor that maintains genome integrity [63]. ITGAV is a kind of α-V integrin that functions in cell surface adhesion [64]. According to our study, some of the differentially expressed genes were related to proliferation, differentiation, and metastasis in AD-MSCs.

In this study, we identified 3 core stemness-related genes (ITGAV, MAD2L1, and PCNA) that comprise a core stemness-related gene set. As shown in previous studies, MAD2L1, PCNA, and ITGAV are related to the proliferation and differentiation of cells. ITGAV is a kind of mRNA, and its product belongs to the integrin α chain family, which serves as a major receptor for differentiation and cell proliferation [64]. MAD2L1 mainly functions during mitosis to ensure that all chromosomes properly align at the metaphase plate [65]. The protein encoded by PCNA is a cofactor of DNA polymerase δ and is involved in the processes of DNA replication and DNA repair [66]. Therefore, the core stemness-related gene set identified in this study may be helpful to assess the stemness of AD-MSCs.

Our study presents several limitations. First, the study is based on bioinformatics prediction; therefore, further experimental verification is needed. In addition, the sample size involved in this study was small, and we need to verify the results in a larger data cohort later.

## Conclusion

The multiomics global landscape of stemness-related gene clusters was constructed for AD-MSCs. Moreover, we identified a core stemness-related gene set comprised of ITGAV, PCNA, and MAD2L, which may be helpful for selecting AD-MSCs with increased stemness.

## Supplementary information

**Additional file 1 : Table S1**. The 555 genes belonging to the 5 stemness-related gene clusters associated with cell passages.

**Additional file 2 : Table S2**. The thirty-five genes belonging to the single stemness-related gene cluster associated with age.

**Additional file 3 : Table S3**. GO_BPs enriched in adipose-derived mesenchymal cells. GO: gene ontology, BPs: biological processes.

**Additional file 4 : Table S4**. KEGG pathways enriched in AD-MSCs. AD-MSCs: adipose-derived mesenchymal cells.

## Data Availability

The datasets used and/or analyzed during the current study are available from the corresponding author on reasonable request.

## Abbreviations

AD-MSCs: Adipose-derived mesenchymal stem cells
GO: Gene Ontology
KEGG: Kyoto Encyclopedia of Genes and Genomes
BPs: Biological processes
ATs: Adipose tissues
SVF: Stromal vascular fraction
HAF: Gene transfer format
RIPA: Radioimmunoprecipitation assay
HPLC: High-performance liquid chromatography
SCX: Strong cation exchange
mRNA: Messenger RNA
lncRNA: Long non-coding RNA
GSVA: Gene set variation analysis
TFs: Transcription factors
ECM: Extracellular matrix
ASCs: Adipose-derived stem cells
HFSCs: Adipose-derived human follicle stem cells
PRF: Platelet-rich plasma
HA: Hyaluronic acid
min: Minute
E: Elderly
Y: Youth
PMSF: Phenylmethylsulfonyl fluoride
iTRAQ: Isobaric tags for relative and absolute quantification
LC: Liquid chromatograph
MS: Mass spectrometer
