# Supplementary material for: Multiomics global landscape of stemness-related gene clusters in adipose-derived mesenchymal stem cells
Source: Stem Cell Res Ther. 2020 Jul 22;11:310. doi: 10.1186/s13287-020-01823-3 (PMC7374825; doi:10.1186/s13287-020-01823-3)
Supplement: Supplementary file 1 — Additional file 1 : Table S1. The 555 genes belonging to the 5 stemness-related gene clusters associated with cell passages. [file 13287_2020_1823_MOESM1_ESM.docx]

| Table S1. The 555 genes belonging to the 5 stemness-related gene clusters associated with cell passages. | |
| --- | --- |
| gene | Profile |
| RACGAP1 | Profile0 |
| RFC3 | Profile0 |
| AC004223.3 | Profile0 |
| CSE1L | Profile0 |
| TPX2 | Profile0 |
| HMMR | Profile0 |
| SCARA3 | Profile0 |
| MYBL1 | Profile0 |
| NUDT1 | Profile0 |
| TUBB | Profile0 |
| TK1 | Profile0 |
| MET | Profile0 |
| CCNA2 | Profile0 |
| SLX4IP | Profile0 |
| MCM4 | Profile0 |
| CENPU | Profile0 |
| TJP2 | Profile0 |
| SPC24 | Profile0 |
| ELFN2 | Profile0 |
| CKAP2 | Profile0 |
| KCNQ5 | Profile0 |
| CCDC84 | Profile0 |
| NUP155 | Profile0 |
| TUBA1C | Profile0 |
| ZNF367 | Profile0 |
| CKS1B | Profile0 |
| FOXRED2 | Profile0 |
| NR2F2 | Profile0 |
| HIST1H2BI | Profile0 |
| RNF26 | Profile0 |
| HIST1H2AE | Profile0 |
| MRPS6 | Profile0 |
| ZNF670 | Profile0 |
| NUDT11 | Profile0 |
| CRX | Profile0 |
| ITGA6 | Profile0 |
| MCM5 | Profile0 |
| WDR34 | Profile0 |
| HIST1H4J | Profile0 |
| EXOSC2 | Profile0 |
| RAD18 | Profile0 |
| SRSF7 | Profile0 |
| FLRT3 | Profile0 |
| POLR3G | Profile0 |
| NEK2 | Profile0 |
| SHMT1 | Profile0 |
| STMN1 | Profile0 |
| CDCA8 | Profile0 |
| KCNMA1 | Profile0 |
| FOXM1 | Profile0 |
| HIST1H4C | Profile0 |
| SKA3 | Profile0 |
| NRAS | Profile0 |
| CCNF | Profile0 |
| EHHADH | Profile0 |
| RTEL1-TNFRSF6B | Profile0 |
| RFC4 | Profile0 |
| UTP20 | Profile0 |
| CHAF1B | Profile0 |
| HIST2H3D | Profile0 |
| HIST1H2BL | Profile0 |
| SUV39H1 | Profile0 |
| GPR1 | Profile0 |
| SGO1 | Profile0 |
| LGALS3 | Profile0 |
| HIST1H1D | Profile0 |
| GINS2 | Profile0 |
| KIF11 | Profile0 |
| SAP30 | Profile0 |
| FANCI | Profile0 |
| HIST2H3A | Profile0 |
| HMGB3 | Profile0 |
| HIST1H2AM | Profile0 |
| HMGN2 | Profile0 |
| MAD2L1 | Profile0 |
| SASS6 | Profile0 |
| FBXO5 | Profile0 |
| LMNB1 | Profile0 |
| SHCBP1 | Profile0 |
| ZNF724 | Profile0 |
| TICRR | Profile0 |
| TCF19 | Profile0 |
| FAM111B | Profile0 |
| ENPP1 | Profile0 |
| ERCC6L | Profile0 |
| FABP5 | Profile0 |
| DTL | Profile0 |
| ADAMTS1 | Profile0 |
| TEDC1 | Profile0 |
| AHNAK | Profile0 |
| AP5Z1 | Profile0 |
| DLC1 | Profile0 |
| DDX25 | Profile0 |
| DSCC1 | Profile0 |
| AURKB | Profile0 |
| CLSPN | Profile0 |
| DDX39A | Profile0 |
| CIP2A | Profile0 |
| CDC25B | Profile0 |
| HNRNPH3 | Profile0 |
| CHTF18 | Profile0 |
| DEPDC1B | Profile0 |
| DIAPH3 | Profile0 |
| DNMT1 | Profile0 |
| E2F1 | Profile0 |
| CCNB1 | Profile0 |
| AC027796.3 | Profile0 |
| ARHGAP11A | Profile0 |
| ARHGEF26 | Profile0 |
| IFIH1 | Profile0 |
| BIRC5 | Profile0 |
| PIMREG | Profile0 |
| BNC1 | Profile0 |
| GINS4 | Profile0 |
| NFKBID | Profile0 |
| CDT1 | Profile0 |
| HIST2H2AB | Profile0 |
| RBL1 | Profile0 |
| TOMM34 | Profile0 |
| CENPH | Profile0 |
| IQCC | Profile0 |
| TSPAN5 | Profile0 |
| ESPL1 | Profile0 |
| ID1 | Profile0 |
| LRR1 | Profile0 |
| MIS18A | Profile0 |
| DSN1 | Profile0 |
| PBK | Profile0 |
| SSX2IP | Profile0 |
| MELK | Profile0 |
| HIST1H2AI | Profile0 |
| TUBA1B | Profile0 |
| HMGB1 | Profile0 |
| PCLAF | Profile0 |
| GPR137C | Profile0 |
| MAGI3 | Profile0 |
| SLC35F1 | Profile0 |
| MXD3 | Profile0 |
| KIF22 | Profile0 |
| GLYCTK | Profile0 |
| CRYBG1 | Profile0 |
| OIP5 | Profile0 |
| ATAD5 | Profile0 |
| ZRANB3 | Profile0 |
| MAP7D3 | Profile0 |
| OSGEPL1 | Profile0 |
| FEN1 | Profile0 |
| RASSF9 | Profile0 |
| TMPO | Profile0 |
| EGR1 | Profile0 |
| DDIAS | Profile0 |
| TMSB15B | Profile0 |
| CDC20 | Profile0 |
| RFWD3 | Profile0 |
| C18ORF54 | Profile0 |
| SFPQ | Profile0 |
| UFSP1 | Profile0 |
| AUNIP | Profile0 |
| KIFC1 | Profile0 |
| TAF9B | Profile0 |
| FANCB | Profile0 |
| RMI1 | Profile0 |
| VRK1 | Profile0 |
| HIST1H4B | Profile0 |
| HIST1H2BJ | Profile0 |
| POLA1 | Profile0 |
| H2AFX | Profile0 |
| HIST1H4H | Profile0 |
| HIST1H1B | Profile0 |
| LIG1 | Profile0 |
| ZNF85 | Profile0 |
| HIST1H2BN | Profile0 |
| PAICS | Profile0 |
| RASL11B | Profile0 |
| REXO5 | Profile0 |
| PKMYT1 | Profile0 |
| WDHD1 | Profile0 |
| CDC25C | Profile0 |
| SYCE2 | Profile0 |
| PLEKHG4 | Profile0 |
| CDH24 | Profile0 |
| FANCE | Profile0 |
| HIST1H1E | Profile0 |
| SPATA5 | Profile0 |
| POLD1 | Profile0 |
| EME1 | Profile0 |
| ASF1B | Profile0 |
| WDR62 | Profile0 |
| MCM2 | Profile0 |
| TUBB4B | Profile0 |
| HIST1H2BC | Profile0 |
| TRIP13 | Profile0 |
| TONSL | Profile0 |
| CAD | Profile0 |
| HIST1H1C | Profile0 |
| PCNA | Profile0 |
| HIST1H2AJ | Profile0 |
| CARD10 | Profile0 |
| KPNA2 | Profile0 |
| PSMC3IP | Profile0 |
| ID4 | Profile0 |
| HIST2H3C | Profile0 |
| TROAP | Profile0 |
| NUF2 | Profile0 |
| NCAPD3 | Profile0 |
| MCM7 | Profile0 |
| HIST2H2AC | Profile0 |
| PRMT3 | Profile0 |
| CDCA3 | Profile0 |
| UGDH | Profile0 |
| EXO1 | Profile0 |
| NOD1 | Profile0 |
| FANCG | Profile0 |
| HIST1H2AH | Profile0 |
| ZNF273 | Profile0 |
| PLK4 | Profile0 |
| TIMELESS | Profile0 |
| POLD3 | Profile0 |
| DDX10 | Profile0 |
| EZH2 | Profile0 |
| DCAF4L2 | Profile0 |
| DDX28 | Profile0 |
| ZNF726 | Profile0 |
| CHAF1A | Profile0 |
| C17ORF53 | Profile0 |
| GINS1 | Profile0 |
| LZTS1 | Profile0 |
| FOSL1 | Profile0 |
| HIST1H2BF | Profile0 |
| ZWINT | Profile0 |
| KIF18A | Profile0 |
| HIST1H2AG | Profile0 |
| HIST1H3H | Profile0 |
| TRAM1L1 | Profile0 |
| TRIM6 | Profile0 |
| TMEM100 | Profile0 |
| HIST1H2BD | Profile0 |
| LAPTM5 | Profile11 |
| ADAM12 | Profile11 |
| C1QTNF1 | Profile11 |
| ITGA1 | Profile11 |
| KCNN3 | Profile11 |
| SLC8A3 | Profile11 |
| RASD1 | Profile11 |
| C1S | Profile11 |
| MVP | Profile11 |
| GPR68 | Profile11 |
| ITGAV | Profile11 |
| MTSS1 | Profile11 |
| FAM174A | Profile11 |
| CSF3 | Profile11 |
| IL1A | Profile11 |
| CASP4 | Profile11 |
| ATP8B4 | Profile11 |
| ADORA2A | Profile11 |
| CFAP44 | Profile11 |
| KIAA0319 | Profile11 |
| SEZ6L2 | Profile11 |
| SHISA2 | Profile11 |
| SLC22A3 | Profile11 |
| TNFSF15 | Profile11 |
| PARD3B | Profile11 |
| CXCL5 | Profile11 |
| BCL2A1 | Profile11 |
| PTGIR | Profile11 |
| CCDC116 | Profile3 |
| SNRPA | Profile3 |
| HNRNPA3 | Profile3 |
| CDX1 | Profile3 |
| FAM72B | Profile3 |
| SCN9A | Profile3 |
| SKA1 | Profile3 |
| CDK1 | Profile3 |
| C9ORF40 | Profile3 |
| ZGRF1 | Profile3 |
| MKI67 | Profile3 |
| ADGRG6 | Profile3 |
| TRIM59 | Profile3 |
| BUB1B | Profile3 |
| CENPJ | Profile3 |
| WDR76 | Profile3 |
| CCNB2 | Profile3 |
| RPL39L | Profile3 |
| PLK1 | Profile3 |
| TMEM221 | Profile3 |
| POC1A | Profile3 |
| BCS1L | Profile3 |
| PKDCC | Profile3 |
| HIRIP3 | Profile3 |
| SGO2 | Profile3 |
| CCDC138 | Profile3 |
| INMT | Profile3 |
| HJURP | Profile3 |
| NSD2 | Profile3 |
| KNL1 | Profile3 |
| KIF18B | Profile3 |
| NKX2-3 | Profile3 |
| IQGAP3 | Profile3 |
| TYMS | Profile3 |
| PGM5 | Profile3 |
| DNA2 | Profile3 |
| NUDT15 | Profile3 |
| CENPA | Profile3 |
| DEPDC1 | Profile3 |
| ARHGAP11B | Profile3 |
| KIF14 | Profile3 |
| ANLN | Profile3 |
| RRM2 | Profile3 |
| CHAC2 | Profile3 |
| CEP128 | Profile3 |
| CDC45 | Profile3 |
| ESCO2 | Profile3 |
| FUS | Profile3 |
| PRKCA | Profile3 |
| SLC4A8 | Profile3 |
| AC091057.6 | Profile3 |
| KIF20B | Profile3 |
| CENPF | Profile3 |
| GTSE1 | Profile3 |
| POLQ | Profile3 |
| PTTG1 | Profile3 |
| UBE2S | Profile3 |
| INCENP | Profile3 |
| NDC80 | Profile3 |
| RNASEH2A | Profile3 |
| KIF23 | Profile3 |
| TNFRSF6B | Profile3 |
| CDCA2 | Profile3 |
| TACC3 | Profile3 |
| HAUS8 | Profile3 |
| CERS6 | Profile3 |
| C2ORF88 | Profile3 |
| DLGAP5 | Profile3 |
| ZNF385C | Profile3 |
| SPAG5 | Profile3 |
| NAPRT | Profile3 |
| BUB1 | Profile3 |
| ASPM | Profile3 |
| AURKA | Profile3 |
| PRPS2 | Profile3 |
| KIF2C | Profile3 |
| CKS2 | Profile3 |
| DBF4 | Profile3 |
| ITGB3BP | Profile3 |
| CTNNAL1 | Profile3 |
| TOP2A | Profile3 |
| RAD51AP1 | Profile3 |
| HNRNPH1 | Profile3 |
| NEIL3 | Profile3 |
| GFI1 | Profile3 |
| TRMU | Profile3 |
| KIF4A | Profile3 |
| CEP55 | Profile3 |
| PASK | Profile3 |
| STIL | Profile3 |
| MYLK2 | Profile3 |
| UBE2C | Profile3 |
| LRRCC1 | Profile3 |
| MCM8 | Profile3 |
| XRCC2 | Profile3 |
| SMAD9 | Profile3 |
| DNAJC22 | Profile3 |
| NR5A2 | Profile3 |
| HMGA1 | Profile3 |
| PIM3 | Profile3 |
| MTFR2 | Profile3 |
| NPHS1 | Profile3 |
| NCAPG | Profile3 |
| KIF15 | Profile3 |
| BARD1 | Profile3 |
| FGD4 | Profile3 |
| FMO1 | Profile3 |
| SDC1 | Profile3 |
| CDCA5 | Profile3 |
| THSD1 | Profile3 |
| PRC1 | Profile3 |
| KIF20A | Profile3 |
| SLF1 | Profile3 |
| RAD54B | Profile3 |
| CDKN3 | Profile3 |
| BLM | Profile3 |
| C4ORF3 | Profile12 |
| GPX3 | Profile12 |
| HAVCR2 | Profile12 |
| GABARAPL1 | Profile12 |
| FCRLA | Profile12 |
| PHF21A | Profile12 |
| COL4A1 | Profile12 |
| F11R | Profile12 |
| RCAN1 | Profile12 |
| CMKLR1 | Profile12 |
| AC018630.2 | Profile12 |
| SYNPO2 | Profile12 |
| COL15A1 | Profile12 |
| GPRIN3 | Profile12 |
| XIRP1 | Profile12 |
| SLMAP | Profile12 |
| ACOT2 | Profile12 |
| JAM2 | Profile12 |
| SYNGR2 | Profile12 |
| DGKB | Profile12 |
| SELENOM | Profile12 |
| ST5 | Profile12 |
| STAT4 | Profile12 |
| SLC9A7 | Profile12 |
| PBXIP1 | Profile12 |
| QPCTL | Profile12 |
| RGS3 | Profile12 |
| ANOS1 | Profile12 |
| BTN2A2 | Profile12 |
| SVEP1 | Profile12 |
| CAMTA2 | Profile12 |
| JMY | Profile12 |
| PCDHB13 | Profile12 |
| PRKAA2 | Profile12 |
| TMEM151A | Profile12 |
| SIDT2 | Profile12 |
| TMEM170B | Profile12 |
| ITM2C | Profile12 |
| GTDC1 | Profile12 |
| CSF1 | Profile12 |
| RSU1 | Profile12 |
| SLC46A1 | Profile12 |
| CHRD | Profile12 |
| GRN | Profile12 |
| HSD17B14 | Profile12 |
| HAPLN3 | Profile12 |
| SLC25A23 | Profile12 |
| FMOD | Profile12 |
| MYOZ2 | Profile12 |
| ZHX2 | Profile12 |
| SLC46A3 | Profile12 |
| C2CD4A | Profile12 |
| SNX18 | Profile12 |
| IDUA | Profile12 |
| PLXNB1 | Profile12 |
| IGIP | Profile12 |
| DACT1 | Profile12 |
| RAB3D | Profile12 |
| NPC1 | Profile12 |
| ARRDC4 | Profile12 |
| ORAI3 | Profile12 |
| TMEM86A | Profile12 |
| HSPB7 | Profile12 |
| BNIP3L | Profile12 |
| ST6GALNAC5 | Profile12 |
| CDH4 | Profile12 |
| DIPK1A | Profile12 |
| SPX | Profile12 |
| TCP11L2 | Profile15 |
| RIMBP3C | Profile15 |
| PLPPR2 | Profile15 |
| ZNF561 | Profile15 |
| ZC3H12A | Profile15 |
| TAGLN | Profile15 |
| TNIP1 | Profile15 |
| H2AFJ | Profile15 |
| PDZK1 | Profile15 |
| VEGFA | Profile15 |
| CCDC69 | Profile15 |
| AL121594.1 | Profile15 |
| CCL20 | Profile15 |
| BIRC3 | Profile15 |
| SARAF | Profile15 |
| ZNF425 | Profile15 |
| DSC3 | Profile15 |
| QPCT | Profile15 |
| RRAD | Profile15 |
| KCNK1 | Profile15 |
| EEF1AKMT4 | Profile15 |
| C19ORF81 | Profile15 |
| IGFBP7 | Profile15 |
| LYN | Profile15 |
| GALNT5 | Profile15 |
| BMP6 | Profile15 |
| SPRY1 | Profile15 |
| ATP6AP2 | Profile15 |
| SQOR | Profile15 |
| PIGZ | Profile15 |
| SHISA4 | Profile15 |
| FLT1 | Profile15 |
| C3 | Profile15 |
| GFOD1 | Profile15 |
| PRKAA1 | Profile15 |
| PDCD1LG2 | Profile15 |
| CHAC1 | Profile15 |
| NDUFA4L2 | Profile15 |
| ADGRB2 | Profile15 |
| ANKRD42 | Profile15 |
| FBXO32 | Profile15 |
| MSRB3 | Profile15 |
| GBP2 | Profile15 |
| CDCP1 | Profile15 |
| DNAJB9 | Profile15 |
| SCG5 | Profile15 |
| TCIM | Profile15 |
| TPCN1 | Profile15 |
| ENPEP | Profile15 |
| IGSF8 | Profile15 |
| VLDLR | Profile15 |
| TSTD3 | Profile15 |
| CELSR2 | Profile15 |
| RIPOR3 | Profile15 |
| IL13RA1 | Profile15 |
| PLOD1 | Profile15 |
| PRR15 | Profile15 |
| TXNIP | Profile15 |
| SAMD8 | Profile15 |
| FNDC5 | Profile15 |
| MXI1 | Profile15 |
| CCPG1 | Profile15 |
| RIN3 | Profile15 |
| DPP4 | Profile15 |
| RAB33A | Profile15 |
| FAM47E-STBD1 | Profile15 |
| HEPHL1 | Profile15 |
| EBI3 | Profile15 |
| NEXN | Profile15 |
| ZNF763 | Profile15 |
| INHBE | Profile15 |
| CYP19A1 | Profile15 |
| KLHL28 | Profile15 |
| SERPINB10 | Profile15 |
| COL4A2 | Profile15 |
| PDLIM3 | Profile15 |
| AHCYL2 | Profile15 |
| PHETA1 | Profile15 |
| SH3BGRL | Profile15 |
| RNF130 | Profile15 |
| GUCY1A2 | Profile15 |
| MEGF10 | Profile15 |
| PLAT | Profile15 |
| CALY | Profile15 |
| HNMT | Profile15 |
| NR4A2 | Profile15 |
| TUBE1 | Profile15 |
| AMPD3 | Profile15 |
| ULBP2 | Profile15 |
| IL4I1 | Profile15 |
| FILIP1L | Profile15 |
| ACOT1 | Profile15 |
| RNASE4 | Profile15 |
| PFKFB4 | Profile15 |
| SLC11A2 | Profile15 |
| TOM1 | Profile15 |
| SUSD6 | Profile15 |
| PPARA | Profile15 |
| LMOD1 | Profile15 |
| ITGB1BP2 | Profile15 |
| MID2 | Profile15 |
| LRP12 | Profile15 |
| MAN2B1 | Profile15 |
| ASS1 | Profile15 |
| PLOD2 | Profile15 |
| CLDN1 | Profile15 |
