# Supplementary material for: Multiomics global landscape of stemness-related gene clusters in adipose-derived mesenchymal stem cells
Source: Stem Cell Res Ther. 2020 Jul 22;11:310. doi: 10.1186/s13287-020-01823-3 (PMC7374825; doi:10.1186/s13287-020-01823-3)
Supplement: Supplementary file 2 — Additional file 2 : Table S2. The thirty-five genes belonging to the single stemness-related gene cluster associated with age. [file 13287_2020_1823_MOESM2_ESM.docx]

| Table S2. The thirty-five genes belonging to the single stemness-related gene cluster associated with age. | |
| --- | --- |
| gene | Profile |
| ANOS1 | Profile1 |
| ASS1 | Profile1 |
| ATP8B4 | Profile1 |
| BCL2A1 | Profile1 |
| BMP6 | Profile1 |
| C2CD4A | Profile1 |
| C3 | Profile1 |
| CALY | Profile1 |
| CCDC69 | Profile1 |
| CDH4 | Profile1 |
| CHAC1 | Profile1 |
| CLDN1 | Profile1 |
| COL15A1 | Profile1 |
| EBI3 | Profile1 |
| FMOD | Profile1 |
| GPRIN3 | Profile1 |
| GUCY1A2 | Profile1 |
| HEPHL1 | Profile1 |
| INHBE | Profile1 |
| ITGA1 | Profile1 |
| LAPTM5 | Profile1 |
| MEGF10 | Profile1 |
| NDUFA4L2 | Profile1 |
| PRKAA2 | Profile1 |
| RAB3D | Profile1 |
| RIMBP3C | Profile1 |
| RIPOR3 | Profile1 |
| RRAD | Profile1 |
| SCG5 | Profile1 |
| SHISA2 | Profile1 |
| SLC46A3 | Profile1 |
| SYNPO2 | Profile1 |
| TCIM | Profile1 |
| TMEM151A | Profile1 |
| TPBGL | Profile1 |
