# Supplementary material for: Multiomics global landscape of stemness-related gene clusters in adipose-derived mesenchymal stem cells
Source: Stem Cell Res Ther. 2020 Jul 22;11:310. doi: 10.1186/s13287-020-01823-3 (PMC7374825; doi:10.1186/s13287-020-01823-3)
Supplement: Supplementary file 3 — Additional file 3 : Table S3. GO_BPs enriched in adipose-derived mesenchymal cells. GO: gene ontology, BPs: biological processes. [file 13287_2020_1823_MOESM3_ESM.docx]

| Table S3. GO_BPs enriched in adipose-derived mesenchymal cells. | | | | | | | |
| --- | --- | --- | --- | --- | --- | --- | --- |
| Module | ID | Description | GeneRatio | BgRatio | pvalue | p.adjust | qvalue |
| GO_BP_Profile0 | GO:0071103 | DNA conformation change | 37/224 | 279/17913 | 2.64065E-27 | 7.13768E-24 | 6E-24 |
| GO_BP_Profile0 | GO:0006334 | nucleosome assembly | 27/224 | 135/17913 | 4.89154E-25 | 6.61092E-22 | 5.56E-22 |
| GO_BP_Profile0 | GO:0031497 | chromatin assembly | 27/224 | 153/17913 | 1.67925E-23 | 1.513E-20 | 1.27E-20 |
| GO_BP_Profile0 | GO:0006323 | DNA packaging | 29/224 | 194/17913 | 4.82086E-23 | 3.25769E-20 | 2.74E-20 |
| GO_BP_Profile0 | GO:0006260 | DNA replication | 32/224 | 260/17913 | 1.15227E-22 | 6.16535E-20 | 5.19E-20 |
| GO_BP_Profile0 | GO:0034728 | nucleosome organization | 27/224 | 165/17913 | 1.36856E-22 | 6.16535E-20 | 5.19E-20 |
| GO_BP_Profile0 | GO:0065004 | protein-DNA complex assembly | 29/224 | 210/17913 | 4.82641E-22 | 1.86368E-19 | 1.57E-19 |
| GO_BP_Profile0 | GO:0006333 | chromatin assembly or disassembly | 27/224 | 178/17913 | 1.09714E-21 | 3.70697E-19 | 3.12E-19 |
| GO_BP_Profile0 | GO:0071824 | protein-DNA complex subunit organization | 29/224 | 240/17913 | 2.19286E-20 | 6.58589E-18 | 5.54E-18 |
| GO_BP_Profile0 | GO:0006261 | DNA-dependent DNA replication | 23/224 | 130/17913 | 3.4411E-20 | 9.3013E-18 | 7.82E-18 |
| GO_BP_Profile0 | GO:0000819 | sister chromatid segregation | 20/224 | 157/17913 | 7.92375E-15 | 1.94708E-12 | 1.64E-12 |
| GO_BP_Profile0 | GO:0007059 | chromosome segregation | 25/224 | 275/17913 | 9.14767E-15 | 2.06051E-12 | 1.73E-12 |
| GO_BP_Profile0 | GO:0000070 | mitotic sister chromatid segregation | 18/224 | 132/17913 | 5.49119E-14 | 1.14175E-11 | 9.6E-12 |
| GO_BP_Profile0 | GO:0140014 | mitotic nuclear division | 22/224 | 237/17913 | 2.61875E-13 | 5.05605E-11 | 4.25E-11 |
| GO_BP_Profile0 | GO:0000280 | nuclear division | 26/224 | 357/17913 | 4.84022E-13 | 8.72207E-11 | 7.34E-11 |
| GO_BP_Profile0 | GO:0098813 | nuclear chromosome segregation | 21/224 | 220/17913 | 5.42281E-13 | 9.16116E-11 | 7.71E-11 |
| GO_BP_Profile0 | GO:0006271 | DNA strand elongation involved in DNA replication | 7/224 | 9/17913 | 1.5346E-12 | 2.44002E-10 | 2.05E-10 |
| GO_BP_Profile0 | GO:0048285 | organelle fission | 26/224 | 395/17913 | 4.87198E-12 | 7.31609E-10 | 6.15E-10 |
| GO_BP_Profile0 | GO:0051983 | regulation of chromosome segregation | 13/224 | 93/17913 | 1.38599E-10 | 1.92448E-08 | 1.62E-08 |
| GO_BP_Profile0 | GO:0000226 | microtubule cytoskeleton organization | 26/224 | 460/17913 | 1.42396E-10 | 1.92448E-08 | 1.62E-08 |
| GO_BP_Profile0 | GO:0022616 | DNA strand elongation | 7/224 | 16/17913 | 4.52616E-10 | 5.82582E-08 | 4.9E-08 |
| GO_BP_Profile0 | GO:0006342 | chromatin silencing | 12/224 | 83/17913 | 4.82733E-10 | 5.93103E-08 | 4.99E-08 |
| GO_BP_Profile0 | GO:0044839 | cell cycle G2/M phase transition | 19/224 | 257/17913 | 6.03741E-10 | 7.09527E-08 | 5.97E-08 |
| GO_BP_Profile0 | GO:1901990 | regulation of mitotic cell cycle phase transition | 24/224 | 428/17913 | 8.95165E-10 | 1.00818E-07 | 8.48E-08 |
| GO_BP_Profile0 | GO:0000183 | chromatin silencing at rDNA | 9/224 | 39/17913 | 9.74102E-10 | 1.0532E-07 | 8.86E-08 |
| GO_BP_Profile0 | GO:0000086 | G2/M transition of mitotic cell cycle | 18/224 | 238/17913 | 1.20846E-09 | 1.25634E-07 | 1.06E-07 |
| GO_BP_Profile0 | GO:0044786 | cell cycle DNA replication | 10/224 | 57/17913 | 1.97648E-09 | 1.97868E-07 | 1.66E-07 |
| GO_BP_Profile0 | GO:0032200 | telomere organization | 15/224 | 167/17913 | 2.92791E-09 | 2.82648E-07 | 2.38E-07 |
| GO_BP_Profile0 | GO:0006335 | DNA replication-dependent nucleosome assembly | 8/224 | 32/17913 | 4.28647E-09 | 3.85157E-07 | 3.24E-07 |
| GO_BP_Profile0 | GO:0034723 | DNA replication-dependent nucleosome organization | 8/224 | 32/17913 | 4.28647E-09 | 3.85157E-07 | 3.24E-07 |
| GO_BP_Profile0 | GO:1901987 | regulation of cell cycle phase transition | 24/224 | 464/17913 | 4.41726E-09 | 3.85157E-07 | 3.24E-07 |
| GO_BP_Profile0 | GO:0007051 | spindle organization | 14/224 | 147/17913 | 4.67532E-09 | 3.94918E-07 | 3.32E-07 |
| GO_BP_Profile0 | GO:0045814 | negative regulation of gene expression, epigenetic | 12/224 | 101/17913 | 4.87633E-09 | 3.99416E-07 | 3.36E-07 |
| GO_BP_Profile0 | GO:0033260 | nuclear DNA replication | 9/224 | 47/17913 | 5.74319E-09 | 4.56583E-07 | 3.84E-07 |
| GO_BP_Profile0 | GO:0000723 | telomere maintenance | 14/224 | 154/17913 | 8.5546E-09 | 6.60659E-07 | 5.56E-07 |
| GO_BP_Profile0 | GO:0033046 | negative regulation of sister chromatid segregation | 8/224 | 37/17913 | 1.49099E-08 | 1.11949E-06 | 9.42E-07 |
| GO_BP_Profile0 | GO:0033045 | regulation of sister chromatid segregation | 10/224 | 70/17913 | 1.57582E-08 | 1.1512E-06 | 9.68E-07 |
| GO_BP_Profile0 | GO:0006336 | DNA replication-independent nucleosome assembly | 9/224 | 53/17913 | 1.7502E-08 | 1.21303E-06 | 1.02E-06 |
| GO_BP_Profile0 | GO:0034724 | DNA replication-independent nucleosome organization | 9/224 | 53/17913 | 1.7502E-08 | 1.21303E-06 | 1.02E-06 |
| GO_BP_Profile0 | GO:0090068 | positive regulation of cell cycle process | 18/224 | 283/17913 | 1.85048E-08 | 1.23177E-06 | 1.04E-06 |
| GO_BP_Profile0 | GO:0051985 | negative regulation of chromosome segregation | 8/224 | 38/17913 | 1.86839E-08 | 1.23177E-06 | 1.04E-06 |
| GO_BP_Profile0 | GO:0000075 | cell cycle checkpoint | 15/224 | 197/17913 | 2.77487E-08 | 1.78582E-06 | 1.5E-06 |
| GO_BP_Profile0 | GO:0032201 | telomere maintenance via semi-conservative replication | 7/224 | 27/17913 | 3.1253E-08 | 1.96458E-06 | 1.65E-06 |
| GO_BP_Profile0 | GO:0051052 | regulation of DNA metabolic process | 21/224 | 403/17913 | 3.8048E-08 | 2.33736E-06 | 1.97E-06 |
| GO_BP_Profile0 | GO:0007093 | mitotic cell cycle checkpoint | 13/224 | 147/17913 | 4.10439E-08 | 2.46537E-06 | 2.07E-06 |
| GO_BP_Profile0 | GO:0034080 | CENP-A containing nucleosome assembly | 8/224 | 42/17913 | 4.31961E-08 | 2.48424E-06 | 2.09E-06 |
| GO_BP_Profile0 | GO:0061641 | CENP-A containing chromatin organization | 8/224 | 42/17913 | 4.31961E-08 | 2.48424E-06 | 2.09E-06 |
| GO_BP_Profile0 | GO:0045787 | positive regulation of cell cycle | 20/224 | 373/17913 | 5.10956E-08 | 2.87732E-06 | 2.42E-06 |
| GO_BP_Profile0 | GO:0006275 | regulation of DNA replication | 11/224 | 102/17913 | 6.00946E-08 | 3.31502E-06 | 2.79E-06 |
| GO_BP_Profile0 | GO:0030071 | regulation of mitotic metaphase/anaphase transition | 8/224 | 44/17913 | 6.34834E-08 | 3.43191E-06 | 2.89E-06 |
| GO_BP_Profile0 | GO:0006302 | double-strand break repair | 15/224 | 211/17913 | 6.91267E-08 | 3.60735E-06 | 3.03E-06 |
| GO_BP_Profile0 | GO:0006270 | DNA replication initiation | 7/224 | 30/17913 | 6.93978E-08 | 3.60735E-06 | 3.03E-06 |
| GO_BP_Profile0 | GO:0006310 | DNA recombination | 16/224 | 244/17913 | 7.70838E-08 | 3.93127E-06 | 3.31E-06 |
| GO_BP_Profile0 | GO:0031055 | chromatin remodeling at centromere | 8/224 | 46/17913 | 9.14769E-08 | 4.49567E-06 | 3.78E-06 |
| GO_BP_Profile0 | GO:1902099 | regulation of metaphase/anaphase transition of cell cycle | 8/224 | 46/17913 | 9.14769E-08 | 4.49567E-06 | 3.78E-06 |
| GO_BP_Profile0 | GO:0007091 | metaphase/anaphase transition of mitotic cell cycle | 8/224 | 47/17913 | 1.09063E-07 | 5.14598E-06 | 4.33E-06 |
| GO_BP_Profile0 | GO:0010965 | regulation of mitotic sister chromatid separation | 8/224 | 47/17913 | 1.09063E-07 | 5.14598E-06 | 4.33E-06 |
| GO_BP_Profile0 | GO:0045841 | negative regulation of mitotic metaphase/anaphase transition | 7/224 | 32/17913 | 1.12324E-07 | 5.14598E-06 | 4.33E-06 |
| GO_BP_Profile0 | GO:2000816 | negative regulation of mitotic sister chromatid separation | 7/224 | 32/17913 | 1.12324E-07 | 5.14598E-06 | 4.33E-06 |
| GO_BP_Profile0 | GO:0090329 | regulation of DNA-dependent DNA replication | 8/224 | 48/17913 | 1.29478E-07 | 5.83296E-06 | 4.91E-06 |
| GO_BP_Profile0 | GO:0071897 | DNA biosynthetic process | 14/224 | 192/17913 | 1.39682E-07 | 6.05208E-06 | 5.09E-06 |
| GO_BP_Profile0 | GO:1902100 | negative regulation of metaphase/anaphase transition of cell cycle | 7/224 | 33/17913 | 1.41058E-07 | 6.05208E-06 | 5.09E-06 |
| GO_BP_Profile0 | GO:1905819 | negative regulation of chromosome separation | 7/224 | 33/17913 | 1.41058E-07 | 6.05208E-06 | 5.09E-06 |
| GO_BP_Profile0 | GO:0044784 | metaphase/anaphase transition of cell cycle | 8/224 | 49/17913 | 1.53088E-07 | 6.36611E-06 | 5.35E-06 |
| GO_BP_Profile0 | GO:0051306 | mitotic sister chromatid separation | 8/224 | 49/17913 | 1.53088E-07 | 6.36611E-06 | 5.35E-06 |
| GO_BP_Profile0 | GO:0000731 | DNA synthesis involved in DNA repair | 8/224 | 50/17913 | 1.80301E-07 | 7.38413E-06 | 6.21E-06 |
| GO_BP_Profile0 | GO:0033048 | negative regulation of mitotic sister chromatid segregation | 7/224 | 35/17913 | 2.17368E-07 | 8.76934E-06 | 7.38E-06 |
| GO_BP_Profile0 | GO:1902850 | microtubule cytoskeleton organization involved in mitosis | 11/224 | 116/17913 | 2.27527E-07 | 9.04419E-06 | 7.61E-06 |
| GO_BP_Profile0 | GO:1905818 | regulation of chromosome separation | 8/224 | 52/17913 | 2.47356E-07 | 9.68989E-06 | 8.15E-06 |
| GO_BP_Profile0 | GO:0006297 | nucleotide-excision repair, DNA gap filling | 6/224 | 23/17913 | 3.02186E-07 | 1.16687E-05 | 9.82E-06 |
| GO_BP_Profile0 | GO:0034508 | centromere complex assembly | 8/224 | 54/17913 | 3.3473E-07 | 1.27433E-05 | 1.07E-05 |
| GO_BP_Profile0 | GO:1900262 | regulation of DNA-directed DNA polymerase activity | 4/224 | 6/17913 | 3.50175E-07 | 1.27982E-05 | 1.08E-05 |
| GO_BP_Profile0 | GO:1900264 | positive regulation of DNA-directed DNA polymerase activity | 4/224 | 6/17913 | 3.50175E-07 | 1.27982E-05 | 1.08E-05 |
| GO_BP_Profile0 | GO:0010948 | negative regulation of cell cycle process | 18/224 | 344/17913 | 3.50376E-07 | 1.27982E-05 | 1.08E-05 |
| GO_BP_Profile0 | GO:0043486 | histone exchange | 8/224 | 55/17913 | 3.87523E-07 | 1.39663E-05 | 1.17E-05 |
| GO_BP_Profile0 | GO:0042769 | DNA damage response, detection of DNA damage | 7/224 | 38/17913 | 3.95152E-07 | 1.39967E-05 | 1.18E-05 |
| GO_BP_Profile0 | GO:0051984 | positive regulation of chromosome segregation | 6/224 | 24/17913 | 3.98723E-07 | 1.39967E-05 | 1.18E-05 |
| GO_BP_Profile0 | GO:0007088 | regulation of mitotic nuclear division | 12/224 | 150/17913 | 4.12107E-07 | 1.42811E-05 | 1.2E-05 |
| GO_BP_Profile0 | GO:0033047 | regulation of mitotic sister chromatid segregation | 8/224 | 57/17913 | 5.14754E-07 | 1.76124E-05 | 1.48E-05 |
| GO_BP_Profile0 | GO:0019985 | translesion synthesis | 7/224 | 40/17913 | 5.71481E-07 | 1.93089E-05 | 1.62E-05 |
| GO_BP_Profile0 | GO:0044843 | cell cycle G1/S phase transition | 16/224 | 288/17913 | 7.2847E-07 | 2.43093E-05 | 2.04E-05 |
| GO_BP_Profile0 | GO:1901988 | negative regulation of cell cycle phase transition | 15/224 | 254/17913 | 7.59372E-07 | 2.50315E-05 | 2.11E-05 |
| GO_BP_Profile0 | GO:0051054 | positive regulation of DNA metabolic process | 14/224 | 222/17913 | 8.19653E-07 | 2.6693E-05 | 2.25E-05 |
| GO_BP_Profile0 | GO:0006298 | mismatch repair | 6/224 | 27/17913 | 8.49815E-07 | 2.73458E-05 | 2.3E-05 |
| GO_BP_Profile0 | GO:0032392 | DNA geometric change | 9/224 | 86/17913 | 1.27068E-06 | 4.04075E-05 | 3.4E-05 |
| GO_BP_Profile0 | GO:0045839 | negative regulation of mitotic nuclear division | 7/224 | 45/17913 | 1.31913E-06 | 4.14606E-05 | 3.49E-05 |
| GO_BP_Profile0 | GO:0008608 | attachment of spindle microtubules to kinetochore | 6/224 | 29/17913 | 1.33553E-06 | 4.14935E-05 | 3.49E-05 |
| GO_BP_Profile0 | GO:0000082 | G1/S transition of mitotic cell cycle | 15/224 | 269/17913 | 1.55921E-06 | 4.70055E-05 | 3.95E-05 |
| GO_BP_Profile0 | GO:0098532 | histone H3-K27 trimethylation | 4/224 | 8/17913 | 1.60222E-06 | 4.70055E-05 | 3.95E-05 |
| GO_BP_Profile0 | GO:1901991 | negative regulation of mitotic cell cycle phase transition | 14/224 | 235/17913 | 1.61362E-06 | 4.70055E-05 | 3.95E-05 |
| GO_BP_Profile0 | GO:1902749 | regulation of cell cycle G2/M phase transition | 13/224 | 202/17913 | 1.62673E-06 | 4.70055E-05 | 3.95E-05 |
| GO_BP_Profile0 | GO:0007094 | mitotic spindle assembly checkpoint | 6/224 | 30/17913 | 1.65206E-06 | 4.70055E-05 | 3.95E-05 |
| GO_BP_Profile0 | GO:0031577 | spindle checkpoint | 6/224 | 30/17913 | 1.65206E-06 | 4.70055E-05 | 3.95E-05 |
| GO_BP_Profile0 | GO:0071173 | spindle assembly checkpoint | 6/224 | 30/17913 | 1.65206E-06 | 4.70055E-05 | 3.95E-05 |
| GO_BP_Profile0 | GO:0071174 | mitotic spindle checkpoint | 6/224 | 30/17913 | 1.65206E-06 | 4.70055E-05 | 3.95E-05 |
| GO_BP_Profile0 | GO:0051321 | meiotic cell cycle | 13/224 | 203/17913 | 1.71905E-06 | 4.84021E-05 | 4.07E-05 |
| GO_BP_Profile0 | GO:0051783 | regulation of nuclear division | 12/224 | 172/17913 | 1.77525E-06 | 4.93656E-05 | 4.15E-05 |
| GO_BP_Profile0 | GO:0006301 | postreplication repair | 7/224 | 47/17913 | 1.7898E-06 | 4.93656E-05 | 4.15E-05 |
| GO_BP_Profile0 | GO:0051225 | spindle assembly | 9/224 | 90/17913 | 1.86895E-06 | 5.10279E-05 | 4.29E-05 |
| GO_BP_Profile0 | GO:0051304 | chromosome separation | 8/224 | 68/17913 | 2.04644E-06 | 5.53152E-05 | 4.65E-05 |
| GO_BP_Profile0 | GO:0007062 | sister chromatid cohesion | 7/224 | 50/17913 | 2.75356E-06 | 7.36917E-05 | 6.2E-05 |
| GO_BP_Profile0 | GO:0007052 | mitotic spindle organization | 9/224 | 95/17913 | 2.94585E-06 | 7.80651E-05 | 6.57E-05 |
| GO_BP_Profile0 | GO:0051784 | negative regulation of nuclear division | 7/224 | 52/17913 | 3.61073E-06 | 9.47553E-05 | 7.97E-05 |
| GO_BP_Profile0 | GO:0010389 | regulation of G2/M transition of mitotic cell cycle | 12/224 | 185/17913 | 3.79667E-06 | 9.86769E-05 | 8.3E-05 |
| GO_BP_Profile0 | GO:2001251 | negative regulation of chromosome organization | 10/224 | 125/17913 | 3.8728E-06 | 9.9697E-05 | 8.39E-05 |
| GO_BP_Profile0 | GO:0043044 | ATP-dependent chromatin remodeling | 8/224 | 77/17913 | 5.28981E-06 | 0.00013489 | 0.000113 |
| GO_BP_Profile0 | GO:0045652 | regulation of megakaryocyte differentiation | 8/224 | 79/17913 | 6.41952E-06 | 0.000162168 | 0.000136 |
| GO_BP_Profile0 | GO:0090305 | nucleic acid phosphodiester bond hydrolysis | 14/224 | 268/17913 | 7.42452E-06 | 0.000185819 | 0.000156 |
| GO_BP_Profile0 | GO:0045930 | negative regulation of mitotic cell cycle | 15/224 | 308/17913 | 8.14068E-06 | 0.000201874 | 0.00017 |
| GO_BP_Profile0 | GO:0010639 | negative regulation of organelle organization | 16/224 | 352/17913 | 9.65181E-06 | 0.000237171 | 0.0002 |
| GO_BP_Profile0 | GO:0060968 | regulation of gene silencing | 10/224 | 139/17913 | 9.99085E-06 | 0.000243291 | 0.000205 |
| GO_BP_Profile0 | GO:0007080 | mitotic metaphase plate congression | 6/224 | 41/17913 | 1.11536E-05 | 0.000269181 | 0.000226 |
| GO_BP_Profile0 | GO:0031570 | DNA integrity checkpoint | 10/224 | 141/17913 | 1.13321E-05 | 0.000271067 | 0.000228 |
| GO_BP_Profile0 | GO:0007098 | centrosome cycle | 9/224 | 112/17913 | 1.1466E-05 | 0.000271864 | 0.000229 |
| GO_BP_Profile0 | GO:0140013 | meiotic nuclear division | 10/224 | 142/17913 | 1.2059E-05 | 0.000283438 | 0.000238 |
| GO_BP_Profile0 | GO:0060964 | regulation of gene silencing by miRNA | 9/224 | 113/17913 | 1.23246E-05 | 0.000287184 | 0.000242 |
| GO_BP_Profile0 | GO:0060147 | regulation of posttranscriptional gene silencing | 9/224 | 117/17913 | 1.63298E-05 | 0.000374063 | 0.000315 |
| GO_BP_Profile0 | GO:0060966 | regulation of gene silencing by RNA | 9/224 | 117/17913 | 1.63298E-05 | 0.000374063 | 0.000315 |
| GO_BP_Profile0 | GO:1903046 | meiotic cell cycle process | 10/224 | 151/17913 | 2.06239E-05 | 0.000468458 | 0.000394 |
| GO_BP_Profile0 | GO:0031023 | microtubule organizing center organization | 9/224 | 122/17913 | 2.28501E-05 | 0.000514699 | 0.000433 |
| GO_BP_Profile0 | GO:0000083 | regulation of transcription involved in G1/S transition of mitotic cell cycle | 5/224 | 29/17913 | 2.71843E-05 | 0.000602689 | 0.000507 |
| GO_BP_Profile0 | GO:0030219 | megakaryocyte differentiation | 8/224 | 96/17913 | 2.72024E-05 | 0.000602689 | 0.000507 |
| GO_BP_Profile0 | GO:1901989 | positive regulation of cell cycle phase transition | 8/224 | 97/17913 | 2.93324E-05 | 0.000640548 | 0.000539 |
| GO_BP_Profile0 | GO:0072331 | signal transduction by p53 class mediator | 13/224 | 264/17913 | 2.93851E-05 | 0.000640548 | 0.000539 |
| GO_BP_Profile0 | GO:0036297 | interstrand cross-link repair | 6/224 | 50/17913 | 3.58909E-05 | 0.000776106 | 0.000653 |
| GO_BP_Profile0 | GO:0033044 | regulation of chromosome organization | 14/224 | 311/17913 | 3.90242E-05 | 0.000837162 | 0.000704 |
| GO_BP_Profile0 | GO:0090307 | mitotic spindle assembly | 6/224 | 52/17913 | 4.50351E-05 | 0.000958502 | 0.000806 |
| GO_BP_Profile0 | GO:0032508 | DNA duplex unwinding | 7/224 | 76/17913 | 4.57706E-05 | 0.000966546 | 0.000813 |
| GO_BP_Profile0 | GO:2001020 | regulation of response to DNA damage stimulus | 11/224 | 201/17913 | 4.69843E-05 | 0.000984486 | 0.000828 |
| GO_BP_Profile0 | GO:0016233 | telomere capping | 6/224 | 53/17913 | 5.02587E-05 | 0.001044995 | 0.000879 |
| GO_BP_Profile0 | GO:0070507 | regulation of microtubule cytoskeleton organization | 10/224 | 170/17913 | 5.68496E-05 | 0.001169285 | 0.000984 |
| GO_BP_Profile0 | GO:0009411 | response to UV | 9/224 | 137/17913 | 5.71016E-05 | 0.001169285 | 0.000984 |
| GO_BP_Profile0 | GO:0051310 | metaphase plate congression | 6/224 | 55/17913 | 6.21583E-05 | 0.001263262 | 0.001063 |
| GO_BP_Profile0 | GO:0045653 | negative regulation of megakaryocyte differentiation | 4/224 | 18/17913 | 6.34731E-05 | 0.00127198 | 0.00107 |
| GO_BP_Profile0 | GO:0000724 | double-strand break repair via homologous recombination | 8/224 | 108/17913 | 6.35284E-05 | 0.00127198 | 0.00107 |
| GO_BP_Profile0 | GO:0007144 | female meiosis I | 3/224 | 7/17913 | 6.50735E-05 | 0.001293335 | 0.001088 |
| GO_BP_Profile0 | GO:0000725 | recombinational repair | 8/224 | 109/17913 | 6.78325E-05 | 0.00133833 | 0.001126 |
| GO_BP_Profile0 | GO:0006303 | double-strand break repair via nonhomologous end joining | 7/224 | 81/17913 | 6.90561E-05 | 0.001352598 | 0.001138 |
| GO_BP_Profile0 | GO:0006296 | nucleotide-excision repair, DNA incision, 5'-to lesion | 5/224 | 37/17913 | 9.1984E-05 | 0.001788725 | 0.001505 |
| GO_BP_Profile0 | GO:0070734 | histone H3-K27 methylation | 4/224 | 20/17913 | 9.8544E-05 | 0.001902604 | 0.0016 |
| GO_BP_Profile0 | GO:0006268 | DNA unwinding involved in DNA replication | 3/224 | 8/17913 | 0.000103156 | 0.001977527 | 0.001663 |
| GO_BP_Profile0 | GO:0007127 | meiosis I | 7/224 | 88/17913 | 0.000117101 | 0.002229035 | 0.001875 |
| GO_BP_Profile0 | GO:0033683 | nucleotide-excision repair, DNA incision | 5/224 | 39/17913 | 0.000119054 | 0.002250376 | 0.001893 |
| GO_BP_Profile0 | GO:0002227 | innate immune response in mucosa | 4/224 | 21/17913 | 0.000120542 | 0.002262669 | 0.001903 |
| GO_BP_Profile0 | GO:0000726 | non-recombinational repair | 7/224 | 89/17913 | 0.000125764 | 0.002344416 | 0.001972 |
| GO_BP_Profile0 | GO:0044774 | mitotic DNA integrity checkpoint | 7/224 | 90/17913 | 0.000134941 | 0.002498251 | 0.002101 |
| GO_BP_Profile0 | GO:0061982 | meiosis I cell cycle process | 7/224 | 91/17913 | 0.000144653 | 0.002659845 | 0.002237 |
| GO_BP_Profile0 | GO:2000573 | positive regulation of DNA biosynthetic process | 6/224 | 66/17913 | 0.000173785 | 0.003173919 | 0.00267 |
| GO_BP_Profile0 | GO:0031109 | microtubule polymerization or depolymerization | 7/224 | 94/17913 | 0.00017724 | 0.003215301 | 0.002705 |
| GO_BP_Profile0 | GO:0032886 | regulation of microtubule-based process | 10/224 | 199/17913 | 0.000208865 | 0.003763744 | 0.003166 |
| GO_BP_Profile0 | GO:0001886 | endothelial cell morphogenesis | 3/224 | 10/17913 | 0.00021699 | 0.003884256 | 0.003267 |
| GO_BP_Profile0 | GO:0001556 | oocyte maturation | 4/224 | 25/17913 | 0.000244978 | 0.004356419 | 0.003664 |
| GO_BP_Profile0 | GO:0000077 | DNA damage checkpoint | 8/224 | 133/17913 | 0.000270875 | 0.00478545 | 0.004025 |
| GO_BP_Profile0 | GO:0050000 | chromosome localization | 6/224 | 72/17913 | 0.000280815 | 0.004886021 | 0.00411 |
| GO_BP_Profile0 | GO:0051303 | establishment of chromosome localization | 6/224 | 72/17913 | 0.000280815 | 0.004886021 | 0.00411 |
| GO_BP_Profile0 | GO:2001252 | positive regulation of chromosome organization | 9/224 | 169/17913 | 0.00028199 | 0.004886021 | 0.00411 |
| GO_BP_Profile0 | GO:0000076 | DNA replication checkpoint | 3/224 | 11/17913 | 0.000295611 | 0.005057187 | 0.004254 |
| GO_BP_Profile0 | GO:0035404 | histone-serine phosphorylation | 3/224 | 11/17913 | 0.000295611 | 0.005057187 | 0.004254 |
| GO_BP_Profile0 | GO:0006283 | transcription-coupled nucleotide-excision repair | 6/224 | 73/17913 | 0.000302809 | 0.005147759 | 0.00433 |
| GO_BP_Profile0 | GO:1902751 | positive regulation of cell cycle G2/M phase transition | 4/224 | 27/17913 | 0.000333277 | 0.005630299 | 0.004736 |
| GO_BP_Profile0 | GO:0046112 | nucleobase biosynthetic process | 3/224 | 12/17913 | 0.000390517 | 0.006515846 | 0.005481 |
| GO_BP_Profile0 | GO:0051988 | regulation of attachment of spindle microtubules to kinetochore | 3/224 | 12/17913 | 0.000390517 | 0.006515846 | 0.005481 |
| GO_BP_Profile0 | GO:0034968 | histone lysine methylation | 7/224 | 109/17913 | 0.000440621 | 0.007289719 | 0.006132 |
| GO_BP_Profile0 | GO:0031297 | replication fork processing | 4/224 | 29/17913 | 0.000442291 | 0.007289719 | 0.006132 |
| GO_BP_Profile0 | GO:0006273 | lagging strand elongation | 2/224 | 3/17913 | 0.000463189 | 0.007497005 | 0.006306 |
| GO_BP_Profile0 | GO:0070494 | regulation of thrombin-activated receptor signaling pathway | 2/224 | 3/17913 | 0.000463189 | 0.007497005 | 0.006306 |
| GO_BP_Profile0 | GO:0070495 | negative regulation of thrombin-activated receptor signaling pathway | 2/224 | 3/17913 | 0.000463189 | 0.007497005 | 0.006306 |
| GO_BP_Profile0 | GO:0038111 | interleukin-7-mediated signaling pathway | 4/224 | 30/17913 | 0.000505369 | 0.008131018 | 0.00684 |
| GO_BP_Profile0 | GO:0051290 | protein heterotetramerization | 5/224 | 53/17913 | 0.000514896 | 0.008235289 | 0.006927 |
| GO_BP_Profile0 | GO:1901992 | positive regulation of mitotic cell cycle phase transition | 6/224 | 82/17913 | 0.000567482 | 0.009022965 | 0.00759 |
| GO_BP_Profile0 | GO:1901976 | regulation of cell cycle checkpoint | 4/224 | 31/17913 | 0.000574592 | 0.009082584 | 0.00764 |
| GO_BP_Profile0 | GO:0061844 | antimicrobial humoral immune response mediated by antimicrobial peptide | 5/224 | 55/17913 | 0.000611683 | 0.009612676 | 0.008086 |
| GO_BP_Profile0 | GO:0090231 | regulation of spindle checkpoint | 3/224 | 14/17913 | 0.00063429 | 0.009797057 | 0.008241 |
| GO_BP_Profile0 | GO:0090266 | regulation of mitotic cell cycle spindle assembly checkpoint | 3/224 | 14/17913 | 0.00063429 | 0.009797057 | 0.008241 |
| GO_BP_Profile0 | GO:1903504 | regulation of mitotic spindle checkpoint | 3/224 | 14/17913 | 0.00063429 | 0.009797057 | 0.008241 |
| GO_BP_Profile0 | GO:0045931 | positive regulation of mitotic cell cycle | 8/224 | 154/17913 | 0.000721303 | 0.011004057 | 0.009256 |
| GO_BP_Profile0 | GO:0060249 | anatomical structure homeostasis | 14/224 | 413/17913 | 0.000728679 | 0.011004057 | 0.009256 |
| GO_BP_Profile0 | GO:0044773 | mitotic DNA damage checkpoint | 6/224 | 86/17913 | 0.000731092 | 0.011004057 | 0.009256 |
| GO_BP_Profile0 | GO:0002385 | mucosal immune response | 4/224 | 33/17913 | 0.00073279 | 0.011004057 | 0.009256 |
| GO_BP_Profile0 | GO:0045005 | DNA-dependent DNA replication maintenance of fidelity | 4/224 | 33/17913 | 0.00073279 | 0.011004057 | 0.009256 |
| GO_BP_Profile0 | GO:0006338 | chromatin remodeling | 8/224 | 156/17913 | 0.000784834 | 0.011667035 | 0.009814 |
| GO_BP_Profile0 | GO:0080182 | histone H3-K4 trimethylation | 3/224 | 15/17913 | 0.000785572 | 0.011667035 | 0.009814 |
| GO_BP_Profile0 | GO:0018022 | peptidyl-lysine methylation | 7/224 | 122/17913 | 0.000864168 | 0.012764183 | 0.010737 |
| GO_BP_Profile0 | GO:0033314 | mitotic DNA replication checkpoint | 2/224 | 4/17913 | 0.000918731 | 0.013496356 | 0.011353 |
| GO_BP_Profile0 | GO:0002251 | organ or tissue specific immune response | 4/224 | 36/17913 | 0.001024416 | 0.01496755 | 0.01259 |
| GO_BP_Profile0 | GO:0045739 | positive regulation of DNA repair | 5/224 | 62/17913 | 0.001060162 | 0.01540655 | 0.01296 |
| GO_BP_Profile0 | GO:0031571 | mitotic G1 DNA damage checkpoint | 5/224 | 63/17913 | 0.001139998 | 0.016390501 | 0.013787 |
| GO_BP_Profile0 | GO:0044819 | mitotic G1/S transition checkpoint | 5/224 | 63/17913 | 0.001139998 | 0.016390501 | 0.013787 |
| GO_BP_Profile0 | GO:0009314 | response to radiation | 14/224 | 435/17913 | 0.001198134 | 0.017135219 | 0.014414 |
| GO_BP_Profile0 | GO:0044783 | G1 DNA damage checkpoint | 5/224 | 64/17913 | 0.0012242 | 0.01741586 | 0.01465 |
| GO_BP_Profile0 | GO:0045637 | regulation of myeloid cell differentiation | 10/224 | 250/17913 | 0.001237373 | 0.017511095 | 0.01473 |
| GO_BP_Profile0 | GO:0016571 | histone methylation | 7/224 | 130/17913 | 0.001253258 | 0.017540507 | 0.014755 |
| GO_BP_Profile0 | GO:0006284 | base-excision repair | 4/224 | 38/17913 | 0.001258919 | 0.017540507 | 0.014755 |
| GO_BP_Profile0 | GO:0016572 | histone phosphorylation | 4/224 | 38/17913 | 0.001258919 | 0.017540507 | 0.014755 |
| GO_BP_Profile0 | GO:0042770 | signal transduction in response to DNA damage | 7/224 | 131/17913 | 0.001310216 | 0.018054737 | 0.015187 |
| GO_BP_Profile0 | GO:0006479 | protein methylation | 8/224 | 169/17913 | 0.001315865 | 0.018054737 | 0.015187 |
| GO_BP_Profile0 | GO:0008213 | protein alkylation | 8/224 | 169/17913 | 0.001315865 | 0.018054737 | 0.015187 |
| GO_BP_Profile0 | GO:0042276 | error-prone translesion synthesis | 3/224 | 18/17913 | 0.001370388 | 0.018707875 | 0.015737 |
| GO_BP_Profile0 | GO:0030174 | regulation of DNA-dependent DNA replication initiation | 2/224 | 5/17913 | 0.001518591 | 0.020421647 | 0.017178 |
| GO_BP_Profile0 | GO:0043987 | histone H3-S10 phosphorylation | 2/224 | 5/17913 | 0.001518591 | 0.020421647 | 0.017178 |
| GO_BP_Profile0 | GO:1905098 | negative regulation of guanyl-nucleotide exchange factor activity | 2/224 | 5/17913 | 0.001518591 | 0.020421647 | 0.017178 |
| GO_BP_Profile0 | GO:0070987 | error-free translesion synthesis | 3/224 | 19/17913 | 0.001612409 | 0.021575942 | 0.018149 |
| GO_BP_Profile0 | GO:0098760 | response to interleukin-7 | 4/224 | 41/17913 | 0.001677414 | 0.022225742 | 0.018696 |
| GO_BP_Profile0 | GO:0098761 | cellular response to interleukin-7 | 4/224 | 41/17913 | 0.001677414 | 0.022225742 | 0.018696 |
| GO_BP_Profile0 | GO:0006289 | nucleotide-excision repair | 6/224 | 102/17913 | 0.001775419 | 0.023409551 | 0.019691 |
| GO_BP_Profile0 | GO:0018023 | peptidyl-lysine trimethylation | 4/224 | 42/17913 | 0.001836024 | 0.02409113 | 0.020265 |
| GO_BP_Profile0 | GO:1901796 | regulation of signal transduction by p53 class mediator | 8/224 | 179/17913 | 0.001892847 | 0.02471674 | 0.020791 |
| GO_BP_Profile0 | GO:0045446 | endothelial cell differentiation | 6/224 | 105/17913 | 0.002057413 | 0.02673648 | 0.02249 |
| GO_BP_Profile0 | GO:2000278 | regulation of DNA biosynthetic process | 6/224 | 106/17913 | 0.002158543 | 0.027693005 | 0.023295 |
| GO_BP_Profile0 | GO:0007063 | regulation of sister chromatid cohesion | 3/224 | 21/17913 | 0.002172731 | 0.027693005 | 0.023295 |
| GO_BP_Profile0 | GO:0035024 | negative regulation of Rho protein signal transduction | 3/224 | 21/17913 | 0.002172731 | 0.027693005 | 0.023295 |
| GO_BP_Profile0 | GO:0019731 | antibacterial humoral response | 4/224 | 44/17913 | 0.002183859 | 0.027693005 | 0.023295 |
| GO_BP_Profile0 | GO:0048599 | oocyte development | 4/224 | 44/17913 | 0.002183859 | 0.027693005 | 0.023295 |
| GO_BP_Profile0 | GO:0072401 | signal transduction involved in DNA integrity checkpoint | 5/224 | 73/17913 | 0.002202736 | 0.027693005 | 0.023295 |
| GO_BP_Profile0 | GO:0072422 | signal transduction involved in DNA damage checkpoint | 5/224 | 73/17913 | 0.002202736 | 0.027693005 | 0.023295 |
| GO_BP_Profile0 | GO:0006287 | base-excision repair, gap-filling | 2/224 | 6/17913 | 0.00225912 | 0.028011022 | 0.023562 |
| GO_BP_Profile0 | GO:0045842 | positive regulation of mitotic metaphase/anaphase transition | 2/224 | 6/17913 | 0.00225912 | 0.028011022 | 0.023562 |
| GO_BP_Profile0 | GO:1901970 | positive regulation of mitotic sister chromatid separation | 2/224 | 6/17913 | 0.00225912 | 0.028011022 | 0.023562 |
| GO_BP_Profile0 | GO:0072395 | signal transduction involved in cell cycle checkpoint | 5/224 | 74/17913 | 0.002338722 | 0.028865596 | 0.024281 |
| GO_BP_Profile0 | GO:1904666 | regulation of ubiquitin protein ligase activity | 3/224 | 22/17913 | 0.002492758 | 0.03059952 | 0.025739 |
| GO_BP_Profile0 | GO:0016570 | histone modification | 13/224 | 420/17913 | 0.002501848 | 0.03059952 | 0.025739 |
| GO_BP_Profile0 | GO:0007064 | mitotic sister chromatid cohesion | 3/224 | 23/17913 | 0.00284044 | 0.034498773 | 0.029019 |
| GO_BP_Profile0 | GO:0006282 | regulation of DNA repair | 6/224 | 112/17913 | 0.002846181 | 0.034498773 | 0.029019 |
| GO_BP_Profile0 | GO:1903707 | negative regulation of hemopoiesis | 7/224 | 152/17913 | 0.003047651 | 0.036775899 | 0.030935 |
| GO_BP_Profile0 | GO:2001021 | negative regulation of response to DNA damage stimulus | 5/224 | 79/17913 | 0.003111786 | 0.036863506 | 0.031008 |
| GO_BP_Profile0 | GO:0009113 | purine nucleobase biosynthetic process | 2/224 | 7/17913 | 0.003136739 | 0.036863506 | 0.031008 |
| GO_BP_Profile0 | GO:0009157 | deoxyribonucleoside monophosphate biosynthetic process | 2/224 | 7/17913 | 0.003136739 | 0.036863506 | 0.031008 |
| GO_BP_Profile0 | GO:0051256 | mitotic spindle midzone assembly | 2/224 | 7/17913 | 0.003136739 | 0.036863506 | 0.031008 |
| GO_BP_Profile0 | GO:1902101 | positive regulation of metaphase/anaphase transition of cell cycle | 2/224 | 7/17913 | 0.003136739 | 0.036863506 | 0.031008 |
| GO_BP_Profile0 | GO:1905097 | regulation of guanyl-nucleotide exchange factor activity | 2/224 | 7/17913 | 0.003136739 | 0.036863506 | 0.031008 |
| GO_BP_Profile0 | GO:0010971 | positive regulation of G2/M transition of mitotic cell cycle | 3/224 | 24/17913 | 0.00321653 | 0.037637584 | 0.03166 |
| GO_BP_Profile0 | GO:0016569 | covalent chromatin modification | 13/224 | 433/17913 | 0.003246424 | 0.037666785 | 0.031684 |
| GO_BP_Profile0 | GO:0009994 | oocyte differentiation | 4/224 | 49/17913 | 0.003246896 | 0.037666785 | 0.031684 |
| GO_BP_Profile0 | GO:0031145 | anaphase-promoting complex-dependent catabolic process | 5/224 | 81/17913 | 0.003467321 | 0.040052004 | 0.033691 |
| GO_BP_Profile0 | GO:0051497 | negative regulation of stress fiber assembly | 3/224 | 25/17913 | 0.003621742 | 0.041657741 | 0.035041 |
| GO_BP_Profile0 | GO:0061640 | cytoskeleton-dependent cytokinesis | 5/224 | 82/17913 | 0.003655622 | 0.041869267 | 0.035219 |
| GO_BP_Profile0 | GO:0000079 | regulation of cyclin-dependent protein serine/threonine kinase activity | 5/224 | 83/17913 | 0.003851149 | 0.043922595 | 0.036946 |
| GO_BP_Profile0 | GO:0051568 | histone H3-K4 methylation | 4/224 | 52/17913 | 0.004029761 | 0.045766573 | 0.038497 |
| GO_BP_Profile0 | GO:0046602 | regulation of mitotic centrosome separation | 2/224 | 8/17913 | 0.004147934 | 0.046716103 | 0.039296 |
| GO_BP_Profile0 | GO:1905820 | positive regulation of chromosome separation | 2/224 | 8/17913 | 0.004147934 | 0.046716103 | 0.039296 |
| GO_BP_Profile0 | GO:0050830 | defense response to Gram-positive bacterium | 5/224 | 86/17913 | 0.004482594 | 0.050275733 | 0.04229 |
| GO_BP_Profile0 | GO:0009112 | nucleobase metabolic process | 3/224 | 27/17913 | 0.004522182 | 0.050510157 | 0.042488 |
| GO_BP_Profile0 | GO:0046605 | regulation of centrosome cycle | 4/224 | 54/17913 | 0.004617086 | 0.051357955 | 0.043201 |
| GO_BP_Profile0 | GO:0003158 | endothelium development | 6/224 | 124/17913 | 0.004697324 | 0.051736453 | 0.043519 |
| GO_BP_Profile0 | GO:2000134 | negative regulation of G1/S transition of mitotic cell cycle | 6/224 | 124/17913 | 0.004697324 | 0.051736453 | 0.043519 |
| GO_BP_Profile0 | GO:1904029 | regulation of cyclin-dependent protein kinase activity | 5/224 | 87/17913 | 0.004708534 | 0.051736453 | 0.043519 |
| GO_BP_Profile0 | GO:0001885 | endothelial cell development | 4/224 | 55/17913 | 0.004931235 | 0.053836715 | 0.045286 |
| GO_BP_Profile0 | GO:0051262 | protein tetramerization | 7/224 | 166/17913 | 0.004939514 | 0.053836715 | 0.045286 |
| GO_BP_Profile0 | GO:0032232 | negative regulation of actin filament bundle assembly | 3/224 | 28/17913 | 0.00501864 | 0.054261531 | 0.045643 |
| GO_BP_Profile0 | GO:0090344 | negative regulation of cell aging | 3/224 | 28/17913 | 0.00501864 | 0.054261531 | 0.045643 |
| GO_BP_Profile0 | GO:0006977 | DNA damage response, signal transduction by p53 class mediator resulting in cell cycle arrest | 4/224 | 56/17913 | 0.005259415 | 0.05606611 | 0.047161 |
| GO_BP_Profile0 | GO:0000022 | mitotic spindle elongation | 2/224 | 9/17913 | 0.005289256 | 0.05606611 | 0.047161 |
| GO_BP_Profile0 | GO:0045876 | positive regulation of sister chromatid cohesion | 2/224 | 9/17913 | 0.005289256 | 0.05606611 | 0.047161 |
| GO_BP_Profile0 | GO:0051103 | DNA ligation involved in DNA repair | 2/224 | 9/17913 | 0.005289256 | 0.05606611 | 0.047161 |
| GO_BP_Profile0 | GO:1904667 | negative regulation of ubiquitin protein ligase activity | 2/224 | 9/17913 | 0.005289256 | 0.05606611 | 0.047161 |
| GO_BP_Profile0 | GO:0051494 | negative regulation of cytoskeleton organization | 6/224 | 128/17913 | 0.005476344 | 0.057822487 | 0.048639 |
| GO_BP_Profile0 | GO:0072431 | signal transduction involved in mitotic G1 DNA damage checkpoint | 4/224 | 57/17913 | 0.005601899 | 0.058689661 | 0.049368 |
| GO_BP_Profile0 | GO:1902400 | intracellular signal transduction involved in G1 DNA damage checkpoint | 4/224 | 57/17913 | 0.005601899 | 0.058689661 | 0.049368 |
| GO_BP_Profile0 | GO:0007264 | small GTPase mediated signal transduction | 13/224 | 465/17913 | 0.005867299 | 0.061232851 | 0.051507 |
| GO_BP_Profile0 | GO:1902807 | negative regulation of cell cycle G1/S phase transition | 6/224 | 130/17913 | 0.005899471 | 0.061331811 | 0.051591 |
| GO_BP_Profile0 | GO:0045638 | negative regulation of myeloid cell differentiation | 5/224 | 92/17913 | 0.005960891 | 0.061497281 | 0.05173 |
| GO_BP_Profile0 | GO:2001022 | positive regulation of response to DNA damage stimulus | 5/224 | 92/17913 | 0.005960891 | 0.061497281 | 0.05173 |
| GO_BP_Profile0 | GO:0007143 | female meiotic nuclear division | 3/224 | 30/17913 | 0.006106825 | 0.062763305 | 0.052795 |
| GO_BP_Profile0 | GO:0051298 | centrosome duplication | 4/224 | 59/17913 | 0.006330849 | 0.064004352 | 0.053839 |
| GO_BP_Profile0 | GO:0072413 | signal transduction involved in mitotic cell cycle checkpoint | 4/224 | 59/17913 | 0.006330849 | 0.064004352 | 0.053839 |
| GO_BP_Profile0 | GO:1902402 | signal transduction involved in mitotic DNA damage checkpoint | 4/224 | 59/17913 | 0.006330849 | 0.064004352 | 0.053839 |
| GO_BP_Profile0 | GO:1902403 | signal transduction involved in mitotic DNA integrity checkpoint | 4/224 | 59/17913 | 0.006330849 | 0.064004352 | 0.053839 |
| GO_BP_Profile0 | GO:0007292 | female gamete generation | 6/224 | 132/17913 | 0.006345973 | 0.064004352 | 0.053839 |
| GO_BP_Profile0 | GO:0009162 | deoxyribonucleoside monophosphate metabolic process | 2/224 | 10/17913 | 0.006557322 | 0.064924696 | 0.054613 |
| GO_BP_Profile0 | GO:0032070 | regulation of deoxyribonuclease activity | 2/224 | 10/17913 | 0.006557322 | 0.064924696 | 0.054613 |
| GO_BP_Profile0 | GO:0046710 | GDP metabolic process | 2/224 | 10/17913 | 0.006557322 | 0.064924696 | 0.054613 |
| GO_BP_Profile0 | GO:0051231 | spindle elongation | 2/224 | 10/17913 | 0.006557322 | 0.064924696 | 0.054613 |
| GO_BP_Profile0 | GO:0051255 | spindle midzone assembly | 2/224 | 10/17913 | 0.006557322 | 0.064924696 | 0.054613 |
| GO_BP_Profile0 | GO:0051056 | regulation of small GTPase mediated signal transduction | 10/224 | 316/17913 | 0.006595467 | 0.065064043 | 0.05473 |
| GO_BP_Profile0 | GO:0003382 | epithelial cell morphogenesis | 3/224 | 31/17913 | 0.006699565 | 0.065375175 | 0.054992 |
| GO_BP_Profile0 | GO:0007099 | centriole replication | 3/224 | 31/17913 | 0.006699565 | 0.065375175 | 0.054992 |
| GO_BP_Profile0 | GO:1902230 | negative regulation of intrinsic apoptotic signaling pathway in response to DNA damage | 3/224 | 31/17913 | 0.006699565 | 0.065375175 | 0.054992 |
| GO_BP_Profile0 | GO:2000045 | regulation of G1/S transition of mitotic cell cycle | 7/224 | 177/17913 | 0.006959839 | 0.067670663 | 0.056923 |
| GO_BP_Profile0 | GO:0007265 | Ras protein signal transduction | 10/224 | 324/17913 | 0.007803024 | 0.075597034 | 0.06359 |
| GO_BP_Profile0 | GO:0007100 | mitotic centrosome separation | 2/224 | 11/17913 | 0.007948814 | 0.076190225 | 0.064089 |
| GO_BP_Profile0 | GO:0009143 | nucleoside triphosphate catabolic process | 2/224 | 11/17913 | 0.007948814 | 0.076190225 | 0.064089 |
| GO_BP_Profile0 | GO:1904424 | regulation of GTP binding | 2/224 | 11/17913 | 0.007948814 | 0.076190225 | 0.064089 |
| GO_BP_Profile0 | GO:0043414 | macromolecule methylation | 9/224 | 276/17913 | 0.008102087 | 0.077384956 | 0.065094 |
| GO_BP_Profile0 | GO:0007019 | microtubule depolymerization | 3/224 | 34/17913 | 0.008677723 | 0.082591143 | 0.069473 |
| GO_BP_Profile0 | GO:0006352 | DNA-templated transcription, initiation | 8/224 | 231/17913 | 0.008728663 | 0.082784475 | 0.069636 |
| GO_BP_Profile0 | GO:0046785 | microtubule polymerization | 4/224 | 66/17913 | 0.009370553 | 0.086978431 | 0.073164 |
| GO_BP_Profile0 | GO:0045740 | positive regulation of DNA replication | 3/224 | 35/17913 | 0.009405054 | 0.086978431 | 0.073164 |
| GO_BP_Profile0 | GO:0098534 | centriole assembly | 3/224 | 35/17913 | 0.009405054 | 0.086978431 | 0.073164 |
| GO_BP_Profile0 | GO:0000212 | meiotic spindle organization | 2/224 | 12/17913 | 0.009460473 | 0.086978431 | 0.073164 |
| GO_BP_Profile0 | GO:0007077 | mitotic nuclear envelope disassembly | 2/224 | 12/17913 | 0.009460473 | 0.086978431 | 0.073164 |
| GO_BP_Profile0 | GO:0010457 | centriole-centriole cohesion | 2/224 | 12/17913 | 0.009460473 | 0.086978431 | 0.073164 |
| GO_BP_Profile0 | GO:0051299 | centrosome separation | 2/224 | 12/17913 | 0.009460473 | 0.086978431 | 0.073164 |
| GO_BP_Profile0 | GO:0060707 | trophoblast giant cell differentiation | 2/224 | 12/17913 | 0.009460473 | 0.086978431 | 0.073164 |
| GO_BP_Profile0 | GO:0070493 | thrombin-activated receptor signaling pathway | 2/224 | 12/17913 | 0.009460473 | 0.086978431 | 0.073164 |
| GO_BP_Profile0 | GO:0045132 | meiotic chromosome segregation | 4/224 | 67/17913 | 0.009869988 | 0.090435853 | 0.076072 |
| GO_BP_Profile0 | GO:0048511 | rhythmic process | 9/224 | 287/17913 | 0.010303424 | 0.094072141 | 0.079131 |
| GO_BP_Profile0 | GO:0031503 | protein-containing complex localization | 8/224 | 238/17913 | 0.010336451 | 0.094072141 | 0.079131 |
| GO_BP_Profile0 | GO:0000910 | cytokinesis | 6/224 | 147/17913 | 0.010511779 | 0.095346772 | 0.080203 |
| GO_BP_Profile0 | GO:0019730 | antimicrobial humoral response | 5/224 | 106/17913 | 0.010682871 | 0.096252665 | 0.080965 |
| GO_BP_Profile0 | GO:0030330 | DNA damage response, signal transduction by p53 class mediator | 5/224 | 106/17913 | 0.010682871 | 0.096252665 | 0.080965 |
| GO_BP_Profile0 | GO:0000281 | mitotic cytokinesis | 4/224 | 69/17913 | 0.010919961 | 0.097317696 | 0.081861 |
| GO_BP_Profile0 | GO:0031110 | regulation of microtubule polymerization or depolymerization | 4/224 | 69/17913 | 0.010919961 | 0.097317696 | 0.081861 |
| GO_BP_Profile0 | GO:0032467 | positive regulation of cytokinesis | 3/224 | 37/17913 | 0.01096365 | 0.097317696 | 0.081861 |
| GO_BP_Profile0 | GO:0051567 | histone H3-K9 methylation | 3/224 | 37/17913 | 0.01096365 | 0.097317696 | 0.081861 |
| GO_BP_Profile0 | GO:0001833 | inner cell mass cell proliferation | 2/224 | 13/17913 | 0.011089105 | 0.097317696 | 0.081861 |
| GO_BP_Profile0 | GO:0009130 | pyrimidine nucleoside monophosphate biosynthetic process | 2/224 | 13/17913 | 0.011089105 | 0.097317696 | 0.081861 |
| GO_BP_Profile0 | GO:0009650 | UV protection | 2/224 | 13/17913 | 0.011089105 | 0.097317696 | 0.081861 |
| GO_BP_Profile0 | GO:0062033 | positive regulation of mitotic sister chromatid segregation | 2/224 | 13/17913 | 0.011089105 | 0.097317696 | 0.081861 |
| GO_BP_Profile0 | GO:1902806 | regulation of cell cycle G1/S phase transition | 7/224 | 195/17913 | 0.011505273 | 0.100438821 | 0.084486 |
| GO_BP_Profile0 | GO:0071156 | regulation of cell cycle arrest | 5/224 | 108/17913 | 0.011519066 | 0.100438821 | 0.084486 |
| GO_BP_Profile0 | GO:0003345 | proepicardium cell migration involved in pericardium morphogenesis | 1/224 | 1/17913 | 0.012504885 | 0.102426374 | 0.086158 |
| GO_BP_Profile0 | GO:0006272 | leading strand elongation | 1/224 | 1/17913 | 0.012504885 | 0.102426374 | 0.086158 |
| GO_BP_Profile0 | GO:0009956 | radial pattern formation | 1/224 | 1/17913 | 0.012504885 | 0.102426374 | 0.086158 |
| GO_BP_Profile0 | GO:0032072 | regulation of restriction endodeoxyribonuclease activity | 1/224 | 1/17913 | 0.012504885 | 0.102426374 | 0.086158 |
| GO_BP_Profile0 | GO:0036164 | cell-abiotic substrate adhesion | 1/224 | 1/17913 | 0.012504885 | 0.102426374 | 0.086158 |
| GO_BP_Profile0 | GO:0036292 | DNA rewinding | 1/224 | 1/17913 | 0.012504885 | 0.102426374 | 0.086158 |
| GO_BP_Profile0 | GO:0036333 | hepatocyte homeostasis | 1/224 | 1/17913 | 0.012504885 | 0.102426374 | 0.086158 |
| GO_BP_Profile0 | GO:0046534 | positive regulation of photoreceptor cell differentiation | 1/224 | 1/17913 | 0.012504885 | 0.102426374 | 0.086158 |
| GO_BP_Profile0 | GO:0046680 | response to DDT | 1/224 | 1/17913 | 0.012504885 | 0.102426374 | 0.086158 |
| GO_BP_Profile0 | GO:0061682 | seminal vesicle morphogenesis | 1/224 | 1/17913 | 0.012504885 | 0.102426374 | 0.086158 |
| GO_BP_Profile0 | GO:0072303 | positive regulation of glomerular metanephric mesangial cell proliferation | 1/224 | 1/17913 | 0.012504885 | 0.102426374 | 0.086158 |
| GO_BP_Profile0 | GO:0090233 | negative regulation of spindle checkpoint | 1/224 | 1/17913 | 0.012504885 | 0.102426374 | 0.086158 |
| GO_BP_Profile0 | GO:1902426 | deactivation of mitotic spindle assembly checkpoint | 1/224 | 1/17913 | 0.012504885 | 0.102426374 | 0.086158 |
| GO_BP_Profile0 | GO:1903126 | negative regulation of centriole-centriole cohesion | 1/224 | 1/17913 | 0.012504885 | 0.102426374 | 0.086158 |
| GO_BP_Profile0 | GO:1904481 | response to tetrahydrofolate | 1/224 | 1/17913 | 0.012504885 | 0.102426374 | 0.086158 |
| GO_BP_Profile0 | GO:1904482 | cellular response to tetrahydrofolate | 1/224 | 1/17913 | 0.012504885 | 0.102426374 | 0.086158 |
| GO_BP_Profile0 | GO:1904772 | response to tetrachloromethane | 1/224 | 1/17913 | 0.012504885 | 0.102426374 | 0.086158 |
| GO_BP_Profile0 | GO:1905341 | negative regulation of protein localization to kinetochore | 1/224 | 1/17913 | 0.012504885 | 0.102426374 | 0.086158 |
| GO_BP_Profile0 | GO:1990787 | negative regulation of hh target transcription factor activity | 1/224 | 1/17913 | 0.012504885 | 0.102426374 | 0.086158 |
| GO_BP_Profile0 | GO:2000775 | histone H3-S10 phosphorylation involved in chromosome condensation | 1/224 | 1/17913 | 0.012504885 | 0.102426374 | 0.086158 |
| GO_BP_Profile0 | GO:1902229 | regulation of intrinsic apoptotic signaling pathway in response to DNA damage | 3/224 | 39/17913 | 0.012662676 | 0.103405474 | 0.086982 |
| GO_BP_Profile0 | GO:0009129 | pyrimidine nucleoside monophosphate metabolic process | 2/224 | 14/17913 | 0.012831574 | 0.103843547 | 0.08735 |
| GO_BP_Profile0 | GO:0046037 | GMP metabolic process | 2/224 | 14/17913 | 0.012831574 | 0.103843547 | 0.08735 |
| GO_BP_Profile0 | GO:0046606 | negative regulation of centrosome cycle | 2/224 | 14/17913 | 0.012831574 | 0.103843547 | 0.08735 |
| GO_BP_Profile0 | GO:0030705 | cytoskeleton-dependent intracellular transport | 6/224 | 154/17913 | 0.013002773 | 0.104914909 | 0.088251 |
| GO_BP_Profile0 | GO:0031100 | animal organ regeneration | 4/224 | 75/17913 | 0.014492955 | 0.11659065 | 0.098073 |
| GO_BP_Profile0 | GO:0006144 | purine nucleobase metabolic process | 2/224 | 15/17913 | 0.014684805 | 0.11708858 | 0.098491 |
| GO_BP_Profile0 | GO:0042754 | negative regulation of circadian rhythm | 2/224 | 15/17913 | 0.014684805 | 0.11708858 | 0.098491 |
| GO_BP_Profile0 | GO:0048012 | hepatocyte growth factor receptor signaling pathway | 2/224 | 15/17913 | 0.014684805 | 0.11708858 | 0.098491 |
| GO_BP_Profile0 | GO:1903706 | regulation of hemopoiesis | 12/224 | 468/17913 | 0.015035911 | 0.119535489 | 0.10055 |
| GO_BP_Profile0 | GO:0006890 | retrograde vesicle-mediated transport, Golgi to ER | 4/224 | 76/17913 | 0.015152072 | 0.120105717 | 0.101029 |
| GO_BP_Profile0 | GO:0009416 | response to light stimulus | 9/224 | 307/17913 | 0.015421759 | 0.121886008 | 0.102527 |
| GO_BP_Profile0 | GO:0034644 | cellular response to UV | 4/224 | 77/17913 | 0.015829811 | 0.124746297 | 0.104933 |
| GO_BP_Profile0 | GO:0051493 | regulation of cytoskeleton organization | 12/224 | 472/17913 | 0.015970653 | 0.125490337 | 0.105559 |
| GO_BP_Profile0 | GO:0051100 | negative regulation of binding | 6/224 | 162/17913 | 0.016325508 | 0.127822576 | 0.107521 |
| GO_BP_Profile0 | GO:0071364 | cellular response to epidermal growth factor stimulus | 3/224 | 43/17913 | 0.016488953 | 0.127822576 | 0.107521 |
| GO_BP_Profile0 | GO:0030397 | membrane disassembly | 2/224 | 16/17913 | 0.016645781 | 0.127822576 | 0.107521 |
| GO_BP_Profile0 | GO:0034501 | protein localization to kinetochore | 2/224 | 16/17913 | 0.016645781 | 0.127822576 | 0.107521 |
| GO_BP_Profile0 | GO:0044849 | estrous cycle | 2/224 | 16/17913 | 0.016645781 | 0.127822576 | 0.107521 |
| GO_BP_Profile0 | GO:0051081 | nuclear envelope disassembly | 2/224 | 16/17913 | 0.016645781 | 0.127822576 | 0.107521 |
| GO_BP_Profile0 | GO:0051444 | negative regulation of ubiquitin-protein transferase activity | 2/224 | 16/17913 | 0.016645781 | 0.127822576 | 0.107521 |
| GO_BP_Profile0 | GO:1902166 | negative regulation of intrinsic apoptotic signaling pathway in response to DNA damage by p53 class mediator | 2/224 | 16/17913 | 0.016645781 | 0.127822576 | 0.107521 |
| GO_BP_Profile0 | GO:0051101 | regulation of DNA binding | 5/224 | 120/17913 | 0.01748284 | 0.133870022 | 0.112608 |
| GO_BP_Profile0 | GO:0007140 | male meiotic nuclear division | 3/224 | 44/17913 | 0.017535667 | 0.13389522 | 0.112629 |
| GO_BP_Profile0 | GO:0051098 | regulation of binding | 10/224 | 368/17913 | 0.017746039 | 0.135119844 | 0.113659 |
| GO_BP_Profile0 | GO:0006998 | nuclear envelope organization | 3/224 | 45/17913 | 0.018618663 | 0.140626216 | 0.118291 |
| GO_BP_Profile0 | GO:0046580 | negative regulation of Ras protein signal transduction | 3/224 | 45/17913 | 0.018618663 | 0.140626216 | 0.118291 |
| GO_BP_Profile0 | GO:0006266 | DNA ligation | 2/224 | 17/17913 | 0.018711543 | 0.140626216 | 0.118291 |
| GO_BP_Profile0 | GO:1904355 | positive regulation of telomere capping | 2/224 | 17/17913 | 0.018711543 | 0.140626216 | 0.118291 |
| GO_BP_Profile0 | GO:0046578 | regulation of Ras protein signal transduction | 7/224 | 215/17913 | 0.018729352 | 0.140626216 | 0.118291 |
| GO_BP_Profile0 | GO:0034502 | protein localization to chromosome | 4/224 | 82/17913 | 0.019503037 | 0.145626265 | 0.122497 |
| GO_BP_Profile0 | GO:0071158 | positive regulation of cell cycle arrest | 4/224 | 82/17913 | 0.019503037 | 0.145626265 | 0.122497 |
| GO_BP_Profile0 | GO:0035023 | regulation of Rho protein signal transduction | 5/224 | 124/17913 | 0.01985259 | 0.147827966 | 0.124349 |
| GO_BP_Profile0 | GO:0018205 | peptidyl-lysine modification | 10/224 | 376/17913 | 0.020265807 | 0.150490317 | 0.126588 |
| GO_BP_Profile0 | GO:0007130 | synaptonemal complex assembly | 2/224 | 18/17913 | 0.020879187 | 0.153051087 | 0.128742 |
| GO_BP_Profile0 | GO:1902165 | regulation of intrinsic apoptotic signaling pathway in response to DNA damage by p53 class mediator | 2/224 | 18/17913 | 0.020879187 | 0.153051087 | 0.128742 |
| GO_BP_Profile0 | GO:0045143 | homologous chromosome segregation | 3/224 | 47/17913 | 0.020893767 | 0.153051087 | 0.128742 |
| GO_BP_Profile0 | GO:0051438 | regulation of ubiquitin-protein transferase activity | 3/224 | 47/17913 | 0.020893767 | 0.153051087 | 0.128742 |
| GO_BP_Profile0 | GO:0070849 | response to epidermal growth factor | 3/224 | 47/17913 | 0.020893767 | 0.153051087 | 0.128742 |
| GO_BP_Profile0 | GO:0000018 | regulation of DNA recombination | 4/224 | 84/17913 | 0.021107612 | 0.153784027 | 0.129359 |
| GO_BP_Profile0 | GO:0048477 | oogenesis | 4/224 | 84/17913 | 0.021107612 | 0.153784027 | 0.129359 |
| GO_BP_Profile0 | GO:0071478 | cellular response to radiation | 6/224 | 173/17913 | 0.021791635 | 0.15815121 | 0.133032 |
| GO_BP_Profile0 | GO:0022412 | cellular process involved in reproduction in multicellular organism | 9/224 | 326/17913 | 0.021824048 | 0.15815121 | 0.133032 |
| GO_BP_Profile0 | GO:0061647 | histone H3-K9 modification | 3/224 | 48/17913 | 0.022085956 | 0.159621227 | 0.134269 |
| GO_BP_Profile0 | GO:0032355 | response to estradiol | 5/224 | 128/17913 | 0.022423831 | 0.161201107 | 0.135598 |
| GO_BP_Profile0 | GO:0051291 | protein heterooligomerization | 5/224 | 128/17913 | 0.022423831 | 0.161201107 | 0.135598 |
| GO_BP_Profile0 | GO:0007018 | microtubule-based movement | 7/224 | 224/17913 | 0.022840983 | 0.162664882 | 0.136829 |
| GO_BP_Profile0 | GO:0001832 | blastocyst growth | 2/224 | 19/17913 | 0.023145867 | 0.162664882 | 0.136829 |
| GO_BP_Profile0 | GO:0032069 | regulation of nuclease activity | 2/224 | 19/17913 | 0.023145867 | 0.162664882 | 0.136829 |
| GO_BP_Profile0 | GO:0032732 | positive regulation of interleukin-1 production | 3/224 | 50/17913 | 0.02457966 | 0.162664882 | 0.136829 |
| GO_BP_Profile0 | GO:0000472 | endonucleolytic cleavage to generate mature 5'-end of SSU-rRNA from (SSU-rRNA, 5.8S rRNA, LSU-rRNA) | 1/224 | 2/17913 | 0.024854087 | 0.162664882 | 0.136829 |
| GO_BP_Profile0 | GO:0000967 | rRNA 5'-end processing | 1/224 | 2/17913 | 0.024854087 | 0.162664882 | 0.136829 |
| GO_BP_Profile0 | GO:0006231 | dTMP biosynthetic process | 1/224 | 2/17913 | 0.024854087 | 0.162664882 | 0.136829 |
| GO_BP_Profile0 | GO:0007057 | spindle assembly involved in female meiosis I | 1/224 | 2/17913 | 0.024854087 | 0.162664882 | 0.136829 |
| GO_BP_Profile0 | GO:0017055 | negative regulation of RNA polymerase II transcriptional preinitiation complex assembly | 1/224 | 2/17913 | 0.024854087 | 0.162664882 | 0.136829 |
| GO_BP_Profile0 | GO:0030505 | inorganic diphosphate transport | 1/224 | 2/17913 | 0.024854087 | 0.162664882 | 0.136829 |
| GO_BP_Profile0 | GO:0031049 | programmed DNA elimination | 1/224 | 2/17913 | 0.024854087 | 0.162664882 | 0.136829 |
| GO_BP_Profile0 | GO:0031052 | chromosome breakage | 1/224 | 2/17913 | 0.024854087 | 0.162664882 | 0.136829 |
| GO_BP_Profile0 | GO:0033567 | DNA replication, Okazaki fragment processing | 1/224 | 2/17913 | 0.024854087 | 0.162664882 | 0.136829 |
| GO_BP_Profile0 | GO:0036034 | mediator complex assembly | 1/224 | 2/17913 | 0.024854087 | 0.162664882 | 0.136829 |
| GO_BP_Profile0 | GO:0043137 | DNA replication, removal of RNA primer | 1/224 | 2/17913 | 0.024854087 | 0.162664882 | 0.136829 |
| GO_BP_Profile0 | GO:0046073 | dTMP metabolic process | 1/224 | 2/17913 | 0.024854087 | 0.162664882 | 0.136829 |
| GO_BP_Profile0 | GO:0060849 | regulation of transcription involved in lymphatic endothelial cell fate commitment | 1/224 | 2/17913 | 0.024854087 | 0.162664882 | 0.136829 |
| GO_BP_Profile0 | GO:0071163 | DNA replication preinitiation complex assembly | 1/224 | 2/17913 | 0.024854087 | 0.162664882 | 0.136829 |
| GO_BP_Profile0 | GO:0071283 | cellular response to iron(III) ion | 1/224 | 2/17913 | 0.024854087 | 0.162664882 | 0.136829 |
| GO_BP_Profile0 | GO:0071505 | response to mycophenolic acid | 1/224 | 2/17913 | 0.024854087 | 0.162664882 | 0.136829 |
| GO_BP_Profile0 | GO:0071506 | cellular response to mycophenolic acid | 1/224 | 2/17913 | 0.024854087 | 0.162664882 | 0.136829 |
| GO_BP_Profile0 | GO:0072301 | regulation of metanephric glomerular mesangial cell proliferation | 1/224 | 2/17913 | 0.024854087 | 0.162664882 | 0.136829 |
| GO_BP_Profile0 | GO:0072355 | histone H3-T3 phosphorylation | 1/224 | 2/17913 | 0.024854087 | 0.162664882 | 0.136829 |
| GO_BP_Profile0 | GO:0075506 | entry of viral genome into host nucleus through nuclear pore complex via importin | 1/224 | 2/17913 | 0.024854087 | 0.162664882 | 0.136829 |
| GO_BP_Profile0 | GO:0090618 | DNA clamp unloading | 1/224 | 2/17913 | 0.024854087 | 0.162664882 | 0.136829 |
| GO_BP_Profile0 | GO:0099178 | regulation of retrograde trans-synaptic signaling by endocanabinoid | 1/224 | 2/17913 | 0.024854087 | 0.162664882 | 0.136829 |
| GO_BP_Profile0 | GO:1902595 | regulation of DNA replication origin binding | 1/224 | 2/17913 | 0.024854087 | 0.162664882 | 0.136829 |
| GO_BP_Profile0 | GO:1904975 | response to bleomycin | 1/224 | 2/17913 | 0.024854087 | 0.162664882 | 0.136829 |
| GO_BP_Profile0 | GO:1904976 | cellular response to bleomycin | 1/224 | 2/17913 | 0.024854087 | 0.162664882 | 0.136829 |
| GO_BP_Profile0 | GO:1905719 | protein localization to perinuclear region of cytoplasm | 1/224 | 2/17913 | 0.024854087 | 0.162664882 | 0.136829 |
| GO_BP_Profile0 | GO:1905775 | negative regulation of DNA helicase activity | 1/224 | 2/17913 | 0.024854087 | 0.162664882 | 0.136829 |
| GO_BP_Profile0 | GO:1905916 | negative regulation of cell differentiation involved in phenotypic switching | 1/224 | 2/17913 | 0.024854087 | 0.162664882 | 0.136829 |
| GO_BP_Profile0 | GO:1905931 | negative regulation of vascular smooth muscle cell differentiation involved in phenotypic switching | 1/224 | 2/17913 | 0.024854087 | 0.162664882 | 0.136829 |
| GO_BP_Profile0 | GO:2000426 | negative regulation of apoptotic cell clearance | 1/224 | 2/17913 | 0.024854087 | 0.162664882 | 0.136829 |
| GO_BP_Profile0 | GO:2000521 | negative regulation of immunological synapse formation | 1/224 | 2/17913 | 0.024854087 | 0.162664882 | 0.136829 |
| GO_BP_Profile0 | GO:2001176 | regulation of mediator complex assembly | 1/224 | 2/17913 | 0.024854087 | 0.162664882 | 0.136829 |
| GO_BP_Profile0 | GO:2001178 | positive regulation of mediator complex assembly | 1/224 | 2/17913 | 0.024854087 | 0.162664882 | 0.136829 |
| GO_BP_Profile0 | GO:0051383 | kinetochore organization | 2/224 | 20/17913 | 0.02550879 | 0.166546521 | 0.140094 |
| GO_BP_Profile0 | GO:0032259 | methylation | 9/224 | 336/17913 | 0.025872029 | 0.16656361 | 0.140108 |
| GO_BP_Profile0 | GO:0045599 | negative regulation of fat cell differentiation | 3/224 | 51/17913 | 0.025881138 | 0.16656361 | 0.140108 |
| GO_BP_Profile0 | GO:0045620 | negative regulation of lymphocyte differentiation | 3/224 | 51/17913 | 0.025881138 | 0.16656361 | 0.140108 |
| GO_BP_Profile0 | GO:0048146 | positive regulation of fibroblast proliferation | 3/224 | 51/17913 | 0.025881138 | 0.16656361 | 0.140108 |
| GO_BP_Profile0 | GO:0051058 | negative regulation of small GTPase mediated signal transduction | 3/224 | 51/17913 | 0.025881138 | 0.16656361 | 0.140108 |
| GO_BP_Profile0 | GO:0070228 | regulation of lymphocyte apoptotic process | 3/224 | 51/17913 | 0.025881138 | 0.16656361 | 0.140108 |
| GO_BP_Profile0 | GO:0051099 | positive regulation of binding | 6/224 | 181/17913 | 0.026466256 | 0.169924679 | 0.142936 |
| GO_BP_Profile0 | GO:0072528 | pyrimidine-containing compound biosynthetic process | 3/224 | 52/17913 | 0.027218978 | 0.174343356 | 0.146653 |
| GO_BP_Profile0 | GO:0010829 | negative regulation of glucose transmembrane transport | 2/224 | 21/17913 | 0.027965217 | 0.177441273 | 0.149258 |
| GO_BP_Profile0 | GO:0070193 | synaptonemal complex organization | 2/224 | 21/17913 | 0.027965217 | 0.177441273 | 0.149258 |
| GO_BP_Profile0 | GO:0071459 | protein localization to chromosome, centromeric region | 2/224 | 21/17913 | 0.027965217 | 0.177441273 | 0.149258 |
| GO_BP_Profile0 | GO:2000104 | negative regulation of DNA-dependent DNA replication | 2/224 | 21/17913 | 0.027965217 | 0.177441273 | 0.149258 |
| GO_BP_Profile0 | GO:0008630 | intrinsic apoptotic signaling pathway in response to DNA damage | 4/224 | 92/17913 | 0.028320987 | 0.179277819 | 0.150803 |
| GO_BP_Profile0 | GO:0001701 | in utero embryonic development | 9/224 | 342/17913 | 0.028542097 | 0.180255343 | 0.151626 |
| GO_BP_Profile0 | GO:0032231 | regulation of actin filament bundle assembly | 4/224 | 94/17913 | 0.030326494 | 0.19107812 | 0.160729 |
| GO_BP_Profile0 | GO:0043496 | regulation of protein homodimerization activity | 2/224 | 22/17913 | 0.030512463 | 0.191357745 | 0.160965 |
| GO_BP_Profile0 | GO:2000773 | negative regulation of cellular senescence | 2/224 | 22/17913 | 0.030512463 | 0.191357745 | 0.160965 |
| GO_BP_Profile0 | GO:0097711 | ciliary basal body-plasma membrane docking | 4/224 | 95/17913 | 0.031359935 | 0.196217368 | 0.165052 |
| GO_BP_Profile0 | GO:0009123 | nucleoside monophosphate metabolic process | 9/224 | 349/17913 | 0.031895549 | 0.199107782 | 0.167484 |
| GO_BP_Profile0 | GO:0007141 | male meiosis I | 2/224 | 23/17913 | 0.033147894 | 0.204097397 | 0.171681 |
| GO_BP_Profile0 | GO:0044818 | mitotic G2/M transition checkpoint | 2/224 | 23/17913 | 0.033147894 | 0.204097397 | 0.171681 |
| GO_BP_Profile0 | GO:0051570 | regulation of histone H3-K9 methylation | 2/224 | 23/17913 | 0.033147894 | 0.204097397 | 0.171681 |
| GO_BP_Profile0 | GO:0061436 | establishment of skin barrier | 2/224 | 23/17913 | 0.033147894 | 0.204097397 | 0.171681 |
| GO_BP_Profile0 | GO:0071677 | positive regulation of mononuclear cell migration | 2/224 | 23/17913 | 0.033147894 | 0.204097397 | 0.171681 |
| GO_BP_Profile0 | GO:1902254 | negative regulation of intrinsic apoptotic signaling pathway by p53 class mediator | 2/224 | 23/17913 | 0.033147894 | 0.204097397 | 0.171681 |
| GO_BP_Profile0 | GO:0019886 | antigen processing and presentation of exogenous peptide antigen via MHC class II | 4/224 | 97/17913 | 0.033488427 | 0.204377336 | 0.171916 |
| GO_BP_Profile0 | GO:1902750 | negative regulation of cell cycle G2/M phase transition | 4/224 | 97/17913 | 0.033488427 | 0.204377336 | 0.171916 |
| GO_BP_Profile0 | GO:0030099 | myeloid cell differentiation | 10/224 | 411/17913 | 0.034425958 | 0.204377336 | 0.171916 |
| GO_BP_Profile0 | GO:0061351 | neural precursor cell proliferation | 5/224 | 144/17913 | 0.034825055 | 0.204377336 | 0.171916 |
| GO_BP_Profile0 | GO:0001824 | blastocyst development | 4/224 | 99/17913 | 0.035699318 | 0.204377336 | 0.171916 |
| GO_BP_Profile0 | GO:0002495 | antigen processing and presentation of peptide antigen via MHC class II | 4/224 | 100/17913 | 0.036835737 | 0.204377336 | 0.171916 |
| GO_BP_Profile0 | GO:0000447 | endonucleolytic cleavage in ITS1 to separate SSU-rRNA from 5.8S rRNA and LSU-rRNA from tricistronic rRNA transcript (SSU-rRNA, 5.8S rRNA, LSU-rRNA) | 1/224 | 3/17913 | 0.037049536 | 0.204377336 | 0.171916 |
| GO_BP_Profile0 | GO:0002424 | T cell mediated immune response to tumor cell | 1/224 | 3/17913 | 0.037049536 | 0.204377336 | 0.171916 |
| GO_BP_Profile0 | GO:0002840 | regulation of T cell mediated immune response to tumor cell | 1/224 | 3/17913 | 0.037049536 | 0.204377336 | 0.171916 |
| GO_BP_Profile0 | GO:0002949 | tRNA threonylcarbamoyladenosine modification | 1/224 | 3/17913 | 0.037049536 | 0.204377336 | 0.171916 |
| GO_BP_Profile0 | GO:0006065 | UDP-glucuronate biosynthetic process | 1/224 | 3/17913 | 0.037049536 | 0.204377336 | 0.171916 |
| GO_BP_Profile0 | GO:0006203 | dGTP catabolic process | 1/224 | 3/17913 | 0.037049536 | 0.204377336 | 0.171916 |
| GO_BP_Profile0 | GO:0006565 | L-serine catabolic process | 1/224 | 3/17913 | 0.037049536 | 0.204377336 | 0.171916 |
| GO_BP_Profile0 | GO:0007056 | spindle assembly involved in female meiosis | 1/224 | 3/17913 | 0.037049536 | 0.204377336 | 0.171916 |
| GO_BP_Profile0 | GO:0009177 | pyrimidine deoxyribonucleoside monophosphate biosynthetic process | 1/224 | 3/17913 | 0.037049536 | 0.204377336 | 0.171916 |
| GO_BP_Profile0 | GO:0010621 | negative regulation of transcription by transcription factor localization | 1/224 | 3/17913 | 0.037049536 | 0.204377336 | 0.171916 |
| GO_BP_Profile0 | GO:0030997 | regulation of centriole-centriole cohesion | 1/224 | 3/17913 | 0.037049536 | 0.204377336 | 0.171916 |
| GO_BP_Profile0 | GO:0032425 | positive regulation of mismatch repair | 1/224 | 3/17913 | 0.037049536 | 0.204377336 | 0.171916 |
| GO_BP_Profile0 | GO:0034165 | positive regulation of toll-like receptor 9 signaling pathway | 1/224 | 3/17913 | 0.037049536 | 0.204377336 | 0.171916 |
| GO_BP_Profile0 | GO:0034343 | type III interferon production | 1/224 | 3/17913 | 0.037049536 | 0.204377336 | 0.171916 |
| GO_BP_Profile0 | GO:0034344 | regulation of type III interferon production | 1/224 | 3/17913 | 0.037049536 | 0.204377336 | 0.171916 |
| GO_BP_Profile0 | GO:0034421 | post-translational protein acetylation | 1/224 | 3/17913 | 0.037049536 | 0.204377336 | 0.171916 |
| GO_BP_Profile0 | GO:0035983 | response to trichostatin A | 1/224 | 3/17913 | 0.037049536 | 0.204377336 | 0.171916 |
| GO_BP_Profile0 | GO:0035984 | cellular response to trichostatin A | 1/224 | 3/17913 | 0.037049536 | 0.204377336 | 0.171916 |
| GO_BP_Profile0 | GO:0043988 | histone H3-S28 phosphorylation | 1/224 | 3/17913 | 0.037049536 | 0.204377336 | 0.171916 |
| GO_BP_Profile0 | GO:0044205 | 'de novo' UMP biosynthetic process | 1/224 | 3/17913 | 0.037049536 | 0.204377336 | 0.171916 |
| GO_BP_Profile0 | GO:0046061 | dATP catabolic process | 1/224 | 3/17913 | 0.037049536 | 0.204377336 | 0.171916 |
| GO_BP_Profile0 | GO:0060083 | smooth muscle contraction involved in micturition | 1/224 | 3/17913 | 0.037049536 | 0.204377336 | 0.171916 |
| GO_BP_Profile0 | GO:0060086 | circadian temperature homeostasis | 1/224 | 3/17913 | 0.037049536 | 0.204377336 | 0.171916 |
| GO_BP_Profile0 | GO:0060741 | prostate gland stromal morphogenesis | 1/224 | 3/17913 | 0.037049536 | 0.204377336 | 0.171916 |
| GO_BP_Profile0 | GO:0060838 | lymphatic endothelial cell fate commitment | 1/224 | 3/17913 | 0.037049536 | 0.204377336 | 0.171916 |
| GO_BP_Profile0 | GO:0061107 | seminal vesicle development | 1/224 | 3/17913 | 0.037049536 | 0.204377336 | 0.171916 |
| GO_BP_Profile0 | GO:0071314 | cellular response to cocaine | 1/224 | 3/17913 | 0.037049536 | 0.204377336 | 0.171916 |
| GO_BP_Profile0 | GO:0071373 | cellular response to luteinizing hormone stimulus | 1/224 | 3/17913 | 0.037049536 | 0.204377336 | 0.171916 |
| GO_BP_Profile0 | GO:0071930 | negative regulation of transcription involved in G1/S transition of mitotic cell cycle | 1/224 | 3/17913 | 0.037049536 | 0.204377336 | 0.171916 |
| GO_BP_Profile0 | GO:0072262 | metanephric glomerular mesangial cell proliferation involved in metanephros development | 1/224 | 3/17913 | 0.037049536 | 0.204377336 | 0.171916 |
| GO_BP_Profile0 | GO:0098758 | response to interleukin-8 | 1/224 | 3/17913 | 0.037049536 | 0.204377336 | 0.171916 |
| GO_BP_Profile0 | GO:0098759 | cellular response to interleukin-8 | 1/224 | 3/17913 | 0.037049536 | 0.204377336 | 0.171916 |
| GO_BP_Profile0 | GO:0098920 | retrograde trans-synaptic signaling by lipid | 1/224 | 3/17913 | 0.037049536 | 0.204377336 | 0.171916 |
| GO_BP_Profile0 | GO:0098921 | retrograde trans-synaptic signaling by endocannabinoid | 1/224 | 3/17913 | 0.037049536 | 0.204377336 | 0.171916 |
| GO_BP_Profile0 | GO:0098961 | dendritic transport of ribonucleoprotein complex | 1/224 | 3/17913 | 0.037049536 | 0.204377336 | 0.171916 |
| GO_BP_Profile0 | GO:0098963 | dendritic transport of messenger ribonucleoprotein complex | 1/224 | 3/17913 | 0.037049536 | 0.204377336 | 0.171916 |
| GO_BP_Profile0 | GO:1902775 | mitochondrial large ribosomal subunit assembly | 1/224 | 3/17913 | 0.037049536 | 0.204377336 | 0.171916 |
| GO_BP_Profile0 | GO:1902990 | mitotic telomere maintenance via semi-conservative replication | 1/224 | 3/17913 | 0.037049536 | 0.204377336 | 0.171916 |
| GO_BP_Profile0 | GO:1903614 | negative regulation of protein tyrosine phosphatase activity | 1/224 | 3/17913 | 0.037049536 | 0.204377336 | 0.171916 |
| GO_BP_Profile0 | GO:1905319 | mesenchymal stem cell migration | 1/224 | 3/17913 | 0.037049536 | 0.204377336 | 0.171916 |
| GO_BP_Profile0 | GO:1905320 | regulation of mesenchymal stem cell migration | 1/224 | 3/17913 | 0.037049536 | 0.204377336 | 0.171916 |
| GO_BP_Profile0 | GO:1905322 | positive regulation of mesenchymal stem cell migration | 1/224 | 3/17913 | 0.037049536 | 0.204377336 | 0.171916 |
| GO_BP_Profile0 | GO:1905463 | negative regulation of DNA duplex unwinding | 1/224 | 3/17913 | 0.037049536 | 0.204377336 | 0.171916 |
| GO_BP_Profile0 | GO:2000520 | regulation of immunological synapse formation | 1/224 | 3/17913 | 0.037049536 | 0.204377336 | 0.171916 |
| GO_BP_Profile0 | GO:2001189 | negative regulation of T cell activation via T cell receptor contact with antigen bound to MHC molecule on antigen presenting cell | 1/224 | 3/17913 | 0.037049536 | 0.204377336 | 0.171916 |
| GO_BP_Profile0 | GO:0071236 | cellular response to antibiotic | 5/224 | 147/17913 | 0.037541103 | 0.206540905 | 0.173736 |
| GO_BP_Profile0 | GO:0090342 | regulation of cell aging | 3/224 | 59/17913 | 0.037594571 | 0.206540905 | 0.173736 |
| GO_BP_Profile0 | GO:0002504 | antigen processing and presentation of peptide or polysaccharide antigen via MHC class II | 4/224 | 101/17913 | 0.037992834 | 0.208305539 | 0.175221 |
| GO_BP_Profile0 | GO:0035690 | cellular response to drug | 9/224 | 362/17913 | 0.038833155 | 0.212481819 | 0.178733 |
| GO_BP_Profile0 | GO:0002562 | somatic diversification of immune receptors via germline recombination within a single locus | 3/224 | 60/17913 | 0.039219662 | 0.213731345 | 0.179785 |
| GO_BP_Profile0 | GO:0016444 | somatic cell DNA recombination | 3/224 | 60/17913 | 0.039219662 | 0.213731345 | 0.179785 |
| GO_BP_Profile0 | GO:0032091 | negative regulation of protein binding | 4/224 | 103/17913 | 0.040369114 | 0.219552747 | 0.184681 |
| GO_BP_Profile0 | GO:0007623 | circadian rhythm | 6/224 | 201/17913 | 0.040932623 | 0.222170443 | 0.186883 |
| GO_BP_Profile0 | GO:0033561 | regulation of water loss via skin | 2/224 | 26/17913 | 0.04155772 | 0.22421261 | 0.188601 |
| GO_BP_Profile0 | GO:0060706 | cell differentiation involved in embryonic placenta development | 2/224 | 26/17913 | 0.04155772 | 0.22421261 | 0.188601 |
| GO_BP_Profile0 | GO:1904353 | regulation of telomere capping | 2/224 | 26/17913 | 0.04155772 | 0.22421261 | 0.188601 |
| GO_BP_Profile0 | GO:0009394 | 2'-deoxyribonucleotide metabolic process | 2/224 | 27/17913 | 0.044520563 | 0.239719288 | 0.201645 |
| GO_BP_Profile0 | GO:0006406 | mRNA export from nucleus | 4/224 | 107/17913 | 0.045370105 | 0.243324195 | 0.204677 |
| GO_BP_Profile0 | GO:0071427 | mRNA-containing ribonucleoprotein complex export from nucleus | 4/224 | 107/17913 | 0.045370105 | 0.243324195 | 0.204677 |
| GO_BP_Profile0 | GO:0040001 | establishment of mitotic spindle localization | 2/224 | 28/17913 | 0.047559173 | 0.244831641 | 0.205945 |
| GO_BP_Profile0 | GO:0045737 | positive regulation of cyclin-dependent protein serine/threonine kinase activity | 2/224 | 28/17913 | 0.047559173 | 0.244831641 | 0.205945 |
| GO_BP_Profile0 | GO:0071168 | protein localization to chromatin | 2/224 | 28/17913 | 0.047559173 | 0.244831641 | 0.205945 |
| GO_BP_Profile0 | GO:0002285 | lymphocyte activation involved in immune response | 5/224 | 158/17913 | 0.048589138 | 0.244831641 | 0.205945 |
| GO_BP_Profile0 | GO:0046777 | protein autophosphorylation | 6/224 | 210/17913 | 0.048805703 | 0.244831641 | 0.205945 |
| GO_BP_Profile0 | GO:0000480 | endonucleolytic cleavage in 5'-ETS of tricistronic rRNA transcript (SSU-rRNA, 5.8S rRNA, LSU-rRNA) | 1/224 | 4/17913 | 0.049093137 | 0.244831641 | 0.205945 |
| GO_BP_Profile0 | GO:0000912 | assembly of actomyosin apparatus involved in cytokinesis | 1/224 | 4/17913 | 0.049093137 | 0.244831641 | 0.205945 |
| GO_BP_Profile0 | GO:0000915 | actomyosin contractile ring assembly | 1/224 | 4/17913 | 0.049093137 | 0.244831641 | 0.205945 |
| GO_BP_Profile0 | GO:0006269 | DNA replication, synthesis of RNA primer | 1/224 | 4/17913 | 0.049093137 | 0.244831641 | 0.205945 |
| GO_BP_Profile0 | GO:0007296 | vitellogenesis | 1/224 | 4/17913 | 0.049093137 | 0.244831641 | 0.205945 |
| GO_BP_Profile0 | GO:0009176 | pyrimidine deoxyribonucleoside monophosphate metabolic process | 1/224 | 4/17913 | 0.049093137 | 0.244831641 | 0.205945 |
| GO_BP_Profile0 | GO:0014834 | skeletal muscle satellite cell maintenance involved in skeletal muscle regeneration | 1/224 | 4/17913 | 0.049093137 | 0.244831641 | 0.205945 |
| GO_BP_Profile0 | GO:0032423 | regulation of mismatch repair | 1/224 | 4/17913 | 0.049093137 | 0.244831641 | 0.205945 |
| GO_BP_Profile0 | GO:0034465 | response to carbon monoxide | 1/224 | 4/17913 | 0.049093137 | 0.244831641 | 0.205945 |
| GO_BP_Profile0 | GO:0034699 | response to luteinizing hormone | 1/224 | 4/17913 | 0.049093137 | 0.244831641 | 0.205945 |
| GO_BP_Profile0 | GO:0035549 | positive regulation of interferon-beta secretion | 1/224 | 4/17913 | 0.049093137 | 0.244831641 | 0.205945 |
| GO_BP_Profile0 | GO:0035711 | T-helper 1 cell activation | 1/224 | 4/17913 | 0.049093137 | 0.244831641 | 0.205945 |
| GO_BP_Profile0 | GO:0036146 | cellular response to mycotoxin | 1/224 | 4/17913 | 0.049093137 | 0.244831641 | 0.205945 |
| GO_BP_Profile0 | GO:0044878 | mitotic cytokinesis checkpoint | 1/224 | 4/17913 | 0.049093137 | 0.244831641 | 0.205945 |
| GO_BP_Profile0 | GO:0046070 | dGTP metabolic process | 1/224 | 4/17913 | 0.049093137 | 0.244831641 | 0.205945 |
| GO_BP_Profile0 | GO:0046398 | UDP-glucuronate metabolic process | 1/224 | 4/17913 | 0.049093137 | 0.244831641 | 0.205945 |
| GO_BP_Profile0 | GO:0060623 | regulation of chromosome condensation | 1/224 | 4/17913 | 0.049093137 | 0.244831641 | 0.205945 |
| GO_BP_Profile0 | GO:0071962 | mitotic sister chromatid cohesion, centromeric | 1/224 | 4/17913 | 0.049093137 | 0.244831641 | 0.205945 |
| GO_BP_Profile0 | GO:0072429 | response to intra-S DNA damage checkpoint signaling | 1/224 | 4/17913 | 0.049093137 | 0.244831641 | 0.205945 |
| GO_BP_Profile0 | GO:0075732 | viral penetration into host nucleus | 1/224 | 4/17913 | 0.049093137 | 0.244831641 | 0.205945 |
| GO_BP_Profile0 | GO:0090074 | negative regulation of protein homodimerization activity | 1/224 | 4/17913 | 0.049093137 | 0.244831641 | 0.205945 |
| GO_BP_Profile0 | GO:0090116 | C-5 methylation of cytosine | 1/224 | 4/17913 | 0.049093137 | 0.244831641 | 0.205945 |
| GO_BP_Profile0 | GO:0097742 | de novo centriole assembly | 1/224 | 4/17913 | 0.049093137 | 0.244831641 | 0.205945 |
| GO_BP_Profile0 | GO:0097752 | regulation of DNA stability | 1/224 | 4/17913 | 0.049093137 | 0.244831641 | 0.205945 |
| GO_BP_Profile0 | GO:0098535 | de novo centriole assembly involved in multi-ciliated epithelial cell differentiation | 1/224 | 4/17913 | 0.049093137 | 0.244831641 | 0.205945 |
| GO_BP_Profile0 | GO:1900240 | negative regulation of phenotypic switching | 1/224 | 4/17913 | 0.049093137 | 0.244831641 | 0.205945 |
| GO_BP_Profile0 | GO:1902594 | multi-organism nuclear import | 1/224 | 4/17913 | 0.049093137 | 0.244831641 | 0.205945 |
| GO_BP_Profile0 | GO:1905340 | regulation of protein localization to kinetochore | 1/224 | 4/17913 | 0.049093137 | 0.244831641 | 0.205945 |
| GO_BP_Profile0 | GO:1905342 | positive regulation of protein localization to kinetochore | 1/224 | 4/17913 | 0.049093137 | 0.244831641 | 0.205945 |
| GO_BP_Profile0 | GO:1905774 | regulation of DNA helicase activity | 1/224 | 4/17913 | 0.049093137 | 0.244831641 | 0.205945 |
| GO_BP_Profile0 | GO:1990086 | lens fiber cell apoptotic process | 1/224 | 4/17913 | 0.049093137 | 0.244831641 | 0.205945 |
| GO_BP_Profile0 | GO:2000182 | regulation of progesterone biosynthetic process | 1/224 | 4/17913 | 0.049093137 | 0.244831641 | 0.205945 |
| GO_BP_Profile0 | GO:2001200 | positive regulation of dendritic cell differentiation | 1/224 | 4/17913 | 0.049093137 | 0.244831641 | 0.205945 |
| GO_BP_Profile0 | GO:0007266 | Rho protein signal transduction | 5/224 | 159/17913 | 0.049679122 | 0.247297729 | 0.20802 |
| GO_BP_Profile11 | GO:0042311 | vasodilation | 2月25日 | 28/17913 | 0.000691302 | 0.111340615 | 0.077698 |
| GO_BP_Profile11 | GO:0015695 | organic cation transport | 2月25日 | 31/17913 | 0.000848233 | 0.111340615 | 0.077698 |
| GO_BP_Profile11 | GO:0051615 | histamine uptake | 1月25日 | 1/17913 | 0.001395634 | 0.111340615 | 0.077698 |
| GO_BP_Profile11 | GO:0052066 | entry of symbiont into host cell by promotion of host phagocytosis | 1月25日 | 1/17913 | 0.001395634 | 0.111340615 | 0.077698 |
| GO_BP_Profile11 | GO:0052190 | modulation by symbiont of host phagocytosis | 1月25日 | 1/17913 | 0.001395634 | 0.111340615 | 0.077698 |
| GO_BP_Profile11 | GO:0052191 | positive regulation by symbiont of host phagocytosis | 1月25日 | 1/17913 | 0.001395634 | 0.111340615 | 0.077698 |
| GO_BP_Profile11 | GO:0052231 | modulation of phagocytosis in other organism involved in symbiotic interaction | 1月25日 | 1/17913 | 0.001395634 | 0.111340615 | 0.077698 |
| GO_BP_Profile11 | GO:0052370 | entry of organism into cell of other organism by promotion of phagocytosis in other organism involved in symbiotic interaction | 1月25日 | 1/17913 | 0.001395634 | 0.111340615 | 0.077698 |
| GO_BP_Profile11 | GO:0052522 | positive regulation by organism of phagocytosis in other organism involved in symbiotic interaction | 1月25日 | 1/17913 | 0.001395634 | 0.111340615 | 0.077698 |
| GO_BP_Profile11 | GO:0051937 | catecholamine transport | 2月25日 | 54/17913 | 0.002559584 | 0.123924125 | 0.08648 |
| GO_BP_Profile11 | GO:0097755 | positive regulation of blood vessel diameter | 2月25日 | 54/17913 | 0.002559584 | 0.123924125 | 0.08648 |
| GO_BP_Profile11 | GO:0014057 | positive regulation of acetylcholine secretion, neurotransmission | 1月25日 | 2/17913 | 0.002789399 | 0.123924125 | 0.08648 |
| GO_BP_Profile11 | GO:2000536 | negative regulation of entry of bacterium into host cell | 1月25日 | 2/17913 | 0.002789399 | 0.123924125 | 0.08648 |
| GO_BP_Profile11 | GO:2000857 | positive regulation of mineralocorticoid secretion | 1月25日 | 2/17913 | 0.002789399 | 0.123924125 | 0.08648 |
| GO_BP_Profile11 | GO:2000860 | positive regulation of aldosterone secretion | 1月25日 | 2/17913 | 0.002789399 | 0.123924125 | 0.08648 |
| GO_BP_Profile11 | GO:0038034 | signal transduction in absence of ligand | 2月25日 | 62/17913 | 0.003359361 | 0.123924125 | 0.08648 |
| GO_BP_Profile11 | GO:0097192 | extrinsic apoptotic signaling pathway in absence of ligand | 2月25日 | 62/17913 | 0.003359361 | 0.123924125 | 0.08648 |
| GO_BP_Profile11 | GO:0015844 | monoamine transport | 2月25日 | 66/17913 | 0.003797613 | 0.123924125 | 0.08648 |
| GO_BP_Profile11 | GO:0015850 | organic hydroxy compound transport | 3月25日 | 228/17913 | 0.003806156 | 0.123924125 | 0.08648 |
| GO_BP_Profile11 | GO:0014055 | acetylcholine secretion, neurotransmission | 1月25日 | 3/17913 | 0.004181296 | 0.123924125 | 0.08648 |
| GO_BP_Profile11 | GO:0014056 | regulation of acetylcholine secretion, neurotransmission | 1月25日 | 3/17913 | 0.004181296 | 0.123924125 | 0.08648 |
| GO_BP_Profile11 | GO:0045715 | negative regulation of low-density lipoprotein particle receptor biosynthetic process | 1月25日 | 3/17913 | 0.004181296 | 0.123924125 | 0.08648 |
| GO_BP_Profile11 | GO:0060291 | long-term synaptic potentiation | 2月25日 | 74/17913 | 0.004749476 | 0.123924125 | 0.08648 |
| GO_BP_Profile11 | GO:0007204 | positive regulation of cytosolic calcium ion concentration | 3月25日 | 255/17913 | 0.005201807 | 0.123924125 | 0.08648 |
| GO_BP_Profile11 | GO:0015870 | acetylcholine transport | 1月25日 | 4/17913 | 0.005571327 | 0.123924125 | 0.08648 |
| GO_BP_Profile11 | GO:0030035 | microspike assembly | 1月25日 | 4/17913 | 0.005571327 | 0.123924125 | 0.08648 |
| GO_BP_Profile11 | GO:0050748 | negative regulation of lipoprotein metabolic process | 1月25日 | 4/17913 | 0.005571327 | 0.123924125 | 0.08648 |
| GO_BP_Profile11 | GO:0061526 | acetylcholine secretion | 1月25日 | 4/17913 | 0.005571327 | 0.123924125 | 0.08648 |
| GO_BP_Profile11 | GO:2001013 | epithelial cell proliferation involved in renal tubule morphogenesis | 1月25日 | 4/17913 | 0.005571327 | 0.123924125 | 0.08648 |
| GO_BP_Profile11 | GO:0015696 | ammonium transport | 2月25日 | 81/17913 | 0.005663377 | 0.123924125 | 0.08648 |
| GO_BP_Profile11 | GO:0032224 | positive regulation of synaptic transmission, cholinergic | 1月25日 | 5/17913 | 0.006959496 | 0.123924125 | 0.08648 |
| GO_BP_Profile11 | GO:0038044 | transforming growth factor-beta secretion | 1月25日 | 5/17913 | 0.006959496 | 0.123924125 | 0.08648 |
| GO_BP_Profile11 | GO:0051480 | regulation of cytosolic calcium ion concentration | 3月25日 | 289/17913 | 0.007350129 | 0.123924125 | 0.08648 |
| GO_BP_Profile11 | GO:0007189 | adenylate cyclase-activating G protein-coupled receptor signaling pathway | 2月25日 | 94/17913 | 0.007556199 | 0.123924125 | 0.08648 |
| GO_BP_Profile11 | GO:0007229 | integrin-mediated signaling pathway | 2月25日 | 94/17913 | 0.007556199 | 0.123924125 | 0.08648 |
| GO_BP_Profile11 | GO:0010871 | negative regulation of receptor biosynthetic process | 1月25日 | 6/17913 | 0.008345805 | 0.123924125 | 0.08648 |
| GO_BP_Profile11 | GO:0035931 | mineralocorticoid secretion | 1月25日 | 6/17913 | 0.008345805 | 0.123924125 | 0.08648 |
| GO_BP_Profile11 | GO:0035932 | aldosterone secretion | 1月25日 | 6/17913 | 0.008345805 | 0.123924125 | 0.08648 |
| GO_BP_Profile11 | GO:0045938 | positive regulation of circadian sleep/wake cycle, sleep | 1月25日 | 6/17913 | 0.008345805 | 0.123924125 | 0.08648 |
| GO_BP_Profile11 | GO:1901374 | acetate ester transport | 1月25日 | 6/17913 | 0.008345805 | 0.123924125 | 0.08648 |
| GO_BP_Profile11 | GO:2000855 | regulation of mineralocorticoid secretion | 1月25日 | 6/17913 | 0.008345805 | 0.123924125 | 0.08648 |
| GO_BP_Profile11 | GO:2000858 | regulation of aldosterone secretion | 1月25日 | 6/17913 | 0.008345805 | 0.123924125 | 0.08648 |
| GO_BP_Profile11 | GO:2001237 | negative regulation of extrinsic apoptotic signaling pathway | 2月25日 | 100/17913 | 0.00851354 | 0.123924125 | 0.08648 |
| GO_BP_Profile11 | GO:1901214 | regulation of neuron death | 3月25日 | 313/17913 | 0.009141825 | 0.123924125 | 0.08648 |
| GO_BP_Profile11 | GO:0002248 | connective tissue replacement involved in inflammatory response wound healing | 1月25日 | 7/17913 | 0.009730255 | 0.123924125 | 0.08648 |
| GO_BP_Profile11 | GO:0032222 | regulation of synaptic transmission, cholinergic | 1月25日 | 7/17913 | 0.009730255 | 0.123924125 | 0.08648 |
| GO_BP_Profile11 | GO:0035234 | ectopic germ cell programmed cell death | 1月25日 | 7/17913 | 0.009730255 | 0.123924125 | 0.08648 |
| GO_BP_Profile11 | GO:1901388 | regulation of transforming growth factor beta activation | 1月25日 | 7/17913 | 0.009730255 | 0.123924125 | 0.08648 |
| GO_BP_Profile11 | GO:2000535 | regulation of entry of bacterium into host cell | 1月25日 | 7/17913 | 0.009730255 | 0.123924125 | 0.08648 |
| GO_BP_Profile11 | GO:2000848 | positive regulation of corticosteroid hormone secretion | 1月25日 | 7/17913 | 0.009730255 | 0.123924125 | 0.08648 |
| GO_BP_Profile11 | GO:0030198 | extracellular matrix organization | 3月25日 | 334/17913 | 0.010903136 | 0.123924125 | 0.08648 |
| GO_BP_Profile11 | GO:0002246 | wound healing involved in inflammatory response | 1月25日 | 8/17913 | 0.01111285 | 0.123924125 | 0.08648 |
| GO_BP_Profile11 | GO:0038027 | apolipoprotein A-I-mediated signaling pathway | 1月25日 | 8/17913 | 0.01111285 | 0.123924125 | 0.08648 |
| GO_BP_Profile11 | GO:0072102 | glomerulus morphogenesis | 1月25日 | 8/17913 | 0.01111285 | 0.123924125 | 0.08648 |
| GO_BP_Profile11 | GO:1990034 | calcium ion export across plasma membrane | 1月25日 | 8/17913 | 0.01111285 | 0.123924125 | 0.08648 |
| GO_BP_Profile11 | GO:0070997 | neuron death | 3月25日 | 347/17913 | 0.012085924 | 0.123924125 | 0.08648 |
| GO_BP_Profile11 | GO:0042107 | cytokine metabolic process | 2月25日 | 121/17913 | 0.012265558 | 0.123924125 | 0.08648 |
| GO_BP_Profile11 | GO:0032368 | regulation of lipid transport | 2月25日 | 122/17913 | 0.012459394 | 0.123924125 | 0.08648 |
| GO_BP_Profile11 | GO:0036363 | transforming growth factor beta activation | 1月25日 | 9/17913 | 0.012493592 | 0.123924125 | 0.08648 |
| GO_BP_Profile11 | GO:0045714 | regulation of low-density lipoprotein particle receptor biosynthetic process | 1月25日 | 9/17913 | 0.012493592 | 0.123924125 | 0.08648 |
| GO_BP_Profile11 | GO:2000833 | positive regulation of steroid hormone secretion | 1月25日 | 9/17913 | 0.012493592 | 0.123924125 | 0.08648 |
| GO_BP_Profile11 | GO:0035296 | regulation of tube diameter | 2月25日 | 125/17913 | 0.013048983 | 0.123924125 | 0.08648 |
| GO_BP_Profile11 | GO:0046887 | positive regulation of hormone secretion | 2月25日 | 125/17913 | 0.013048983 | 0.123924125 | 0.08648 |
| GO_BP_Profile11 | GO:0097746 | regulation of blood vessel diameter | 2月25日 | 125/17913 | 0.013048983 | 0.123924125 | 0.08648 |
| GO_BP_Profile11 | GO:0019932 | second-messenger-mediated signaling | 3月25日 | 360/17913 | 0.013340416 | 0.123924125 | 0.08648 |
| GO_BP_Profile11 | GO:0060078 | regulation of postsynaptic membrane potential | 2月25日 | 127/17913 | 0.013448738 | 0.123924125 | 0.08648 |
| GO_BP_Profile11 | GO:0001660 | fever generation | 1月25日 | 10/17913 | 0.013872483 | 0.123924125 | 0.08648 |
| GO_BP_Profile11 | GO:0014049 | positive regulation of glutamate secretion | 1月25日 | 10/17913 | 0.013872483 | 0.123924125 | 0.08648 |
| GO_BP_Profile11 | GO:0033690 | positive regulation of osteoblast proliferation | 1月25日 | 10/17913 | 0.013872483 | 0.123924125 | 0.08648 |
| GO_BP_Profile11 | GO:0045713 | low-density lipoprotein particle receptor biosynthetic process | 1月25日 | 10/17913 | 0.013872483 | 0.123924125 | 0.08648 |
| GO_BP_Profile11 | GO:1903265 | positive regulation of tumor necrosis factor-mediated signaling pathway | 1月25日 | 10/17913 | 0.013872483 | 0.123924125 | 0.08648 |
| GO_BP_Profile11 | GO:0050880 | regulation of blood vessel size | 2月25日 | 133/17913 | 0.014679807 | 0.123924125 | 0.08648 |
| GO_BP_Profile11 | GO:0006874 | cellular calcium ion homeostasis | 3月25日 | 375/17913 | 0.014877937 | 0.123924125 | 0.08648 |
| GO_BP_Profile11 | GO:0035150 | regulation of tube size | 2月25日 | 134/17913 | 0.014889584 | 0.123924125 | 0.08648 |
| GO_BP_Profile11 | GO:0010623 | programmed cell death involved in cell development | 1月25日 | 11/17913 | 0.015249525 | 0.123924125 | 0.08648 |
| GO_BP_Profile11 | GO:0015697 | quaternary ammonium group transport | 1月25日 | 11/17913 | 0.015249525 | 0.123924125 | 0.08648 |
| GO_BP_Profile11 | GO:0098703 | calcium ion import across plasma membrane | 1月25日 | 11/17913 | 0.015249525 | 0.123924125 | 0.08648 |
| GO_BP_Profile11 | GO:1901660 | calcium ion export | 1月25日 | 11/17913 | 0.015249525 | 0.123924125 | 0.08648 |
| GO_BP_Profile11 | GO:1903532 | positive regulation of secretion by cell | 3月25日 | 381/17913 | 0.015520155 | 0.123924125 | 0.08648 |
| GO_BP_Profile11 | GO:0038127 | ERBB signaling pathway | 2月25日 | 138/17913 | 0.015741692 | 0.123924125 | 0.08648 |
| GO_BP_Profile11 | GO:0043062 | extracellular structure organization | 3月25日 | 387/17913 | 0.016178004 | 0.123924125 | 0.08648 |
| GO_BP_Profile11 | GO:0055074 | calcium ion homeostasis | 3月25日 | 388/17913 | 0.016289168 | 0.123924125 | 0.08648 |
| GO_BP_Profile11 | GO:0001973 | adenosine receptor signaling pathway | 1月25日 | 12/17913 | 0.016624721 | 0.123924125 | 0.08648 |
| GO_BP_Profile11 | GO:0007288 | sperm axoneme assembly | 1月25日 | 12/17913 | 0.016624721 | 0.123924125 | 0.08648 |
| GO_BP_Profile11 | GO:0032230 | positive regulation of synaptic transmission, GABAergic | 1月25日 | 12/17913 | 0.016624721 | 0.123924125 | 0.08648 |
| GO_BP_Profile11 | GO:0051608 | histamine transport | 1月25日 | 12/17913 | 0.016624721 | 0.123924125 | 0.08648 |
| GO_BP_Profile11 | GO:0090331 | negative regulation of platelet aggregation | 1月25日 | 12/17913 | 0.016624721 | 0.123924125 | 0.08648 |
| GO_BP_Profile11 | GO:1902656 | calcium ion import into cytosol | 1月25日 | 12/17913 | 0.016624721 | 0.123924125 | 0.08648 |
| GO_BP_Profile11 | GO:0072503 | cellular divalent inorganic cation homeostasis | 3月25日 | 392/17913 | 0.016738185 | 0.123924125 | 0.08648 |
| GO_BP_Profile11 | GO:0019933 | cAMP-mediated signaling | 2月25日 | 143/17913 | 0.016835801 | 0.123924125 | 0.08648 |
| GO_BP_Profile11 | GO:0014074 | response to purine-containing compound | 2月25日 | 145/17913 | 0.017282363 | 0.123924125 | 0.08648 |
| GO_BP_Profile11 | GO:0050806 | positive regulation of synaptic transmission | 2月25日 | 145/17913 | 0.017282363 | 0.123924125 | 0.08648 |
| GO_BP_Profile11 | GO:2001233 | regulation of apoptotic signaling pathway | 3月25日 | 397/17913 | 0.017309279 | 0.123924125 | 0.08648 |
| GO_BP_Profile11 | GO:0010642 | negative regulation of platelet-derived growth factor receptor signaling pathway | 1月25日 | 13/17913 | 0.017998074 | 0.123924125 | 0.08648 |
| GO_BP_Profile11 | GO:0010745 | negative regulation of macrophage derived foam cell differentiation | 1月25日 | 13/17913 | 0.017998074 | 0.123924125 | 0.08648 |
| GO_BP_Profile11 | GO:0014819 | regulation of skeletal muscle contraction | 1月25日 | 13/17913 | 0.017998074 | 0.123924125 | 0.08648 |
| GO_BP_Profile11 | GO:0031953 | negative regulation of protein autophosphorylation | 1月25日 | 13/17913 | 0.017998074 | 0.123924125 | 0.08648 |
| GO_BP_Profile11 | GO:0043116 | negative regulation of vascular permeability | 1月25日 | 13/17913 | 0.017998074 | 0.123924125 | 0.08648 |
| GO_BP_Profile11 | GO:0045086 | positive regulation of interleukin-2 biosynthetic process | 1月25日 | 13/17913 | 0.017998074 | 0.123924125 | 0.08648 |
| GO_BP_Profile11 | GO:0050746 | regulation of lipoprotein metabolic process | 1月25日 | 13/17913 | 0.017998074 | 0.123924125 | 0.08648 |
| GO_BP_Profile11 | GO:0070208 | protein heterotrimerization | 1月25日 | 13/17913 | 0.017998074 | 0.123924125 | 0.08648 |
| GO_BP_Profile11 | GO:0090594 | inflammatory response to wounding | 1月25日 | 13/17913 | 0.017998074 | 0.123924125 | 0.08648 |
| GO_BP_Profile11 | GO:1905952 | regulation of lipid localization | 2月25日 | 151/17913 | 0.018652247 | 0.123924125 | 0.08648 |
| GO_BP_Profile11 | GO:2001236 | regulation of extrinsic apoptotic signaling pathway | 2月25日 | 151/17913 | 0.018652247 | 0.123924125 | 0.08648 |
| GO_BP_Profile11 | GO:0051047 | positive regulation of secretion | 3月25日 | 409/17913 | 0.018724565 | 0.123924125 | 0.08648 |
| GO_BP_Profile11 | GO:0072507 | divalent inorganic cation homeostasis | 3月25日 | 412/17913 | 0.019088263 | 0.123924125 | 0.08648 |
| GO_BP_Profile11 | GO:0014048 | regulation of glutamate secretion | 1月25日 | 14/17913 | 0.019369585 | 0.123924125 | 0.08648 |
| GO_BP_Profile11 | GO:0035810 | positive regulation of urine volume | 1月25日 | 14/17913 | 0.019369585 | 0.123924125 | 0.08648 |
| GO_BP_Profile11 | GO:0090494 | dopamine uptake | 1月25日 | 14/17913 | 0.019369585 | 0.123924125 | 0.08648 |
| GO_BP_Profile11 | GO:2000846 | regulation of corticosteroid hormone secretion | 1月25日 | 14/17913 | 0.019369585 | 0.123924125 | 0.08648 |
| GO_BP_Profile11 | GO:0006816 | calcium ion transport | 3月25日 | 415/17913 | 0.01945592 | 0.123924125 | 0.08648 |
| GO_BP_Profile11 | GO:0014061 | regulation of norepinephrine secretion | 1月25日 | 15/17913 | 0.020739257 | 0.123924125 | 0.08648 |
| GO_BP_Profile11 | GO:0034111 | negative regulation of homotypic cell-cell adhesion | 1月25日 | 15/17913 | 0.020739257 | 0.123924125 | 0.08648 |
| GO_BP_Profile11 | GO:0035635 | entry of bacterium into host cell | 1月25日 | 15/17913 | 0.020739257 | 0.123924125 | 0.08648 |
| GO_BP_Profile11 | GO:0035815 | positive regulation of renal sodium excretion | 1月25日 | 15/17913 | 0.020739257 | 0.123924125 | 0.08648 |
| GO_BP_Profile11 | GO:0035930 | corticosteroid hormone secretion | 1月25日 | 15/17913 | 0.020739257 | 0.123924125 | 0.08648 |
| GO_BP_Profile11 | GO:0060134 | prepulse inhibition | 1月25日 | 15/17913 | 0.020739257 | 0.123924125 | 0.08648 |
| GO_BP_Profile11 | GO:0072160 | nephron tubule epithelial cell differentiation | 1月25日 | 15/17913 | 0.020739257 | 0.123924125 | 0.08648 |
| GO_BP_Profile11 | GO:0003018 | vascular process in circulatory system | 2月25日 | 161/17913 | 0.021034304 | 0.123924125 | 0.08648 |
| GO_BP_Profile11 | GO:0007188 | adenylate cyclase-modulating G protein-coupled receptor signaling pathway | 2月25日 | 162/17913 | 0.021279198 | 0.123924125 | 0.08648 |
| GO_BP_Profile11 | GO:0031649 | heat generation | 1月25日 | 16/17913 | 0.022107092 | 0.123924125 | 0.08648 |
| GO_BP_Profile11 | GO:0048243 | norepinephrine secretion | 1月25日 | 16/17913 | 0.022107092 | 0.123924125 | 0.08648 |
| GO_BP_Profile11 | GO:0090493 | catecholamine uptake | 1月25日 | 16/17913 | 0.022107092 | 0.123924125 | 0.08648 |
| GO_BP_Profile11 | GO:2000251 | positive regulation of actin cytoskeleton reorganization | 1月25日 | 16/17913 | 0.022107092 | 0.123924125 | 0.08648 |
| GO_BP_Profile11 | GO:0019935 | cyclic-nucleotide-mediated signaling | 2月25日 | 166/17913 | 0.022270756 | 0.123924125 | 0.08648 |
| GO_BP_Profile11 | GO:0033209 | tumor necrosis factor-mediated signaling pathway | 2月25日 | 167/17913 | 0.022521624 | 0.123924125 | 0.08648 |
| GO_BP_Profile11 | GO:0090066 | regulation of anatomical structure size | 3月25日 | 442/17913 | 0.02294346 | 0.123924125 | 0.08648 |
| GO_BP_Profile11 | GO:0022408 | negative regulation of cell-cell adhesion | 2月25日 | 169/17913 | 0.023026908 | 0.123924125 | 0.08648 |
| GO_BP_Profile11 | GO:0048167 | regulation of synaptic plasticity | 2月25日 | 169/17913 | 0.023026908 | 0.123924125 | 0.08648 |
| GO_BP_Profile11 | GO:0031000 | response to caffeine | 1月25日 | 17/17913 | 0.023473093 | 0.123924125 | 0.08648 |
| GO_BP_Profile11 | GO:0036270 | response to diuretic | 1月25日 | 17/17913 | 0.023473093 | 0.123924125 | 0.08648 |
| GO_BP_Profile11 | GO:0042753 | positive regulation of circadian rhythm | 1月25日 | 17/17913 | 0.023473093 | 0.123924125 | 0.08648 |
| GO_BP_Profile11 | GO:0045187 | regulation of circadian sleep/wake cycle, sleep | 1月25日 | 17/17913 | 0.023473093 | 0.123924125 | 0.08648 |
| GO_BP_Profile11 | GO:0051957 | positive regulation of amino acid transport | 1月25日 | 17/17913 | 0.023473093 | 0.123924125 | 0.08648 |
| GO_BP_Profile11 | GO:0060080 | inhibitory postsynaptic potential | 1月25日 | 17/17913 | 0.023473093 | 0.123924125 | 0.08648 |
| GO_BP_Profile11 | GO:2000831 | regulation of steroid hormone secretion | 1月25日 | 17/17913 | 0.023473093 | 0.123924125 | 0.08648 |
| GO_BP_Profile11 | GO:0051897 | positive regulation of protein kinase B signaling | 2月25日 | 173/17913 | 0.024051574 | 0.124707374 | 0.087026 |
| GO_BP_Profile11 | GO:0015893 | drug transport | 2月25日 | 175/17913 | 0.024570901 | 0.124707374 | 0.087026 |
| GO_BP_Profile11 | GO:0070838 | divalent metal ion transport | 3月25日 | 454/17913 | 0.024596955 | 0.124707374 | 0.087026 |
| GO_BP_Profile11 | GO:0007250 | activation of NF-kappaB-inducing kinase activity | 1月25日 | 18/17913 | 0.024837262 | 0.124707374 | 0.087026 |
| GO_BP_Profile11 | GO:0007271 | synaptic transmission, cholinergic | 1月25日 | 18/17913 | 0.024837262 | 0.124707374 | 0.087026 |
| GO_BP_Profile11 | GO:0010888 | negative regulation of lipid storage | 1月25日 | 18/17913 | 0.024837262 | 0.124707374 | 0.087026 |
| GO_BP_Profile11 | GO:0090330 | regulation of platelet aggregation | 1月25日 | 18/17913 | 0.024837262 | 0.124707374 | 0.087026 |
| GO_BP_Profile11 | GO:0072511 | divalent inorganic cation transport | 3月25日 | 458/17913 | 0.025162293 | 0.125461988 | 0.087553 |
| GO_BP_Profile11 | GO:0007187 | G protein-coupled receptor signaling pathway, coupled to cyclic nucleotide second messenger | 2月25日 | 178/17913 | 0.025358553 | 0.12556856 | 0.087627 |
| GO_BP_Profile11 | GO:0010544 | negative regulation of platelet activation | 1月25日 | 19/17913 | 0.026199602 | 0.127968125 | 0.089302 |
| GO_BP_Profile11 | GO:0045076 | regulation of interleukin-2 biosynthetic process | 1月25日 | 19/17913 | 0.026199602 | 0.127968125 | 0.089302 |
| GO_BP_Profile11 | GO:0006875 | cellular metal ion homeostasis | 3月25日 | 467/17913 | 0.026460222 | 0.128367835 | 0.089581 |
| GO_BP_Profile11 | GO:0010869 | regulation of receptor biosynthetic process | 1月25日 | 20/17913 | 0.027560115 | 0.130185278 | 0.090849 |
| GO_BP_Profile11 | GO:0015874 | norepinephrine transport | 1月25日 | 20/17913 | 0.027560115 | 0.130185278 | 0.090849 |
| GO_BP_Profile11 | GO:0032516 | positive regulation of phosphoprotein phosphatase activity | 1月25日 | 20/17913 | 0.027560115 | 0.130185278 | 0.090849 |
| GO_BP_Profile11 | GO:0035929 | steroid hormone secretion | 1月25日 | 20/17913 | 0.027560115 | 0.130185278 | 0.090849 |
| GO_BP_Profile11 | GO:0050866 | negative regulation of cell activation | 2月25日 | 187/17913 | 0.027783021 | 0.130262517 | 0.090903 |
| GO_BP_Profile11 | GO:0001956 | positive regulation of neurotransmitter secretion | 1月25日 | 21/17913 | 0.028918802 | 0.130262517 | 0.090903 |
| GO_BP_Profile11 | GO:0042749 | regulation of circadian sleep/wake cycle | 1月25日 | 21/17913 | 0.028918802 | 0.130262517 | 0.090903 |
| GO_BP_Profile11 | GO:0050802 | circadian sleep/wake cycle, sleep | 1月25日 | 21/17913 | 0.028918802 | 0.130262517 | 0.090903 |
| GO_BP_Profile11 | GO:0071498 | cellular response to fluid shear stress | 1月25日 | 21/17913 | 0.028918802 | 0.130262517 | 0.090903 |
| GO_BP_Profile11 | GO:0097709 | connective tissue replacement | 1月25日 | 21/17913 | 0.028918802 | 0.130262517 | 0.090903 |
| GO_BP_Profile11 | GO:0043312 | neutrophil degranulation | 3月25日 | 485/17913 | 0.02916371 | 0.130262517 | 0.090903 |
| GO_BP_Profile11 | GO:0002283 | neutrophil activation involved in immune response | 3月25日 | 488/17913 | 0.029628233 | 0.130262517 | 0.090903 |
| GO_BP_Profile11 | GO:0032799 | low-density lipoprotein receptor particle metabolic process | 1月25日 | 22/17913 | 0.030275667 | 0.130262517 | 0.090903 |
| GO_BP_Profile11 | GO:0035809 | regulation of urine volume | 1月25日 | 22/17913 | 0.030275667 | 0.130262517 | 0.090903 |
| GO_BP_Profile11 | GO:0035813 | regulation of renal sodium excretion | 1月25日 | 22/17913 | 0.030275667 | 0.130262517 | 0.090903 |
| GO_BP_Profile11 | GO:0042094 | interleukin-2 biosynthetic process | 1月25日 | 22/17913 | 0.030275667 | 0.130262517 | 0.090903 |
| GO_BP_Profile11 | GO:0045117 | azole transport | 1月25日 | 22/17913 | 0.030275667 | 0.130262517 | 0.090903 |
| GO_BP_Profile11 | GO:1900273 | positive regulation of long-term synaptic potentiation | 1月25日 | 22/17913 | 0.030275667 | 0.130262517 | 0.090903 |
| GO_BP_Profile11 | GO:0043281 | regulation of cysteine-type endopeptidase activity involved in apoptotic process | 2月25日 | 196/17913 | 0.030297828 | 0.130262517 | 0.090903 |
| GO_BP_Profile11 | GO:0002446 | neutrophil mediated immunity | 3月25日 | 499/17913 | 0.031365515 | 0.132039833 | 0.092143 |
| GO_BP_Profile11 | GO:0042119 | neutrophil activation | 3月25日 | 499/17913 | 0.031365515 | 0.132039833 | 0.092143 |
| GO_BP_Profile11 | GO:0032098 | regulation of appetite | 1月25日 | 23/17913 | 0.031630712 | 0.132039833 | 0.092143 |
| GO_BP_Profile11 | GO:0035588 | G protein-coupled purinergic receptor signaling pathway | 1月25日 | 23/17913 | 0.031630712 | 0.132039833 | 0.092143 |
| GO_BP_Profile11 | GO:0051968 | positive regulation of synaptic transmission, glutamatergic | 1月25日 | 23/17913 | 0.031630712 | 0.132039833 | 0.092143 |
| GO_BP_Profile11 | GO:0045766 | positive regulation of angiogenesis | 2月25日 | 203/17913 | 0.03231468 | 0.132304293 | 0.092328 |
| GO_BP_Profile11 | GO:0010640 | regulation of platelet-derived growth factor receptor signaling pathway | 1月25日 | 24/17913 | 0.032983939 | 0.132304293 | 0.092328 |
| GO_BP_Profile11 | GO:0022410 | circadian sleep/wake cycle process | 1月25日 | 24/17913 | 0.032983939 | 0.132304293 | 0.092328 |
| GO_BP_Profile11 | GO:0032800 | receptor biosynthetic process | 1月25日 | 24/17913 | 0.032983939 | 0.132304293 | 0.092328 |
| GO_BP_Profile11 | GO:0035812 | renal sodium excretion | 1月25日 | 24/17913 | 0.032983939 | 0.132304293 | 0.092328 |
| GO_BP_Profile11 | GO:0048143 | astrocyte activation | 1月25日 | 24/17913 | 0.032983939 | 0.132304293 | 0.092328 |
| GO_BP_Profile11 | GO:0051560 | mitochondrial calcium ion homeostasis | 1月25日 | 24/17913 | 0.032983939 | 0.132304293 | 0.092328 |
| GO_BP_Profile11 | GO:1901215 | negative regulation of neuron death | 2月25日 | 207/17913 | 0.033490604 | 0.133445431 | 0.093124 |
| GO_BP_Profile11 | GO:0001963 | synaptic transmission, dopaminergic | 1月25日 | 25/17913 | 0.034335351 | 0.133445431 | 0.093124 |
| GO_BP_Profile11 | GO:0006851 | mitochondrial calcium ion transmembrane transport | 1月25日 | 25/17913 | 0.034335351 | 0.133445431 | 0.093124 |
| GO_BP_Profile11 | GO:0034110 | regulation of homotypic cell-cell adhesion | 1月25日 | 25/17913 | 0.034335351 | 0.133445431 | 0.093124 |
| GO_BP_Profile11 | GO:0044062 | regulation of excretion | 1月25日 | 25/17913 | 0.034335351 | 0.133445431 | 0.093124 |
| GO_BP_Profile11 | GO:0043523 | regulation of neuron apoptotic process | 2月25日 | 210/17913 | 0.034383572 | 0.133445431 | 0.093124 |
| GO_BP_Profile11 | GO:0010001 | glial cell differentiation | 2月25日 | 212/17913 | 0.034984086 | 0.133446841 | 0.093125 |
| GO_BP_Profile11 | GO:0097191 | extrinsic apoptotic signaling pathway | 2月25日 | 213/17913 | 0.035285894 | 0.133446841 | 0.093125 |
| GO_BP_Profile11 | GO:0007160 | cell-matrix adhesion | 2月25日 | 214/17913 | 0.035588731 | 0.133446841 | 0.093125 |
| GO_BP_Profile11 | GO:0007205 | protein kinase C-activating G protein-coupled receptor signaling pathway | 1月25日 | 26/17913 | 0.035684949 | 0.133446841 | 0.093125 |
| GO_BP_Profile11 | GO:0007263 | nitric oxide mediated signal transduction | 1月25日 | 26/17913 | 0.035684949 | 0.133446841 | 0.093125 |
| GO_BP_Profile11 | GO:0050919 | negative chemotaxis | 1月25日 | 26/17913 | 0.035684949 | 0.133446841 | 0.093125 |
| GO_BP_Profile11 | GO:0061099 | negative regulation of protein tyrosine kinase activity | 1月25日 | 26/17913 | 0.035684949 | 0.133446841 | 0.093125 |
| GO_BP_Profile11 | GO:0042745 | circadian sleep/wake cycle | 1月25日 | 27/17913 | 0.037032737 | 0.13566074 | 0.09467 |
| GO_BP_Profile11 | GO:0048520 | positive regulation of behavior | 1月25日 | 27/17913 | 0.037032737 | 0.13566074 | 0.09467 |
| GO_BP_Profile11 | GO:2000171 | negative regulation of dendrite development | 1月25日 | 27/17913 | 0.037032737 | 0.13566074 | 0.09467 |
| GO_BP_Profile11 | GO:2001025 | positive regulation of response to drug | 1月25日 | 27/17913 | 0.037032737 | 0.13566074 | 0.09467 |
| GO_BP_Profile11 | GO:0002526 | acute inflammatory response | 2月25日 | 220/17913 | 0.037427198 | 0.136409787 | 0.095193 |
| GO_BP_Profile11 | GO:0006469 | negative regulation of protein kinase activity | 2月25日 | 222/17913 | 0.038048112 | 0.136415436 | 0.095197 |
| GO_BP_Profile11 | GO:2000116 | regulation of cysteine-type endopeptidase activity | 2月25日 | 222/17913 | 0.038048112 | 0.136415436 | 0.095197 |
| GO_BP_Profile11 | GO:2001234 | negative regulation of apoptotic signaling pathway | 2月25日 | 223/17913 | 0.038360072 | 0.136415436 | 0.095197 |
| GO_BP_Profile11 | GO:0001964 | startle response | 1月25日 | 28/17913 | 0.038378716 | 0.136415436 | 0.095197 |
| GO_BP_Profile11 | GO:0033688 | regulation of osteoblast proliferation | 1月25日 | 28/17913 | 0.038378716 | 0.136415436 | 0.095197 |
| GO_BP_Profile11 | GO:0010575 | positive regulation of vascular endothelial growth factor production | 1月25日 | 29/17913 | 0.039722889 | 0.140497705 | 0.098046 |
| GO_BP_Profile11 | GO:0050663 | cytokine secretion | 2月25日 | 230/17913 | 0.040571556 | 0.142120444 | 0.099178 |
| GO_BP_Profile11 | GO:0010543 | regulation of platelet activation | 1月25日 | 30/17913 | 0.041065258 | 0.142120444 | 0.099178 |
| GO_BP_Profile11 | GO:0010743 | regulation of macrophage derived foam cell differentiation | 1月25日 | 30/17913 | 0.041065258 | 0.142120444 | 0.099178 |
| GO_BP_Profile11 | GO:0032743 | positive regulation of interleukin-2 production | 1月25日 | 30/17913 | 0.041065258 | 0.142120444 | 0.099178 |
| GO_BP_Profile11 | GO:1904018 | positive regulation of vasculature development | 2月25日 | 232/17913 | 0.041212238 | 0.142120444 | 0.099178 |
| GO_BP_Profile11 | GO:0015872 | dopamine transport | 1月25日 | 31/17913 | 0.042405825 | 0.142120444 | 0.099178 |
| GO_BP_Profile11 | GO:0030431 | sleep | 1月25日 | 31/17913 | 0.042405825 | 0.142120444 | 0.099178 |
| GO_BP_Profile11 | GO:0033687 | osteoblast proliferation | 1月25日 | 31/17913 | 0.042405825 | 0.142120444 | 0.099178 |
| GO_BP_Profile11 | GO:0035587 | purinergic receptor signaling pathway | 1月25日 | 31/17913 | 0.042405825 | 0.142120444 | 0.099178 |
| GO_BP_Profile11 | GO:0044068 | modulation by symbiont of host cellular process | 1月25日 | 31/17913 | 0.042405825 | 0.142120444 | 0.099178 |
| GO_BP_Profile11 | GO:0051955 | regulation of amino acid transport | 1月25日 | 31/17913 | 0.042405825 | 0.142120444 | 0.099178 |
| GO_BP_Profile11 | GO:0051402 | neuron apoptotic process | 2月25日 | 238/17913 | 0.04315746 | 0.142120444 | 0.099178 |
| GO_BP_Profile11 | GO:0001975 | response to amphetamine | 1月25日 | 32/17913 | 0.043744594 | 0.142120444 | 0.099178 |
| GO_BP_Profile11 | GO:0030851 | granulocyte differentiation | 1月25日 | 32/17913 | 0.043744594 | 0.142120444 | 0.099178 |
| GO_BP_Profile11 | GO:0032228 | regulation of synaptic transmission, GABAergic | 1月25日 | 32/17913 | 0.043744594 | 0.142120444 | 0.099178 |
| GO_BP_Profile11 | GO:0046688 | response to copper ion | 1月25日 | 32/17913 | 0.043744594 | 0.142120444 | 0.099178 |
| GO_BP_Profile11 | GO:1901099 | negative regulation of signal transduction in absence of ligand | 1月25日 | 32/17913 | 0.043744594 | 0.142120444 | 0.099178 |
| GO_BP_Profile11 | GO:2001240 | negative regulation of extrinsic apoptotic signaling pathway in absence of ligand | 1月25日 | 32/17913 | 0.043744594 | 0.142120444 | 0.099178 |
| GO_BP_Profile11 | GO:0006836 | neurotransmitter transport | 2月25日 | 241/17913 | 0.044142954 | 0.142128436 | 0.099184 |
| GO_BP_Profile11 | GO:0051896 | regulation of protein kinase B signaling | 2月25日 | 241/17913 | 0.044142954 | 0.142128436 | 0.099184 |
| GO_BP_Profile11 | GO:0010922 | positive regulation of phosphatase activity | 1月25日 | 33/17913 | 0.045081565 | 0.142141729 | 0.099193 |
| GO_BP_Profile11 | GO:0032892 | positive regulation of organic acid transport | 1月25日 | 33/17913 | 0.045081565 | 0.142141729 | 0.099193 |
| GO_BP_Profile11 | GO:0042755 | eating behavior | 1月25日 | 33/17913 | 0.045081565 | 0.142141729 | 0.099193 |
| GO_BP_Profile11 | GO:0051954 | positive regulation of amine transport | 1月25日 | 33/17913 | 0.045081565 | 0.142141729 | 0.099193 |
| GO_BP_Profile11 | GO:0033673 | negative regulation of kinase activity | 2月25日 | 244/17913 | 0.045136928 | 0.142141729 | 0.099193 |
| GO_BP_Profile11 | GO:0050730 | regulation of peptidyl-tyrosine phosphorylation | 2月25日 | 245/17913 | 0.045470123 | 0.142565712 | 0.099489 |
| GO_BP_Profile11 | GO:0021537 | telencephalon development | 2月25日 | 246/17913 | 0.045804247 | 0.142988911 | 0.099784 |
| GO_BP_Profile11 | GO:0010574 | regulation of vascular endothelial growth factor production | 1月25日 | 34/17913 | 0.046416742 | 0.143651813 | 0.100247 |
| GO_BP_Profile11 | GO:2000249 | regulation of actin cytoskeleton reorganization | 1月25日 | 34/17913 | 0.046416742 | 0.143651813 | 0.100247 |
| GO_BP_Profile11 | GO:1901998 | toxin transport | 1月25日 | 35/17913 | 0.047750126 | 0.145961645 | 0.101859 |
| GO_BP_Profile11 | GO:0046883 | regulation of hormone secretion | 2月25日 | 254/17913 | 0.048510366 | 0.145961645 | 0.101859 |
| GO_BP_Profile11 | GO:0010573 | vascular endothelial growth factor production | 1月25日 | 36/17913 | 0.049081721 | 0.145961645 | 0.101859 |
| GO_BP_Profile11 | GO:0010742 | macrophage derived foam cell differentiation | 1月25日 | 36/17913 | 0.049081721 | 0.145961645 | 0.101859 |
| GO_BP_Profile11 | GO:0043114 | regulation of vascular permeability | 1月25日 | 36/17913 | 0.049081721 | 0.145961645 | 0.101859 |
| GO_BP_Profile11 | GO:0046636 | negative regulation of alpha-beta T cell activation | 1月25日 | 36/17913 | 0.049081721 | 0.145961645 | 0.101859 |
| GO_BP_Profile11 | GO:0071634 | regulation of transforming growth factor beta production | 1月25日 | 36/17913 | 0.049081721 | 0.145961645 | 0.101859 |
| GO_BP_Profile11 | GO:0090077 | foam cell differentiation | 1月25日 | 36/17913 | 0.049081721 | 0.145961645 | 0.101859 |
| GO_BP_Profile11 | GO:0098801 | regulation of renal system process | 1月25日 | 36/17913 | 0.049081721 | 0.145961645 | 0.101859 |
| GO_BP_Profile11 | GO:0050714 | positive regulation of protein secretion | 2月25日 | 256/17913 | 0.049195986 | 0.145961645 | 0.101859 |
| GO_BP_Profile12 | GO:1900426 | positive regulation of defense response to bacterium | 2/59 | 10/17913 | 0.000471853 | 0.193237164 | 0.165296 |
| GO_BP_Profile12 | GO:0002281 | macrophage activation involved in immune response | 2/59 | 12/17913 | 0.000689124 | 0.193237164 | 0.165296 |
| GO_BP_Profile12 | GO:0010759 | positive regulation of macrophage chemotaxis | 2/59 | 15/17913 | 0.001089387 | 0.193237164 | 0.165296 |
| GO_BP_Profile12 | GO:0070885 | negative regulation of calcineurin-NFAT signaling cascade | 2/59 | 17/17913 | 0.001405053 | 0.193237164 | 0.165296 |
| GO_BP_Profile12 | GO:0106057 | negative regulation of calcineurin-mediated signaling | 2/59 | 17/17913 | 0.001405053 | 0.193237164 | 0.165296 |
| GO_BP_Profile12 | GO:1900424 | regulation of defense response to bacterium | 2/59 | 20/17913 | 0.001950519 | 0.193237164 | 0.165296 |
| GO_BP_Profile12 | GO:1905523 | positive regulation of macrophage migration | 2/59 | 20/17913 | 0.001950519 | 0.193237164 | 0.165296 |
| GO_BP_Profile12 | GO:0016236 | macroautophagy | 5/59 | 272/17913 | 0.001995643 | 0.193237164 | 0.165296 |
| GO_BP_Profile12 | GO:0010758 | regulation of macrophage chemotaxis | 2/59 | 23/17913 | 0.002580844 | 0.193237164 | 0.165296 |
| GO_BP_Profile12 | GO:0021919 | BMP signaling pathway involved in spinal cord dorsal/ventral patterning | 1/59 | 1/17913 | 0.003293697 | 0.193237164 | 0.165296 |
| GO_BP_Profile12 | GO:0030209 | dermatan sulfate catabolic process | 1/59 | 1/17913 | 0.003293697 | 0.193237164 | 0.165296 |
| GO_BP_Profile12 | GO:0030211 | heparin catabolic process | 1/59 | 1/17913 | 0.003293697 | 0.193237164 | 0.165296 |
| GO_BP_Profile12 | GO:0032712 | negative regulation of interleukin-3 production | 1/59 | 1/17913 | 0.003293697 | 0.193237164 | 0.165296 |
| GO_BP_Profile12 | GO:0060611 | mammary gland fat development | 1/59 | 1/17913 | 0.003293697 | 0.193237164 | 0.165296 |
| GO_BP_Profile12 | GO:0070060 | 'de novo' actin filament nucleation | 1/59 | 1/17913 | 0.003293697 | 0.193237164 | 0.165296 |
| GO_BP_Profile12 | GO:0071656 | negative regulation of granulocyte colony-stimulating factor production | 1/59 | 1/17913 | 0.003293697 | 0.193237164 | 0.165296 |
| GO_BP_Profile12 | GO:0098829 | intestinal folate absorption | 1/59 | 1/17913 | 0.003293697 | 0.193237164 | 0.165296 |
| GO_BP_Profile12 | GO:1901257 | negative regulation of macrophage colony-stimulating factor production | 1/59 | 1/17913 | 0.003293697 | 0.193237164 | 0.165296 |
| GO_BP_Profile12 | GO:1902228 | positive regulation of macrophage colony-stimulating factor signaling pathway | 1/59 | 1/17913 | 0.003293697 | 0.193237164 | 0.165296 |
| GO_BP_Profile12 | GO:1903334 | positive regulation of protein folding | 1/59 | 1/17913 | 0.003293697 | 0.193237164 | 0.165296 |
| GO_BP_Profile12 | GO:1904863 | regulation of beta-catenin-TCF complex assembly | 1/59 | 1/17913 | 0.003293697 | 0.193237164 | 0.165296 |
| GO_BP_Profile12 | GO:1904864 | negative regulation of beta-catenin-TCF complex assembly | 1/59 | 1/17913 | 0.003293697 | 0.193237164 | 0.165296 |
| GO_BP_Profile12 | GO:1905673 | positive regulation of lysosome organization | 1/59 | 1/17913 | 0.003293697 | 0.193237164 | 0.165296 |
| GO_BP_Profile12 | GO:0032103 | positive regulation of response to external stimulus | 5/59 | 315/17913 | 0.003753516 | 0.193237164 | 0.165296 |
| GO_BP_Profile12 | GO:0050848 | regulation of calcium-mediated signaling | 3/59 | 98/17913 | 0.004135245 | 0.193237164 | 0.165296 |
| GO_BP_Profile12 | GO:0043547 | positive regulation of GTPase activity | 5/59 | 339/17913 | 0.005115885 | 0.193237164 | 0.165296 |
| GO_BP_Profile12 | GO:0045777 | positive regulation of blood pressure | 2/59 | 34/17913 | 0.005591362 | 0.193237164 | 0.165296 |
| GO_BP_Profile12 | GO:0050849 | negative regulation of calcium-mediated signaling | 2/59 | 34/17913 | 0.005591362 | 0.193237164 | 0.165296 |
| GO_BP_Profile12 | GO:0048246 | macrophage chemotaxis | 2/59 | 35/17913 | 0.005917742 | 0.193237164 | 0.165296 |
| GO_BP_Profile12 | GO:0070884 | regulation of calcineurin-NFAT signaling cascade | 2/59 | 35/17913 | 0.005917742 | 0.193237164 | 0.165296 |
| GO_BP_Profile12 | GO:0106056 | regulation of calcineurin-mediated signaling | 2/59 | 35/17913 | 0.005917742 | 0.193237164 | 0.165296 |
| GO_BP_Profile12 | GO:0050892 | intestinal absorption | 2/59 | 36/17913 | 0.006252651 | 0.193237164 | 0.165296 |
| GO_BP_Profile12 | GO:1905521 | regulation of macrophage migration | 2/59 | 36/17913 | 0.006252651 | 0.193237164 | 0.165296 |
| GO_BP_Profile12 | GO:0002835 | negative regulation of response to tumor cell | 1/59 | 2/17913 | 0.006576729 | 0.193237164 | 0.165296 |
| GO_BP_Profile12 | GO:0002838 | negative regulation of immune response to tumor cell | 1/59 | 2/17913 | 0.006576729 | 0.193237164 | 0.165296 |
| GO_BP_Profile12 | GO:0002856 | negative regulation of natural killer cell mediated immune response to tumor cell | 1/59 | 2/17913 | 0.006576729 | 0.193237164 | 0.165296 |
| GO_BP_Profile12 | GO:0002859 | negative regulation of natural killer cell mediated cytotoxicity directed against tumor cell target | 1/59 | 2/17913 | 0.006576729 | 0.193237164 | 0.165296 |
| GO_BP_Profile12 | GO:0017186 | peptidyl-pyroglutamic acid biosynthetic process, using glutaminyl-peptide cyclotransferase | 1/59 | 2/17913 | 0.006576729 | 0.193237164 | 0.165296 |
| GO_BP_Profile12 | GO:0032672 | regulation of interleukin-3 production | 1/59 | 2/17913 | 0.006576729 | 0.193237164 | 0.165296 |
| GO_BP_Profile12 | GO:0042488 | positive regulation of odontogenesis of dentin-containing tooth | 1/59 | 2/17913 | 0.006576729 | 0.193237164 | 0.165296 |
| GO_BP_Profile12 | GO:0051958 | methotrexate transport | 1/59 | 2/17913 | 0.006576729 | 0.193237164 | 0.165296 |
| GO_BP_Profile12 | GO:0097274 | urea homeostasis | 1/59 | 2/17913 | 0.006576729 | 0.193237164 | 0.165296 |
| GO_BP_Profile12 | GO:1900220 | semaphorin-plexin signaling pathway involved in bone trabecula morphogenesis | 1/59 | 2/17913 | 0.006576729 | 0.193237164 | 0.165296 |
| GO_BP_Profile12 | GO:2000521 | negative regulation of immunological synapse formation | 1/59 | 2/17913 | 0.006576729 | 0.193237164 | 0.165296 |
| GO_BP_Profile12 | GO:0032365 | intracellular lipid transport | 2/59 | 37/17913 | 0.006596029 | 0.193237164 | 0.165296 |
| GO_BP_Profile12 | GO:0055067 | monovalent inorganic cation homeostasis | 3/59 | 122/17913 | 0.007592686 | 0.193237164 | 0.165296 |
| GO_BP_Profile12 | GO:0033173 | calcineurin-NFAT signaling cascade | 2/59 | 41/17913 | 0.008053103 | 0.193237164 | 0.165296 |
| GO_BP_Profile12 | GO:0060612 | adipose tissue development | 2/59 | 41/17913 | 0.008053103 | 0.193237164 | 0.165296 |
| GO_BP_Profile12 | GO:0009311 | oligosaccharide metabolic process | 2/59 | 42/17913 | 0.008437978 | 0.193237164 | 0.165296 |
| GO_BP_Profile12 | GO:0045785 | positive regulation of cell adhesion | 5/59 | 388/17913 | 0.008929333 | 0.193237164 | 0.165296 |
| GO_BP_Profile12 | GO:0071622 | regulation of granulocyte chemotaxis | 2/59 | 44/17913 | 0.009232059 | 0.193237164 | 0.165296 |
| GO_BP_Profile12 | GO:0097720 | calcineurin-mediated signaling | 2/59 | 44/17913 | 0.009232059 | 0.193237164 | 0.165296 |
| GO_BP_Profile12 | GO:0002833 | positive regulation of response to biotic stimulus | 2/59 | 45/17913 | 0.009641155 | 0.193237164 | 0.165296 |
| GO_BP_Profile12 | GO:0071675 | regulation of mononuclear cell migration | 2/59 | 45/17913 | 0.009641155 | 0.193237164 | 0.165296 |
| GO_BP_Profile12 | GO:0002282 | microglial cell activation involved in immune response | 1/59 | 3/17913 | 0.00984913 | 0.193237164 | 0.165296 |
| GO_BP_Profile12 | GO:0002519 | natural killer cell tolerance induction | 1/59 | 3/17913 | 0.00984913 | 0.193237164 | 0.165296 |
| GO_BP_Profile12 | GO:0002652 | regulation of tolerance induction dependent upon immune response | 1/59 | 3/17913 | 0.00984913 | 0.193237164 | 0.165296 |
| GO_BP_Profile12 | GO:0006982 | response to lipid hydroperoxide | 1/59 | 3/17913 | 0.00984913 | 0.193237164 | 0.165296 |
| GO_BP_Profile12 | GO:0007181 | transforming growth factor beta receptor complex assembly | 1/59 | 3/17913 | 0.00984913 | 0.193237164 | 0.165296 |
| GO_BP_Profile12 | GO:0018125 | peptidyl-cysteine methylation | 1/59 | 3/17913 | 0.00984913 | 0.193237164 | 0.165296 |
| GO_BP_Profile12 | GO:0021914 | negative regulation of smoothened signaling pathway involved in ventral spinal cord patterning | 1/59 | 3/17913 | 0.00984913 | 0.193237164 | 0.165296 |
| GO_BP_Profile12 | GO:0030886 | negative regulation of myeloid dendritic cell activation | 1/59 | 3/17913 | 0.00984913 | 0.193237164 | 0.165296 |
| GO_BP_Profile12 | GO:0032632 | interleukin-3 production | 1/59 | 3/17913 | 0.00984913 | 0.193237164 | 0.165296 |
| GO_BP_Profile12 | GO:0048499 | synaptic vesicle membrane organization | 1/59 | 3/17913 | 0.00984913 | 0.193237164 | 0.165296 |
| GO_BP_Profile12 | GO:0048619 | embryonic hindgut morphogenesis | 1/59 | 3/17913 | 0.00984913 | 0.193237164 | 0.165296 |
| GO_BP_Profile12 | GO:0060763 | mammary duct terminal end bud growth | 1/59 | 3/17913 | 0.00984913 | 0.193237164 | 0.165296 |
| GO_BP_Profile12 | GO:1902226 | regulation of macrophage colony-stimulating factor signaling pathway | 1/59 | 3/17913 | 0.00984913 | 0.193237164 | 0.165296 |
| GO_BP_Profile12 | GO:1903971 | positive regulation of response to macrophage colony-stimulating factor | 1/59 | 3/17913 | 0.00984913 | 0.193237164 | 0.165296 |
| GO_BP_Profile12 | GO:1903974 | positive regulation of cellular response to macrophage colony-stimulating factor stimulus | 1/59 | 3/17913 | 0.00984913 | 0.193237164 | 0.165296 |
| GO_BP_Profile12 | GO:2000520 | regulation of immunological synapse formation | 1/59 | 3/17913 | 0.00984913 | 0.193237164 | 0.165296 |
| GO_BP_Profile12 | GO:2001189 | negative regulation of T cell activation via T cell receptor contact with antigen bound to MHC molecule on antigen presenting cell | 1/59 | 3/17913 | 0.00984913 | 0.193237164 | 0.165296 |
| GO_BP_Profile12 | GO:0044273 | sulfur compound catabolic process | 2/59 | 48/17913 | 0.010916001 | 0.20324332 | 0.173855 |
| GO_BP_Profile12 | GO:0043087 | regulation of GTPase activity | 5/59 | 410/17913 | 0.01115515 | 0.20324332 | 0.173855 |
| GO_BP_Profile12 | GO:1905517 | macrophage migration | 2/59 | 50/17913 | 0.011804982 | 0.20324332 | 0.173855 |
| GO_BP_Profile12 | GO:0007162 | negative regulation of cell adhesion | 4/59 | 271/17913 | 0.012161131 | 0.20324332 | 0.173855 |
| GO_BP_Profile12 | GO:0048016 | inositol phosphate-mediated signaling | 2/59 | 52/17913 | 0.012724725 | 0.20324332 | 0.173855 |
| GO_BP_Profile12 | GO:0002265 | astrocyte activation involved in immune response | 1/59 | 4/17913 | 0.013110934 | 0.20324332 | 0.173855 |
| GO_BP_Profile12 | GO:0002528 | regulation of vascular permeability involved in acute inflammatory response | 1/59 | 4/17913 | 0.013110934 | 0.20324332 | 0.173855 |
| GO_BP_Profile12 | GO:0018199 | peptidyl-glutamine modification | 1/59 | 4/17913 | 0.013110934 | 0.20324332 | 0.173855 |
| GO_BP_Profile12 | GO:0035814 | negative regulation of renal sodium excretion | 1/59 | 4/17913 | 0.013110934 | 0.20324332 | 0.173855 |
| GO_BP_Profile12 | GO:0035934 | corticosterone secretion | 1/59 | 4/17913 | 0.013110934 | 0.20324332 | 0.173855 |
| GO_BP_Profile12 | GO:0043503 | skeletal muscle fiber adaptation | 1/59 | 4/17913 | 0.013110934 | 0.20324332 | 0.173855 |
| GO_BP_Profile12 | GO:0071655 | regulation of granulocyte colony-stimulating factor production | 1/59 | 4/17913 | 0.013110934 | 0.20324332 | 0.173855 |
| GO_BP_Profile12 | GO:0106016 | positive regulation of inflammatory response to wounding | 1/59 | 4/17913 | 0.013110934 | 0.20324332 | 0.173855 |
| GO_BP_Profile12 | GO:1900069 | regulation of cellular hyperosmotic salinity response | 1/59 | 4/17913 | 0.013110934 | 0.20324332 | 0.173855 |
| GO_BP_Profile12 | GO:1901000 | regulation of response to salt stress | 1/59 | 4/17913 | 0.013110934 | 0.20324332 | 0.173855 |
| GO_BP_Profile12 | GO:1905150 | regulation of voltage-gated sodium channel activity | 1/59 | 4/17913 | 0.013110934 | 0.20324332 | 0.173855 |
| GO_BP_Profile12 | GO:0042130 | negative regulation of T cell proliferation | 2/59 | 53/17913 | 0.013195998 | 0.20324332 | 0.173855 |
| GO_BP_Profile12 | GO:0014888 | striated muscle adaptation | 2/59 | 56/17913 | 0.014654786 | 0.20324332 | 0.173855 |
| GO_BP_Profile12 | GO:0045824 | negative regulation of innate immune response | 2/59 | 57/17913 | 0.015155862 | 0.20324332 | 0.173855 |
| GO_BP_Profile12 | GO:0002461 | tolerance induction dependent upon immune response | 1/59 | 5/17913 | 0.016362174 | 0.20324332 | 0.173855 |
| GO_BP_Profile12 | GO:0032815 | negative regulation of natural killer cell activation | 1/59 | 5/17913 | 0.016362174 | 0.20324332 | 0.173855 |
| GO_BP_Profile12 | GO:0033227 | dsRNA transport | 1/59 | 5/17913 | 0.016362174 | 0.20324332 | 0.173855 |
| GO_BP_Profile12 | GO:0035694 | mitochondrial protein catabolic process | 1/59 | 5/17913 | 0.016362174 | 0.20324332 | 0.173855 |
| GO_BP_Profile12 | GO:0036301 | macrophage colony-stimulating factor production | 1/59 | 5/17913 | 0.016362174 | 0.20324332 | 0.173855 |
| GO_BP_Profile12 | GO:0060040 | retinal bipolar neuron differentiation | 1/59 | 5/17913 | 0.016362174 | 0.20324332 | 0.173855 |
| GO_BP_Profile12 | GO:0060266 | negative regulation of respiratory burst involved in inflammatory response | 1/59 | 5/17913 | 0.016362174 | 0.20324332 | 0.173855 |
| GO_BP_Profile12 | GO:0061304 | retinal blood vessel morphogenesis | 1/59 | 5/17913 | 0.016362174 | 0.20324332 | 0.173855 |
| GO_BP_Profile12 | GO:0062028 | regulation of stress granule assembly | 1/59 | 5/17913 | 0.016362174 | 0.20324332 | 0.173855 |
| GO_BP_Profile12 | GO:0071611 | granulocyte colony-stimulating factor production | 1/59 | 5/17913 | 0.016362174 | 0.20324332 | 0.173855 |
| GO_BP_Profile12 | GO:1901256 | regulation of macrophage colony-stimulating factor production | 1/59 | 5/17913 | 0.016362174 | 0.20324332 | 0.173855 |
| GO_BP_Profile12 | GO:1902564 | negative regulation of neutrophil activation | 1/59 | 5/17913 | 0.016362174 | 0.20324332 | 0.173855 |
| GO_BP_Profile12 | GO:1903969 | regulation of response to macrophage colony-stimulating factor | 1/59 | 5/17913 | 0.016362174 | 0.20324332 | 0.173855 |
| GO_BP_Profile12 | GO:1903972 | regulation of cellular response to macrophage colony-stimulating factor stimulus | 1/59 | 5/17913 | 0.016362174 | 0.20324332 | 0.173855 |
| GO_BP_Profile12 | GO:1904141 | positive regulation of microglial cell migration | 1/59 | 5/17913 | 0.016362174 | 0.20324332 | 0.173855 |
| GO_BP_Profile12 | GO:1904428 | negative regulation of tubulin deacetylation | 1/59 | 5/17913 | 0.016362174 | 0.20324332 | 0.173855 |
| GO_BP_Profile12 | GO:1904447 | folate import across plasma membrane | 1/59 | 5/17913 | 0.016362174 | 0.20324332 | 0.173855 |
| GO_BP_Profile12 | GO:1905671 | regulation of lysosome organization | 1/59 | 5/17913 | 0.016362174 | 0.20324332 | 0.173855 |
| GO_BP_Profile12 | GO:0002695 | negative regulation of leukocyte activation | 3/59 | 163/17913 | 0.016576407 | 0.20324332 | 0.173855 |
| GO_BP_Profile12 | GO:0006027 | glycosaminoglycan catabolic process | 2/59 | 61/17913 | 0.017232845 | 0.20324332 | 0.173855 |
| GO_BP_Profile12 | GO:0016241 | regulation of macroautophagy | 3/59 | 166/17913 | 0.017393967 | 0.20324332 | 0.173855 |
| GO_BP_Profile12 | GO:0006026 | aminoglycan catabolic process | 2/59 | 63/17913 | 0.018314223 | 0.20324332 | 0.173855 |
| GO_BP_Profile12 | GO:0071277 | cellular response to calcium ion | 2/59 | 63/17913 | 0.018314223 | 0.20324332 | 0.173855 |
| GO_BP_Profile12 | GO:0015909 | long-chain fatty acid transport | 2/59 | 64/17913 | 0.018865456 | 0.20324332 | 0.173855 |
| GO_BP_Profile12 | GO:0006914 | autophagy | 5/59 | 471/17913 | 0.019250198 | 0.20324332 | 0.173855 |
| GO_BP_Profile12 | GO:0061919 | process utilizing autophagic mechanism | 5/59 | 471/17913 | 0.019250198 | 0.20324332 | 0.173855 |
| GO_BP_Profile12 | GO:0032945 | negative regulation of mononuclear cell proliferation | 2/59 | 65/17913 | 0.019423649 | 0.20324332 | 0.173855 |
| GO_BP_Profile12 | GO:0050672 | negative regulation of lymphocyte proliferation | 2/59 | 65/17913 | 0.019423649 | 0.20324332 | 0.173855 |
| GO_BP_Profile12 | GO:0015886 | heme transport | 1/59 | 6/17913 | 0.019602884 | 0.20324332 | 0.173855 |
| GO_BP_Profile12 | GO:0030885 | regulation of myeloid dendritic cell activation | 1/59 | 6/17913 | 0.019602884 | 0.20324332 | 0.173855 |
| GO_BP_Profile12 | GO:0032687 | negative regulation of interferon-alpha production | 1/59 | 6/17913 | 0.019602884 | 0.20324332 | 0.173855 |
| GO_BP_Profile12 | GO:0034154 | toll-like receptor 7 signaling pathway | 1/59 | 6/17913 | 0.019602884 | 0.20324332 | 0.173855 |
| GO_BP_Profile12 | GO:0038063 | collagen-activated tyrosine kinase receptor signaling pathway | 1/59 | 6/17913 | 0.019602884 | 0.20324332 | 0.173855 |
| GO_BP_Profile12 | GO:0038145 | macrophage colony-stimulating factor signaling pathway | 1/59 | 6/17913 | 0.019602884 | 0.20324332 | 0.173855 |
| GO_BP_Profile12 | GO:0060264 | regulation of respiratory burst involved in inflammatory response | 1/59 | 6/17913 | 0.019602884 | 0.20324332 | 0.173855 |
| GO_BP_Profile12 | GO:0060268 | negative regulation of respiratory burst | 1/59 | 6/17913 | 0.019602884 | 0.20324332 | 0.173855 |
| GO_BP_Profile12 | GO:0061518 | microglial cell proliferation | 1/59 | 6/17913 | 0.019602884 | 0.20324332 | 0.173855 |
| GO_BP_Profile12 | GO:0070358 | actin polymerization-dependent cell motility | 1/59 | 6/17913 | 0.019602884 | 0.20324332 | 0.173855 |
| GO_BP_Profile12 | GO:0098838 | folate transmembrane transport | 1/59 | 6/17913 | 0.019602884 | 0.20324332 | 0.173855 |
| GO_BP_Profile12 | GO:0106014 | regulation of inflammatory response to wounding | 1/59 | 6/17913 | 0.019602884 | 0.20324332 | 0.173855 |
| GO_BP_Profile12 | GO:1900825 | regulation of membrane depolarization during cardiac muscle cell action potential | 1/59 | 6/17913 | 0.019602884 | 0.20324332 | 0.173855 |
| GO_BP_Profile12 | GO:1904304 | regulation of gastro-intestinal system smooth muscle contraction | 1/59 | 6/17913 | 0.019602884 | 0.20324332 | 0.173855 |
| GO_BP_Profile12 | GO:1904306 | positive regulation of gastro-intestinal system smooth muscle contraction | 1/59 | 6/17913 | 0.019602884 | 0.20324332 | 0.173855 |
| GO_BP_Profile12 | GO:2001188 | regulation of T cell activation via T cell receptor contact with antigen bound to MHC molecule on antigen presenting cell | 1/59 | 6/17913 | 0.019602884 | 0.20324332 | 0.173855 |
| GO_BP_Profile12 | GO:0016239 | positive regulation of macroautophagy | 2/59 | 66/17913 | 0.019988754 | 0.20324332 | 0.173855 |
| GO_BP_Profile12 | GO:0043030 | regulation of macrophage activation | 2/59 | 66/17913 | 0.019988754 | 0.20324332 | 0.173855 |
| GO_BP_Profile12 | GO:1990542 | mitochondrial transmembrane transport | 2/59 | 66/17913 | 0.019988754 | 0.20324332 | 0.173855 |
| GO_BP_Profile12 | GO:0071674 | mononuclear cell migration | 2/59 | 68/17913 | 0.021139498 | 0.21240414 | 0.181692 |
| GO_BP_Profile12 | GO:0043409 | negative regulation of MAPK cascade | 3/59 | 179/17913 | 0.02119467 | 0.21240414 | 0.181692 |
| GO_BP_Profile12 | GO:0002536 | respiratory burst involved in inflammatory response | 1/59 | 7/17913 | 0.022833097 | 0.212815189 | 0.182043 |
| GO_BP_Profile12 | GO:0002826 | negative regulation of T-helper 1 type immune response | 1/59 | 7/17913 | 0.022833097 | 0.212815189 | 0.182043 |
| GO_BP_Profile12 | GO:0007442 | hindgut morphogenesis | 1/59 | 7/17913 | 0.022833097 | 0.212815189 | 0.182043 |
| GO_BP_Profile12 | GO:0010269 | response to selenium ion | 1/59 | 7/17913 | 0.022833097 | 0.212815189 | 0.182043 |
| GO_BP_Profile12 | GO:0033504 | floor plate development | 1/59 | 7/17913 | 0.022833097 | 0.212815189 | 0.182043 |
| GO_BP_Profile12 | GO:0036089 | cleavage furrow formation | 1/59 | 7/17913 | 0.022833097 | 0.212815189 | 0.182043 |
| GO_BP_Profile12 | GO:0071475 | cellular hyperosmotic salinity response | 1/59 | 7/17913 | 0.022833097 | 0.212815189 | 0.182043 |
| GO_BP_Profile12 | GO:0098902 | regulation of membrane depolarization during action potential | 1/59 | 7/17913 | 0.022833097 | 0.212815189 | 0.182043 |
| GO_BP_Profile12 | GO:1904124 | microglial cell migration | 1/59 | 7/17913 | 0.022833097 | 0.212815189 | 0.182043 |
| GO_BP_Profile12 | GO:1904139 | regulation of microglial cell migration | 1/59 | 7/17913 | 0.022833097 | 0.212815189 | 0.182043 |
| GO_BP_Profile12 | GO:0070664 | negative regulation of leukocyte proliferation | 2/59 | 71/17913 | 0.022916208 | 0.212815189 | 0.182043 |
| GO_BP_Profile12 | GO:0050866 | negative regulation of cell activation | 3/59 | 187/17913 | 0.023742629 | 0.217315066 | 0.185892 |
| GO_BP_Profile12 | GO:0006869 | lipid transport | 4/59 | 332/17913 | 0.023745215 | 0.217315066 | 0.185892 |
| GO_BP_Profile12 | GO:0019722 | calcium-mediated signaling | 3/59 | 188/17913 | 0.02407234 | 0.217315066 | 0.185892 |
| GO_BP_Profile12 | GO:0030198 | extracellular matrix organization | 4/59 | 334/17913 | 0.024208554 | 0.217315066 | 0.185892 |
| GO_BP_Profile12 | GO:0006885 | regulation of pH | 2/59 | 76/17913 | 0.026009056 | 0.217315066 | 0.185892 |
| GO_BP_Profile12 | GO:0002420 | natural killer cell mediated cytotoxicity directed against tumor cell target | 1/59 | 8/17913 | 0.026052847 | 0.217315066 | 0.185892 |
| GO_BP_Profile12 | GO:0002858 | regulation of natural killer cell mediated cytotoxicity directed against tumor cell target | 1/59 | 8/17913 | 0.026052847 | 0.217315066 | 0.185892 |
| GO_BP_Profile12 | GO:0007042 | lysosomal lumen acidification | 1/59 | 8/17913 | 0.026052847 | 0.217315066 | 0.185892 |
| GO_BP_Profile12 | GO:0015884 | folic acid transport | 1/59 | 8/17913 | 0.026052847 | 0.217315066 | 0.185892 |
| GO_BP_Profile12 | GO:0038114 | interleukin-21-mediated signaling pathway | 1/59 | 8/17913 | 0.026052847 | 0.217315066 | 0.185892 |
| GO_BP_Profile12 | GO:0061517 | macrophage proliferation | 1/59 | 8/17913 | 0.026052847 | 0.217315066 | 0.185892 |
| GO_BP_Profile12 | GO:0061525 | hindgut development | 1/59 | 8/17913 | 0.026052847 | 0.217315066 | 0.185892 |
| GO_BP_Profile12 | GO:0098756 | response to interleukin-21 | 1/59 | 8/17913 | 0.026052847 | 0.217315066 | 0.185892 |
| GO_BP_Profile12 | GO:0098757 | cellular response to interleukin-21 | 1/59 | 8/17913 | 0.026052847 | 0.217315066 | 0.185892 |
| GO_BP_Profile12 | GO:1900425 | negative regulation of defense response to bacterium | 1/59 | 8/17913 | 0.026052847 | 0.217315066 | 0.185892 |
| GO_BP_Profile12 | GO:1903332 | regulation of protein folding | 1/59 | 8/17913 | 0.026052847 | 0.217315066 | 0.185892 |
| GO_BP_Profile12 | GO:1905247 | positive regulation of aspartic-type peptidase activity | 1/59 | 8/17913 | 0.026052847 | 0.217315066 | 0.185892 |
| GO_BP_Profile12 | GO:0032944 | regulation of mononuclear cell proliferation | 3/59 | 200/17913 | 0.028223145 | 0.225205522 | 0.192642 |
| GO_BP_Profile12 | GO:0002690 | positive regulation of leukocyte chemotaxis | 2/59 | 81/17913 | 0.029261295 | 0.225205522 | 0.192642 |
| GO_BP_Profile12 | GO:0002158 | osteoclast proliferation | 1/59 | 9/17913 | 0.029262168 | 0.225205522 | 0.192642 |
| GO_BP_Profile12 | GO:0002423 | natural killer cell mediated immune response to tumor cell | 1/59 | 9/17913 | 0.029262168 | 0.225205522 | 0.192642 |
| GO_BP_Profile12 | GO:0002855 | regulation of natural killer cell mediated immune response to tumor cell | 1/59 | 9/17913 | 0.029262168 | 0.225205522 | 0.192642 |
| GO_BP_Profile12 | GO:0005984 | disaccharide metabolic process | 1/59 | 9/17913 | 0.029262168 | 0.225205522 | 0.192642 |
| GO_BP_Profile12 | GO:0030202 | heparin metabolic process | 1/59 | 9/17913 | 0.029262168 | 0.225205522 | 0.192642 |
| GO_BP_Profile12 | GO:0035933 | glucocorticoid secretion | 1/59 | 9/17913 | 0.029262168 | 0.225205522 | 0.192642 |
| GO_BP_Profile12 | GO:0038065 | collagen-activated signaling pathway | 1/59 | 9/17913 | 0.029262168 | 0.225205522 | 0.192642 |
| GO_BP_Profile12 | GO:0038155 | interleukin-23-mediated signaling pathway | 1/59 | 9/17913 | 0.029262168 | 0.225205522 | 0.192642 |
| GO_BP_Profile12 | GO:0042117 | monocyte activation | 1/59 | 9/17913 | 0.029262168 | 0.225205522 | 0.192642 |
| GO_BP_Profile12 | GO:0042482 | positive regulation of odontogenesis | 1/59 | 9/17913 | 0.029262168 | 0.225205522 | 0.192642 |
| GO_BP_Profile12 | GO:0090043 | regulation of tubulin deacetylation | 1/59 | 9/17913 | 0.029262168 | 0.225205522 | 0.192642 |
| GO_BP_Profile12 | GO:1901678 | iron coordination entity transport | 1/59 | 9/17913 | 0.029262168 | 0.225205522 | 0.192642 |
| GO_BP_Profile12 | GO:0071621 | granulocyte chemotaxis | 2/59 | 84/17913 | 0.031286704 | 0.236745028 | 0.202513 |
| GO_BP_Profile12 | GO:0010876 | lipid localization | 4/59 | 366/17913 | 0.032376736 | 0.236745028 | 0.202513 |
| GO_BP_Profile12 | GO:0002291 | T cell activation via T cell receptor contact with antigen bound to MHC molecule on antigen presenting cell | 1/59 | 10/17913 | 0.032461091 | 0.236745028 | 0.202513 |
| GO_BP_Profile12 | GO:0036444 | calcium import into the mitochondrion | 1/59 | 10/17913 | 0.032461091 | 0.236745028 | 0.202513 |
| GO_BP_Profile12 | GO:0044539 | long-chain fatty acid import | 1/59 | 10/17913 | 0.032461091 | 0.236745028 | 0.202513 |
| GO_BP_Profile12 | GO:0045657 | positive regulation of monocyte differentiation | 1/59 | 10/17913 | 0.032461091 | 0.236745028 | 0.202513 |
| GO_BP_Profile12 | GO:0048680 | positive regulation of axon regeneration | 1/59 | 10/17913 | 0.032461091 | 0.236745028 | 0.202513 |
| GO_BP_Profile12 | GO:0061299 | retina vasculature morphogenesis in camera-type eye | 1/59 | 10/17913 | 0.032461091 | 0.236745028 | 0.202513 |
| GO_BP_Profile12 | GO:0090557 | establishment of endothelial intestinal barrier | 1/59 | 10/17913 | 0.032461091 | 0.236745028 | 0.202513 |
| GO_BP_Profile12 | GO:0106049 | regulation of cellular response to osmotic stress | 1/59 | 10/17913 | 0.032461091 | 0.236745028 | 0.202513 |
| GO_BP_Profile12 | GO:0070663 | regulation of leukocyte proliferation | 3/59 | 214/17913 | 0.033517469 | 0.242243731 | 0.207216 |
| GO_BP_Profile12 | GO:0032088 | negative regulation of NF-kappaB transcription factor activity | 2/59 | 89/17913 | 0.034781847 | 0.242243731 | 0.207216 |
| GO_BP_Profile12 | GO:0015908 | fatty acid transport | 2/59 | 90/17913 | 0.035498389 | 0.242243731 | 0.207216 |
| GO_BP_Profile12 | GO:0006853 | carnitine shuttle | 1/59 | 11/17913 | 0.035649652 | 0.242243731 | 0.207216 |
| GO_BP_Profile12 | GO:0014831 | gastro-intestinal system smooth muscle contraction | 1/59 | 11/17913 | 0.035649652 | 0.242243731 | 0.207216 |
| GO_BP_Profile12 | GO:0035404 | histone-serine phosphorylation | 1/59 | 11/17913 | 0.035649652 | 0.242243731 | 0.207216 |
| GO_BP_Profile12 | GO:0046007 | negative regulation of activated T cell proliferation | 1/59 | 11/17913 | 0.035649652 | 0.242243731 | 0.207216 |
| GO_BP_Profile12 | GO:0051561 | positive regulation of mitochondrial calcium ion concentration | 1/59 | 11/17913 | 0.035649652 | 0.242243731 | 0.207216 |
| GO_BP_Profile12 | GO:0070572 | positive regulation of neuron projection regeneration | 1/59 | 11/17913 | 0.035649652 | 0.242243731 | 0.207216 |
| GO_BP_Profile12 | GO:0070757 | interleukin-35-mediated signaling pathway | 1/59 | 11/17913 | 0.035649652 | 0.242243731 | 0.207216 |
| GO_BP_Profile12 | GO:0090042 | tubulin deacetylation | 1/59 | 11/17913 | 0.035649652 | 0.242243731 | 0.207216 |
| GO_BP_Profile12 | GO:1903977 | positive regulation of glial cell migration | 1/59 | 11/17913 | 0.035649652 | 0.242243731 | 0.207216 |
| GO_BP_Profile12 | GO:1903979 | negative regulation of microglial cell activation | 1/59 | 11/17913 | 0.035649652 | 0.242243731 | 0.207216 |
| GO_BP_Profile12 | GO:1905245 | regulation of aspartic-type peptidase activity | 1/59 | 11/17913 | 0.035649652 | 0.242243731 | 0.207216 |
| GO_BP_Profile12 | GO:0006839 | mitochondrial transport | 3/59 | 224/17913 | 0.037594571 | 0.246973694 | 0.211262 |
| GO_BP_Profile12 | GO:0007041 | lysosomal transport | 2/59 | 94/17913 | 0.038421454 | 0.246973694 | 0.211262 |
| GO_BP_Profile12 | GO:0022600 | digestive system process | 2/59 | 94/17913 | 0.038421454 | 0.246973694 | 0.211262 |
| GO_BP_Profile12 | GO:0043062 | extracellular structure organization | 4/59 | 387/17913 | 0.038521128 | 0.246973694 | 0.211262 |
| GO_BP_Profile12 | GO:0003084 | positive regulation of systemic arterial blood pressure | 1/59 | 12/17913 | 0.038827882 | 0.246973694 | 0.211262 |
| GO_BP_Profile12 | GO:0015867 | ATP transport | 1/59 | 12/17913 | 0.038827882 | 0.246973694 | 0.211262 |
| GO_BP_Profile12 | GO:0035751 | regulation of lysosomal lumen pH | 1/59 | 12/17913 | 0.038827882 | 0.246973694 | 0.211262 |
| GO_BP_Profile12 | GO:0036005 | response to macrophage colony-stimulating factor | 1/59 | 12/17913 | 0.038827882 | 0.246973694 | 0.211262 |
| GO_BP_Profile12 | GO:0036006 | cellular response to macrophage colony-stimulating factor stimulus | 1/59 | 12/17913 | 0.038827882 | 0.246973694 | 0.211262 |
| GO_BP_Profile12 | GO:0042340 | keratan sulfate catabolic process | 1/59 | 12/17913 | 0.038827882 | 0.246973694 | 0.211262 |
| GO_BP_Profile12 | GO:0042487 | regulation of odontogenesis of dentin-containing tooth | 1/59 | 12/17913 | 0.038827882 | 0.246973694 | 0.211262 |
| GO_BP_Profile12 | GO:0071472 | cellular response to salt stress | 1/59 | 12/17913 | 0.038827882 | 0.246973694 | 0.211262 |
| GO_BP_Profile12 | GO:1902563 | regulation of neutrophil activation | 1/59 | 12/17913 | 0.038827882 | 0.246973694 | 0.211262 |
| GO_BP_Profile12 | GO:1904862 | inhibitory synapse assembly | 1/59 | 12/17913 | 0.038827882 | 0.246973694 | 0.211262 |
| GO_BP_Profile12 | GO:0042116 | macrophage activation | 2/59 | 96/17913 | 0.039916512 | 0.251073685 | 0.21477 |
| GO_BP_Profile12 | GO:0050808 | synapse organization | 4/59 | 394/17913 | 0.040708841 | 0.251073685 | 0.21477 |
| GO_BP_Profile12 | GO:0002679 | respiratory burst involved in defense response | 1/59 | 13/17913 | 0.041995814 | 0.251073685 | 0.21477 |
| GO_BP_Profile12 | GO:0006703 | estrogen biosynthetic process | 1/59 | 13/17913 | 0.041995814 | 0.251073685 | 0.21477 |
| GO_BP_Profile12 | GO:0009312 | oligosaccharide biosynthetic process | 1/59 | 13/17913 | 0.041995814 | 0.251073685 | 0.21477 |
| GO_BP_Profile12 | GO:0010459 | negative regulation of heart rate | 1/59 | 13/17913 | 0.041995814 | 0.251073685 | 0.21477 |
| GO_BP_Profile12 | GO:0030205 | dermatan sulfate metabolic process | 1/59 | 13/17913 | 0.041995814 | 0.251073685 | 0.21477 |
| GO_BP_Profile12 | GO:0032096 | negative regulation of response to food | 1/59 | 13/17913 | 0.041995814 | 0.251073685 | 0.21477 |
| GO_BP_Profile12 | GO:0032099 | negative regulation of appetite | 1/59 | 13/17913 | 0.041995814 | 0.251073685 | 0.21477 |
| GO_BP_Profile12 | GO:0042538 | hyperosmotic salinity response | 1/59 | 13/17913 | 0.041995814 | 0.251073685 | 0.21477 |
| GO_BP_Profile12 | GO:0044090 | positive regulation of vacuole organization | 1/59 | 13/17913 | 0.041995814 | 0.251073685 | 0.21477 |
| GO_BP_Profile12 | GO:0047484 | regulation of response to osmotic stress | 1/59 | 13/17913 | 0.041995814 | 0.251073685 | 0.21477 |
| GO_BP_Profile12 | GO:0061430 | bone trabecula morphogenesis | 1/59 | 13/17913 | 0.041995814 | 0.251073685 | 0.21477 |
| GO_BP_Profile12 | GO:0090594 | inflammatory response to wounding | 1/59 | 13/17913 | 0.041995814 | 0.251073685 | 0.21477 |
| GO_BP_Profile12 | GO:0097530 | granulocyte migration | 2/59 | 100/17913 | 0.042971952 | 0.255586481 | 0.21863 |
| GO_BP_Profile12 | GO:0001771 | immunological synapse formation | 1/59 | 14/17913 | 0.045153481 | 0.255586481 | 0.21863 |
| GO_BP_Profile12 | GO:0002834 | regulation of response to tumor cell | 1/59 | 14/17913 | 0.045153481 | 0.255586481 | 0.21863 |
| GO_BP_Profile12 | GO:0002837 | regulation of immune response to tumor cell | 1/59 | 14/17913 | 0.045153481 | 0.255586481 | 0.21863 |
| GO_BP_Profile12 | GO:0007035 | vacuolar acidification | 1/59 | 14/17913 | 0.045153481 | 0.255586481 | 0.21863 |
| GO_BP_Profile12 | GO:0010917 | negative regulation of mitochondrial membrane potential | 1/59 | 14/17913 | 0.045153481 | 0.255586481 | 0.21863 |
| GO_BP_Profile12 | GO:0030207 | chondroitin sulfate catabolic process | 1/59 | 14/17913 | 0.045153481 | 0.255586481 | 0.21863 |
| GO_BP_Profile12 | GO:0035461 | vitamin transmembrane transport | 1/59 | 14/17913 | 0.045153481 | 0.255586481 | 0.21863 |
| GO_BP_Profile12 | GO:0045591 | positive regulation of regulatory T cell differentiation | 1/59 | 14/17913 | 0.045153481 | 0.255586481 | 0.21863 |
| GO_BP_Profile12 | GO:0045651 | positive regulation of macrophage differentiation | 1/59 | 14/17913 | 0.045153481 | 0.255586481 | 0.21863 |
| GO_BP_Profile12 | GO:0045953 | negative regulation of natural killer cell mediated cytotoxicity | 1/59 | 14/17913 | 0.045153481 | 0.255586481 | 0.21863 |
| GO_BP_Profile12 | GO:2000095 | regulation of Wnt signaling pathway, planar cell polarity pathway | 1/59 | 14/17913 | 0.045153481 | 0.255586481 | 0.21863 |
| GO_BP_Profile12 | GO:0007034 | vacuolar transport | 2/59 | 103/17913 | 0.045319355 | 0.255586481 | 0.21863 |
| GO_BP_Profile12 | GO:0032091 | negative regulation of protein binding | 2/59 | 103/17913 | 0.045319355 | 0.255586481 | 0.21863 |
| GO_BP_Profile12 | GO:0050868 | negative regulation of T cell activation | 2/59 | 104/17913 | 0.046112231 | 0.257789949 | 0.220515 |
| GO_BP_Profile12 | GO:0002688 | regulation of leukocyte chemotaxis | 2/59 | 105/17913 | 0.046910243 | 0.257789949 | 0.220515 |
| GO_BP_Profile12 | GO:0032943 | mononuclear cell proliferation | 3/59 | 246/17913 | 0.047416552 | 0.257789949 | 0.220515 |
| GO_BP_Profile12 | GO:0002716 | negative regulation of natural killer cell mediated immunity | 1/59 | 15/17913 | 0.048300917 | 0.257789949 | 0.220515 |
| GO_BP_Profile12 | GO:0032105 | negative regulation of response to extracellular stimulus | 1/59 | 15/17913 | 0.048300917 | 0.257789949 | 0.220515 |
| GO_BP_Profile12 | GO:0032108 | negative regulation of response to nutrient levels | 1/59 | 15/17913 | 0.048300917 | 0.257789949 | 0.220515 |
| GO_BP_Profile12 | GO:0033689 | negative regulation of osteoblast proliferation | 1/59 | 15/17913 | 0.048300917 | 0.257789949 | 0.220515 |
| GO_BP_Profile12 | GO:0035930 | corticosteroid hormone secretion | 1/59 | 15/17913 | 0.048300917 | 0.257789949 | 0.220515 |
| GO_BP_Profile12 | GO:0043931 | ossification involved in bone maturation | 1/59 | 15/17913 | 0.048300917 | 0.257789949 | 0.220515 |
| GO_BP_Profile12 | GO:0045837 | negative regulation of membrane potential | 1/59 | 15/17913 | 0.048300917 | 0.257789949 | 0.220515 |
| GO_BP_Profile12 | GO:0055089 | fatty acid homeostasis | 1/59 | 15/17913 | 0.048300917 | 0.257789949 | 0.220515 |
| GO_BP_Profile12 | GO:0071380 | cellular response to prostaglandin E stimulus | 1/59 | 15/17913 | 0.048300917 | 0.257789949 | 0.220515 |
| GO_BP_Profile12 | GO:0071474 | cellular hyperosmotic response | 1/59 | 15/17913 | 0.048300917 | 0.257789949 | 0.220515 |
| GO_BP_Profile12 | GO:1902001 | fatty acid transmembrane transport | 1/59 | 15/17913 | 0.048300917 | 0.257789949 | 0.220515 |
| GO_BP_Profile15 | GO:0046946 | hydroxylysine metabolic process | 2/95 | 3/17913 | 8.32059E-05 | 0.067989152 | 0.055855 |
| GO_BP_Profile15 | GO:0046947 | hydroxylysine biosynthetic process | 2/95 | 3/17913 | 8.32059E-05 | 0.067989152 | 0.055855 |
| GO_BP_Profile15 | GO:0051384 | response to glucocorticoid | 6/95 | 139/17913 | 9.6638E-05 | 0.067989152 | 0.055855 |
| GO_BP_Profile15 | GO:0097327 | response to antineoplastic agent | 5/95 | 96/17913 | 0.000157573 | 0.067989152 | 0.055855 |
| GO_BP_Profile15 | GO:0048545 | response to steroid hormone | 9/95 | 375/17913 | 0.000168944 | 0.067989152 | 0.055855 |
| GO_BP_Profile15 | GO:0031960 | response to corticosteroid | 6/95 | 155/17913 | 0.000175607 | 0.067989152 | 0.055855 |
| GO_BP_Profile15 | GO:0071398 | cellular response to fatty acid | 4/95 | 56/17913 | 0.000220756 | 0.073259561 | 0.060185 |
| GO_BP_Profile15 | GO:0007159 | leukocyte cell-cell adhesion | 8/95 | 328/17913 | 0.000354068 | 0.086949405 | 0.071432 |
| GO_BP_Profile15 | GO:0001101 | response to acid chemical | 8/95 | 330/17913 | 0.000368715 | 0.086949405 | 0.071432 |
| GO_BP_Profile15 | GO:0014823 | response to activity | 4/95 | 65/17913 | 0.000392385 | 0.086949405 | 0.071432 |
| GO_BP_Profile15 | GO:1903003 | positive regulation of protein deubiquitination | 2/95 | 6/17913 | 0.000411728 | 0.086949405 | 0.071432 |
| GO_BP_Profile15 | GO:1901654 | response to ketone | 6/95 | 191/17913 | 0.000537819 | 0.095906039 | 0.07879 |
| GO_BP_Profile15 | GO:0061744 | motor behavior | 2/95 | 7/17913 | 0.000574427 | 0.095906039 | 0.07879 |
| GO_BP_Profile15 | GO:0001666 | response to hypoxia | 8/95 | 354/17913 | 0.000586434 | 0.095906039 | 0.07879 |
| GO_BP_Profile15 | GO:0016486 | peptide hormone processing | 3/95 | 32/17913 | 0.000641085 | 0.095906039 | 0.07879 |
| GO_BP_Profile15 | GO:0071229 | cellular response to acid chemical | 6/95 | 199/17913 | 0.000667201 | 0.095906039 | 0.07879 |
| GO_BP_Profile15 | GO:0036293 | response to decreased oxygen levels | 8/95 | 365/17913 | 0.000716152 | 0.095906039 | 0.07879 |
| GO_BP_Profile15 | GO:0017185 | peptidyl-lysine hydroxylation | 2/95 | 8/17913 | 0.000763256 | 0.095906039 | 0.07879 |
| GO_BP_Profile15 | GO:1905954 | positive regulation of lipid localization | 4/95 | 78/17913 | 0.000784423 | 0.095906039 | 0.07879 |
| GO_BP_Profile15 | GO:0060586 | multicellular organismal iron ion homeostasis | 2/95 | 9/17913 | 0.00097794 | 0.100584582 | 0.082634 |
| GO_BP_Profile15 | GO:1903037 | regulation of leukocyte cell-cell adhesion | 7/95 | 295/17913 | 0.000982349 | 0.100584582 | 0.082634 |
| GO_BP_Profile15 | GO:0045834 | positive regulation of lipid metabolic process | 5/95 | 144/17913 | 0.001015634 | 0.100584582 | 0.082634 |
| GO_BP_Profile15 | GO:0022407 | regulation of cell-cell adhesion | 8/95 | 389/17913 | 0.001080085 | 0.100584582 | 0.082634 |
| GO_BP_Profile15 | GO:0070482 | response to oxygen levels | 8/95 | 389/17913 | 0.001080085 | 0.100584582 | 0.082634 |
| GO_BP_Profile15 | GO:0070542 | response to fatty acid | 4/95 | 85/17913 | 0.001082486 | 0.100584582 | 0.082634 |
| GO_BP_Profile15 | GO:0071548 | response to dexamethasone | 3/95 | 39/17913 | 0.001149952 | 0.10274377 | 0.084407 |
| GO_BP_Profile15 | GO:0010749 | regulation of nitric oxide mediated signal transduction | 2/95 | 10/17913 | 0.001218206 | 0.103114809 | 0.084712 |
| GO_BP_Profile15 | GO:1905952 | regulation of lipid localization | 5/95 | 151/17913 | 0.001254949 | 0.103114809 | 0.084712 |
| GO_BP_Profile15 | GO:0009636 | response to toxic substance | 9/95 | 499/17913 | 0.001323592 | 0.103114809 | 0.084712 |
| GO_BP_Profile15 | GO:0019216 | regulation of lipid metabolic process | 8/95 | 402/17913 | 0.001331659 | 0.103114809 | 0.084712 |
| GO_BP_Profile15 | GO:0090085 | regulation of protein deubiquitination | 2/95 | 11/17913 | 0.00148378 | 0.111014564 | 0.091202 |
| GO_BP_Profile15 | GO:0010883 | regulation of lipid storage | 3/95 | 43/17913 | 0.001529258 | 0.111014564 | 0.091202 |
| GO_BP_Profile15 | GO:0042398 | cellular modified amino acid biosynthetic process | 3/95 | 44/17913 | 0.001634887 | 0.115086163 | 0.094547 |
| GO_BP_Profile15 | GO:0002002 | regulation of angiotensin levels in blood | 2/95 | 12/17913 | 0.001774395 | 0.117769117 | 0.096751 |
| GO_BP_Profile15 | GO:0002003 | angiotensin maturation | 2/95 | 12/17913 | 0.001774395 | 0.117769117 | 0.096751 |
| GO_BP_Profile15 | GO:0014075 | response to amine | 3/95 | 46/17913 | 0.001859594 | 0.119995475 | 0.09858 |
| GO_BP_Profile15 | GO:0009066 | aspartate family amino acid metabolic process | 3/95 | 47/17913 | 0.001978804 | 0.124236799 | 0.102065 |
| GO_BP_Profile15 | GO:0006825 | copper ion transport | 2/95 | 14/17913 | 0.002429676 | 0.146263181 | 0.12016 |
| GO_BP_Profile15 | GO:1901607 | alpha-amino acid biosynthetic process | 3/95 | 51/17913 | 0.002502836 | 0.146263181 | 0.12016 |
| GO_BP_Profile15 | GO:0043112 | receptor metabolic process | 5/95 | 177/17913 | 0.002518522 | 0.146263181 | 0.12016 |
| GO_BP_Profile15 | GO:0010508 | positive regulation of autophagy | 4/95 | 113/17913 | 0.003075626 | 0.157511429 | 0.129401 |
| GO_BP_Profile15 | GO:0031295 | T cell costimulation | 3/95 | 55/17913 | 0.003105245 | 0.157511429 | 0.129401 |
| GO_BP_Profile15 | GO:0009410 | response to xenobiotic stimulus | 6/95 | 270/17913 | 0.003147549 | 0.157511429 | 0.129401 |
| GO_BP_Profile15 | GO:0090153 | regulation of sphingolipid biosynthetic process | 2/95 | 16/17913 | 0.003181934 | 0.157511429 | 0.129401 |
| GO_BP_Profile15 | GO:1905038 | regulation of membrane lipid metabolic process | 2/95 | 16/17913 | 0.003181934 | 0.157511429 | 0.129401 |
| GO_BP_Profile15 | GO:2000303 | regulation of ceramide biosynthetic process | 2/95 | 16/17913 | 0.003181934 | 0.157511429 | 0.129401 |
| GO_BP_Profile15 | GO:0031294 | lymphocyte costimulation | 3/95 | 56/17913 | 0.003268518 | 0.157511429 | 0.129401 |
| GO_BP_Profile15 | GO:0009067 | aspartate family amino acid biosynthetic process | 2/95 | 17/17913 | 0.003593778 | 0.157511429 | 0.129401 |
| GO_BP_Profile15 | GO:0046890 | regulation of lipid biosynthetic process | 5/95 | 195/17913 | 0.003814584 | 0.157511429 | 0.129401 |
| GO_BP_Profile15 | GO:0034101 | erythrocyte homeostasis | 4/95 | 120/17913 | 0.003815232 | 0.157511429 | 0.129401 |
| GO_BP_Profile15 | GO:0046031 | ADP metabolic process | 4/95 | 121/17913 | 0.003929878 | 0.157511429 | 0.129401 |
| GO_BP_Profile15 | GO:1903038 | negative regulation of leukocyte cell-cell adhesion | 4/95 | 121/17913 | 0.003929878 | 0.157511429 | 0.129401 |
| GO_BP_Profile15 | GO:0051235 | maintenance of location | 6/95 | 286/17913 | 0.00416985 | 0.157511429 | 0.129401 |
| GO_BP_Profile15 | GO:1901605 | alpha-amino acid metabolic process | 5/95 | 200/17913 | 0.004246445 | 0.157511429 | 0.129401 |
| GO_BP_Profile15 | GO:0001991 | regulation of systemic arterial blood pressure by circulatory renin-angiotensin | 2/95 | 19/17913 | 0.004487609 | 0.157511429 | 0.129401 |
| GO_BP_Profile15 | GO:0030949 | positive regulation of vascular endothelial growth factor receptor signaling pathway | 2/95 | 19/17913 | 0.004487609 | 0.157511429 | 0.129401 |
| GO_BP_Profile15 | GO:0019915 | lipid storage | 3/95 | 64/17913 | 0.004765135 | 0.157511429 | 0.129401 |
| GO_BP_Profile15 | GO:0071216 | cellular response to biotic stimulus | 5/95 | 206/17913 | 0.00480953 | 0.157511429 | 0.129401 |
| GO_BP_Profile15 | GO:0016485 | protein processing | 6/95 | 295/17913 | 0.00484318 | 0.157511429 | 0.129401 |
| GO_BP_Profile15 | GO:0045821 | positive regulation of glycolytic process | 2/95 | 20/17913 | 0.004969088 | 0.157511429 | 0.129401 |
| GO_BP_Profile15 | GO:0000053 | argininosuccinate metabolic process | 1/95 | 1/17913 | 0.005303411 | 0.157511429 | 0.129401 |
| GO_BP_Profile15 | GO:0001695 | histamine catabolic process | 1/95 | 1/17913 | 0.005303411 | 0.157511429 | 0.129401 |
| GO_BP_Profile15 | GO:0002264 | endothelial cell activation involved in immune response | 1/95 | 1/17913 | 0.005303411 | 0.157511429 | 0.129401 |
| GO_BP_Profile15 | GO:0015675 | nickel cation transport | 1/95 | 1/17913 | 0.005303411 | 0.157511429 | 0.129401 |
| GO_BP_Profile15 | GO:0015692 | lead ion transport | 1/95 | 1/17913 | 0.005303411 | 0.157511429 | 0.129401 |
| GO_BP_Profile15 | GO:0035444 | nickel cation transmembrane transport | 1/95 | 1/17913 | 0.005303411 | 0.157511429 | 0.129401 |
| GO_BP_Profile15 | GO:0036323 | vascular endothelial growth factor receptor-1 signaling pathway | 1/95 | 1/17913 | 0.005303411 | 0.157511429 | 0.129401 |
| GO_BP_Profile15 | GO:0046317 | regulation of glucosylceramide biosynthetic process | 1/95 | 1/17913 | 0.005303411 | 0.157511429 | 0.129401 |
| GO_BP_Profile15 | GO:0046318 | negative regulation of glucosylceramide biosynthetic process | 1/95 | 1/17913 | 0.005303411 | 0.157511429 | 0.129401 |
| GO_BP_Profile15 | GO:0061772 | drug transport across blood-nerve barrier | 1/95 | 1/17913 | 0.005303411 | 0.157511429 | 0.129401 |
| GO_BP_Profile15 | GO:0072366 | regulation of cellular ketone metabolic process by positive regulation of transcription from RNA polymerase II promoter | 1/95 | 1/17913 | 0.005303411 | 0.157511429 | 0.129401 |
| GO_BP_Profile15 | GO:0085032 | modulation by symbiont of host I-kappaB kinase/NF-kappaB cascade | 1/95 | 1/17913 | 0.005303411 | 0.157511429 | 0.129401 |
| GO_BP_Profile15 | GO:1903544 | response to butyrate | 1/95 | 1/17913 | 0.005303411 | 0.157511429 | 0.129401 |
| GO_BP_Profile15 | GO:1903545 | cellular response to butyrate | 1/95 | 1/17913 | 0.005303411 | 0.157511429 | 0.129401 |
| GO_BP_Profile15 | GO:1903570 | regulation of protein kinase D signaling | 1/95 | 1/17913 | 0.005303411 | 0.157511429 | 0.129401 |
| GO_BP_Profile15 | GO:1903572 | positive regulation of protein kinase D signaling | 1/95 | 1/17913 | 0.005303411 | 0.157511429 | 0.129401 |
| GO_BP_Profile15 | GO:0051701 | interaction with host | 5/95 | 211/17913 | 0.005317796 | 0.157511429 | 0.129401 |
| GO_BP_Profile15 | GO:0010884 | positive regulation of lipid storage | 2/95 | 21/17913 | 0.005473274 | 0.157511429 | 0.129401 |
| GO_BP_Profile15 | GO:0010893 | positive regulation of steroid biosynthetic process | 2/95 | 21/17913 | 0.005473274 | 0.157511429 | 0.129401 |
| GO_BP_Profile15 | GO:0009135 | purine nucleoside diphosphate metabolic process | 4/95 | 133/17913 | 0.005492219 | 0.157511429 | 0.129401 |
| GO_BP_Profile15 | GO:0009179 | purine ribonucleoside diphosphate metabolic process | 4/95 | 133/17913 | 0.005492219 | 0.157511429 | 0.129401 |
| GO_BP_Profile15 | GO:0007517 | muscle organ development | 7/95 | 403/17913 | 0.005611501 | 0.158969705 | 0.130599 |
| GO_BP_Profile15 | GO:0042445 | hormone metabolic process | 5/95 | 215/17913 | 0.005750959 | 0.160053836 | 0.13149 |
| GO_BP_Profile15 | GO:0009185 | ribonucleoside diphosphate metabolic process | 4/95 | 135/17913 | 0.005787569 | 0.160053836 | 0.13149 |
| GO_BP_Profile15 | GO:0018126 | protein hydroxylation | 2/95 | 22/17913 | 0.005999917 | 0.160204692 | 0.131613 |
| GO_BP_Profile15 | GO:0030813 | positive regulation of nucleotide catabolic process | 2/95 | 22/17913 | 0.005999917 | 0.160204692 | 0.131613 |
| GO_BP_Profile15 | GO:0051197 | positive regulation of coenzyme metabolic process | 2/95 | 22/17913 | 0.005999917 | 0.160204692 | 0.131613 |
| GO_BP_Profile15 | GO:0002347 | response to tumor cell | 2/95 | 23/17913 | 0.006548771 | 0.172872677 | 0.142021 |
| GO_BP_Profile15 | GO:0008652 | cellular amino acid biosynthetic process | 3/95 | 73/17913 | 0.006873967 | 0.175913147 | 0.144518 |
| GO_BP_Profile15 | GO:0062013 | positive regulation of small molecule metabolic process | 4/95 | 143/17913 | 0.007074758 | 0.175913147 | 0.144518 |
| GO_BP_Profile15 | GO:0070373 | negative regulation of ERK1 and ERK2 cascade | 3/95 | 74/17913 | 0.007137245 | 0.175913147 | 0.144518 |
| GO_BP_Profile15 | GO:0006110 | regulation of glycolytic process | 3/95 | 75/17913 | 0.007406441 | 0.175913147 | 0.144518 |
| GO_BP_Profile15 | GO:0002262 | myeloid cell homeostasis | 4/95 | 145/17913 | 0.007423771 | 0.175913147 | 0.144518 |
| GO_BP_Profile15 | GO:0001655 | urogenital system development | 6/95 | 324/17913 | 0.00756139 | 0.175913147 | 0.144518 |
| GO_BP_Profile15 | GO:0030811 | regulation of nucleotide catabolic process | 3/95 | 77/17913 | 0.007962709 | 0.175913147 | 0.144518 |
| GO_BP_Profile15 | GO:0045913 | positive regulation of carbohydrate metabolic process | 3/95 | 78/17913 | 0.00824984 | 0.175913147 | 0.144518 |
| GO_BP_Profile15 | GO:0007263 | nitric oxide mediated signal transduction | 2/95 | 26/17913 | 0.008326151 | 0.175913147 | 0.144518 |
| GO_BP_Profile15 | GO:0010038 | response to metal ion | 6/95 | 335/17913 | 0.008835776 | 0.175913147 | 0.144518 |
| GO_BP_Profile15 | GO:0050886 | endocrine process | 3/95 | 80/17913 | 0.008842238 | 0.175913147 | 0.144518 |
| GO_BP_Profile15 | GO:0009132 | nucleoside diphosphate metabolic process | 4/95 | 153/17913 | 0.008933103 | 0.175913147 | 0.144518 |
| GO_BP_Profile15 | GO:0003081 | regulation of systemic arterial blood pressure by renin-angiotensin | 2/95 | 27/17913 | 0.008961413 | 0.175913147 | 0.144518 |
| GO_BP_Profile15 | GO:0031331 | positive regulation of cellular catabolic process | 6/95 | 341/17913 | 0.009592533 | 0.175913147 | 0.144518 |
| GO_BP_Profile15 | GO:0006972 | hyperosmotic response | 2/95 | 28/17913 | 0.009617677 | 0.175913147 | 0.144518 |
| GO_BP_Profile15 | GO:0009074 | aromatic amino acid family catabolic process | 2/95 | 28/17913 | 0.009617677 | 0.175913147 | 0.144518 |
| GO_BP_Profile15 | GO:1902932 | positive regulation of alcohol biosynthetic process | 2/95 | 28/17913 | 0.009617677 | 0.175913147 | 0.144518 |
| GO_BP_Profile15 | GO:0043470 | regulation of carbohydrate catabolic process | 3/95 | 84/17913 | 0.010100301 | 0.175913147 | 0.144518 |
| GO_BP_Profile15 | GO:0055006 | cardiac cell development | 3/95 | 84/17913 | 0.010100301 | 0.175913147 | 0.144518 |
| GO_BP_Profile15 | GO:0045940 | positive regulation of steroid metabolic process | 2/95 | 29/17913 | 0.010294708 | 0.175913147 | 0.144518 |
| GO_BP_Profile15 | GO:0071549 | cellular response to dexamethasone stimulus | 2/95 | 29/17913 | 0.010294708 | 0.175913147 | 0.144518 |
| GO_BP_Profile15 | GO:1903901 | negative regulation of viral life cycle | 3/95 | 85/17913 | 0.010430221 | 0.175913147 | 0.144518 |
| GO_BP_Profile15 | GO:0000294 | nuclear-transcribed mRNA catabolic process, endonucleolytic cleavage-dependent decay | 1/95 | 2/17913 | 0.01057899 | 0.175913147 | 0.144518 |
| GO_BP_Profile15 | GO:0001970 | positive regulation of activation of membrane attack complex | 1/95 | 2/17913 | 0.01057899 | 0.175913147 | 0.144518 |
| GO_BP_Profile15 | GO:0003169 | coronary vein morphogenesis | 1/95 | 2/17913 | 0.01057899 | 0.175913147 | 0.144518 |
| GO_BP_Profile15 | GO:0006196 | AMP catabolic process | 1/95 | 2/17913 | 0.01057899 | 0.175913147 | 0.144518 |
| GO_BP_Profile15 | GO:0006526 | arginine biosynthetic process | 1/95 | 2/17913 | 0.01057899 | 0.175913147 | 0.144518 |
| GO_BP_Profile15 | GO:0017186 | peptidyl-pyroglutamic acid biosynthetic process, using glutaminyl-peptide cyclotransferase | 1/95 | 2/17913 | 0.01057899 | 0.175913147 | 0.144518 |
| GO_BP_Profile15 | GO:0032346 | positive regulation of aldosterone metabolic process | 1/95 | 2/17913 | 0.01057899 | 0.175913147 | 0.144518 |
| GO_BP_Profile15 | GO:0032349 | positive regulation of aldosterone biosynthetic process | 1/95 | 2/17913 | 0.01057899 | 0.175913147 | 0.144518 |
| GO_BP_Profile15 | GO:0033212 | iron assimilation | 1/95 | 2/17913 | 0.01057899 | 0.175913147 | 0.144518 |
| GO_BP_Profile15 | GO:0035772 | interleukin-13-mediated signaling pathway | 1/95 | 2/17913 | 0.01057899 | 0.175913147 | 0.144518 |
| GO_BP_Profile15 | GO:0038190 | VEGF-activated neuropilin signaling pathway | 1/95 | 2/17913 | 0.01057899 | 0.175913147 | 0.144518 |
| GO_BP_Profile15 | GO:0051866 | general adaptation syndrome | 1/95 | 2/17913 | 0.01057899 | 0.175913147 | 0.144518 |
| GO_BP_Profile15 | GO:0052027 | modulation by symbiont of host signal transduction pathway | 1/95 | 2/17913 | 0.01057899 | 0.175913147 | 0.144518 |
| GO_BP_Profile15 | GO:0070667 | negative regulation of mast cell proliferation | 1/95 | 2/17913 | 0.01057899 | 0.175913147 | 0.144518 |
| GO_BP_Profile15 | GO:0070668 | positive regulation of mast cell proliferation | 1/95 | 2/17913 | 0.01057899 | 0.175913147 | 0.144518 |
| GO_BP_Profile15 | GO:0071228 | cellular response to tumor cell | 1/95 | 2/17913 | 0.01057899 | 0.175913147 | 0.144518 |
| GO_BP_Profile15 | GO:0071418 | cellular response to amine stimulus | 1/95 | 2/17913 | 0.01057899 | 0.175913147 | 0.144518 |
| GO_BP_Profile15 | GO:0072363 | regulation of glycolytic process by positive regulation of transcription from RNA polymerase II promoter | 1/95 | 2/17913 | 0.01057899 | 0.175913147 | 0.144518 |
| GO_BP_Profile15 | GO:0072364 | regulation of cellular ketone metabolic process by regulation of transcription from RNA polymerase II promoter | 1/95 | 2/17913 | 0.01057899 | 0.175913147 | 0.144518 |
| GO_BP_Profile15 | GO:0072369 | regulation of lipid transport by positive regulation of transcription from RNA polymerase II promoter | 1/95 | 2/17913 | 0.01057899 | 0.175913147 | 0.144518 |
| GO_BP_Profile15 | GO:1902336 | positive regulation of retinal ganglion cell axon guidance | 1/95 | 2/17913 | 0.01057899 | 0.175913147 | 0.144518 |
| GO_BP_Profile15 | GO:1903108 | regulation of mitochondrial transcription | 1/95 | 2/17913 | 0.01057899 | 0.175913147 | 0.144518 |
| GO_BP_Profile15 | GO:1903109 | positive regulation of mitochondrial transcription | 1/95 | 2/17913 | 0.01057899 | 0.175913147 | 0.144518 |
| GO_BP_Profile15 | GO:1905371 | ceramide phosphoethanolamine metabolic process | 1/95 | 2/17913 | 0.01057899 | 0.175913147 | 0.144518 |
| GO_BP_Profile15 | GO:1905373 | ceramide phosphoethanolamine biosynthetic process | 1/95 | 2/17913 | 0.01057899 | 0.175913147 | 0.144518 |
| GO_BP_Profile15 | GO:2000627 | positive regulation of miRNA catabolic process | 1/95 | 2/17913 | 0.01057899 | 0.175913147 | 0.144518 |
| GO_BP_Profile15 | GO:2000857 | positive regulation of mineralocorticoid secretion | 1/95 | 2/17913 | 0.01057899 | 0.175913147 | 0.144518 |
| GO_BP_Profile15 | GO:2000860 | positive regulation of aldosterone secretion | 1/95 | 2/17913 | 0.01057899 | 0.175913147 | 0.144518 |
| GO_BP_Profile15 | GO:2000866 | positive regulation of estradiol secretion | 1/95 | 2/17913 | 0.01057899 | 0.175913147 | 0.144518 |
| GO_BP_Profile15 | GO:0032689 | negative regulation of interferon-gamma production | 2/95 | 30/17913 | 0.010992273 | 0.175913147 | 0.144518 |
| GO_BP_Profile15 | GO:0051194 | positive regulation of cofactor metabolic process | 2/95 | 30/17913 | 0.010992273 | 0.175913147 | 0.144518 |
| GO_BP_Profile15 | GO:0060326 | cell chemotaxis | 5/95 | 253/17913 | 0.011177778 | 0.175913147 | 0.144518 |
| GO_BP_Profile15 | GO:0051604 | protein maturation | 6/95 | 353/17913 | 0.01124339 | 0.175913147 | 0.144518 |
| GO_BP_Profile15 | GO:0060538 | skeletal muscle organ development | 4/95 | 165/17913 | 0.011551971 | 0.175913147 | 0.144518 |
| GO_BP_Profile15 | GO:0044068 | modulation by symbiont of host cellular process | 2/95 | 31/17913 | 0.011710139 | 0.175913147 | 0.144518 |
| GO_BP_Profile15 | GO:0051196 | regulation of coenzyme metabolic process | 3/95 | 90/17913 | 0.012173318 | 0.175913147 | 0.144518 |
| GO_BP_Profile15 | GO:2001169 | regulation of ATP biosynthetic process | 3/95 | 90/17913 | 0.012173318 | 0.175913147 | 0.144518 |
| GO_BP_Profile15 | GO:0043552 | positive regulation of phosphatidylinositol 3-kinase activity | 2/95 | 32/17913 | 0.012448076 | 0.175913147 | 0.144518 |
| GO_BP_Profile15 | GO:1900745 | positive regulation of p38MAPK cascade | 2/95 | 32/17913 | 0.012448076 | 0.175913147 | 0.144518 |
| GO_BP_Profile15 | GO:2001171 | positive regulation of ATP biosynthetic process | 2/95 | 32/17913 | 0.012448076 | 0.175913147 | 0.144518 |
| GO_BP_Profile15 | GO:0009166 | nucleotide catabolic process | 4/95 | 169/17913 | 0.012523638 | 0.175913147 | 0.144518 |
| GO_BP_Profile15 | GO:0022408 | negative regulation of cell-cell adhesion | 4/95 | 169/17913 | 0.012523638 | 0.175913147 | 0.144518 |
| GO_BP_Profile15 | GO:1901655 | cellular response to ketone | 3/95 | 91/17913 | 0.012540769 | 0.175913147 | 0.144518 |
| GO_BP_Profile15 | GO:0010039 | response to iron ion | 2/95 | 33/17913 | 0.013205856 | 0.175913147 | 0.144518 |
| GO_BP_Profile15 | GO:0030947 | regulation of vascular endothelial growth factor receptor signaling pathway | 2/95 | 33/17913 | 0.013205856 | 0.175913147 | 0.144518 |
| GO_BP_Profile15 | GO:1901890 | positive regulation of cell junction assembly | 2/95 | 33/17913 | 0.013205856 | 0.175913147 | 0.144518 |
| GO_BP_Profile15 | GO:0010876 | lipid localization | 6/95 | 366/17913 | 0.013249174 | 0.175913147 | 0.144518 |
| GO_BP_Profile15 | GO:0032649 | regulation of interferon-gamma production | 3/95 | 93/17913 | 0.013294642 | 0.175913147 | 0.144518 |
| GO_BP_Profile15 | GO:0009152 | purine ribonucleotide biosynthetic process | 5/95 | 266/17913 | 0.013641301 | 0.175913147 | 0.144518 |
| GO_BP_Profile15 | GO:0071356 | cellular response to tumor necrosis factor | 5/95 | 266/17913 | 0.013641301 | 0.175913147 | 0.144518 |
| GO_BP_Profile15 | GO:0050727 | regulation of inflammatory response | 7/95 | 479/17913 | 0.013735732 | 0.175913147 | 0.144518 |
| GO_BP_Profile15 | GO:0009127 | purine nucleoside monophosphate biosynthetic process | 4/95 | 174/17913 | 0.013809929 | 0.175913147 | 0.144518 |
| GO_BP_Profile15 | GO:0009168 | purine ribonucleoside monophosphate biosynthetic process | 4/95 | 174/17913 | 0.013809929 | 0.175913147 | 0.144518 |
| GO_BP_Profile15 | GO:0097009 | energy homeostasis | 2/95 | 34/17913 | 0.013983251 | 0.175913147 | 0.144518 |
| GO_BP_Profile15 | GO:0050810 | regulation of steroid biosynthetic process | 3/95 | 95/17913 | 0.014073908 | 0.175913147 | 0.144518 |
| GO_BP_Profile15 | GO:1901292 | nucleoside phosphate catabolic process | 4/95 | 175/17913 | 0.014076884 | 0.175913147 | 0.144518 |
| GO_BP_Profile15 | GO:0071222 | cellular response to lipopolysaccharide | 4/95 | 176/17913 | 0.014347102 | 0.175913147 | 0.144518 |
| GO_BP_Profile15 | GO:0043312 | neutrophil degranulation | 7/95 | 485/17913 | 0.014618427 | 0.175913147 | 0.144518 |
| GO_BP_Profile15 | GO:0060416 | response to growth hormone | 2/95 | 35/17913 | 0.014780038 | 0.175913147 | 0.144518 |
| GO_BP_Profile15 | GO:0071542 | dopaminergic neuron differentiation | 2/95 | 35/17913 | 0.014780038 | 0.175913147 | 0.144518 |
| GO_BP_Profile15 | GO:0002283 | neutrophil activation involved in immune response | 7/95 | 488/17913 | 0.015074716 | 0.175913147 | 0.144518 |
| GO_BP_Profile15 | GO:0006575 | cellular modified amino acid metabolic process | 4/95 | 179/17913 | 0.015177457 | 0.175913147 | 0.144518 |
| GO_BP_Profile15 | GO:0090218 | positive regulation of lipid kinase activity | 2/95 | 36/17913 | 0.015595992 | 0.175913147 | 0.144518 |
| GO_BP_Profile15 | GO:0048525 | negative regulation of viral process | 3/95 | 99/17913 | 0.015709043 | 0.175913147 | 0.144518 |
| GO_BP_Profile15 | GO:0001822 | kidney development | 5/95 | 276/17913 | 0.01576897 | 0.175913147 | 0.144518 |
| GO_BP_Profile15 | GO:0001692 | histamine metabolic process | 1/95 | 3/17913 | 0.015826882 | 0.175913147 | 0.144518 |
| GO_BP_Profile15 | GO:0001798 | positive regulation of type IIa hypersensitivity | 1/95 | 3/17913 | 0.015826882 | 0.175913147 | 0.144518 |
| GO_BP_Profile15 | GO:0001905 | activation of membrane attack complex | 1/95 | 3/17913 | 0.015826882 | 0.175913147 | 0.144518 |
| GO_BP_Profile15 | GO:0001969 | regulation of activation of membrane attack complex | 1/95 | 3/17913 | 0.015826882 | 0.175913147 | 0.144518 |
| GO_BP_Profile15 | GO:0002774 | Fc receptor mediated inhibitory signaling pathway | 1/95 | 3/17913 | 0.015826882 | 0.175913147 | 0.144518 |
| GO_BP_Profile15 | GO:0002894 | positive regulation of type II hypersensitivity | 1/95 | 3/17913 | 0.015826882 | 0.175913147 | 0.144518 |
| GO_BP_Profile15 | GO:0006679 | glucosylceramide biosynthetic process | 1/95 | 3/17913 | 0.015826882 | 0.175913147 | 0.144518 |
| GO_BP_Profile15 | GO:0008065 | establishment of blood-nerve barrier | 1/95 | 3/17913 | 0.015826882 | 0.175913147 | 0.144518 |
| GO_BP_Profile15 | GO:0009631 | cold acclimation | 1/95 | 3/17913 | 0.015826882 | 0.175913147 | 0.144518 |
| GO_BP_Profile15 | GO:0021538 | epithalamus development | 1/95 | 3/17913 | 0.015826882 | 0.175913147 | 0.144518 |
| GO_BP_Profile15 | GO:0021986 | habenula development | 1/95 | 3/17913 | 0.015826882 | 0.175913147 | 0.144518 |
| GO_BP_Profile15 | GO:0035938 | estradiol secretion | 1/95 | 3/17913 | 0.015826882 | 0.175913147 | 0.144518 |
| GO_BP_Profile15 | GO:0038086 | VEGF-activated platelet-derived growth factor receptor signaling pathway | 1/95 | 3/17913 | 0.015826882 | 0.175913147 | 0.144518 |
| GO_BP_Profile15 | GO:0038091 | positive regulation of cell proliferation by VEGF-activated platelet derived growth factor receptor signaling pathway | 1/95 | 3/17913 | 0.015826882 | 0.175913147 | 0.144518 |
| GO_BP_Profile15 | GO:0060940 | epithelial to mesenchymal transition involved in cardiac fibroblast development | 1/95 | 3/17913 | 0.015826882 | 0.175913147 | 0.144518 |
| GO_BP_Profile15 | GO:0060948 | cardiac vascular smooth muscle cell development | 1/95 | 3/17913 | 0.015826882 | 0.175913147 | 0.144518 |
| GO_BP_Profile15 | GO:0070447 | positive regulation of oligodendrocyte progenitor proliferation | 1/95 | 3/17913 | 0.015826882 | 0.175913147 | 0.144518 |
| GO_BP_Profile15 | GO:0070666 | regulation of mast cell proliferation | 1/95 | 3/17913 | 0.015826882 | 0.175913147 | 0.144518 |
| GO_BP_Profile15 | GO:0071400 | cellular response to oleic acid | 1/95 | 3/17913 | 0.015826882 | 0.175913147 | 0.144518 |
| GO_BP_Profile15 | GO:0072361 | regulation of glycolytic process by regulation of transcription from RNA polymerase II promoter | 1/95 | 3/17913 | 0.015826882 | 0.175913147 | 0.144518 |
| GO_BP_Profile15 | GO:0089700 | protein kinase D signaling | 1/95 | 3/17913 | 0.015826882 | 0.175913147 | 0.144518 |
| GO_BP_Profile15 | GO:0090031 | positive regulation of steroid hormone biosynthetic process | 1/95 | 3/17913 | 0.015826882 | 0.175913147 | 0.144518 |
| GO_BP_Profile15 | GO:0097532 | stress response to acid chemical | 1/95 | 3/17913 | 0.015826882 | 0.175913147 | 0.144518 |
| GO_BP_Profile15 | GO:0097533 | cellular stress response to acid chemical | 1/95 | 3/17913 | 0.015826882 | 0.175913147 | 0.144518 |
| GO_BP_Profile15 | GO:0098679 | regulation of carbohydrate catabolic process by regulation of transcription from RNA polymerase II promoter | 1/95 | 3/17913 | 0.015826882 | 0.175913147 | 0.144518 |
| GO_BP_Profile15 | GO:0099183 | trans-synaptic signaling by BDNF, modulating synaptic transmission | 1/95 | 3/17913 | 0.015826882 | 0.175913147 | 0.144518 |
| GO_BP_Profile15 | GO:0099191 | trans-synaptic signaling by BDNF | 1/95 | 3/17913 | 0.015826882 | 0.175913147 | 0.144518 |
| GO_BP_Profile15 | GO:0150062 | complement-mediated synapse pruning | 1/95 | 3/17913 | 0.015826882 | 0.175913147 | 0.144518 |
| GO_BP_Profile15 | GO:0150064 | vertebrate eye-specific patterning | 1/95 | 3/17913 | 0.015826882 | 0.175913147 | 0.144518 |
| GO_BP_Profile15 | GO:1903895 | negative regulation of IRE1-mediated unfolded protein response | 1/95 | 3/17913 | 0.015826882 | 0.175913147 | 0.144518 |
| GO_BP_Profile15 | GO:1904475 | regulation of Ras GTPase binding | 1/95 | 3/17913 | 0.015826882 | 0.175913147 | 0.144518 |
| GO_BP_Profile15 | GO:2000625 | regulation of miRNA catabolic process | 1/95 | 3/17913 | 0.015826882 | 0.175913147 | 0.144518 |
| GO_BP_Profile15 | GO:2000864 | regulation of estradiol secretion | 1/95 | 3/17913 | 0.015826882 | 0.175913147 | 0.144518 |
| GO_BP_Profile15 | GO:2001106 | regulation of Rho guanyl-nucleotide exchange factor activity | 1/95 | 3/17913 | 0.015826882 | 0.175913147 | 0.144518 |
| GO_BP_Profile15 | GO:0045765 | regulation of angiogenesis | 6/95 | 383/17913 | 0.016235169 | 0.178440576 | 0.146595 |
| GO_BP_Profile15 | GO:0071219 | cellular response to molecule of bacterial origin | 4/95 | 183/17913 | 0.016331026 | 0.178440576 | 0.146595 |
| GO_BP_Profile15 | GO:0001990 | regulation of systemic arterial blood pressure by hormone | 2/95 | 37/17913 | 0.016430894 | 0.178440576 | 0.146595 |
| GO_BP_Profile15 | GO:0042181 | ketone biosynthetic process | 2/95 | 37/17913 | 0.016430894 | 0.178440576 | 0.146595 |
| GO_BP_Profile15 | GO:0009260 | ribonucleotide biosynthetic process | 5/95 | 279/17913 | 0.01644817 | 0.178440576 | 0.146595 |
| GO_BP_Profile15 | GO:0006164 | purine nucleotide biosynthetic process | 5/95 | 280/17913 | 0.01667884 | 0.178440576 | 0.146595 |
| GO_BP_Profile15 | GO:0002446 | neutrophil mediated immunity | 7/95 | 499/17913 | 0.016835176 | 0.178440576 | 0.146595 |
| GO_BP_Profile15 | GO:0042119 | neutrophil activation | 7/95 | 499/17913 | 0.016835176 | 0.178440576 | 0.146595 |
| GO_BP_Profile15 | GO:0016052 | carbohydrate catabolic process | 4/95 | 185/17913 | 0.016927918 | 0.178440576 | 0.146595 |
| GO_BP_Profile15 | GO:0002685 | regulation of leukocyte migration | 4/95 | 186/17913 | 0.01723143 | 0.178440576 | 0.146595 |
| GO_BP_Profile15 | GO:0042149 | cellular response to glucose starvation | 2/95 | 38/17913 | 0.017284523 | 0.178440576 | 0.146595 |
| GO_BP_Profile15 | GO:0043277 | apoptotic cell clearance | 2/95 | 38/17913 | 0.017284523 | 0.178440576 | 0.146595 |
| GO_BP_Profile15 | GO:0032526 | response to retinoic acid | 3/95 | 103/17913 | 0.017446903 | 0.178440576 | 0.146595 |
| GO_BP_Profile15 | GO:0034612 | response to tumor necrosis factor | 5/95 | 284/17913 | 0.017623087 | 0.178440576 | 0.146595 |
| GO_BP_Profile15 | GO:0046390 | ribose phosphate biosynthetic process | 5/95 | 284/17913 | 0.017623087 | 0.178440576 | 0.146595 |
| GO_BP_Profile15 | GO:0009156 | ribonucleoside monophosphate biosynthetic process | 4/95 | 188/17913 | 0.017848642 | 0.178440576 | 0.146595 |
| GO_BP_Profile15 | GO:0009896 | positive regulation of catabolic process | 6/95 | 392/17913 | 0.0179915 | 0.178440576 | 0.146595 |
| GO_BP_Profile15 | GO:0009072 | aromatic amino acid family metabolic process | 2/95 | 39/17913 | 0.018156663 | 0.178440576 | 0.146595 |
| GO_BP_Profile15 | GO:0045746 | negative regulation of Notch signaling pathway | 2/95 | 39/17913 | 0.018156663 | 0.178440576 | 0.146595 |
| GO_BP_Profile15 | GO:0032609 | interferon-gamma production | 3/95 | 105/17913 | 0.018354532 | 0.178440576 | 0.146595 |
| GO_BP_Profile15 | GO:0006694 | steroid biosynthetic process | 4/95 | 190/17913 | 0.018479505 | 0.178440576 | 0.146595 |
| GO_BP_Profile15 | GO:0030810 | positive regulation of nucleotide biosynthetic process | 2/95 | 40/17913 | 0.019047095 | 0.178440576 | 0.146595 |
| GO_BP_Profile15 | GO:0038084 | vascular endothelial growth factor signaling pathway | 2/95 | 40/17913 | 0.019047095 | 0.178440576 | 0.146595 |
| GO_BP_Profile15 | GO:1900373 | positive regulation of purine nucleotide biosynthetic process | 2/95 | 40/17913 | 0.019047095 | 0.178440576 | 0.146595 |
| GO_BP_Profile15 | GO:0072001 | renal system development | 5/95 | 291/17913 | 0.019359715 | 0.178440576 | 0.146595 |
| GO_BP_Profile15 | GO:0072522 | purine-containing compound biosynthetic process | 5/95 | 291/17913 | 0.019359715 | 0.178440576 | 0.146595 |
| GO_BP_Profile15 | GO:0006911 | phagocytosis, engulfment | 2/95 | 41/17913 | 0.019955608 | 0.178440576 | 0.146595 |
| GO_BP_Profile15 | GO:1903580 | positive regulation of ATP metabolic process | 2/95 | 41/17913 | 0.019955608 | 0.178440576 | 0.146595 |
| GO_BP_Profile15 | GO:1900371 | regulation of purine nucleotide biosynthetic process | 3/95 | 109/17913 | 0.020247438 | 0.178440576 | 0.146595 |
| GO_BP_Profile15 | GO:0032496 | response to lipopolysaccharide | 5/95 | 295/17913 | 0.020400997 | 0.178440576 | 0.146595 |
| GO_BP_Profile15 | GO:0009124 | nucleoside monophosphate biosynthetic process | 4/95 | 196/17913 | 0.020454804 | 0.178440576 | 0.146595 |
| GO_BP_Profile15 | GO:0006096 | glycolytic process | 3/95 | 110/17913 | 0.020736867 | 0.178440576 | 0.146595 |
| GO_BP_Profile15 | GO:0030808 | regulation of nucleotide biosynthetic process | 3/95 | 110/17913 | 0.020736867 | 0.178440576 | 0.146595 |
| GO_BP_Profile15 | GO:1903578 | regulation of ATP metabolic process | 3/95 | 110/17913 | 0.020736867 | 0.178440576 | 0.146595 |
| GO_BP_Profile15 | GO:0090311 | regulation of protein deacetylation | 2/95 | 42/17913 | 0.020881987 | 0.178440576 | 0.146595 |
| GO_BP_Profile15 | GO:0001794 | type IIa hypersensitivity | 1/95 | 4/17913 | 0.021047231 | 0.178440576 | 0.146595 |
| GO_BP_Profile15 | GO:0001796 | regulation of type IIa hypersensitivity | 1/95 | 4/17913 | 0.021047231 | 0.178440576 | 0.146595 |
| GO_BP_Profile15 | GO:0002445 | type II hypersensitivity | 1/95 | 4/17913 | 0.021047231 | 0.178440576 | 0.146595 |
| GO_BP_Profile15 | GO:0002677 | negative regulation of chronic inflammatory response | 1/95 | 4/17913 | 0.021047231 | 0.178440576 | 0.146595 |
| GO_BP_Profile15 | GO:0002892 | regulation of type II hypersensitivity | 1/95 | 4/17913 | 0.021047231 | 0.178440576 | 0.146595 |
| GO_BP_Profile15 | GO:0009158 | ribonucleoside monophosphate catabolic process | 1/95 | 4/17913 | 0.021047231 | 0.178440576 | 0.146595 |
| GO_BP_Profile15 | GO:0009169 | purine ribonucleoside monophosphate catabolic process | 1/95 | 4/17913 | 0.021047231 | 0.178440576 | 0.146595 |
| GO_BP_Profile15 | GO:0015691 | cadmium ion transport | 1/95 | 4/17913 | 0.021047231 | 0.178440576 | 0.146595 |
| GO_BP_Profile15 | GO:0015879 | carnitine transport | 1/95 | 4/17913 | 0.021047231 | 0.178440576 | 0.146595 |
| GO_BP_Profile15 | GO:0018199 | peptidyl-glutamine modification | 1/95 | 4/17913 | 0.021047231 | 0.178440576 | 0.146595 |
| GO_BP_Profile15 | GO:0032264 | IMP salvage | 1/95 | 4/17913 | 0.021047231 | 0.178440576 | 0.146595 |
| GO_BP_Profile15 | GO:0034436 | glycoprotein transport | 1/95 | 4/17913 | 0.021047231 | 0.178440576 | 0.146595 |
| GO_BP_Profile15 | GO:0035963 | cellular response to interleukin-13 | 1/95 | 4/17913 | 0.021047231 | 0.178440576 | 0.146595 |
| GO_BP_Profile15 | GO:0036343 | psychomotor behavior | 1/95 | 4/17913 | 0.021047231 | 0.178440576 | 0.146595 |
| GO_BP_Profile15 | GO:0038189 | neuropilin signaling pathway | 1/95 | 4/17913 | 0.021047231 | 0.178440576 | 0.146595 |
| GO_BP_Profile15 | GO:0051387 | negative regulation of neurotrophin TRK receptor signaling pathway | 1/95 | 4/17913 | 0.021047231 | 0.178440576 | 0.146595 |
| GO_BP_Profile15 | GO:0060319 | primitive erythrocyte differentiation | 1/95 | 4/17913 | 0.021047231 | 0.178440576 | 0.146595 |
| GO_BP_Profile15 | GO:0060935 | cardiac fibroblast cell differentiation | 1/95 | 4/17913 | 0.021047231 | 0.178440576 | 0.146595 |
| GO_BP_Profile15 | GO:0060936 | cardiac fibroblast cell development | 1/95 | 4/17913 | 0.021047231 | 0.178440576 | 0.146595 |
| GO_BP_Profile15 | GO:0060938 | epicardium-derived cardiac fibroblast cell differentiation | 1/95 | 4/17913 | 0.021047231 | 0.178440576 | 0.146595 |
| GO_BP_Profile15 | GO:0060939 | epicardium-derived cardiac fibroblast cell development | 1/95 | 4/17913 | 0.021047231 | 0.178440576 | 0.146595 |
| GO_BP_Profile15 | GO:0070574 | cadmium ion transmembrane transport | 1/95 | 4/17913 | 0.021047231 | 0.178440576 | 0.146595 |
| GO_BP_Profile15 | GO:0070662 | mast cell proliferation | 1/95 | 4/17913 | 0.021047231 | 0.178440576 | 0.146595 |
| GO_BP_Profile15 | GO:0071284 | cellular response to lead ion | 1/95 | 4/17913 | 0.021047231 | 0.178440576 | 0.146595 |
| GO_BP_Profile15 | GO:0072367 | regulation of lipid transport by regulation of transcription from RNA polymerase II promoter | 1/95 | 4/17913 | 0.021047231 | 0.178440576 | 0.146595 |
| GO_BP_Profile15 | GO:1900086 | positive regulation of peptidyl-tyrosine autophosphorylation | 1/95 | 4/17913 | 0.021047231 | 0.178440576 | 0.146595 |
| GO_BP_Profile15 | GO:1903348 | positive regulation of bicellular tight junction assembly | 1/95 | 4/17913 | 0.021047231 | 0.178440576 | 0.146595 |
| GO_BP_Profile15 | GO:1904636 | response to ionomycin | 1/95 | 4/17913 | 0.021047231 | 0.178440576 | 0.146595 |
| GO_BP_Profile15 | GO:1904637 | cellular response to ionomycin | 1/95 | 4/17913 | 0.021047231 | 0.178440576 | 0.146595 |
| GO_BP_Profile15 | GO:2000670 | positive regulation of dendritic cell apoptotic process | 1/95 | 4/17913 | 0.021047231 | 0.178440576 | 0.146595 |
| GO_BP_Profile15 | GO:0050870 | positive regulation of T cell activation | 4/95 | 198/17913 | 0.021141057 | 0.178584275 | 0.146713 |
| GO_BP_Profile15 | GO:0006757 | ATP generation from ADP | 3/95 | 111/17913 | 0.021232784 | 0.178709264 | 0.146816 |
| GO_BP_Profile15 | GO:0030218 | erythrocyte differentiation | 3/95 | 112/17913 | 0.02173519 | 0.181727063 | 0.149295 |
| GO_BP_Profile15 | GO:0003044 | regulation of systemic arterial blood pressure mediated by a chemical signal | 2/95 | 43/17913 | 0.021826023 | 0.181727063 | 0.149295 |
| GO_BP_Profile15 | GO:0035094 | response to nicotine | 2/95 | 43/17913 | 0.021826023 | 0.181727063 | 0.149295 |
| GO_BP_Profile15 | GO:0046879 | hormone secretion | 5/95 | 301/17913 | 0.022030825 | 0.182777166 | 0.150157 |
| GO_BP_Profile15 | GO:0042866 | pyruvate biosynthetic process | 3/95 | 114/17913 | 0.022759476 | 0.184156909 | 0.151291 |
| GO_BP_Profile15 | GO:0051193 | regulation of cofactor metabolic process | 3/95 | 114/17913 | 0.022759476 | 0.184156909 | 0.151291 |
| GO_BP_Profile15 | GO:0002548 | monocyte chemotaxis | 2/95 | 44/17913 | 0.022787505 | 0.184156909 | 0.151291 |
| GO_BP_Profile15 | GO:0045766 | positive regulation of angiogenesis | 4/95 | 203/17913 | 0.022918204 | 0.184156909 | 0.151291 |
| GO_BP_Profile15 | GO:0050863 | regulation of T cell activation | 5/95 | 307/17913 | 0.023743407 | 0.184156909 | 0.151291 |
| GO_BP_Profile15 | GO:0033628 | regulation of cell adhesion mediated by integrin | 2/95 | 45/17913 | 0.023766227 | 0.184156909 | 0.151291 |
| GO_BP_Profile15 | GO:0042551 | neuron maturation | 2/95 | 45/17913 | 0.023766227 | 0.184156909 | 0.151291 |
| GO_BP_Profile15 | GO:0002237 | response to molecule of bacterial origin | 5/95 | 309/17913 | 0.024332893 | 0.184156909 | 0.151291 |
| GO_BP_Profile15 | GO:0051188 | cofactor biosynthetic process | 5/95 | 310/17913 | 0.024631155 | 0.184156909 | 0.151291 |
| GO_BP_Profile15 | GO:0048806 | genitalia development | 2/95 | 46/17913 | 0.024761984 | 0.184156909 | 0.151291 |
| GO_BP_Profile15 | GO:0009914 | hormone transport | 5/95 | 311/17913 | 0.024931771 | 0.184156909 | 0.151291 |
| GO_BP_Profile15 | GO:1901342 | regulation of vasculature development | 6/95 | 425/17913 | 0.025544612 | 0.184156909 | 0.151291 |
| GO_BP_Profile15 | GO:0043124 | negative regulation of I-kappaB kinase/NF-kappaB signaling | 2/95 | 47/17913 | 0.025774569 | 0.184156909 | 0.151291 |
| GO_BP_Profile15 | GO:0048641 | regulation of skeletal muscle tissue development | 2/95 | 47/17913 | 0.025774569 | 0.184156909 | 0.151291 |
| GO_BP_Profile15 | GO:1900744 | regulation of p38MAPK cascade | 2/95 | 47/17913 | 0.025774569 | 0.184156909 | 0.151291 |
| GO_BP_Profile15 | GO:0046394 | carboxylic acid biosynthetic process | 6/95 | 426/17913 | 0.025802013 | 0.184156909 | 0.151291 |
| GO_BP_Profile15 | GO:0034404 | nucleobase-containing small molecule biosynthetic process | 4/95 | 211/17913 | 0.025946425 | 0.184156909 | 0.151291 |
| GO_BP_Profile15 | GO:0016053 | organic acid biosynthetic process | 6/95 | 427/17913 | 0.026061145 | 0.184156909 | 0.151291 |
| GO_BP_Profile15 | GO:0002513 | tolerance induction to self antigen | 1/95 | 5/17913 | 0.026240179 | 0.184156909 | 0.151291 |
| GO_BP_Profile15 | GO:0006710 | androgen catabolic process | 1/95 | 5/17913 | 0.026240179 | 0.184156909 | 0.151291 |
| GO_BP_Profile15 | GO:0006824 | cobalt ion transport | 1/95 | 5/17913 | 0.026240179 | 0.184156909 | 0.151291 |
| GO_BP_Profile15 | GO:0009128 | purine nucleoside monophosphate catabolic process | 1/95 | 5/17913 | 0.026240179 | 0.184156909 | 0.151291 |
| GO_BP_Profile15 | GO:0010716 | negative regulation of extracellular matrix disassembly | 1/95 | 5/17913 | 0.026240179 | 0.184156909 | 0.151291 |
| GO_BP_Profile15 | GO:0010750 | positive regulation of nitric oxide mediated signal transduction | 1/95 | 5/17913 | 0.026240179 | 0.184156909 | 0.151291 |
| GO_BP_Profile15 | GO:0010891 | negative regulation of sequestering of triglyceride | 1/95 | 5/17913 | 0.026240179 | 0.184156909 | 0.151291 |
| GO_BP_Profile15 | GO:0015838 | amino-acid betaine transport | 1/95 | 5/17913 | 0.026240179 | 0.184156909 | 0.151291 |
| GO_BP_Profile15 | GO:0019418 | sulfide oxidation | 1/95 | 5/17913 | 0.026240179 | 0.184156909 | 0.151291 |
| GO_BP_Profile15 | GO:0021999 | neural plate anterior/posterior regionalization | 1/95 | 5/17913 | 0.026240179 | 0.184156909 | 0.151291 |
| GO_BP_Profile15 | GO:0034136 | negative regulation of toll-like receptor 2 signaling pathway | 1/95 | 5/17913 | 0.026240179 | 0.184156909 | 0.151291 |
| GO_BP_Profile15 | GO:0035962 | response to interleukin-13 | 1/95 | 5/17913 | 0.026240179 | 0.184156909 | 0.151291 |
| GO_BP_Profile15 | GO:0038033 | positive regulation of endothelial cell chemotaxis by VEGF-activated vascular endothelial growth factor receptor signaling pathway | 1/95 | 5/17913 | 0.026240179 | 0.184156909 | 0.151291 |
| GO_BP_Profile15 | GO:0043435 | response to corticotropin-releasing hormone | 1/95 | 5/17913 | 0.026240179 | 0.184156909 | 0.151291 |
| GO_BP_Profile15 | GO:0044501 | modulation of signal transduction in other organism | 1/95 | 5/17913 | 0.026240179 | 0.184156909 | 0.151291 |
| GO_BP_Profile15 | GO:0051414 | response to cortisol | 1/95 | 5/17913 | 0.026240179 | 0.184156909 | 0.151291 |
| GO_BP_Profile15 | GO:0052250 | modulation of signal transduction in other organism involved in symbiotic interaction | 1/95 | 5/17913 | 0.026240179 | 0.184156909 | 0.151291 |
| GO_BP_Profile15 | GO:0062028 | regulation of stress granule assembly | 1/95 | 5/17913 | 0.026240179 | 0.184156909 | 0.151291 |
| GO_BP_Profile15 | GO:0070221 | sulfide oxidation, using sulfide:quinone oxidoreductase | 1/95 | 5/17913 | 0.026240179 | 0.184156909 | 0.151291 |
| GO_BP_Profile15 | GO:0071362 | cellular response to ether | 1/95 | 5/17913 | 0.026240179 | 0.184156909 | 0.151291 |
| GO_BP_Profile15 | GO:0071376 | cellular response to corticotropin-releasing hormone stimulus | 1/95 | 5/17913 | 0.026240179 | 0.184156909 | 0.151291 |
| GO_BP_Profile15 | GO:0090155 | negative regulation of sphingolipid biosynthetic process | 1/95 | 5/17913 | 0.026240179 | 0.184156909 | 0.151291 |
| GO_BP_Profile15 | GO:0090259 | regulation of retinal ganglion cell axon guidance | 1/95 | 5/17913 | 0.026240179 | 0.184156909 | 0.151291 |
| GO_BP_Profile15 | GO:1900019 | regulation of protein kinase C activity | 1/95 | 5/17913 | 0.026240179 | 0.184156909 | 0.151291 |
| GO_BP_Profile15 | GO:1900020 | positive regulation of protein kinase C activity | 1/95 | 5/17913 | 0.026240179 | 0.184156909 | 0.151291 |
| GO_BP_Profile15 | GO:1900060 | negative regulation of ceramide biosynthetic process | 1/95 | 5/17913 | 0.026240179 | 0.184156909 | 0.151291 |
| GO_BP_Profile15 | GO:1900084 | regulation of peptidyl-tyrosine autophosphorylation | 1/95 | 5/17913 | 0.026240179 | 0.184156909 | 0.151291 |
| GO_BP_Profile15 | GO:1901563 | response to camptothecin | 1/95 | 5/17913 | 0.026240179 | 0.184156909 | 0.151291 |
| GO_BP_Profile15 | GO:1901842 | negative regulation of high voltage-gated calcium channel activity | 1/95 | 5/17913 | 0.026240179 | 0.184156909 | 0.151291 |
| GO_BP_Profile15 | GO:1902714 | negative regulation of interferon-gamma secretion | 1/95 | 5/17913 | 0.026240179 | 0.184156909 | 0.151291 |
| GO_BP_Profile15 | GO:1903936 | cellular response to sodium arsenite | 1/95 | 5/17913 | 0.026240179 | 0.184156909 | 0.151291 |
| GO_BP_Profile15 | GO:1904428 | negative regulation of tubulin deacetylation | 1/95 | 5/17913 | 0.026240179 | 0.184156909 | 0.151291 |
| GO_BP_Profile15 | GO:2000630 | positive regulation of miRNA metabolic process | 1/95 | 5/17913 | 0.026240179 | 0.184156909 | 0.151291 |
| GO_BP_Profile15 | GO:0046718 | viral entry into host cell | 3/95 | 121/17913 | 0.026548905 | 0.185762367 | 0.15261 |
| GO_BP_Profile15 | GO:0044003 | modification by symbiont of host morphology or physiology | 2/95 | 48/17913 | 0.026803782 | 0.186422713 | 0.153152 |
| GO_BP_Profile15 | GO:1903727 | positive regulation of phospholipid metabolic process | 2/95 | 48/17913 | 0.026803782 | 0.186422713 | 0.153152 |
| GO_BP_Profile15 | GO:0032368 | regulation of lipid transport | 3/95 | 122/17913 | 0.027116184 | 0.187646183 | 0.154158 |
| GO_BP_Profile15 | GO:1903039 | positive regulation of leukocyte cell-cell adhesion | 4/95 | 214/17913 | 0.027141247 | 0.187646183 | 0.154158 |
| GO_BP_Profile15 | GO:0071496 | cellular response to external stimulus | 5/95 | 320/17913 | 0.027744169 | 0.190277078 | 0.156319 |
| GO_BP_Profile15 | GO:0045981 | positive regulation of nucleotide metabolic process | 2/95 | 49/17913 | 0.027849422 | 0.190277078 | 0.156319 |
| GO_BP_Profile15 | GO:0048260 | positive regulation of receptor-mediated endocytosis | 2/95 | 49/17913 | 0.027849422 | 0.190277078 | 0.156319 |
| GO_BP_Profile15 | GO:1900544 | positive regulation of purine nucleotide metabolic process | 2/95 | 49/17913 | 0.027849422 | 0.190277078 | 0.156319 |
| GO_BP_Profile15 | GO:0008202 | steroid metabolic process | 5/95 | 321/17913 | 0.028068629 | 0.191212392 | 0.157087 |
| GO_BP_Profile15 | GO:0019218 | regulation of steroid metabolic process | 3/95 | 124/17913 | 0.028270154 | 0.192022126 | 0.157753 |
| GO_BP_Profile15 | GO:0099024 | plasma membrane invagination | 2/95 | 50/17913 | 0.028911288 | 0.193516806 | 0.15898 |
| GO_BP_Profile15 | GO:2000179 | positive regulation of neural precursor cell proliferation | 2/95 | 50/17913 | 0.028911288 | 0.193516806 | 0.15898 |
| GO_BP_Profile15 | GO:0019058 | viral life cycle | 5/95 | 324/17913 | 0.029056509 | 0.193516806 | 0.15898 |
| GO_BP_Profile15 | GO:0009266 | response to temperature stimulus | 4/95 | 219/17913 | 0.029205051 | 0.193516806 | 0.15898 |
| GO_BP_Profile15 | GO:0046467 | membrane lipid biosynthetic process | 3/95 | 127/17913 | 0.030049556 | 0.193516806 | 0.15898 |
| GO_BP_Profile15 | GO:0051291 | protein heterooligomerization | 3/95 | 128/17913 | 0.030655584 | 0.193516806 | 0.15898 |
| GO_BP_Profile15 | GO:1900542 | regulation of purine nucleotide metabolic process | 3/95 | 128/17913 | 0.030655584 | 0.193516806 | 0.15898 |
| GO_BP_Profile15 | GO:1903322 | positive regulation of protein modification by small protein conjugation or removal | 3/95 | 128/17913 | 0.030655584 | 0.193516806 | 0.15898 |
| GO_BP_Profile15 | GO:0002886 | regulation of myeloid leukocyte mediated immunity | 2/95 | 52/17913 | 0.031082914 | 0.193516806 | 0.15898 |
| GO_BP_Profile15 | GO:0006165 | nucleoside diphosphate phosphorylation | 3/95 | 129/17913 | 0.031268047 | 0.193516806 | 0.15898 |
| GO_BP_Profile15 | GO:0043122 | regulation of I-kappaB kinase/NF-kappaB signaling | 4/95 | 224/17913 | 0.031359935 | 0.193516806 | 0.15898 |
| GO_BP_Profile15 | GO:0046434 | organophosphate catabolic process | 4/95 | 224/17913 | 0.031359935 | 0.193516806 | 0.15898 |
| GO_BP_Profile15 | GO:0002767 | immune response-inhibiting cell surface receptor signaling pathway | 1/95 | 6/17913 | 0.03140587 | 0.193516806 | 0.15898 |
| GO_BP_Profile15 | GO:0002885 | positive regulation of hypersensitivity | 1/95 | 6/17913 | 0.03140587 | 0.193516806 | 0.15898 |
| GO_BP_Profile15 | GO:0006531 | aspartate metabolic process | 1/95 | 6/17913 | 0.03140587 | 0.193516806 | 0.15898 |
| GO_BP_Profile15 | GO:0006686 | sphingomyelin biosynthetic process | 1/95 | 6/17913 | 0.03140587 | 0.193516806 | 0.15898 |
| GO_BP_Profile15 | GO:0006828 | manganese ion transport | 1/95 | 6/17913 | 0.03140587 | 0.193516806 | 0.15898 |
| GO_BP_Profile15 | GO:0010871 | negative regulation of receptor biosynthetic process | 1/95 | 6/17913 | 0.03140587 | 0.193516806 | 0.15898 |
| GO_BP_Profile15 | GO:0030091 | protein repair | 1/95 | 6/17913 | 0.03140587 | 0.193516806 | 0.15898 |
| GO_BP_Profile15 | GO:0034201 | response to oleic acid | 1/95 | 6/17913 | 0.03140587 | 0.193516806 | 0.15898 |
| GO_BP_Profile15 | GO:0035434 | copper ion transmembrane transport | 1/95 | 6/17913 | 0.03140587 | 0.193516806 | 0.15898 |
| GO_BP_Profile15 | GO:0035931 | mineralocorticoid secretion | 1/95 | 6/17913 | 0.03140587 | 0.193516806 | 0.15898 |
| GO_BP_Profile15 | GO:0035932 | aldosterone secretion | 1/95 | 6/17913 | 0.03140587 | 0.193516806 | 0.15898 |
| GO_BP_Profile15 | GO:0045917 | positive regulation of complement activation | 1/95 | 6/17913 | 0.03140587 | 0.193516806 | 0.15898 |
| GO_BP_Profile15 | GO:0060897 | neural plate regionalization | 1/95 | 6/17913 | 0.03140587 | 0.193516806 | 0.15898 |
| GO_BP_Profile15 | GO:0061419 | positive regulation of transcription from RNA polymerase II promoter in response to hypoxia | 1/95 | 6/17913 | 0.03140587 | 0.193516806 | 0.15898 |
| GO_BP_Profile15 | GO:0070120 | ciliary neurotrophic factor-mediated signaling pathway | 1/95 | 6/17913 | 0.03140587 | 0.193516806 | 0.15898 |
| GO_BP_Profile15 | GO:0071421 | manganese ion transmembrane transport | 1/95 | 6/17913 | 0.03140587 | 0.193516806 | 0.15898 |
| GO_BP_Profile15 | GO:0072679 | thymocyte migration | 1/95 | 6/17913 | 0.03140587 | 0.193516806 | 0.15898 |
| GO_BP_Profile15 | GO:0097475 | motor neuron migration | 1/95 | 6/17913 | 0.03140587 | 0.193516806 | 0.15898 |
| GO_BP_Profile15 | GO:1901727 | positive regulation of histone deacetylase activity | 1/95 | 6/17913 | 0.03140587 | 0.193516806 | 0.15898 |
| GO_BP_Profile15 | GO:1903935 | response to sodium arsenite | 1/95 | 6/17913 | 0.03140587 | 0.193516806 | 0.15898 |
| GO_BP_Profile15 | GO:2000259 | positive regulation of protein activation cascade | 1/95 | 6/17913 | 0.03140587 | 0.193516806 | 0.15898 |
| GO_BP_Profile15 | GO:2000855 | regulation of mineralocorticoid secretion | 1/95 | 6/17913 | 0.03140587 | 0.193516806 | 0.15898 |
| GO_BP_Profile15 | GO:2000858 | regulation of aldosterone secretion | 1/95 | 6/17913 | 0.03140587 | 0.193516806 | 0.15898 |
| GO_BP_Profile15 | GO:0038066 | p38MAPK cascade | 2/95 | 53/17913 | 0.032192282 | 0.196796505 | 0.161675 |
| GO_BP_Profile15 | GO:0043551 | regulation of phosphatidylinositol 3-kinase activity | 2/95 | 53/17913 | 0.032192282 | 0.196796505 | 0.161675 |
| GO_BP_Profile15 | GO:0050994 | regulation of lipid catabolic process | 2/95 | 53/17913 | 0.032192282 | 0.196796505 | 0.161675 |
| GO_BP_Profile15 | GO:0046939 | nucleotide phosphorylation | 3/95 | 131/17913 | 0.032512244 | 0.197711894 | 0.162427 |
| GO_BP_Profile15 | GO:0048813 | dendrite morphogenesis | 3/95 | 131/17913 | 0.032512244 | 0.197711894 | 0.162427 |
| GO_BP_Profile15 | GO:0006140 | regulation of nucleotide metabolic process | 3/95 | 132/17913 | 0.033143962 | 0.19974763 | 0.164099 |
| GO_BP_Profile15 | GO:0046513 | ceramide biosynthetic process | 2/95 | 54/17913 | 0.033317097 | 0.19974763 | 0.164099 |
| GO_BP_Profile15 | GO:0071385 | cellular response to glucocorticoid stimulus | 2/95 | 54/17913 | 0.033317097 | 0.19974763 | 0.164099 |
| GO_BP_Profile15 | GO:0030260 | entry into host cell | 3/95 | 134/17913 | 0.034426586 | 0.19974763 | 0.164099 |
| GO_BP_Profile15 | GO:0044409 | entry into host | 3/95 | 134/17913 | 0.034426586 | 0.19974763 | 0.164099 |
| GO_BP_Profile15 | GO:0051806 | entry into cell of other organism involved in symbiotic interaction | 3/95 | 134/17913 | 0.034426586 | 0.19974763 | 0.164099 |
| GO_BP_Profile15 | GO:0051828 | entry into other organism involved in symbiotic interaction | 3/95 | 134/17913 | 0.034426586 | 0.19974763 | 0.164099 |
| GO_BP_Profile15 | GO:0031663 | lipopolysaccharide-mediated signaling pathway | 2/95 | 55/17913 | 0.034457167 | 0.19974763 | 0.164099 |
| GO_BP_Profile15 | GO:0048008 | platelet-derived growth factor receptor signaling pathway | 2/95 | 55/17913 | 0.034457167 | 0.19974763 | 0.164099 |
| GO_BP_Profile15 | GO:0098586 | cellular response to virus | 2/95 | 55/17913 | 0.034457167 | 0.19974763 | 0.164099 |
| GO_BP_Profile15 | GO:1904018 | positive regulation of vasculature development | 4/95 | 232/17913 | 0.034998446 | 0.19974763 | 0.164099 |
| GO_BP_Profile15 | GO:0000052 | citrulline metabolic process | 1/95 | 7/17913 | 0.036544444 | 0.19974763 | 0.164099 |
| GO_BP_Profile15 | GO:0002349 | histamine production involved in inflammatory response | 1/95 | 7/17913 | 0.036544444 | 0.19974763 | 0.164099 |
| GO_BP_Profile15 | GO:0002441 | histamine secretion involved in inflammatory response | 1/95 | 7/17913 | 0.036544444 | 0.19974763 | 0.164099 |
| GO_BP_Profile15 | GO:0002553 | histamine secretion by mast cell | 1/95 | 7/17913 | 0.036544444 | 0.19974763 | 0.164099 |
| GO_BP_Profile15 | GO:0009125 | nucleoside monophosphate catabolic process | 1/95 | 7/17913 | 0.036544444 | 0.19974763 | 0.164099 |
| GO_BP_Profile15 | GO:0010046 | response to mycotoxin | 1/95 | 7/17913 | 0.036544444 | 0.19974763 | 0.164099 |
| GO_BP_Profile15 | GO:0010760 | negative regulation of macrophage chemotaxis | 1/95 | 7/17913 | 0.036544444 | 0.19974763 | 0.164099 |
| GO_BP_Profile15 | GO:0010887 | negative regulation of cholesterol storage | 1/95 | 7/17913 | 0.036544444 | 0.19974763 | 0.164099 |
| GO_BP_Profile15 | GO:0014719 | skeletal muscle satellite cell activation | 1/95 | 7/17913 | 0.036544444 | 0.19974763 | 0.164099 |
| GO_BP_Profile15 | GO:0031077 | post-embryonic camera-type eye development | 1/95 | 7/17913 | 0.036544444 | 0.19974763 | 0.164099 |
| GO_BP_Profile15 | GO:0032261 | purine nucleotide salvage | 1/95 | 7/17913 | 0.036544444 | 0.19974763 | 0.164099 |
| GO_BP_Profile15 | GO:0032344 | regulation of aldosterone metabolic process | 1/95 | 7/17913 | 0.036544444 | 0.19974763 | 0.164099 |
| GO_BP_Profile15 | GO:0032347 | regulation of aldosterone biosynthetic process | 1/95 | 7/17913 | 0.036544444 | 0.19974763 | 0.164099 |
| GO_BP_Profile15 | GO:0032914 | positive regulation of transforming growth factor beta1 production | 1/95 | 7/17913 | 0.036544444 | 0.19974763 | 0.164099 |
| GO_BP_Profile15 | GO:0035095 | behavioral response to nicotine | 1/95 | 7/17913 | 0.036544444 | 0.19974763 | 0.164099 |
| GO_BP_Profile15 | GO:0038026 | reelin-mediated signaling pathway | 1/95 | 7/17913 | 0.036544444 | 0.19974763 | 0.164099 |
| GO_BP_Profile15 | GO:0038089 | positive regulation of cell migration by vascular endothelial growth factor signaling pathway | 1/95 | 7/17913 | 0.036544444 | 0.19974763 | 0.164099 |
| GO_BP_Profile15 | GO:0043652 | engulfment of apoptotic cell | 1/95 | 7/17913 | 0.036544444 | 0.19974763 | 0.164099 |
| GO_BP_Profile15 | GO:0043654 | recognition of apoptotic cell | 1/95 | 7/17913 | 0.036544444 | 0.19974763 | 0.164099 |
| GO_BP_Profile15 | GO:0048069 | eye pigmentation | 1/95 | 7/17913 | 0.036544444 | 0.19974763 | 0.164099 |
| GO_BP_Profile15 | GO:0048842 | positive regulation of axon extension involved in axon guidance | 1/95 | 7/17913 | 0.036544444 | 0.19974763 | 0.164099 |
| GO_BP_Profile15 | GO:0051694 | pointed-end actin filament capping | 1/95 | 7/17913 | 0.036544444 | 0.19974763 | 0.164099 |
| GO_BP_Profile15 | GO:0060369 | positive regulation of Fc receptor mediated stimulatory signaling pathway | 1/95 | 7/17913 | 0.036544444 | 0.19974763 | 0.164099 |
| GO_BP_Profile15 | GO:0060449 | bud elongation involved in lung branching | 1/95 | 7/17913 | 0.036544444 | 0.19974763 | 0.164099 |
| GO_BP_Profile15 | GO:0061370 | testosterone biosynthetic process | 1/95 | 7/17913 | 0.036544444 | 0.19974763 | 0.164099 |
| GO_BP_Profile15 | GO:0061762 | CAMKK-AMPK signaling cascade | 1/95 | 7/17913 | 0.036544444 | 0.19974763 | 0.164099 |
| GO_BP_Profile15 | GO:0070813 | hydrogen sulfide metabolic process | 1/95 | 7/17913 | 0.036544444 | 0.19974763 | 0.164099 |
| GO_BP_Profile15 | GO:1902075 | cellular response to salt | 1/95 | 7/17913 | 0.036544444 | 0.19974763 | 0.164099 |
| GO_BP_Profile15 | GO:1902961 | positive regulation of aspartic-type endopeptidase activity involved in amyloid precursor protein catabolic process | 1/95 | 7/17913 | 0.036544444 | 0.19974763 | 0.164099 |
| GO_BP_Profile15 | GO:1905097 | regulation of guanyl-nucleotide exchange factor activity | 1/95 | 7/17913 | 0.036544444 | 0.19974763 | 0.164099 |
| GO_BP_Profile15 | GO:2000427 | positive regulation of apoptotic cell clearance | 1/95 | 7/17913 | 0.036544444 | 0.19974763 | 0.164099 |
| GO_BP_Profile15 | GO:2000848 | positive regulation of corticosteroid hormone secretion | 1/95 | 7/17913 | 0.036544444 | 0.19974763 | 0.164099 |
| GO_BP_Profile15 | GO:0006879 | cellular iron ion homeostasis | 2/95 | 57/17913 | 0.036782314 | 0.20057586 | 0.16478 |
| GO_BP_Profile15 | GO:0006520 | cellular amino acid metabolic process | 5/95 | 347/17913 | 0.03736482 | 0.203275124 | 0.166997 |
| GO_BP_Profile15 | GO:0002831 | regulation of response to biotic stimulus | 3/95 | 139/17913 | 0.037744593 | 0.204634294 | 0.168114 |
| GO_BP_Profile15 | GO:0010324 | membrane invagination | 2/95 | 58/17913 | 0.037967017 | 0.204634294 | 0.168114 |
| GO_BP_Profile15 | GO:0032370 | positive regulation of lipid transport | 2/95 | 58/17913 | 0.037967017 | 0.204634294 | 0.168114 |
| GO_BP_Profile15 | GO:0071384 | cellular response to corticosteroid stimulus | 2/95 | 58/17913 | 0.037967017 | 0.204634294 | 0.168114 |
| GO_BP_Profile15 | GO:0045807 | positive regulation of endocytosis | 3/95 | 141/17913 | 0.039116129 | 0.209906693 | 0.172445 |
| GO_BP_Profile15 | GO:0045071 | negative regulation of viral genome replication | 2/95 | 59/17913 | 0.039166225 | 0.209906693 | 0.172445 |
| GO_BP_Profile15 | GO:0048872 | homeostasis of number of cells | 4/95 | 241/17913 | 0.039373912 | 0.209906693 | 0.172445 |
| GO_BP_Profile15 | GO:1901617 | organic hydroxy compound biosynthetic process | 4/95 | 241/17913 | 0.039373912 | 0.209906693 | 0.172445 |
| GO_BP_Profile15 | GO:0048871 | multicellular organismal homeostasis | 6/95 | 472/17913 | 0.039577681 | 0.209906693 | 0.172445 |
| GO_BP_Profile15 | GO:0019359 | nicotinamide nucleotide biosynthetic process | 3/95 | 142/17913 | 0.039811347 | 0.209906693 | 0.172445 |
| GO_BP_Profile15 | GO:0019363 | pyridine nucleotide biosynthetic process | 3/95 | 142/17913 | 0.039811347 | 0.209906693 | 0.172445 |
| GO_BP_Profile15 | GO:0009247 | glycolipid biosynthetic process | 2/95 | 60/17913 | 0.040379755 | 0.209906693 | 0.172445 |
| GO_BP_Profile15 | GO:0071383 | cellular response to steroid hormone stimulus | 4/95 | 243/17913 | 0.040386934 | 0.209906693 | 0.172445 |
| GO_BP_Profile15 | GO:0043467 | regulation of generation of precursor metabolites and energy | 3/95 | 144/17913 | 0.04122061 | 0.209906693 | 0.172445 |
| GO_BP_Profile15 | GO:0033627 | cell adhesion mediated by integrin | 2/95 | 61/17913 | 0.041607424 | 0.209906693 | 0.172445 |
| GO_BP_Profile15 | GO:0002765 | immune response-inhibiting signal transduction | 1/95 | 8/17913 | 0.041656042 | 0.209906693 | 0.172445 |
| GO_BP_Profile15 | GO:0002866 | positive regulation of acute inflammatory response to antigenic stimulus | 1/95 | 8/17913 | 0.041656042 | 0.209906693 | 0.172445 |
| GO_BP_Profile15 | GO:0002883 | regulation of hypersensitivity | 1/95 | 8/17913 | 0.041656042 | 0.209906693 | 0.172445 |
| GO_BP_Profile15 | GO:0006182 | cGMP biosynthetic process | 1/95 | 8/17913 | 0.041656042 | 0.209906693 | 0.172445 |
| GO_BP_Profile15 | GO:0006548 | histidine catabolic process | 1/95 | 8/17913 | 0.041656042 | 0.209906693 | 0.172445 |
| GO_BP_Profile15 | GO:0031666 | positive regulation of lipopolysaccharide-mediated signaling pathway | 1/95 | 8/17913 | 0.041656042 | 0.209906693 | 0.172445 |
| GO_BP_Profile15 | GO:0046476 | glycosylceramide biosynthetic process | 1/95 | 8/17913 | 0.041656042 | 0.209906693 | 0.172445 |
| GO_BP_Profile15 | GO:0052805 | imidazole-containing compound catabolic process | 1/95 | 8/17913 | 0.041656042 | 0.209906693 | 0.172445 |
| GO_BP_Profile15 | GO:0055118 | negative regulation of cardiac muscle contraction | 1/95 | 8/17913 | 0.041656042 | 0.209906693 | 0.172445 |
| GO_BP_Profile15 | GO:0060075 | regulation of resting membrane potential | 1/95 | 8/17913 | 0.041656042 | 0.209906693 | 0.172445 |
| GO_BP_Profile15 | GO:0060215 | primitive hemopoiesis | 1/95 | 8/17913 | 0.041656042 | 0.209906693 | 0.172445 |
| GO_BP_Profile15 | GO:0060896 | neural plate pattern specification | 1/95 | 8/17913 | 0.041656042 | 0.209906693 | 0.172445 |
| GO_BP_Profile15 | GO:0070444 | oligodendrocyte progenitor proliferation | 1/95 | 8/17913 | 0.041656042 | 0.209906693 | 0.172445 |
| GO_BP_Profile15 | GO:0070445 | regulation of oligodendrocyte progenitor proliferation | 1/95 | 8/17913 | 0.041656042 | 0.209906693 | 0.172445 |
| GO_BP_Profile15 | GO:0071499 | cellular response to laminar fluid shear stress | 1/95 | 8/17913 | 0.041656042 | 0.209906693 | 0.172445 |
| GO_BP_Profile15 | GO:0090045 | positive regulation of deacetylase activity | 1/95 | 8/17913 | 0.041656042 | 0.209906693 | 0.172445 |
| GO_BP_Profile15 | GO:0099004 | calmodulin dependent kinase signaling pathway | 1/95 | 8/17913 | 0.041656042 | 0.209906693 | 0.172445 |
| GO_BP_Profile15 | GO:1902669 | positive regulation of axon guidance | 1/95 | 8/17913 | 0.041656042 | 0.209906693 | 0.172445 |
| GO_BP_Profile15 | GO:1905247 | positive regulation of aspartic-type peptidase activity | 1/95 | 8/17913 | 0.041656042 | 0.209906693 | 0.172445 |
| GO_BP_Profile15 | GO:0042133 | neurotransmitter metabolic process | 3/95 | 145/17913 | 0.041934628 | 0.210397712 | 0.172849 |
| GO_BP_Profile15 | GO:0072525 | pyridine-containing compound biosynthetic process | 3/95 | 145/17913 | 0.041934628 | 0.210397712 | 0.172849 |
| GO_BP_Profile15 | GO:0009165 | nucleotide biosynthetic process | 5/95 | 360/17913 | 0.042647005 | 0.213051598 | 0.175029 |
| GO_BP_Profile15 | GO:0019932 | second-messenger-mediated signaling | 5/95 | 360/17913 | 0.042647005 | 0.213051598 | 0.175029 |
| GO_BP_Profile15 | GO:0007585 | respiratory gaseous exchange | 2/95 | 62/17913 | 0.042849053 | 0.213144216 | 0.175105 |
| GO_BP_Profile15 | GO:0043550 | regulation of lipid kinase activity | 2/95 | 62/17913 | 0.042849053 | 0.213144216 | 0.175105 |
| GO_BP_Profile15 | GO:0002224 | toll-like receptor signaling pathway | 3/95 | 147/17913 | 0.04338136 | 0.214414682 | 0.176149 |
| GO_BP_Profile15 | GO:0006090 | pyruvate metabolic process | 3/95 | 147/17913 | 0.04338136 | 0.214414682 | 0.176149 |
| GO_BP_Profile15 | GO:1903900 | regulation of viral life cycle | 3/95 | 147/17913 | 0.04338136 | 0.214414682 | 0.176149 |
| GO_BP_Profile15 | GO:0045600 | positive regulation of fat cell differentiation | 2/95 | 63/17913 | 0.044104461 | 0.217157778 | 0.178402 |
| GO_BP_Profile15 | GO:0022409 | positive regulation of cell-cell adhesion | 4/95 | 251/17913 | 0.044587183 | 0.217157778 | 0.178402 |
| GO_BP_Profile15 | GO:0031668 | cellular response to extracellular stimulus | 4/95 | 251/17913 | 0.044587183 | 0.217157778 | 0.178402 |
| GO_BP_Profile15 | GO:1901293 | nucleoside phosphate biosynthetic process | 5/95 | 365/17913 | 0.04479312 | 0.217157778 | 0.178402 |
| GO_BP_Profile15 | GO:0016202 | regulation of striated muscle tissue development | 3/95 | 149/17913 | 0.044852919 | 0.217157778 | 0.178402 |
| GO_BP_Profile15 | GO:0006754 | ATP biosynthetic process | 3/95 | 150/17913 | 0.045597967 | 0.217157778 | 0.178402 |
| GO_BP_Profile15 | GO:0090316 | positive regulation of intracellular protein transport | 3/95 | 150/17913 | 0.045597967 | 0.217157778 | 0.178402 |
| GO_BP_Profile15 | GO:0007249 | I-kappaB kinase/NF-kappaB signaling | 4/95 | 253/17913 | 0.04567428 | 0.217157778 | 0.178402 |
| GO_BP_Profile15 | GO:0046883 | regulation of hormone secretion | 4/95 | 254/17913 | 0.04622338 | 0.217157778 | 0.178402 |
| GO_BP_Profile15 | GO:0035924 | cellular response to vascular endothelial growth factor stimulus | 2/95 | 65/17913 | 0.046655908 | 0.217157778 | 0.178402 |
| GO_BP_Profile15 | GO:0045428 | regulation of nitric oxide biosynthetic process | 2/95 | 65/17913 | 0.046655908 | 0.217157778 | 0.178402 |
| GO_BP_Profile15 | GO:0002524 | hypersensitivity | 1/95 | 9/17913 | 0.046740805 | 0.217157778 | 0.178402 |
| GO_BP_Profile15 | GO:0002676 | regulation of chronic inflammatory response | 1/95 | 9/17913 | 0.046740805 | 0.217157778 | 0.178402 |
| GO_BP_Profile15 | GO:0006678 | glucosylceramide metabolic process | 1/95 | 9/17913 | 0.046740805 | 0.217157778 | 0.178402 |
| GO_BP_Profile15 | GO:0014816 | skeletal muscle satellite cell differentiation | 1/95 | 9/17913 | 0.046740805 | 0.217157778 | 0.178402 |
| GO_BP_Profile15 | GO:0034144 | negative regulation of toll-like receptor 4 signaling pathway | 1/95 | 9/17913 | 0.046740805 | 0.217157778 | 0.178402 |
| GO_BP_Profile15 | GO:0043129 | surfactant homeostasis | 1/95 | 9/17913 | 0.046740805 | 0.217157778 | 0.178402 |
| GO_BP_Profile15 | GO:0044828 | negative regulation by host of viral genome replication | 1/95 | 9/17913 | 0.046740805 | 0.217157778 | 0.178402 |
| GO_BP_Profile15 | GO:0045542 | positive regulation of cholesterol biosynthetic process | 1/95 | 9/17913 | 0.046740805 | 0.217157778 | 0.178402 |
| GO_BP_Profile15 | GO:0048845 | venous blood vessel morphogenesis | 1/95 | 9/17913 | 0.046740805 | 0.217157778 | 0.178402 |
| GO_BP_Profile15 | GO:0060539 | diaphragm development | 1/95 | 9/17913 | 0.046740805 | 0.217157778 | 0.178402 |
| GO_BP_Profile15 | GO:0060736 | prostate gland growth | 1/95 | 9/17913 | 0.046740805 | 0.217157778 | 0.178402 |
| GO_BP_Profile15 | GO:0060982 | coronary artery morphogenesis | 1/95 | 9/17913 | 0.046740805 | 0.217157778 | 0.178402 |
| GO_BP_Profile15 | GO:0070424 | regulation of nucleotide-binding oligomerization domain containing signaling pathway | 1/95 | 9/17913 | 0.046740805 | 0.217157778 | 0.178402 |
| GO_BP_Profile15 | GO:0090043 | regulation of tubulin deacetylation | 1/95 | 9/17913 | 0.046740805 | 0.217157778 | 0.178402 |
| GO_BP_Profile15 | GO:0097084 | vascular smooth muscle cell development | 1/95 | 9/17913 | 0.046740805 | 0.217157778 | 0.178402 |
| GO_BP_Profile15 | GO:0106120 | positive regulation of sterol biosynthetic process | 1/95 | 9/17913 | 0.046740805 | 0.217157778 | 0.178402 |
| GO_BP_Profile15 | GO:1902959 | regulation of aspartic-type endopeptidase activity involved in amyloid precursor protein catabolic process | 1/95 | 9/17913 | 0.046740805 | 0.217157778 | 0.178402 |
| GO_BP_Profile15 | GO:2000320 | negative regulation of T-helper 17 cell differentiation | 1/95 | 9/17913 | 0.046740805 | 0.217157778 | 0.178402 |
| GO_BP_Profile15 | GO:2000833 | positive regulation of steroid hormone secretion | 1/95 | 9/17913 | 0.046740805 | 0.217157778 | 0.178402 |
| GO_BP_Profile15 | GO:0001764 | neuron migration | 3/95 | 152/17913 | 0.047106518 | 0.217984943 | 0.179082 |
| GO_BP_Profile15 | GO:1901861 | regulation of muscle tissue development | 3/95 | 152/17913 | 0.047106518 | 0.217984943 | 0.179082 |
| GO_BP_Profile15 | GO:0048634 | regulation of muscle organ development | 3/95 | 153/17913 | 0.047869986 | 0.220638846 | 0.181262 |
| GO_BP_Profile15 | GO:0071346 | cellular response to interferon-gamma | 3/95 | 153/17913 | 0.047869986 | 0.220638846 | 0.181262 |
| GO_BP_Profile15 | GO:0051251 | positive regulation of lymphocyte activation | 4/95 | 258/17913 | 0.04845676 | 0.221192606 | 0.181717 |
| GO_BP_Profile15 | GO:0035051 | cardiocyte differentiation | 3/95 | 154/17913 | 0.048639559 | 0.221192606 | 0.181717 |
| GO_BP_Profile15 | GO:0009991 | response to extracellular stimulus | 6/95 | 498/17913 | 0.049113722 | 0.221192606 | 0.181717 |
| GO_BP_Profile15 | GO:0016125 | sterol metabolic process | 3/95 | 155/17913 | 0.049415219 | 0.221192606 | 0.181717 |
| GO_BP_Profile15 | GO:0014706 | striated muscle tissue development | 5/95 | 376/17913 | 0.049740129 | 0.221192606 | 0.181717 |
| GO_BP_Profile15 | GO:0009150 | purine ribonucleotide metabolic process | 6/95 | 500/17913 | 0.049901355 | 0.221192606 | 0.181717 |
| GO_BP_Profile3 | GO:0048285 | organelle fission | 28/105 | 395/17913 | 8.27114E-23 | 8.25634E-20 | 6.23E-20 |
| GO_BP_Profile3 | GO:0000280 | nuclear division | 27/105 | 357/17913 | 9.79981E-23 | 8.25634E-20 | 6.23E-20 |
| GO_BP_Profile3 | GO:0007059 | chromosome segregation | 24/105 | 275/17913 | 1.06219E-21 | 5.96595E-19 | 4.5E-19 |
| GO_BP_Profile3 | GO:0140014 | mitotic nuclear division | 22/105 | 237/17913 | 1.63489E-20 | 6.88696E-18 | 5.2E-18 |
| GO_BP_Profile3 | GO:0000819 | sister chromatid segregation | 18/105 | 157/17913 | 1.43665E-18 | 4.84151E-16 | 3.65E-16 |
| GO_BP_Profile3 | GO:0098813 | nuclear chromosome segregation | 19/105 | 220/17913 | 3.28085E-17 | 9.21371E-15 | 6.95E-15 |
| GO_BP_Profile3 | GO:0000070 | mitotic sister chromatid segregation | 16/105 | 132/17913 | 5.3579E-17 | 1.28972E-14 | 9.73E-15 |
| GO_BP_Profile3 | GO:0051983 | regulation of chromosome segregation | 12/105 | 93/17913 | 2.42526E-13 | 5.10819E-11 | 3.85E-11 |
| GO_BP_Profile3 | GO:0000226 | microtubule cytoskeleton organization | 20/105 | 460/17913 | 2.1769E-12 | 4.07564E-10 | 3.08E-10 |
| GO_BP_Profile3 | GO:0007088 | regulation of mitotic nuclear division | 13/105 | 150/17913 | 4.23926E-12 | 7.14315E-10 | 5.39E-10 |
| GO_BP_Profile3 | GO:0051783 | regulation of nuclear division | 13/105 | 172/17913 | 2.4242E-11 | 3.71343E-09 | 2.8E-09 |
| GO_BP_Profile3 | GO:0034502 | protein localization to chromosome | 10/105 | 82/17913 | 4.65908E-11 | 6.54213E-09 | 4.94E-09 |
| GO_BP_Profile3 | GO:0007051 | spindle organization | 12/105 | 147/17913 | 6.01511E-11 | 7.7965E-09 | 5.88E-09 |
| GO_BP_Profile3 | GO:0000281 | mitotic cytokinesis | 9/105 | 69/17913 | 2.43942E-10 | 2.93601E-08 | 2.22E-08 |
| GO_BP_Profile3 | GO:0033045 | regulation of sister chromatid segregation | 9/105 | 70/17913 | 2.78582E-10 | 3.12941E-08 | 2.36E-08 |
| GO_BP_Profile3 | GO:0006302 | double-strand break repair | 13/105 | 211/17913 | 3.12646E-10 | 3.29255E-08 | 2.48E-08 |
| GO_BP_Profile3 | GO:0045787 | positive regulation of cell cycle | 16/105 | 373/17913 | 5.26699E-10 | 5.22052E-08 | 3.94E-08 |
| GO_BP_Profile3 | GO:0000910 | cytokinesis | 11/105 | 147/17913 | 1.00375E-09 | 9.39626E-08 | 7.09E-08 |
| GO_BP_Profile3 | GO:0090068 | positive regulation of cell cycle process | 14/105 | 283/17913 | 1.11078E-09 | 9.8509E-08 | 7.43E-08 |
| GO_BP_Profile3 | GO:0061640 | cytoskeleton-dependent cytokinesis | 9/105 | 82/17913 | 1.18454E-09 | 9.97976E-08 | 7.53E-08 |
| GO_BP_Profile3 | GO:0071103 | DNA conformation change | 13/105 | 279/17913 | 9.3467E-09 | 7.49961E-07 | 5.66E-07 |
| GO_BP_Profile3 | GO:0000075 | cell cycle checkpoint | 11/105 | 197/17913 | 2.17908E-08 | 1.66898E-06 | 1.26E-06 |
| GO_BP_Profile3 | GO:1902850 | microtubule cytoskeleton organization involved in mitosis | 9/105 | 116/17913 | 2.61419E-08 | 1.91518E-06 | 1.45E-06 |
| GO_BP_Profile3 | GO:1901990 | regulation of mitotic cell cycle phase transition | 15/105 | 428/17913 | 2.89203E-08 | 2.03044E-06 | 1.53E-06 |
| GO_BP_Profile3 | GO:0033044 | regulation of chromosome organization | 13/105 | 311/17913 | 3.38525E-08 | 2.28166E-06 | 1.72E-06 |
| GO_BP_Profile3 | GO:0051302 | regulation of cell division | 10/105 | 162/17913 | 3.82103E-08 | 2.47632E-06 | 1.87E-06 |
| GO_BP_Profile3 | GO:0033047 | regulation of mitotic sister chromatid segregation | 7/105 | 57/17913 | 4.03662E-08 | 2.51915E-06 | 1.9E-06 |
| GO_BP_Profile3 | GO:0000082 | G1/S transition of mitotic cell cycle | 12/105 | 269/17913 | 5.81953E-08 | 3.50211E-06 | 2.64E-06 |
| GO_BP_Profile3 | GO:1901987 | regulation of cell cycle phase transition | 15/105 | 464/17913 | 8.33447E-08 | 4.7656E-06 | 3.6E-06 |
| GO_BP_Profile3 | GO:0007052 | mitotic spindle organization | 8/105 | 95/17913 | 8.48475E-08 | 4.7656E-06 | 3.6E-06 |
| GO_BP_Profile3 | GO:0071459 | protein localization to chromosome, centromeric region | 5/105 | 21/17913 | 1.18723E-07 | 6.44717E-06 | 4.87E-06 |
| GO_BP_Profile3 | GO:0044843 | cell cycle G1/S phase transition | 12/105 | 288/17913 | 1.22439E-07 | 6.44717E-06 | 4.87E-06 |
| GO_BP_Profile3 | GO:0007093 | mitotic cell cycle checkpoint | 9/105 | 147/17913 | 2.03087E-07 | 1.00769E-05 | 7.6E-06 |
| GO_BP_Profile3 | GO:0050000 | chromosome localization | 7/105 | 72/17913 | 2.09312E-07 | 1.00769E-05 | 7.6E-06 |
| GO_BP_Profile3 | GO:0051303 | establishment of chromosome localization | 7/105 | 72/17913 | 2.09312E-07 | 1.00769E-05 | 7.6E-06 |
| GO_BP_Profile3 | GO:0045931 | positive regulation of mitotic cell cycle | 9/105 | 154/17913 | 3.0188E-07 | 1.34056E-05 | 1.01E-05 |
| GO_BP_Profile3 | GO:0007098 | centrosome cycle | 8/105 | 112/17913 | 3.05794E-07 | 1.34056E-05 | 1.01E-05 |
| GO_BP_Profile3 | GO:0007091 | metaphase/anaphase transition of mitotic cell cycle | 6/105 | 47/17913 | 3.10277E-07 | 1.34056E-05 | 1.01E-05 |
| GO_BP_Profile3 | GO:0010965 | regulation of mitotic sister chromatid separation | 6/105 | 47/17913 | 3.10277E-07 | 1.34056E-05 | 1.01E-05 |
| GO_BP_Profile3 | GO:0006260 | DNA replication | 11/105 | 260/17913 | 3.64545E-07 | 1.53564E-05 | 1.16E-05 |
| GO_BP_Profile3 | GO:0044784 | metaphase/anaphase transition of cell cycle | 6/105 | 49/17913 | 4.00269E-07 | 1.60584E-05 | 1.21E-05 |
| GO_BP_Profile3 | GO:0051306 | mitotic sister chromatid separation | 6/105 | 49/17913 | 4.00269E-07 | 1.60584E-05 | 1.21E-05 |
| GO_BP_Profile3 | GO:0031145 | anaphase-promoting complex-dependent catabolic process | 7/105 | 81/17913 | 4.73224E-07 | 1.85438E-05 | 1.4E-05 |
| GO_BP_Profile3 | GO:0007077 | mitotic nuclear envelope disassembly | 4/105 | 12/17913 | 5.32151E-07 | 2.03789E-05 | 1.54E-05 |
| GO_BP_Profile3 | GO:0032465 | regulation of cytokinesis | 7/105 | 83/17913 | 5.59659E-07 | 2.09561E-05 | 1.58E-05 |
| GO_BP_Profile3 | GO:1905818 | regulation of chromosome separation | 6/105 | 52/17913 | 5.74511E-07 | 2.10446E-05 | 1.59E-05 |
| GO_BP_Profile3 | GO:0031023 | microtubule organizing center organization | 8/105 | 122/17913 | 5.90105E-07 | 2.11559E-05 | 1.6E-05 |
| GO_BP_Profile3 | GO:0051310 | metaphase plate congression | 6/105 | 55/17913 | 8.06534E-07 | 2.83127E-05 | 2.14E-05 |
| GO_BP_Profile3 | GO:0051225 | spindle assembly | 7/105 | 90/17913 | 9.73905E-07 | 3.34904E-05 | 2.53E-05 |
| GO_BP_Profile3 | GO:0030397 | membrane disassembly | 4/105 | 16/17913 | 1.92157E-06 | 6.34872E-05 | 4.79E-05 |
| GO_BP_Profile3 | GO:0051081 | nuclear envelope disassembly | 4/105 | 16/17913 | 1.92157E-06 | 6.34872E-05 | 4.79E-05 |
| GO_BP_Profile3 | GO:0006323 | DNA packaging | 9/105 | 194/17913 | 2.08934E-06 | 6.77025E-05 | 5.11E-05 |
| GO_BP_Profile3 | GO:0044839 | cell cycle G2/M phase transition | 10/105 | 257/17913 | 2.70793E-06 | 8.60916E-05 | 6.5E-05 |
| GO_BP_Profile3 | GO:0051304 | chromosome separation | 6/105 | 68/17913 | 2.86332E-06 | 8.92647E-05 | 6.74E-05 |
| GO_BP_Profile3 | GO:1902749 | regulation of cell cycle G2/M phase transition | 9/105 | 202/17913 | 2.91369E-06 | 8.92647E-05 | 6.74E-05 |
| GO_BP_Profile3 | GO:0000724 | double-strand break repair via homologous recombination | 7/105 | 108/17913 | 3.33455E-06 | 0.000100334 | 7.57E-05 |
| GO_BP_Profile3 | GO:0000725 | recombinational repair | 7/105 | 109/17913 | 3.54638E-06 | 0.000104836 | 7.91E-05 |
| GO_BP_Profile3 | GO:0032508 | DNA duplex unwinding | 6/105 | 76/17913 | 5.50659E-06 | 0.000159976 | 0.000121 |
| GO_BP_Profile3 | GO:0070507 | regulation of microtubule cytoskeleton organization | 8/105 | 170/17913 | 7.11853E-06 | 0.0002033 | 0.000153 |
| GO_BP_Profile3 | GO:0045840 | positive regulation of mitotic nuclear division | 5/105 | 48/17913 | 8.8109E-06 | 0.00024744 | 0.000187 |
| GO_BP_Profile3 | GO:0007062 | sister chromatid cohesion | 5/105 | 50/17913 | 1.08015E-05 | 0.00029837 | 0.000225 |
| GO_BP_Profile3 | GO:0000086 | G2/M transition of mitotic cell cycle | 9/105 | 238/17913 | 1.10132E-05 | 0.00029931 | 0.000226 |
| GO_BP_Profile3 | GO:0032392 | DNA geometric change | 6/105 | 86/17913 | 1.12951E-05 | 0.0003021 | 0.000228 |
| GO_BP_Profile3 | GO:0010389 | regulation of G2/M transition of mitotic cell cycle | 8/105 | 185/17913 | 1.32062E-05 | 0.000347694 | 0.000262 |
| GO_BP_Profile3 | GO:0034508 | centromere complex assembly | 5/105 | 54/17913 | 1.58258E-05 | 0.000409087 | 0.000309 |
| GO_BP_Profile3 | GO:0000022 | mitotic spindle elongation | 3/105 | 9/17913 | 1.60236E-05 | 0.000409087 | 0.000309 |
| GO_BP_Profile3 | GO:0031570 | DNA integrity checkpoint | 7/105 | 141/17913 | 1.92999E-05 | 0.000485378 | 0.000366 |
| GO_BP_Profile3 | GO:0140013 | meiotic nuclear division | 7/105 | 142/17913 | 2.02038E-05 | 0.000492951 | 0.000372 |
| GO_BP_Profile3 | GO:0010458 | exit from mitosis | 4/105 | 28/17913 | 2.04787E-05 | 0.000492951 | 0.000372 |
| GO_BP_Profile3 | GO:0071168 | protein localization to chromatin | 4/105 | 28/17913 | 2.04787E-05 | 0.000492951 | 0.000372 |
| GO_BP_Profile3 | GO:0032886 | regulation of microtubule-based process | 8/105 | 199/17913 | 2.23724E-05 | 0.000530951 | 0.000401 |
| GO_BP_Profile3 | GO:0051231 | spindle elongation | 3/105 | 10/17913 | 2.27932E-05 | 0.000533424 | 0.000403 |
| GO_BP_Profile3 | GO:0000083 | regulation of transcription involved in G1/S transition of mitotic cell cycle | 4/105 | 29/17913 | 2.36485E-05 | 0.000538482 | 0.000406 |
| GO_BP_Profile3 | GO:0008608 | attachment of spindle microtubules to kinetochore | 4/105 | 29/17913 | 2.36485E-05 | 0.000538482 | 0.000406 |
| GO_BP_Profile3 | GO:0051321 | meiotic cell cycle | 8/105 | 203/17913 | 2.58067E-05 | 0.00057979 | 0.000438 |
| GO_BP_Profile3 | GO:0051785 | positive regulation of nuclear division | 5/105 | 60/17913 | 2.65797E-05 | 0.0005893 | 0.000445 |
| GO_BP_Profile3 | GO:1903046 | meiotic cell cycle process | 7/105 | 151/17913 | 3.00276E-05 | 0.000657098 | 0.000496 |
| GO_BP_Profile3 | GO:0030261 | chromosome condensation | 4/105 | 32/17913 | 3.53242E-05 | 0.000763092 | 0.000576 |
| GO_BP_Profile3 | GO:1904668 | positive regulation of ubiquitin protein ligase activity | 3/105 | 12/17913 | 4.1432E-05 | 0.000883707 | 0.000667 |
| GO_BP_Profile3 | GO:0032506 | cytokinetic process | 4/105 | 37/17913 | 6.34331E-05 | 0.001336059 | 0.001008 |
| GO_BP_Profile3 | GO:2000779 | regulation of double-strand break repair | 5/105 | 72/17913 | 6.44063E-05 | 0.00133981 | 0.001011 |
| GO_BP_Profile3 | GO:0045930 | negative regulation of mitotic cell cycle | 9/105 | 308/17913 | 8.28036E-05 | 0.001701512 | 0.001284 |
| GO_BP_Profile3 | GO:0006310 | DNA recombination | 8/105 | 244/17913 | 9.45243E-05 | 0.001893747 | 0.001429 |
| GO_BP_Profile3 | GO:0007080 | mitotic metaphase plate congression | 4/105 | 41/17913 | 9.55303E-05 | 0.001893747 | 0.001429 |
| GO_BP_Profile3 | GO:0051653 | spindle localization | 4/105 | 41/17913 | 9.55303E-05 | 0.001893747 | 0.001429 |
| GO_BP_Profile3 | GO:0007096 | regulation of exit from mitosis | 3/105 | 16/17913 | 0.000103677 | 0.001990048 | 0.001502 |
| GO_BP_Profile3 | GO:0034501 | protein localization to kinetochore | 3/105 | 16/17913 | 0.000103677 | 0.001990048 | 0.001502 |
| GO_BP_Profile3 | GO:0034080 | CENP-A containing nucleosome assembly | 4/105 | 42/17913 | 0.000105112 | 0.001990048 | 0.001502 |
| GO_BP_Profile3 | GO:0061641 | CENP-A containing chromatin organization | 4/105 | 42/17913 | 0.000105112 | 0.001990048 | 0.001502 |
| GO_BP_Profile3 | GO:0030071 | regulation of mitotic metaphase/anaphase transition | 4/105 | 44/17913 | 0.000126341 | 0.002365386 | 0.001785 |
| GO_BP_Profile3 | GO:0000018 | regulation of DNA recombination | 5/105 | 84/17913 | 0.000134425 | 0.002489084 | 0.001878 |
| GO_BP_Profile3 | GO:0006998 | nuclear envelope organization | 4/105 | 45/17913 | 0.000138045 | 0.002528326 | 0.001908 |
| GO_BP_Profile3 | GO:0031055 | chromatin remodeling at centromere | 4/105 | 46/17913 | 0.000150514 | 0.002698048 | 0.002036 |
| GO_BP_Profile3 | GO:1902099 | regulation of metaphase/anaphase transition of cell cycle | 4/105 | 46/17913 | 0.000150514 | 0.002698048 | 0.002036 |
| GO_BP_Profile3 | GO:0072698 | protein localization to microtubule cytoskeleton | 4/105 | 47/17913 | 0.000163778 | 0.002904903 | 0.002192 |
| GO_BP_Profile3 | GO:0051383 | kinetochore organization | 3/105 | 20/17913 | 0.000207487 | 0.003624219 | 0.002735 |
| GO_BP_Profile3 | GO:0044380 | protein localization to cytoskeleton | 4/105 | 50/17913 | 0.000208635 | 0.003624219 | 0.002735 |
| GO_BP_Profile3 | GO:0007063 | regulation of sister chromatid cohesion | 3/105 | 21/17913 | 0.000241039 | 0.004144388 | 0.003128 |
| GO_BP_Profile3 | GO:0006336 | DNA replication-independent nucleosome assembly | 4/105 | 53/17913 | 0.000261728 | 0.004410115 | 0.003328 |
| GO_BP_Profile3 | GO:0034724 | DNA replication-independent nucleosome organization | 4/105 | 53/17913 | 0.000261728 | 0.004410115 | 0.003328 |
| GO_BP_Profile3 | GO:1904666 | regulation of ubiquitin protein ligase activity | 3/105 | 22/17913 | 0.00027791 | 0.004636426 | 0.003499 |
| GO_BP_Profile3 | GO:0046605 | regulation of centrosome cycle | 4/105 | 54/17913 | 0.0002814 | 0.004648618 | 0.003508 |
| GO_BP_Profile3 | GO:0051656 | establishment of organelle localization | 10/105 | 448/17913 | 0.000300242 | 0.004894789 | 0.003694 |
| GO_BP_Profile3 | GO:0043486 | histone exchange | 4/105 | 55/17913 | 0.000302112 | 0.004894789 | 0.003694 |
| GO_BP_Profile3 | GO:0000729 | DNA double-strand break processing | 3/105 | 23/17913 | 0.000318238 | 0.005106966 | 0.003854 |
| GO_BP_Profile3 | GO:0031536 | positive regulation of exit from mitosis | 2/105 | 5/17913 | 0.00033644 | 0.00534813 | 0.004036 |
| GO_BP_Profile3 | GO:0000132 | establishment of mitotic spindle orientation | 3/105 | 24/17913 | 0.000362155 | 0.005650287 | 0.004264 |
| GO_BP_Profile3 | GO:1902410 | mitotic cytokinetic process | 3/105 | 24/17913 | 0.000362155 | 0.005650287 | 0.004264 |
| GO_BP_Profile3 | GO:0010498 | proteasomal protein catabolic process | 9/105 | 382/17913 | 0.000411665 | 0.006363817 | 0.004802 |
| GO_BP_Profile3 | GO:1901991 | negative regulation of mitotic cell cycle phase transition | 7/105 | 235/17913 | 0.000468034 | 0.007169429 | 0.00541 |
| GO_BP_Profile3 | GO:0010994 | free ubiquitin chain polymerization | 2/105 | 6/17913 | 0.000502728 | 0.007496432 | 0.005657 |
| GO_BP_Profile3 | GO:0090306 | spindle assembly involved in meiosis | 2/105 | 6/17913 | 0.000502728 | 0.007496432 | 0.005657 |
| GO_BP_Profile3 | GO:1902412 | regulation of mitotic cytokinesis | 2/105 | 6/17913 | 0.000502728 | 0.007496432 | 0.005657 |
| GO_BP_Profile3 | GO:0006282 | regulation of DNA repair | 5/105 | 112/17913 | 0.000513203 | 0.007585505 | 0.005724 |
| GO_BP_Profile3 | GO:0040001 | establishment of mitotic spindle localization | 3/105 | 28/17913 | 0.000576279 | 0.008299404 | 0.006263 |
| GO_BP_Profile3 | GO:0051294 | establishment of spindle orientation | 3/105 | 28/17913 | 0.000576279 | 0.008299404 | 0.006263 |
| GO_BP_Profile3 | GO:0051443 | positive regulation of ubiquitin-protein transferase activity | 3/105 | 28/17913 | 0.000576279 | 0.008299404 | 0.006263 |
| GO_BP_Profile3 | GO:0051052 | regulation of DNA metabolic process | 9/105 | 403/17913 | 0.000605201 | 0.008642067 | 0.006522 |
| GO_BP_Profile3 | GO:0006333 | chromatin assembly or disassembly | 6/105 | 178/17913 | 0.000631609 | 0.008943375 | 0.006749 |
| GO_BP_Profile3 | GO:0009262 | deoxyribonucleotide metabolic process | 3/105 | 29/17913 | 0.000640044 | 0.008987286 | 0.006782 |
| GO_BP_Profile3 | GO:0043161 | proteasome-mediated ubiquitin-dependent protein catabolic process | 8/105 | 328/17913 | 0.000693836 | 0.009108543 | 0.006874 |
| GO_BP_Profile3 | GO:0010992 | ubiquitin recycling | 2/105 | 7/17913 | 0.000701126 | 0.009108543 | 0.006874 |
| GO_BP_Profile3 | GO:0044806 | G-quadruplex DNA unwinding | 2/105 | 7/17913 | 0.000701126 | 0.009108543 | 0.006874 |
| GO_BP_Profile3 | GO:0051256 | mitotic spindle midzone assembly | 2/105 | 7/17913 | 0.000701126 | 0.009108543 | 0.006874 |
| GO_BP_Profile3 | GO:0007094 | mitotic spindle assembly checkpoint | 3/105 | 30/17913 | 0.000708142 | 0.009108543 | 0.006874 |
| GO_BP_Profile3 | GO:0007143 | female meiotic nuclear division | 3/105 | 30/17913 | 0.000708142 | 0.009108543 | 0.006874 |
| GO_BP_Profile3 | GO:0031572 | G2 DNA damage checkpoint | 3/105 | 30/17913 | 0.000708142 | 0.009108543 | 0.006874 |
| GO_BP_Profile3 | GO:0031577 | spindle checkpoint | 3/105 | 30/17913 | 0.000708142 | 0.009108543 | 0.006874 |
| GO_BP_Profile3 | GO:0071173 | spindle assembly checkpoint | 3/105 | 30/17913 | 0.000708142 | 0.009108543 | 0.006874 |
| GO_BP_Profile3 | GO:0071174 | mitotic spindle checkpoint | 3/105 | 30/17913 | 0.000708142 | 0.009108543 | 0.006874 |
| GO_BP_Profile3 | GO:0071539 | protein localization to centrosome | 3/105 | 30/17913 | 0.000708142 | 0.009108543 | 0.006874 |
| GO_BP_Profile3 | GO:1901988 | negative regulation of cell cycle phase transition | 7/105 | 254/17913 | 0.000741874 | 0.009470139 | 0.007147 |
| GO_BP_Profile3 | GO:1905508 | protein localization to microtubule organizing center | 3/105 | 31/17913 | 0.000780688 | 0.009890667 | 0.007464 |
| GO_BP_Profile3 | GO:2001251 | negative regulation of chromosome organization | 5/105 | 125/17913 | 0.00084496 | 0.01062506 | 0.008018 |
| GO_BP_Profile3 | GO:0045841 | negative regulation of mitotic metaphase/anaphase transition | 3/105 | 32/17913 | 0.000857794 | 0.010627819 | 0.00802 |
| GO_BP_Profile3 | GO:2000816 | negative regulation of mitotic sister chromatid separation | 3/105 | 32/17913 | 0.000857794 | 0.010627819 | 0.00802 |
| GO_BP_Profile3 | GO:0072401 | signal transduction involved in DNA integrity checkpoint | 4/105 | 73/17913 | 0.000889423 | 0.010859989 | 0.008195 |
| GO_BP_Profile3 | GO:0072422 | signal transduction involved in DNA damage checkpoint | 4/105 | 73/17913 | 0.000889423 | 0.010859989 | 0.008195 |
| GO_BP_Profile3 | GO:0010825 | positive regulation of centrosome duplication | 2/105 | 8/17913 | 0.000931258 | 0.011051226 | 0.00834 |
| GO_BP_Profile3 | GO:0085020 | protein K6-linked ubiquitination | 2/105 | 8/17913 | 0.000931258 | 0.011051226 | 0.00834 |
| GO_BP_Profile3 | GO:0072395 | signal transduction involved in cell cycle checkpoint | 4/105 | 74/17913 | 0.000936049 | 0.011051226 | 0.00834 |
| GO_BP_Profile3 | GO:1902100 | negative regulation of metaphase/anaphase transition of cell cycle | 3/105 | 33/17913 | 0.000939572 | 0.011051226 | 0.00834 |
| GO_BP_Profile3 | GO:1905819 | negative regulation of chromosome separation | 3/105 | 33/17913 | 0.000939572 | 0.011051226 | 0.00834 |
| GO_BP_Profile3 | GO:0010948 | negative regulation of cell cycle process | 8/105 | 344/17913 | 0.000944437 | 0.011051226 | 0.00834 |
| GO_BP_Profile3 | GO:0006890 | retrograde vesicle-mediated transport, Golgi to ER | 4/105 | 76/17913 | 0.00103442 | 0.012020672 | 0.009071 |
| GO_BP_Profile3 | GO:0007292 | female gamete generation | 5/105 | 132/17913 | 0.001079021 | 0.012451015 | 0.009396 |
| GO_BP_Profile3 | GO:0043044 | ATP-dependent chromatin remodeling | 4/105 | 77/17913 | 0.001086231 | 0.012451015 | 0.009396 |
| GO_BP_Profile3 | GO:0000077 | DNA damage checkpoint | 5/105 | 133/17913 | 0.001116016 | 0.012638311 | 0.009537 |
| GO_BP_Profile3 | GO:0033048 | negative regulation of mitotic sister chromatid segregation | 3/105 | 35/17913 | 0.001117572 | 0.012638311 | 0.009537 |
| GO_BP_Profile3 | GO:0051293 | establishment of spindle localization | 3/105 | 36/17913 | 0.001214003 | 0.013637297 | 0.010291 |
| GO_BP_Profile3 | GO:0032467 | positive regulation of cytokinesis | 3/105 | 37/17913 | 0.001315524 | 0.014583271 | 0.011005 |
| GO_BP_Profile3 | GO:0033046 | negative regulation of sister chromatid segregation | 3/105 | 37/17913 | 0.001315524 | 0.014583271 | 0.011005 |
| GO_BP_Profile3 | GO:1901992 | positive regulation of mitotic cell cycle phase transition | 4/105 | 82/17913 | 0.001372881 | 0.01511964 | 0.01141 |
| GO_BP_Profile3 | GO:0006284 | base-excision repair | 3/105 | 38/17913 | 0.001422233 | 0.015361942 | 0.011593 |
| GO_BP_Profile3 | GO:0016572 | histone phosphorylation | 3/105 | 38/17913 | 0.001422233 | 0.015361942 | 0.011593 |
| GO_BP_Profile3 | GO:0051985 | negative regulation of chromosome segregation | 3/105 | 38/17913 | 0.001422233 | 0.015361942 | 0.011593 |
| GO_BP_Profile3 | GO:0000079 | regulation of cyclin-dependent protein serine/threonine kinase activity | 4/105 | 83/17913 | 0.001435963 | 0.015411453 | 0.01163 |
| GO_BP_Profile3 | GO:0009263 | deoxyribonucleotide biosynthetic process | 2/105 | 10/17913 | 0.001485244 | 0.015544326 | 0.01173 |
| GO_BP_Profile3 | GO:0046607 | positive regulation of centrosome cycle | 2/105 | 10/17913 | 0.001485244 | 0.015544326 | 0.01173 |
| GO_BP_Profile3 | GO:0051255 | spindle midzone assembly | 2/105 | 10/17913 | 0.001485244 | 0.015544326 | 0.01173 |
| GO_BP_Profile3 | GO:0051315 | attachment of mitotic spindle microtubules to kinetochore | 2/105 | 10/17913 | 0.001485244 | 0.015544326 | 0.01173 |
| GO_BP_Profile3 | GO:0044773 | mitotic DNA damage checkpoint | 4/105 | 86/17913 | 0.001637311 | 0.017030054 | 0.012852 |
| GO_BP_Profile3 | GO:0010824 | regulation of centrosome duplication | 3/105 | 40/17913 | 0.001651604 | 0.017073328 | 0.012884 |
| GO_BP_Profile3 | GO:1904029 | regulation of cyclin-dependent protein kinase activity | 4/105 | 87/17913 | 0.001708571 | 0.017554521 | 0.013247 |
| GO_BP_Profile3 | GO:0010569 | regulation of double-strand break repair via homologous recombination | 3/105 | 41/17913 | 0.001774452 | 0.017979628 | 0.013568 |
| GO_BP_Profile3 | GO:0090224 | regulation of spindle organization | 3/105 | 41/17913 | 0.001774452 | 0.017979628 | 0.013568 |
| GO_BP_Profile3 | GO:0007127 | meiosis I | 4/105 | 88/17913 | 0.001781957 | 0.017979628 | 0.013568 |
| GO_BP_Profile3 | GO:0000076 | DNA replication checkpoint | 2/105 | 11/17913 | 0.001808364 | 0.018137456 | 0.013687 |
| GO_BP_Profile3 | GO:0051493 | regulation of cytoskeleton organization | 9/105 | 472/17913 | 0.001823886 | 0.018184901 | 0.013723 |
| GO_BP_Profile3 | GO:0044774 | mitotic DNA integrity checkpoint | 4/105 | 90/17913 | 0.001935242 | 0.019181663 | 0.014475 |
| GO_BP_Profile3 | GO:0061982 | meiosis I cell cycle process | 4/105 | 91/17913 | 0.002015205 | 0.019857433 | 0.014985 |
| GO_BP_Profile3 | GO:0031497 | chromatin assembly | 5/105 | 153/17913 | 0.002071367 | 0.020292171 | 0.015313 |
| GO_BP_Profile3 | GO:0000212 | meiotic spindle organization | 2/105 | 12/17913 | 0.00216175 | 0.021055194 | 0.015889 |
| GO_BP_Profile3 | GO:0006338 | chromatin remodeling | 5/105 | 156/17913 | 0.002254194 | 0.021829407 | 0.016473 |
| GO_BP_Profile3 | GO:0031109 | microtubule polymerization or depolymerization | 4/105 | 94/17913 | 0.00226877 | 0.02184501 | 0.016485 |
| GO_BP_Profile3 | GO:0045839 | negative regulation of mitotic nuclear division | 3/105 | 45/17913 | 0.002322343 | 0.022233793 | 0.016779 |
| GO_BP_Profile3 | GO:0097711 | ciliary basal body-plasma membrane docking | 4/105 | 95/17913 | 0.002357957 | 0.022447214 | 0.01694 |
| GO_BP_Profile3 | GO:0007050 | cell cycle arrest | 6/105 | 231/17913 | 0.002399734 | 0.022716586 | 0.017143 |
| GO_BP_Profile3 | GO:0019886 | antigen processing and presentation of exogenous peptide antigen via MHC class II | 4/105 | 97/17913 | 0.002543521 | 0.023562633 | 0.017781 |
| GO_BP_Profile3 | GO:1901989 | positive regulation of cell cycle phase transition | 4/105 | 97/17913 | 0.002543521 | 0.023562633 | 0.017781 |
| GO_BP_Profile3 | GO:1902750 | negative regulation of cell cycle G2/M phase transition | 4/105 | 97/17913 | 0.002543521 | 0.023562633 | 0.017781 |
| GO_BP_Profile3 | GO:0062033 | positive regulation of mitotic sister chromatid segregation | 2/105 | 13/17913 | 0.002545044 | 0.023562633 | 0.017781 |
| GO_BP_Profile3 | GO:0051438 | regulation of ubiquitin-protein transferase activity | 3/105 | 47/17913 | 0.002631372 | 0.02422875 | 0.018284 |
| GO_BP_Profile3 | GO:0002495 | antigen processing and presentation of peptide antigen via MHC class II | 4/105 | 100/17913 | 0.00284032 | 0.026010543 | 0.019629 |
| GO_BP_Profile3 | GO:0034728 | nucleosome organization | 5/105 | 165/17913 | 0.002873312 | 0.026170433 | 0.019749 |
| GO_BP_Profile3 | GO:0071824 | protein-DNA complex subunit organization | 6/105 | 240/17913 | 0.00290094 | 0.026280023 | 0.019832 |
| GO_BP_Profile3 | GO:0002504 | antigen processing and presentation of peptide or polysaccharide antigen via MHC class II | 4/105 | 101/17913 | 0.0029443 | 0.026389074 | 0.019914 |
| GO_BP_Profile3 | GO:0018107 | peptidyl-threonine phosphorylation | 4/105 | 101/17913 | 0.0029443 | 0.026389074 | 0.019914 |
| GO_BP_Profile3 | GO:0006275 | regulation of DNA replication | 4/105 | 102/17913 | 0.003050856 | 0.027199426 | 0.020526 |
| GO_BP_Profile3 | GO:0001325 | formation of extrachromosomal circular DNA | 2/105 | 15/17913 | 0.003399935 | 0.029530363 | 0.022285 |
| GO_BP_Profile3 | GO:0007076 | mitotic chromosome condensation | 2/105 | 15/17913 | 0.003399935 | 0.029530363 | 0.022285 |
| GO_BP_Profile3 | GO:0009200 | deoxyribonucleoside triphosphate metabolic process | 2/105 | 15/17913 | 0.003399935 | 0.029530363 | 0.022285 |
| GO_BP_Profile3 | GO:0090656 | t-circle formation | 2/105 | 15/17913 | 0.003399935 | 0.029530363 | 0.022285 |
| GO_BP_Profile3 | GO:0090737 | telomere maintenance via telomere trimming | 2/105 | 15/17913 | 0.003399935 | 0.029530363 | 0.022285 |
| GO_BP_Profile3 | GO:0007163 | establishment or maintenance of cell polarity | 5/105 | 172/17913 | 0.003433566 | 0.029669529 | 0.02239 |
| GO_BP_Profile3 | GO:0051784 | negative regulation of nuclear division | 3/105 | 52/17913 | 0.00351131 | 0.030033285 | 0.022665 |
| GO_BP_Profile3 | GO:0090307 | mitotic spindle assembly | 3/105 | 52/17913 | 0.00351131 | 0.030033285 | 0.022665 |
| GO_BP_Profile3 | GO:0051382 | kinetochore assembly | 2/105 | 16/17913 | 0.003870829 | 0.032780749 | 0.024738 |
| GO_BP_Profile3 | GO:0018210 | peptidyl-threonine modification | 4/105 | 109/17913 | 0.003871436 | 0.032780749 | 0.024738 |
| GO_BP_Profile3 | GO:0006977 | DNA damage response, signal transduction by p53 class mediator resulting in cell cycle arrest | 3/105 | 56/17913 | 0.0043304 | 0.036483623 | 0.027532 |
| GO_BP_Profile3 | GO:0061952 | midbody abscission | 2/105 | 17/17913 | 0.004370225 | 0.036635966 | 0.027647 |
| GO_BP_Profile3 | GO:0072431 | signal transduction involved in mitotic G1 DNA damage checkpoint | 3/105 | 57/17913 | 0.004551713 | 0.037781457 | 0.028512 |
| GO_BP_Profile3 | GO:1902400 | intracellular signal transduction involved in G1 DNA damage checkpoint | 3/105 | 57/17913 | 0.004551713 | 0.037781457 | 0.028512 |
| GO_BP_Profile3 | GO:0072331 | signal transduction by p53 class mediator | 6/105 | 264/17913 | 0.004620401 | 0.03816361 | 0.0288 |
| GO_BP_Profile3 | GO:1902115 | regulation of organelle assembly | 5/105 | 185/17913 | 0.00467615 | 0.038435668 | 0.029005 |
| GO_BP_Profile3 | GO:0051782 | negative regulation of cell division | 2/105 | 18/17913 | 0.00489778 | 0.040061935 | 0.030233 |
| GO_BP_Profile3 | GO:0051298 | centrosome duplication | 3/105 | 59/17913 | 0.005014608 | 0.040236256 | 0.030364 |
| GO_BP_Profile3 | GO:0072413 | signal transduction involved in mitotic cell cycle checkpoint | 3/105 | 59/17913 | 0.005014608 | 0.040236256 | 0.030364 |
| GO_BP_Profile3 | GO:1902402 | signal transduction involved in mitotic DNA damage checkpoint | 3/105 | 59/17913 | 0.005014608 | 0.040236256 | 0.030364 |
| GO_BP_Profile3 | GO:1902403 | signal transduction involved in mitotic DNA integrity checkpoint | 3/105 | 59/17913 | 0.005014608 | 0.040236256 | 0.030364 |
| GO_BP_Profile3 | GO:0006997 | nucleus organization | 4/105 | 118/17913 | 0.005130742 | 0.040972991 | 0.03092 |
| GO_BP_Profile3 | GO:0007095 | mitotic G2 DNA damage checkpoint | 2/105 | 19/17913 | 0.005453152 | 0.043138781 | 0.032555 |
| GO_BP_Profile3 | GO:0046599 | regulation of centriole replication | 2/105 | 19/17913 | 0.005453152 | 0.043138781 | 0.032555 |
| GO_BP_Profile3 | GO:0000741 | karyogamy | 1/105 | 1/17913 | 0.005861665 | 0.04370312 | 0.03298 |
| GO_BP_Profile3 | GO:0000921 | septin ring assembly | 1/105 | 1/17913 | 0.005861665 | 0.04370312 | 0.03298 |
| GO_BP_Profile3 | GO:0007344 | pronuclear fusion | 1/105 | 1/17913 | 0.005861665 | 0.04370312 | 0.03298 |
| GO_BP_Profile3 | GO:0019088 | immortalization of host cell by virus | 1/105 | 1/17913 | 0.005861665 | 0.04370312 | 0.03298 |
| GO_BP_Profile3 | GO:0019357 | nicotinate nucleotide biosynthetic process | 1/105 | 1/17913 | 0.005861665 | 0.04370312 | 0.03298 |
| GO_BP_Profile3 | GO:0019358 | nicotinate nucleotide salvage | 1/105 | 1/17913 | 0.005861665 | 0.04370312 | 0.03298 |
| GO_BP_Profile3 | GO:0019365 | pyridine nucleotide salvage | 1/105 | 1/17913 | 0.005861665 | 0.04370312 | 0.03298 |
| GO_BP_Profile3 | GO:0020021 | immortalization of host cell | 1/105 | 1/17913 | 0.005861665 | 0.04370312 | 0.03298 |
| GO_BP_Profile3 | GO:0021685 | cerebellar granular layer structural organization | 1/105 | 1/17913 | 0.005861665 | 0.04370312 | 0.03298 |
| GO_BP_Profile3 | GO:0031106 | septin ring organization | 1/105 | 1/17913 | 0.005861665 | 0.04370312 | 0.03298 |
| GO_BP_Profile3 | GO:0045769 | negative regulation of asymmetric cell division | 1/105 | 1/17913 | 0.005861665 | 0.04370312 | 0.03298 |
| GO_BP_Profile3 | GO:0046497 | nicotinate nucleotide metabolic process | 1/105 | 1/17913 | 0.005861665 | 0.04370312 | 0.03298 |
| GO_BP_Profile3 | GO:0048621 | post-embryonic digestive tract morphogenesis | 1/105 | 1/17913 | 0.005861665 | 0.04370312 | 0.03298 |
| GO_BP_Profile3 | GO:0031571 | mitotic G1 DNA damage checkpoint | 3/105 | 63/17913 | 0.006023038 | 0.044512367 | 0.033591 |
| GO_BP_Profile3 | GO:0044819 | mitotic G1/S transition checkpoint | 3/105 | 63/17913 | 0.006023038 | 0.044512367 | 0.033591 |
| GO_BP_Profile3 | GO:0044783 | G1 DNA damage checkpoint | 3/105 | 64/17913 | 0.006292668 | 0.046301949 | 0.034942 |
| GO_BP_Profile3 | GO:0030010 | establishment of cell polarity | 4/105 | 126/17913 | 0.006458646 | 0.0473166 | 0.035707 |
| GO_BP_Profile3 | GO:2001020 | regulation of response to DNA damage stimulus | 5/105 | 201/17913 | 0.006608654 | 0.048205983 | 0.036378 |
| GO_BP_Profile3 | GO:1903429 | regulation of cell maturation | 2/105 | 22/17913 | 0.007282808 | 0.052894534 | 0.039917 |
| GO_BP_Profile3 | GO:0042770 | signal transduction in response to DNA damage | 4/105 | 131/17913 | 0.007394696 | 0.053476666 | 0.040356 |
| GO_BP_Profile3 | GO:0065004 | protein-DNA complex assembly | 5/105 | 210/17913 | 0.007912452 | 0.056733812 | 0.042814 |
| GO_BP_Profile3 | GO:0007064 | mitotic sister chromatid cohesion | 2/105 | 23/17913 | 0.007946101 | 0.056733812 | 0.042814 |
| GO_BP_Profile3 | GO:0044818 | mitotic G2/M transition checkpoint | 2/105 | 23/17913 | 0.007946101 | 0.056733812 | 0.042814 |
| GO_BP_Profile3 | GO:0006334 | nucleosome assembly | 4/105 | 135/17913 | 0.008204909 | 0.058334478 | 0.044022 |
| GO_BP_Profile3 | GO:0022411 | cellular component disassembly | 8/105 | 492/17913 | 0.008356558 | 0.059163025 | 0.044647 |
| GO_BP_Profile3 | GO:0051642 | centrosome localization | 2/105 | 24/17913 | 0.00863555 | 0.060628759 | 0.045753 |
| GO_BP_Profile3 | GO:0051984 | positive regulation of chromosome segregation | 2/105 | 24/17913 | 0.00863555 | 0.060628759 | 0.045753 |
| GO_BP_Profile3 | GO:1902117 | positive regulation of organelle assembly | 3/105 | 72/17913 | 0.00870927 | 0.060892616 | 0.045952 |
| GO_BP_Profile3 | GO:0061842 | microtubule organizing center localization | 2/105 | 25/17913 | 0.009350834 | 0.065091017 | 0.049121 |
| GO_BP_Profile3 | GO:1900182 | positive regulation of protein localization to nucleus | 3/105 | 74/17913 | 0.009387013 | 0.065091017 | 0.049121 |
| GO_BP_Profile3 | GO:0140056 | organelle localization by membrane tethering | 4/105 | 141/17913 | 0.009526125 | 0.065784924 | 0.049644 |
| GO_BP_Profile3 | GO:0031100 | animal organ regeneration | 3/105 | 75/17913 | 0.009737123 | 0.066967562 | 0.050537 |
| GO_BP_Profile3 | GO:0051054 | positive regulation of DNA metabolic process | 5/105 | 222/17913 | 0.009915933 | 0.067920109 | 0.051256 |
| GO_BP_Profile3 | GO:0031440 | regulation of mRNA 3'-end processing | 2/105 | 26/17913 | 0.01009163 | 0.068843713 | 0.051953 |
| GO_BP_Profile3 | GO:0007018 | microtubule-based movement | 5/105 | 224/17913 | 0.01028078 | 0.069851266 | 0.052713 |
| GO_BP_Profile3 | GO:0001578 | microtubule bundle formation | 3/105 | 78/17913 | 0.010832785 | 0.070596642 | 0.053275 |
| GO_BP_Profile3 | GO:0009394 | 2'-deoxyribonucleotide metabolic process | 2/105 | 27/17913 | 0.010857622 | 0.070596642 | 0.053275 |
| GO_BP_Profile3 | GO:0015949 | nucleobase-containing small molecule interconversion | 2/105 | 27/17913 | 0.010857622 | 0.070596642 | 0.053275 |
| GO_BP_Profile3 | GO:0090169 | regulation of spindle assembly | 2/105 | 27/17913 | 0.010857622 | 0.070596642 | 0.053275 |
| GO_BP_Profile3 | GO:0010165 | response to X-ray | 2/105 | 28/17913 | 0.011648495 | 0.070596642 | 0.053275 |
| GO_BP_Profile3 | GO:0070979 | protein K11-linked ubiquitination | 2/105 | 28/17913 | 0.011648495 | 0.070596642 | 0.053275 |
| GO_BP_Profile3 | GO:0003290 | atrial septum secundum morphogenesis | 1/105 | 2/17913 | 0.011689296 | 0.070596642 | 0.053275 |
| GO_BP_Profile3 | GO:0006231 | dTMP biosynthetic process | 1/105 | 2/17913 | 0.011689296 | 0.070596642 | 0.053275 |
| GO_BP_Profile3 | GO:0007057 | spindle assembly involved in female meiosis I | 1/105 | 2/17913 | 0.011689296 | 0.070596642 | 0.053275 |
| GO_BP_Profile3 | GO:0021693 | cerebellar Purkinje cell layer structural organization | 1/105 | 2/17913 | 0.011689296 | 0.070596642 | 0.053275 |
| GO_BP_Profile3 | GO:0021698 | cerebellar cortex structural organization | 1/105 | 2/17913 | 0.011689296 | 0.070596642 | 0.053275 |
| GO_BP_Profile3 | GO:0032185 | septin cytoskeleton organization | 1/105 | 2/17913 | 0.011689296 | 0.070596642 | 0.053275 |
| GO_BP_Profile3 | GO:0033567 | DNA replication, Okazaki fragment processing | 1/105 | 2/17913 | 0.011689296 | 0.070596642 | 0.053275 |
| GO_BP_Profile3 | GO:0033624 | negative regulation of integrin activation | 1/105 | 2/17913 | 0.011689296 | 0.070596642 | 0.053275 |
| GO_BP_Profile3 | GO:0035408 | histone H3-T6 phosphorylation | 1/105 | 2/17913 | 0.011689296 | 0.070596642 | 0.053275 |
| GO_BP_Profile3 | GO:0036298 | recombinational interstrand cross-link repair | 1/105 | 2/17913 | 0.011689296 | 0.070596642 | 0.053275 |
| GO_BP_Profile3 | GO:0043137 | DNA replication, removal of RNA primer | 1/105 | 2/17913 | 0.011689296 | 0.070596642 | 0.053275 |
| GO_BP_Profile3 | GO:0045870 | positive regulation of single stranded viral RNA replication via double stranded DNA intermediate | 1/105 | 2/17913 | 0.011689296 | 0.070596642 | 0.053275 |
| GO_BP_Profile3 | GO:0046073 | dTMP metabolic process | 1/105 | 2/17913 | 0.011689296 | 0.070596642 | 0.053275 |
| GO_BP_Profile3 | GO:0048627 | myoblast development | 1/105 | 2/17913 | 0.011689296 | 0.070596642 | 0.053275 |
| GO_BP_Profile3 | GO:0070105 | positive regulation of interleukin-6-mediated signaling pathway | 1/105 | 2/17913 | 0.011689296 | 0.070596642 | 0.053275 |
| GO_BP_Profile3 | GO:0070194 | synaptonemal complex disassembly | 1/105 | 2/17913 | 0.011689296 | 0.070596642 | 0.053275 |
| GO_BP_Profile3 | GO:0090402 | oncogene-induced cell senescence | 1/105 | 2/17913 | 0.011689296 | 0.070596642 | 0.053275 |
| GO_BP_Profile3 | GO:0097681 | double-strand break repair via alternative nonhomologous end joining | 1/105 | 2/17913 | 0.011689296 | 0.070596642 | 0.053275 |
| GO_BP_Profile3 | GO:1900195 | positive regulation of oocyte maturation | 1/105 | 2/17913 | 0.011689296 | 0.070596642 | 0.053275 |
| GO_BP_Profile3 | GO:1903436 | regulation of mitotic cytokinetic process | 1/105 | 2/17913 | 0.011689296 | 0.070596642 | 0.053275 |
| GO_BP_Profile3 | GO:1903438 | positive regulation of mitotic cytokinetic process | 1/105 | 2/17913 | 0.011689296 | 0.070596642 | 0.053275 |
| GO_BP_Profile3 | GO:1903490 | positive regulation of mitotic cytokinesis | 1/105 | 2/17913 | 0.011689296 | 0.070596642 | 0.053275 |
| GO_BP_Profile3 | GO:1904170 | regulation of bleb assembly | 1/105 | 2/17913 | 0.011689296 | 0.070596642 | 0.053275 |
| GO_BP_Profile3 | GO:1904172 | positive regulation of bleb assembly | 1/105 | 2/17913 | 0.011689296 | 0.070596642 | 0.053275 |
| GO_BP_Profile3 | GO:2000707 | positive regulation of dense core granule biogenesis | 1/105 | 2/17913 | 0.011689296 | 0.070596642 | 0.053275 |
| GO_BP_Profile3 | GO:0022406 | membrane docking | 4/105 | 150/17913 | 0.011755836 | 0.070744942 | 0.053387 |
| GO_BP_Profile3 | GO:0006303 | double-strand break repair via nonhomologous end joining | 3/105 | 81/17913 | 0.011997042 | 0.071939556 | 0.054289 |
| GO_BP_Profile3 | GO:0071158 | positive regulation of cell cycle arrest | 3/105 | 82/17913 | 0.012400491 | 0.074095131 | 0.055916 |
| GO_BP_Profile3 | GO:0019692 | deoxyribose phosphate metabolic process | 2/105 | 29/17913 | 0.012463936 | 0.074211067 | 0.056003 |
| GO_BP_Profile3 | GO:0051781 | positive regulation of cell division | 3/105 | 84/17913 | 0.013230572 | 0.078498291 | 0.059238 |
| GO_BP_Profile3 | GO:2000781 | positive regulation of double-strand break repair | 2/105 | 30/17913 | 0.013303635 | 0.078654827 | 0.059357 |
| GO_BP_Profile3 | GO:0010972 | negative regulation of G2/M transition of mitotic cell cycle | 3/105 | 85/17913 | 0.013657247 | 0.080463149 | 0.060721 |
| GO_BP_Profile3 | GO:0007099 | centriole replication | 2/105 | 31/17913 | 0.014167287 | 0.082888467 | 0.062551 |
| GO_BP_Profile3 | GO:0045910 | negative regulation of DNA recombination | 2/105 | 31/17913 | 0.014167287 | 0.082888467 | 0.062551 |
| GO_BP_Profile3 | GO:0097421 | liver regeneration | 2/105 | 32/17913 | 0.015054587 | 0.087775011 | 0.066239 |
| GO_BP_Profile3 | GO:0000726 | non-recombinational repair | 3/105 | 89/17913 | 0.015441929 | 0.089722934 | 0.067709 |
| GO_BP_Profile3 | GO:0007019 | microtubule depolymerization | 2/105 | 34/17913 | 0.016898927 | 0.092930612 | 0.07013 |
| GO_BP_Profile3 | GO:0003285 | septum secundum development | 1/105 | 3/17913 | 0.017483089 | 0.092930612 | 0.07013 |
| GO_BP_Profile3 | GO:0006203 | dGTP catabolic process | 1/105 | 3/17913 | 0.017483089 | 0.092930612 | 0.07013 |
| GO_BP_Profile3 | GO:0006235 | dTTP biosynthetic process | 1/105 | 3/17913 | 0.017483089 | 0.092930612 | 0.07013 |
| GO_BP_Profile3 | GO:0006273 | lagging strand elongation | 1/105 | 3/17913 | 0.017483089 | 0.092930612 | 0.07013 |
| GO_BP_Profile3 | GO:0007056 | spindle assembly involved in female meiosis | 1/105 | 3/17913 | 0.017483089 | 0.092930612 | 0.07013 |
| GO_BP_Profile3 | GO:0009177 | pyrimidine deoxyribonucleoside monophosphate biosynthetic process | 1/105 | 3/17913 | 0.017483089 | 0.092930612 | 0.07013 |
| GO_BP_Profile3 | GO:0009212 | pyrimidine deoxyribonucleoside triphosphate biosynthetic process | 1/105 | 3/17913 | 0.017483089 | 0.092930612 | 0.07013 |
| GO_BP_Profile3 | GO:0010956 | negative regulation of calcidiol 1-monooxygenase activity | 1/105 | 3/17913 | 0.017483089 | 0.092930612 | 0.07013 |
| GO_BP_Profile3 | GO:0014038 | regulation of Schwann cell differentiation | 1/105 | 3/17913 | 0.017483089 | 0.092930612 | 0.07013 |
| GO_BP_Profile3 | GO:0019087 | transformation of host cell by virus | 1/105 | 3/17913 | 0.017483089 | 0.092930612 | 0.07013 |
| GO_BP_Profile3 | GO:0019860 | uracil metabolic process | 1/105 | 3/17913 | 0.017483089 | 0.092930612 | 0.07013 |
| GO_BP_Profile3 | GO:0032954 | regulation of cytokinetic process | 1/105 | 3/17913 | 0.017483089 | 0.092930612 | 0.07013 |
| GO_BP_Profile3 | GO:0034421 | post-translational protein acetylation | 1/105 | 3/17913 | 0.017483089 | 0.092930612 | 0.07013 |
| GO_BP_Profile3 | GO:0035986 | senescence-associated heterochromatin focus assembly | 1/105 | 3/17913 | 0.017483089 | 0.092930612 | 0.07013 |
| GO_BP_Profile3 | GO:0042148 | strand invasion | 1/105 | 3/17913 | 0.017483089 | 0.092930612 | 0.07013 |
| GO_BP_Profile3 | GO:0046075 | dTTP metabolic process | 1/105 | 3/17913 | 0.017483089 | 0.092930612 | 0.07013 |
| GO_BP_Profile3 | GO:0046078 | dUMP metabolic process | 1/105 | 3/17913 | 0.017483089 | 0.092930612 | 0.07013 |
| GO_BP_Profile3 | GO:0051754 | meiotic sister chromatid cohesion, centromeric | 1/105 | 3/17913 | 0.017483089 | 0.092930612 | 0.07013 |
| GO_BP_Profile3 | GO:0060282 | positive regulation of oocyte development | 1/105 | 3/17913 | 0.017483089 | 0.092930612 | 0.07013 |
| GO_BP_Profile3 | GO:0061110 | dense core granule biogenesis | 1/105 | 3/17913 | 0.017483089 | 0.092930612 | 0.07013 |
| GO_BP_Profile3 | GO:0070995 | NADPH oxidation | 1/105 | 3/17913 | 0.017483089 | 0.092930612 | 0.07013 |
| GO_BP_Profile3 | GO:1902990 | mitotic telomere maintenance via semi-conservative replication | 1/105 | 3/17913 | 0.017483089 | 0.092930612 | 0.07013 |
| GO_BP_Profile3 | GO:1905463 | negative regulation of DNA duplex unwinding | 1/105 | 3/17913 | 0.017483089 | 0.092930612 | 0.07013 |
| GO_BP_Profile3 | GO:1905881 | positive regulation of oogenesis | 1/105 | 3/17913 | 0.017483089 | 0.092930612 | 0.07013 |
| GO_BP_Profile3 | GO:1990166 | protein localization to site of double-strand break | 1/105 | 3/17913 | 0.017483089 | 0.092930612 | 0.07013 |
| GO_BP_Profile3 | GO:2000705 | regulation of dense core granule biogenesis | 1/105 | 3/17913 | 0.017483089 | 0.092930612 | 0.07013 |
| GO_BP_Profile3 | GO:0045070 | positive regulation of viral genome replication | 2/105 | 35/17913 | 0.017855372 | 0.094019695 | 0.070952 |
| GO_BP_Profile3 | GO:0045740 | positive regulation of DNA replication | 2/105 | 35/17913 | 0.017855372 | 0.094019695 | 0.070952 |
| GO_BP_Profile3 | GO:0098534 | centriole assembly | 2/105 | 35/17913 | 0.017855372 | 0.094019695 | 0.070952 |
| GO_BP_Profile3 | GO:0045911 | positive regulation of DNA recombination | 2/105 | 36/17913 | 0.018834276 | 0.098558248 | 0.074377 |
| GO_BP_Profile3 | GO:0046825 | regulation of protein export from nucleus | 2/105 | 36/17913 | 0.018834276 | 0.098558248 | 0.074377 |
| GO_BP_Profile3 | GO:0002478 | antigen processing and presentation of exogenous peptide antigen | 4/105 | 174/17913 | 0.019256619 | 0.100456355 | 0.075809 |
| GO_BP_Profile3 | GO:0014037 | Schwann cell differentiation | 2/105 | 37/17913 | 0.019835348 | 0.102838651 | 0.077607 |
| GO_BP_Profile3 | GO:0060236 | regulation of mitotic spindle organization | 2/105 | 37/17913 | 0.019835348 | 0.102838651 | 0.077607 |
| GO_BP_Profile3 | GO:0019884 | antigen processing and presentation of exogenous antigen | 4/105 | 176/17913 | 0.01998883 | 0.1033165 | 0.077967 |
| GO_BP_Profile3 | GO:2000045 | regulation of G1/S transition of mitotic cell cycle | 4/105 | 177/17913 | 0.020361308 | 0.104919892 | 0.079177 |
| GO_BP_Profile3 | GO:1901796 | regulation of signal transduction by p53 class mediator | 4/105 | 179/17913 | 0.021119069 | 0.108492779 | 0.081874 |
| GO_BP_Profile3 | GO:0043902 | positive regulation of multi-organism process | 4/105 | 180/17913 | 0.021504375 | 0.11001364 | 0.083021 |
| GO_BP_Profile3 | GO:0071900 | regulation of protein serine/threonine kinase activity | 7/105 | 479/17913 | 0.022605283 | 0.11001364 | 0.083021 |
| GO_BP_Profile3 | GO:0000912 | assembly of actomyosin apparatus involved in cytokinesis | 1/105 | 4/17913 | 0.023243238 | 0.11001364 | 0.083021 |
| GO_BP_Profile3 | GO:0000915 | actomyosin contractile ring assembly | 1/105 | 4/17913 | 0.023243238 | 0.11001364 | 0.083021 |
| GO_BP_Profile3 | GO:0002159 | desmosome assembly | 1/105 | 4/17913 | 0.023243238 | 0.11001364 | 0.083021 |
| GO_BP_Profile3 | GO:0002317 | plasma cell differentiation | 1/105 | 4/17913 | 0.023243238 | 0.11001364 | 0.083021 |
| GO_BP_Profile3 | GO:0003431 | growth plate cartilage chondrocyte development | 1/105 | 4/17913 | 0.023243238 | 0.11001364 | 0.083021 |
| GO_BP_Profile3 | GO:0006015 | 5-phosphoribose 1-diphosphate biosynthetic process | 1/105 | 4/17913 | 0.023243238 | 0.11001364 | 0.083021 |
| GO_BP_Profile3 | GO:0007079 | mitotic chromosome movement towards spindle pole | 1/105 | 4/17913 | 0.023243238 | 0.11001364 | 0.083021 |
| GO_BP_Profile3 | GO:0009176 | pyrimidine deoxyribonucleoside monophosphate metabolic process | 1/105 | 4/17913 | 0.023243238 | 0.11001364 | 0.083021 |
| GO_BP_Profile3 | GO:0009211 | pyrimidine deoxyribonucleoside triphosphate metabolic process | 1/105 | 4/17913 | 0.023243238 | 0.11001364 | 0.083021 |
| GO_BP_Profile3 | GO:0009786 | regulation of asymmetric cell division | 1/105 | 4/17913 | 0.023243238 | 0.11001364 | 0.083021 |
| GO_BP_Profile3 | GO:0030951 | establishment or maintenance of microtubule cytoskeleton polarity | 1/105 | 4/17913 | 0.023243238 | 0.11001364 | 0.083021 |
| GO_BP_Profile3 | GO:0032487 | regulation of Rap protein signal transduction | 1/105 | 4/17913 | 0.023243238 | 0.11001364 | 0.083021 |
| GO_BP_Profile3 | GO:0032971 | regulation of muscle filament sliding | 1/105 | 4/17913 | 0.023243238 | 0.11001364 | 0.083021 |
| GO_BP_Profile3 | GO:0042997 | negative regulation of Golgi to plasma membrane protein transport | 1/105 | 4/17913 | 0.023243238 | 0.11001364 | 0.083021 |
| GO_BP_Profile3 | GO:0043504 | mitochondrial DNA repair | 1/105 | 4/17913 | 0.023243238 | 0.11001364 | 0.083021 |
| GO_BP_Profile3 | GO:0046070 | dGTP metabolic process | 1/105 | 4/17913 | 0.023243238 | 0.11001364 | 0.083021 |
| GO_BP_Profile3 | GO:0046391 | 5-phosphoribose 1-diphosphate metabolic process | 1/105 | 4/17913 | 0.023243238 | 0.11001364 | 0.083021 |
| GO_BP_Profile3 | GO:0060574 | intestinal epithelial cell maturation | 1/105 | 4/17913 | 0.023243238 | 0.11001364 | 0.083021 |
| GO_BP_Profile3 | GO:0061113 | pancreas morphogenesis | 1/105 | 4/17913 | 0.023243238 | 0.11001364 | 0.083021 |
| GO_BP_Profile3 | GO:0071922 | regulation of cohesin loading | 1/105 | 4/17913 | 0.023243238 | 0.11001364 | 0.083021 |
| GO_BP_Profile3 | GO:0072757 | cellular response to camptothecin | 1/105 | 4/17913 | 0.023243238 | 0.11001364 | 0.083021 |
| GO_BP_Profile3 | GO:0090521 | glomerular visceral epithelial cell migration | 1/105 | 4/17913 | 0.023243238 | 0.11001364 | 0.083021 |
| GO_BP_Profile3 | GO:1903553 | positive regulation of extracellular exosome assembly | 1/105 | 4/17913 | 0.023243238 | 0.11001364 | 0.083021 |
| GO_BP_Profile3 | GO:1903724 | positive regulation of centriole elongation | 1/105 | 4/17913 | 0.023243238 | 0.11001364 | 0.083021 |
| GO_BP_Profile3 | GO:1905340 | regulation of protein localization to kinetochore | 1/105 | 4/17913 | 0.023243238 | 0.11001364 | 0.083021 |
| GO_BP_Profile3 | GO:1905342 | positive regulation of protein localization to kinetochore | 1/105 | 4/17913 | 0.023243238 | 0.11001364 | 0.083021 |
| GO_BP_Profile3 | GO:0030330 | DNA damage response, signal transduction by p53 class mediator | 3/105 | 106/17913 | 0.024434318 | 0.115327242 | 0.087031 |
| GO_BP_Profile3 | GO:0048002 | antigen processing and presentation of peptide antigen | 4/105 | 188/17913 | 0.024742468 | 0.116455469 | 0.087883 |
| GO_BP_Profile3 | GO:0071156 | regulation of cell cycle arrest | 3/105 | 108/17913 | 0.025642881 | 0.120357256 | 0.090827 |
| GO_BP_Profile3 | GO:0031398 | positive regulation of protein ubiquitination | 3/105 | 110/17913 | 0.026883148 | 0.125828069 | 0.094956 |
| GO_BP_Profile3 | GO:0030225 | macrophage differentiation | 2/105 | 44/17913 | 0.027439645 | 0.127452596 | 0.096182 |
| GO_BP_Profile3 | GO:1902806 | regulation of cell cycle G1/S phase transition | 4/105 | 195/17913 | 0.027805455 | 0.127452596 | 0.096182 |
| GO_BP_Profile3 | GO:0021987 | cerebral cortex development | 3/105 | 112/17913 | 0.028155076 | 0.127452596 | 0.096182 |
| GO_BP_Profile3 | GO:0003289 | atrial septum primum morphogenesis | 1/105 | 5/17913 | 0.028969937 | 0.127452596 | 0.096182 |
| GO_BP_Profile3 | GO:0003418 | growth plate cartilage chondrocyte differentiation | 1/105 | 5/17913 | 0.028969937 | 0.127452596 | 0.096182 |
| GO_BP_Profile3 | GO:0009202 | deoxyribonucleoside triphosphate biosynthetic process | 1/105 | 5/17913 | 0.028969937 | 0.127452596 | 0.096182 |
| GO_BP_Profile3 | GO:0009217 | purine deoxyribonucleoside triphosphate catabolic process | 1/105 | 5/17913 | 0.028969937 | 0.127452596 | 0.096182 |
| GO_BP_Profile3 | GO:0009221 | pyrimidine deoxyribonucleotide biosynthetic process | 1/105 | 5/17913 | 0.028969937 | 0.127452596 | 0.096182 |
| GO_BP_Profile3 | GO:0010957 | negative regulation of vitamin D biosynthetic process | 1/105 | 5/17913 | 0.028969937 | 0.127452596 | 0.096182 |
| GO_BP_Profile3 | GO:0030263 | apoptotic chromosome condensation | 1/105 | 5/17913 | 0.028969937 | 0.127452596 | 0.096182 |
| GO_BP_Profile3 | GO:0034093 | positive regulation of maintenance of sister chromatid cohesion | 1/105 | 5/17913 | 0.028969937 | 0.127452596 | 0.096182 |
| GO_BP_Profile3 | GO:0034184 | positive regulation of maintenance of mitotic sister chromatid cohesion | 1/105 | 5/17913 | 0.028969937 | 0.127452596 | 0.096182 |
| GO_BP_Profile3 | GO:0042262 | DNA protection | 1/105 | 5/17913 | 0.028969937 | 0.127452596 | 0.096182 |
| GO_BP_Profile3 | GO:0044314 | protein K27-linked ubiquitination | 1/105 | 5/17913 | 0.028969937 | 0.127452596 | 0.096182 |
| GO_BP_Profile3 | GO:0044837 | actomyosin contractile ring organization | 1/105 | 5/17913 | 0.028969937 | 0.127452596 | 0.096182 |
| GO_BP_Profile3 | GO:0048298 | positive regulation of isotype switching to IgA isotypes | 1/105 | 5/17913 | 0.028969937 | 0.127452596 | 0.096182 |
| GO_BP_Profile3 | GO:0106071 | positive regulation of adenylate cyclase-activating G protein-coupled receptor signaling pathway | 1/105 | 5/17913 | 0.028969937 | 0.127452596 | 0.096182 |
| GO_BP_Profile3 | GO:1901563 | response to camptothecin | 1/105 | 5/17913 | 0.028969937 | 0.127452596 | 0.096182 |
| GO_BP_Profile3 | GO:1903722 | regulation of centriole elongation | 1/105 | 5/17913 | 0.028969937 | 0.127452596 | 0.096182 |
| GO_BP_Profile3 | GO:1905448 | positive regulation of mitochondrial ATP synthesis coupled electron transport | 1/105 | 5/17913 | 0.028969937 | 0.127452596 | 0.096182 |
| GO_BP_Profile3 | GO:1905462 | regulation of DNA duplex unwinding | 1/105 | 5/17913 | 0.028969937 | 0.127452596 | 0.096182 |
| GO_BP_Profile3 | GO:1905634 | regulation of protein localization to chromatin | 1/105 | 5/17913 | 0.028969937 | 0.127452596 | 0.096182 |
| GO_BP_Profile3 | GO:1905832 | positive regulation of spindle assembly | 1/105 | 5/17913 | 0.028969937 | 0.127452596 | 0.096182 |
| GO_BP_Profile3 | GO:1900180 | regulation of protein localization to nucleus | 3/105 | 117/17913 | 0.031473016 | 0.13810425 | 0.10422 |
| GO_BP_Profile3 | GO:0051495 | positive regulation of cytoskeleton organization | 4/105 | 205/17913 | 0.03255848 | 0.142420615 | 0.107477 |
| GO_BP_Profile3 | GO:0048193 | Golgi vesicle transport | 5/105 | 304/17913 | 0.033325523 | 0.142420615 | 0.107477 |
| GO_BP_Profile3 | GO:1905515 | non-motile cilium assembly | 2/105 | 49/17913 | 0.033474867 | 0.142420615 | 0.107477 |
| GO_BP_Profile3 | GO:0003284 | septum primum development | 1/105 | 6/17913 | 0.034663379 | 0.142420615 | 0.107477 |
| GO_BP_Profile3 | GO:0003433 | chondrocyte development involved in endochondral bone morphogenesis | 1/105 | 6/17913 | 0.034663379 | 0.142420615 | 0.107477 |
| GO_BP_Profile3 | GO:0009146 | purine nucleoside triphosphate catabolic process | 1/105 | 6/17913 | 0.034663379 | 0.142420615 | 0.107477 |
| GO_BP_Profile3 | GO:0009155 | purine deoxyribonucleotide catabolic process | 1/105 | 6/17913 | 0.034663379 | 0.142420615 | 0.107477 |
| GO_BP_Profile3 | GO:0016321 | female meiosis chromosome segregation | 1/105 | 6/17913 | 0.034663379 | 0.142420615 | 0.107477 |
| GO_BP_Profile3 | GO:0030952 | establishment or maintenance of cytoskeleton polarity | 1/105 | 6/17913 | 0.034663379 | 0.142420615 | 0.107477 |
| GO_BP_Profile3 | GO:0033601 | positive regulation of mammary gland epithelial cell proliferation | 1/105 | 6/17913 | 0.034663379 | 0.142420615 | 0.107477 |
| GO_BP_Profile3 | GO:0035519 | protein K29-linked ubiquitination | 1/105 | 6/17913 | 0.034663379 | 0.142420615 | 0.107477 |
| GO_BP_Profile3 | GO:0045144 | meiotic sister chromatid segregation | 1/105 | 6/17913 | 0.034663379 | 0.142420615 | 0.107477 |
| GO_BP_Profile3 | GO:0045719 | negative regulation of glycogen biosynthetic process | 1/105 | 6/17913 | 0.034663379 | 0.142420615 | 0.107477 |
| GO_BP_Profile3 | GO:0045842 | positive regulation of mitotic metaphase/anaphase transition | 1/105 | 6/17913 | 0.034663379 | 0.142420615 | 0.107477 |
| GO_BP_Profile3 | GO:0046137 | negative regulation of vitamin metabolic process | 1/105 | 6/17913 | 0.034663379 | 0.142420615 | 0.107477 |
| GO_BP_Profile3 | GO:0046601 | positive regulation of centriole replication | 1/105 | 6/17913 | 0.034663379 | 0.142420615 | 0.107477 |
| GO_BP_Profile3 | GO:0046826 | negative regulation of protein export from nucleus | 1/105 | 6/17913 | 0.034663379 | 0.142420615 | 0.107477 |
| GO_BP_Profile3 | GO:0048296 | regulation of isotype switching to IgA isotypes | 1/105 | 6/17913 | 0.034663379 | 0.142420615 | 0.107477 |
| GO_BP_Profile3 | GO:0048478 | replication fork protection | 1/105 | 6/17913 | 0.034663379 | 0.142420615 | 0.107477 |
| GO_BP_Profile3 | GO:0051177 | meiotic sister chromatid cohesion | 1/105 | 6/17913 | 0.034663379 | 0.142420615 | 0.107477 |
| GO_BP_Profile3 | GO:0051661 | maintenance of centrosome location | 1/105 | 6/17913 | 0.034663379 | 0.142420615 | 0.107477 |
| GO_BP_Profile3 | GO:0061511 | centriole elongation | 1/105 | 6/17913 | 0.034663379 | 0.142420615 | 0.107477 |
| GO_BP_Profile3 | GO:0071921 | cohesin loading | 1/105 | 6/17913 | 0.034663379 | 0.142420615 | 0.107477 |
| GO_BP_Profile3 | GO:0090166 | Golgi disassembly | 1/105 | 6/17913 | 0.034663379 | 0.142420615 | 0.107477 |
| GO_BP_Profile3 | GO:1901970 | positive regulation of mitotic sister chromatid separation | 1/105 | 6/17913 | 0.034663379 | 0.142420615 | 0.107477 |
| GO_BP_Profile3 | GO:1903551 | regulation of extracellular exosome assembly | 1/105 | 6/17913 | 0.034663379 | 0.142420615 | 0.107477 |
| GO_BP_Profile3 | GO:0036297 | interstrand cross-link repair | 2/105 | 50/17913 | 0.034738797 | 0.142420615 | 0.107477 |
| GO_BP_Profile3 | GO:2000134 | negative regulation of G1/S transition of mitotic cell cycle | 3/105 | 124/17913 | 0.036447546 | 0.149063385 | 0.11249 |
| GO_BP_Profile3 | GO:1903829 | positive regulation of cellular protein localization | 5/105 | 318/17913 | 0.039245488 | 0.153722911 | 0.116006 |
| GO_BP_Profile3 | GO:1903322 | positive regulation of protein modification by small protein conjugation or removal | 3/105 | 128/17913 | 0.03946107 | 0.153722911 | 0.116006 |
| GO_BP_Profile3 | GO:0002069 | columnar/cuboidal epithelial cell maturation | 1/105 | 7/17913 | 0.040323755 | 0.153722911 | 0.116006 |
| GO_BP_Profile3 | GO:0006265 | DNA topological change | 1/105 | 7/17913 | 0.040323755 | 0.153722911 | 0.116006 |
| GO_BP_Profile3 | GO:0007144 | female meiosis I | 1/105 | 7/17913 | 0.040323755 | 0.153722911 | 0.116006 |
| GO_BP_Profile3 | GO:0009157 | deoxyribonucleoside monophosphate biosynthetic process | 1/105 | 7/17913 | 0.040323755 | 0.153722911 | 0.116006 |
| GO_BP_Profile3 | GO:0009204 | deoxyribonucleoside triphosphate catabolic process | 1/105 | 7/17913 | 0.040323755 | 0.153722911 | 0.116006 |
| GO_BP_Profile3 | GO:0017062 | respiratory chain complex III assembly | 1/105 | 7/17913 | 0.040323755 | 0.153722911 | 0.116006 |
| GO_BP_Profile3 | GO:0021873 | forebrain neuroblast division | 1/105 | 7/17913 | 0.040323755 | 0.153722911 | 0.116006 |
| GO_BP_Profile3 | GO:0031936 | negative regulation of chromatin silencing | 1/105 | 7/17913 | 0.040323755 | 0.153722911 | 0.116006 |
| GO_BP_Profile3 | GO:0033504 | floor plate development | 1/105 | 7/17913 | 0.040323755 | 0.153722911 | 0.116006 |
| GO_BP_Profile3 | GO:0034085 | establishment of sister chromatid cohesion | 1/105 | 7/17913 | 0.040323755 | 0.153722911 | 0.116006 |
| GO_BP_Profile3 | GO:0034551 | mitochondrial respiratory chain complex III assembly | 1/105 | 7/17913 | 0.040323755 | 0.153722911 | 0.116006 |
| GO_BP_Profile3 | GO:0035405 | histone-threonine phosphorylation | 1/105 | 7/17913 | 0.040323755 | 0.153722911 | 0.116006 |
| GO_BP_Profile3 | GO:0040016 | embryonic cleavage | 1/105 | 7/17913 | 0.040323755 | 0.153722911 | 0.116006 |
| GO_BP_Profile3 | GO:0042996 | regulation of Golgi to plasma membrane protein transport | 1/105 | 7/17913 | 0.040323755 | 0.153722911 | 0.116006 |
| GO_BP_Profile3 | GO:0048290 | isotype switching to IgA isotypes | 1/105 | 7/17913 | 0.040323755 | 0.153722911 | 0.116006 |
| GO_BP_Profile3 | GO:0051305 | chromosome movement towards spindle pole | 1/105 | 7/17913 | 0.040323755 | 0.153722911 | 0.116006 |
| GO_BP_Profile3 | GO:0060558 | regulation of calcidiol 1-monooxygenase activity | 1/105 | 7/17913 | 0.040323755 | 0.153722911 | 0.116006 |
| GO_BP_Profile3 | GO:0070092 | regulation of glucagon secretion | 1/105 | 7/17913 | 0.040323755 | 0.153722911 | 0.116006 |
| GO_BP_Profile3 | GO:0070874 | negative regulation of glycogen metabolic process | 1/105 | 7/17913 | 0.040323755 | 0.153722911 | 0.116006 |
| GO_BP_Profile3 | GO:0071971 | extracellular exosome assembly | 1/105 | 7/17913 | 0.040323755 | 0.153722911 | 0.116006 |
| GO_BP_Profile3 | GO:0072383 | plus-end-directed vesicle transport along microtubule | 1/105 | 7/17913 | 0.040323755 | 0.153722911 | 0.116006 |
| GO_BP_Profile3 | GO:0090232 | positive regulation of spindle checkpoint | 1/105 | 7/17913 | 0.040323755 | 0.153722911 | 0.116006 |
| GO_BP_Profile3 | GO:0090235 | regulation of metaphase plate congression | 1/105 | 7/17913 | 0.040323755 | 0.153722911 | 0.116006 |
| GO_BP_Profile3 | GO:0090267 | positive regulation of mitotic cell cycle spindle assembly checkpoint | 1/105 | 7/17913 | 0.040323755 | 0.153722911 | 0.116006 |
| GO_BP_Profile3 | GO:0090435 | protein localization to nuclear envelope | 1/105 | 7/17913 | 0.040323755 | 0.153722911 | 0.116006 |
| GO_BP_Profile3 | GO:1900193 | regulation of oocyte maturation | 1/105 | 7/17913 | 0.040323755 | 0.153722911 | 0.116006 |
| GO_BP_Profile3 | GO:1901857 | positive regulation of cellular respiration | 1/105 | 7/17913 | 0.040323755 | 0.153722911 | 0.116006 |
| GO_BP_Profile3 | GO:1902101 | positive regulation of metaphase/anaphase transition of cell cycle | 1/105 | 7/17913 | 0.040323755 | 0.153722911 | 0.116006 |
| GO_BP_Profile3 | GO:0019882 | antigen processing and presentation | 4/105 | 220/17913 | 0.040530555 | 0.154162494 | 0.116338 |
| GO_BP_Profile3 | GO:0006261 | DNA-dependent DNA replication | 3/105 | 130/17913 | 0.041014016 | 0.155300263 | 0.117197 |
| GO_BP_Profile3 | GO:1902807 | negative regulation of cell cycle G1/S phase transition | 3/105 | 130/17913 | 0.041014016 | 0.155300263 | 0.117197 |
| GO_BP_Profile3 | GO:0016445 | somatic diversification of immunoglobulins | 2/105 | 55/17913 | 0.041328763 | 0.156141177 | 0.117831 |
| GO_BP_Profile3 | GO:0034504 | protein localization to nucleus | 4/105 | 223/17913 | 0.042247009 | 0.159253267 | 0.12018 |
| GO_BP_Profile3 | GO:0048565 | digestive tract development | 3/105 | 132/17913 | 0.042597548 | 0.160216225 | 0.120907 |
| GO_BP_Profile3 | GO:0022412 | cellular process involved in reproduction in multicellular organism | 5/105 | 326/17913 | 0.042899695 | 0.160993286 | 0.121493 |
| GO_BP_Profile3 | GO:0000733 | DNA strand renaturation | 1/105 | 8/17913 | 0.045951254 | 0.163695271 | 0.123532 |
| GO_BP_Profile3 | GO:0006268 | DNA unwinding involved in DNA replication | 1/105 | 8/17913 | 0.045951254 | 0.163695271 | 0.123532 |
| GO_BP_Profile3 | GO:0007135 | meiosis II | 1/105 | 8/17913 | 0.045951254 | 0.163695271 | 0.123532 |
| GO_BP_Profile3 | GO:0009265 | 2'-deoxyribonucleotide biosynthetic process | 1/105 | 8/17913 | 0.045951254 | 0.163695271 | 0.123532 |
| GO_BP_Profile3 | GO:0014807 | regulation of somitogenesis | 1/105 | 8/17913 | 0.045951254 | 0.163695271 | 0.123532 |
| GO_BP_Profile3 | GO:0031441 | negative regulation of mRNA 3'-end processing | 1/105 | 8/17913 | 0.045951254 | 0.163695271 | 0.123532 |
| GO_BP_Profile3 | GO:0031666 | positive regulation of lipopolysaccharide-mediated signaling pathway | 1/105 | 8/17913 | 0.045951254 | 0.163695271 | 0.123532 |
| GO_BP_Profile3 | GO:0034091 | regulation of maintenance of sister chromatid cohesion | 1/105 | 8/17913 | 0.045951254 | 0.163695271 | 0.123532 |
| GO_BP_Profile3 | GO:0034182 | regulation of maintenance of mitotic sister chromatid cohesion | 1/105 | 8/17913 | 0.045951254 | 0.163695271 | 0.123532 |
| GO_BP_Profile3 | GO:0034351 | negative regulation of glial cell apoptotic process | 1/105 | 8/17913 | 0.045951254 | 0.163695271 | 0.123532 |
| GO_BP_Profile3 | GO:0046385 | deoxyribose phosphate biosynthetic process | 1/105 | 8/17913 | 0.045951254 | 0.163695271 | 0.123532 |
| GO_BP_Profile3 | GO:0048537 | mucosal-associated lymphoid tissue development | 1/105 | 8/17913 | 0.045951254 | 0.163695271 | 0.123532 |
| GO_BP_Profile3 | GO:0048541 | Peyer's patch development | 1/105 | 8/17913 | 0.045951254 | 0.163695271 | 0.123532 |
| GO_BP_Profile3 | GO:0061983 | meiosis II cell cycle process | 1/105 | 8/17913 | 0.045951254 | 0.163695271 | 0.123532 |
| GO_BP_Profile3 | GO:0070091 | glucagon secretion | 1/105 | 8/17913 | 0.045951254 | 0.163695271 | 0.123532 |
| GO_BP_Profile3 | GO:0072386 | plus-end-directed organelle transport along microtubule | 1/105 | 8/17913 | 0.045951254 | 0.163695271 | 0.123532 |
| GO_BP_Profile3 | GO:0075713 | establishment of integrated proviral latency | 1/105 | 8/17913 | 0.045951254 | 0.163695271 | 0.123532 |
| GO_BP_Profile3 | GO:1902857 | positive regulation of non-motile cilium assembly | 1/105 | 8/17913 | 0.045951254 | 0.163695271 | 0.123532 |
| GO_BP_Profile3 | GO:1903431 | positive regulation of cell maturation | 1/105 | 8/17913 | 0.045951254 | 0.163695271 | 0.123532 |
| GO_BP_Profile3 | GO:1903862 | positive regulation of oxidative phosphorylation | 1/105 | 8/17913 | 0.045951254 | 0.163695271 | 0.123532 |
| GO_BP_Profile3 | GO:1904776 | regulation of protein localization to cell cortex | 1/105 | 8/17913 | 0.045951254 | 0.163695271 | 0.123532 |
| GO_BP_Profile3 | GO:1905168 | positive regulation of double-strand break repair via homologous recombination | 1/105 | 8/17913 | 0.045951254 | 0.163695271 | 0.123532 |
| GO_BP_Profile3 | GO:1905446 | regulation of mitochondrial ATP synthesis coupled electron transport | 1/105 | 8/17913 | 0.045951254 | 0.163695271 | 0.123532 |
| GO_BP_Profile3 | GO:1905820 | positive regulation of chromosome separation | 1/105 | 8/17913 | 0.045951254 | 0.163695271 | 0.123532 |
| GO_BP_Profile3 | GO:1903902 | positive regulation of viral life cycle | 2/105 | 59/17913 | 0.046910243 | 0.166758987 | 0.125844 |
| GO_BP_Profile3 | GO:0018105 | peptidyl-serine phosphorylation | 4/105 | 231/17913 | 0.047023325 | 0.166809057 | 0.125882 |
| GO_BP_Profile3 | GO:0060271 | cilium assembly | 5/105 | 335/17913 | 0.047249164 | 0.16725807 | 0.126221 |
| GO_BP_Profile3 | GO:0051258 | protein polymerization | 4/105 | 232/17913 | 0.047640709 | 0.168290553 | 0.127 |
