# Supplementary material for: Multiomics global landscape of stemness-related gene clusters in adipose-derived mesenchymal stem cells
Source: Stem Cell Res Ther. 2020 Jul 22;11:310. doi: 10.1186/s13287-020-01823-3 (PMC7374825; doi:10.1186/s13287-020-01823-3)
Supplement: Supplementary file 4 — Additional file 4 : Table S4. KEGG pathways enriched in AD-MSCs. AD-MSCs: adipose-derived mesenchymal cells. [file 13287_2020_1823_MOESM4_ESM.docx]

| Table S4. KEGG pathways enriched in AD-MSCs. | | | | | | |
| --- | --- | --- | --- | --- | --- | --- |
| Module | ID | Description | GeneRatio | BgRatio | pvalue | p.adjust |
| KEGG_Pathway_Profile0 | hsa05322 | Systemic lupus erythematosus | 24/112 | 133/7946 | 1.41E-20 | 2.42E-18 |
| KEGG_Pathway_Profile0 | hsa05034 | Alcoholism | 25/112 | 184/7946 | 2.71E-18 | 2.33E-16 |
| KEGG_Pathway_Profile0 | hsa03030 | DNA replication | 12/112 | 36/7946 | 3.18E-14 | 1.82E-12 |
| KEGG_Pathway_Profile0 | hsa04110 | Cell cycle | 15/112 | 124/7946 | 1.48E-10 | 6.36E-09 |
| KEGG_Pathway_Profile0 | hsa03430 | Mismatch repair | 7/112 | 23/7946 | 1.86E-08 | 6.41E-07 |
| KEGG_Pathway_Profile0 | hsa05203 | Viral carcinogenesis | 15/112 | 201/7946 | 1.19E-07 | 3.42E-06 |
| KEGG_Pathway_Profile0 | hsa03410 | Base excision repair | 6/112 | 33/7946 | 5.57E-06 | 0.000137 |
| KEGG_Pathway_Profile0 | hsa03420 | Nucleotide excision repair | 6/112 | 47/7946 | 4.6E-05 | 0.000989 |
| KEGG_Pathway_Profile0 | hsa04217 | Necroptosis | 10/112 | 162/7946 | 8.9E-05 | 0.001702 |
| KEGG_Pathway_Profile0 | hsa03460 | Fanconi anemia pathway | 6/112 | 54/7946 | 0.000102 | 0.001756 |
| KEGG_Pathway_Profile0 | hsa04114 | Oocyte meiosis | 8/112 | 128/7946 | 0.000426 | 0.006663 |
| KEGG_Pathway_Profile0 | hsa04914 | Progesterone-mediated oocyte maturation | 7/112 | 99/7946 | 0.000469 | 0.006723 |
| KEGG_Pathway_Profile0 | hsa04540 | Gap junction | 5/112 | 88/7946 | 0.007882 | 0.104283 |
| KEGG_Pathway_Profile0 | hsa05166 | Human T-cell leukemia virus 1 infection | 8/112 | 219/7946 | 0.011979 | 0.147166 |
| KEGG_Pathway_Profile0 | hsa03440 | Homologous recombination | 3/112 | 41/7946 | 0.019708 | 0.22598 |
| KEGG_Pathway_Profile0 | hsa04218 | Cellular senescence | 6/112 | 160/7946 | 0.02526 | 0.269578 |
| KEGG_Pathway_Profile0 | hsa05161 | Hepatitis B | 6/112 | 162/7946 | 0.026644 | 0.269578 |
| KEGG_Pathway_Profile0 | hsa04210 | Apoptosis | 5/112 | 136/7946 | 0.042778 | 0.40674 |
| KEGG_Pathway_Profile0 | hsa00240 | Pyrimidine metabolism | 3/112 | 57/7946 | 0.046063 | 0.40674 |
| KEGG_Pathway_Profile0 | hsa05202 | Transcriptional misregulation in cancer | 6/112 | 186/7946 | 0.047295 | 0.40674 |
| KEGG_Pathway_Profile11 | hsa05133 | Pertussis | 3月16日 | 76/7946 | 0.000431 | 0.013448 |
| KEGG_Pathway_Profile11 | hsa05412 | Arrhythmogenic right ventricular cardiomyopathy (ARVC) | 3月16日 | 77/7946 | 0.000447 | 0.013448 |
| KEGG_Pathway_Profile11 | hsa05410 | Hypertrophic cardiomyopathy (HCM) | 3月16日 | 90/7946 | 0.000707 | 0.013448 |
| KEGG_Pathway_Profile11 | hsa05414 | Dilated cardiomyopathy (DCM) | 3月16日 | 96/7946 | 0.000854 | 0.013448 |
| KEGG_Pathway_Profile11 | hsa04640 | Hematopoietic cell lineage | 3月16日 | 99/7946 | 0.000934 | 0.013448 |
| KEGG_Pathway_Profile11 | hsa04060 | Cytokine-cytokine receptor interaction | 4月16日 | 294/7946 | 0.002349 | 0.028183 |
| KEGG_Pathway_Profile11 | hsa04512 | ECM-receptor interaction | 2月16日 | 88/7946 | 0.013156 | 0.11934 |
| KEGG_Pathway_Profile11 | hsa05323 | Rheumatoid arthritis | 2月16日 | 93/7946 | 0.014617 | 0.11934 |
| KEGG_Pathway_Profile11 | hsa04657 | IL-17 signaling pathway | 2月16日 | 94/7946 | 0.014918 | 0.11934 |
| KEGG_Pathway_Profile11 | hsa04270 | Vascular smooth muscle contraction | 2月16日 | 132/7946 | 0.028226 | 0.191296 |
| KEGG_Pathway_Profile11 | hsa05418 | Fluid shear stress and atherosclerosis | 2月16日 | 139/7946 | 0.031057 | 0.191296 |
| KEGG_Pathway_Profile11 | hsa04151 | PI3K-Akt signaling pathway | 3月16日 | 354/7946 | 0.031883 | 0.191296 |
| KEGG_Pathway_Profile12 | hsa04514 | Cell adhesion molecules (CAMs) | 3月24日 | 147/7946 | 0.009444 | 0.321316 |
| KEGG_Pathway_Profile12 | hsa04530 | Tight junction | 3月24日 | 169/7946 | 0.013778 | 0.321316 |
| KEGG_Pathway_Profile12 | hsa04137 | Mitophagy - animal | 2月24日 | 65/7946 | 0.016196 | 0.321316 |
| KEGG_Pathway_Profile12 | hsa05120 | Epithelial cell signaling in Helicobacter pylori infection | 2月24日 | 70/7946 | 0.018634 | 0.321316 |
| KEGG_Pathway_Profile12 | hsa04350 | TGF-beta signaling pathway | 2月24日 | 94/7946 | 0.032279 | 0.321316 |
| KEGG_Pathway_Profile12 | hsa04974 | Protein digestion and absorption | 2月24日 | 95/7946 | 0.032913 | 0.321316 |
| KEGG_Pathway_Profile12 | hsa00604 | Glycosphingolipid biosynthesis - ganglio series | 1月24日 | 15/7946 | 0.044399 | 0.321316 |
| KEGG_Pathway_Profile12 | hsa04670 | Leukocyte transendothelial migration | 2月24日 | 112/7946 | 0.044424 | 0.321316 |
| KEGG_Pathway_Profile15 | hsa04614 | Renin-angiotensin system | 2/49 | 23/7946 | 0.008678 | 0.530688 |
| KEGG_Pathway_Profile15 | hsa05418 | Fluid shear stress and atherosclerosis | 4/49 | 139/7946 | 0.010336 | 0.530688 |
| KEGG_Pathway_Profile15 | hsa05323 | Rheumatoid arthritis | 3/49 | 93/7946 | 0.0194 | 0.530688 |
| KEGG_Pathway_Profile15 | hsa00250 | Alanine, aspartate and glutamate metabolism | 2/49 | 36/7946 | 0.020539 | 0.530688 |
| KEGG_Pathway_Profile15 | hsa00400 | Phenylalanine, tyrosine and tryptophan biosynthesis | 1/49 | 5/7946 | 0.030463 | 0.530688 |
| KEGG_Pathway_Profile15 | hsa04510 | Focal adhesion | 4/49 | 199/7946 | 0.033663 | 0.530688 |
| KEGG_Pathway_Profile15 | hsa00270 | Cysteine and methionine metabolism | 2/49 | 49/7946 | 0.03645 | 0.530688 |
| KEGG_Pathway_Profile15 | hsa04913 | Ovarian steroidogenesis | 2/49 | 49/7946 | 0.03645 | 0.530688 |
| KEGG_Pathway_Profile3 | hsa04110 | Cell cycle | 8/44 | 124/7946 | 3.1E-07 | 3.45E-05 |
| KEGG_Pathway_Profile3 | hsa04114 | Oocyte meiosis | 6/44 | 128/7946 | 6.64E-05 | 0.00264 |
| KEGG_Pathway_Profile3 | hsa03440 | Homologous recombination | 4/44 | 41/7946 | 7.13E-05 | 0.00264 |
| KEGG_Pathway_Profile3 | hsa04914 | Progesterone-mediated oocyte maturation | 5/44 | 99/7946 | 0.0002 | 0.005562 |
| KEGG_Pathway_Profile3 | hsa04115 | p53 signaling pathway | 4/44 | 72/7946 | 0.00064 | 0.014204 |
| KEGG_Pathway_Profile3 | hsa03030 | DNA replication | 2/44 | 36/7946 | 0.016757 | 0.309997 |
| KEGG_Pathway_Profile3 | hsa00480 | Glutathione metabolism | 2/44 | 56/7946 | 0.03821 | 0.547543 |
| KEGG_Pathway_Profile3 | hsa00240 | Pyrimidine metabolism | 2/44 | 57/7946 | 0.039463 | 0.547543 |
